# Computer-Aided Discovery of Small-Molecule Inhibitors of Pathogenic New World Arenavirus Entry and Replication

**DOI:** 10.1021/acsinfecdis.6c00138

**Published:** 2026-05-04

**Authors:** Samantha Rae Wasson, Ben Matthew Flude, Martina Salerno, Kie Hoon Jung, Gilda Padalino, Salvatore Ferla, Dylan Joseph Roche-Dugmore, Connor W Bott, Andrea Brancale, Brian B. Gowen, Marcella Bassetto

**Affiliations:** 1 Institute for Antiviral Research, 4606 Utah State University, Logan, Utah 84322, United States; 2 Department of Chemistry, College of Science and Engineering, 7759 Swansea University, Swansea SA2 8PP, U.K.; 3 Medical School, Faculty of Medicine, Health and Life Science, 7759 Swansea University, Swansea SA2 8PP, U.K.; 4 Department of Organic Chemistry, University of Chemistry and Technology, Prague, Prague 16628, Czech Republic; 5 School of Pharmacy and Pharmaceutical Sciences, 170810 Cardiff University, Cardiff CF10 3NB, U.K.

**Keywords:** small-molecule antivirals, New World arenaviruses, transferrin receptor 1 (TfR1), computer-aided drug design (CADD), medicinal chemistry

## Abstract

Pathogenic New World arenaviruses (NWAs), including Junín (JUNV) and Machupo (MACV) viruses, rely on host–virus entry processes that represent attractive points for antiviral intervention. Guided by the known use of human transferrin receptor 1 (hTfR1) by several NWAs for cell entry, we conducted a structure-based virtual screening campaign targeting the MACV GP1-hTfR1 interaction interface to identify small molecules capable of inhibiting early infection. From an *in silico* screen of commercially available drug-like compounds, 25 candidates were selected and tested in cell-based assays, yielding two chemically distinct scaffolds with low-micromolar activity against JUNV. Hit expansion of the primary chemotype produced 107 new analogues, several of which achieved submicromolar inhibition of JUNV replication. Among them, compound **22f** demonstrated antiviral activity across multiple arenaviruses, including both hTfR1-tropic NWAs and viruses that use alternative entry pathways, while showing no effect on the unrelated Rift Valley fever virus. In an hTfR1-expressing mouse model of JUNV infection, **22f** was well tolerated, but did not confer protection. These results provide the foundation for further development and optimization of potent compounds that broadly inhibit infection by the pathogenic NWAs.

## Graphical abstract

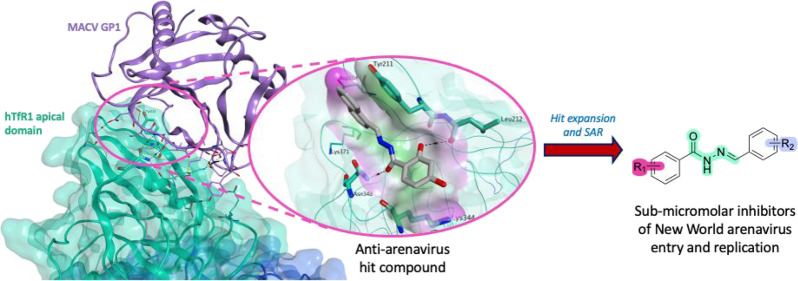

## 1 Introduction

The *Mammarenavirus* genus within the *Arenaviridae* family comprises rodent–borne, enveloped, single–stranded RNA viruses classified into Old World (OW) and New World (NW) species, several of which cause severe arenaviral hemorrhagic fevers (AHFs) in humans.^1^ Transmission typically occurs through exposure to contaminated rodent material, with occasional person–to–person spread, and disease severity ranges from mild to fulminant multisystem illness.^2^ Among OW arenaviruses, Lassa virus (LASV) is responsible for substantial morbidity and mortality in West Africa, with an estimated 100,000–300,000 infections and ∼5000 deaths annually.^3,4^ Although its overall case fatality rate is ∼1%, hospitalised patients experience much higher mortality and frequent long–term sequelae.^3,5–7^ In South America, pathogenic NW arenaviruses, including Junín (JUNV), Machupo (MACV), Guanarito (GTOV), Sabiá (SBAV), and Chapare (CHAPV), cause sporadic but highly lethal outbreaks, with reported case fatality rates of 30–60%.^8,9^ Despite the successful use of the Candid#1 vaccine against JUNV in Argentina,^10–12^ no FDA–approved antivirals or broadly effective vaccines exist for any arenavirus. Ribavirin remains used off–label for LASV,^13^ but concerns about efficacy and toxicity persist.^14–16^ Experimental therapeutics including favipiravir,^17,18^ viral entry inhibitors,^19^ and monoclonal antibodies,^20,21^ show promise but are limited by virus specificity, resistance potential, or scalability challenges. These shortcomings highlight the need for host–directed antiviral strategies with broader applicability across pathogenic arenaviruses.

A central entry determinant for pathogenic NW arenaviruses is human transferrin receptor 1 (hTfR1), which all known pathogenic clade B viruses utilize for host–cell entry, whereas nonpathogenic NW strains use TfR1–independent mechanisms.^9,22–24^ Species–specific susceptibility correlates with TfR1 engagement: animals whose TfR1 orthologs permit viral entry (e.g., guinea pigs, marmosets, macaques) are susceptible, while those with non–permissive orthologs (e.g., laboratory mice, hamsters) are resistant.^22,25,26^ These relationships support TfR1 as a determinant of host susceptibility and indicate that hTfR1 interaction is a hallmark of pathogenic NWAs.^27^ At the molecular level, the arenavirus GP1 subunit binds the apical domain of hTfR1,^22^ a region spatially distinct from the physiological binding sites for transferrin (Tf) and hereditary hemochromatosis protein (HFE).^28,29^ This makes the GP1-hTfR1 interface an attractive surface for therapeutic intervention. Antibody studies validate this interface as targetable: ch128.1 binds the apical domain, blocks GP1 attachment, and protects hTfR1–expressing mice from JUNV infection.^25,30^ While monoclonal antibodies demonstrate feasibility, small molecules would offer several advantages, including oral dosing and large–scale manufacture, while still reducing resistance risk by targeting a host–factor interaction.^31^

Here, we report a structure–guided virtual screening campaign targeting the MACV GP1-hTfR1 interface, from which we identified two chemical scaffolds with low–micromolar activity against JUNV. We then optimized the primary scaffold through synthesis and SAR analysis of 107 analogues, yielding several sub–micromolar inhibitors. Lead compound **22f** also blocked MACV and GTOV entry and inhibited early and early postentry infection steps, providing a strong foundation for developing small–molecule arenavirus therapeutics.

## 2 Results and Discussion

### 2.1 Structure-Based Virtual Screening for Small-Molecule Inhibitors of the GP1-hTfR1 Interaction

The crystal structure of the complex between GP1 and hTfR1 has been resolved for one representative NWA, MACV,^30^ providing a solid starting point to identify potential small-molecule inhibitors. Importantly, the viral GP1 binds to the apical domain of the extracellular portion of the receptor, in a distinct region to the binding areas of the major natural ligands, Tf,^32^ and HFE,^29^ as illustrated in Figure S1 (Supporting Information).

The contact surface between hTfR1 and MACV GP1 was analyzed with Molecular Operating Environment (MOE),^33^ and a druggable site was identified within the hTfR1 surface interacting with GP1 (Figure 1). This site is centered around residues Tyr211 and Asn348, crucial for binding with GP1 and subsequent GP-mediated viral entry into host cells.^22^

**Figure 1:**
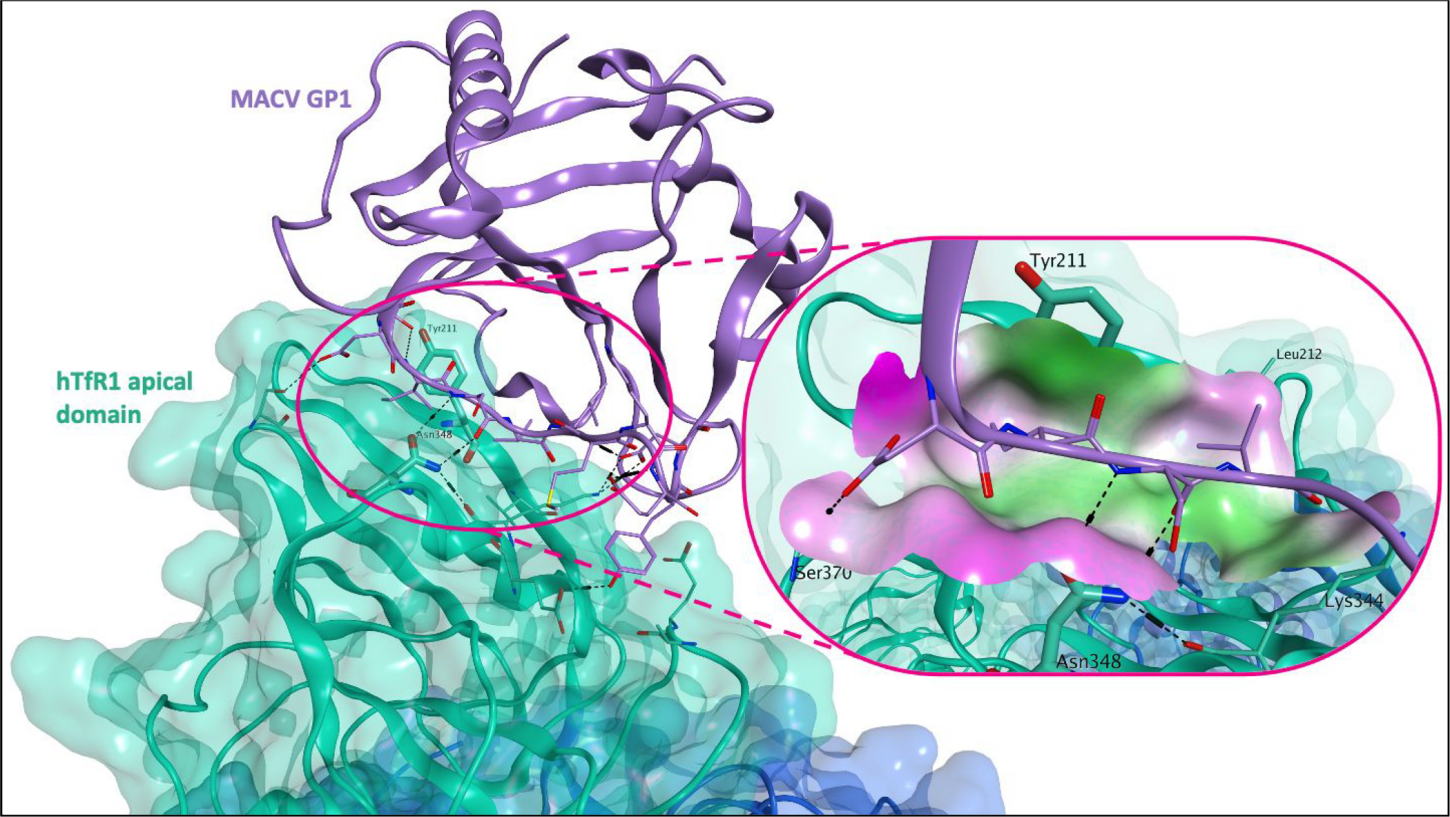
Zoom on the GP1 binding area on the hTfR1 apical domain in the 3KAS crystal structure, with a druggable subpocket, centered around Tyr211 and Asn348, highlighted with a hydrophilic/lipophilic molecular surface (pink = hydrophilic, green = lipophilic, and white = neutral). The apical domain of hTfR1 is represented as a green ribbon and a green molecular surface, with the carbon atoms of interacting residues shown in green. MACV GP1 is represented as a lilac ribbon with carbon atoms of interacting residues in lilac. Nonbonded interactions between GP1 and hTfR1 are shown as black dotted lines.

This site was used for structure-based virtual screening of the SPECS library, comprising ∼300,000 commercially available drug-like compounds.^34^ As an initial filter, Glide High-Throughput Virtual Screening (HTVS)^35^ was applied using the Glide-HTVS scoring function. The top 10% of ranked compounds were subsequently redocked into the selected site using Glide Standard Precision (SP). The resulting Glide SP poses were then rescored using three scoring functions in accordance with a protocol previously optimized in our laboratory:^36,37^ Glide XP, CHEMPLP (PLANTS) and FlexX Score (SeeSAR).^35,38,39^ After combining the three scoring outputs (see Section 4.1), 1288 molecules were shortlisted and visually inspected for plausible fit and interactions within the selected site. From this set, 25 virtual hits were selected (Supporting Information, Figure S2), purchased, and evaluated for inhibition of JUNV replication in a virus yield reduction (VYR) assay (Supporting Information, Table S1).

This initial biological evaluation identified two chemically diverse scaffolds (**1a** and **2**; Figure 2a) as promising antiviral hits, with mean EC_90_ values of 2.8 and 1.0 μM, respectively (Table S1), and no associated cytotoxicity. The present work focuses on hit expansion, structure–activity relationship (SAR) analysis, and antiviral evaluation of scaffold **1a**. Although the antiviral properties of scaffold **2** were also thoroughly investigated, structural optimization of that chemotype will be reported separately.

**Figure 2:**
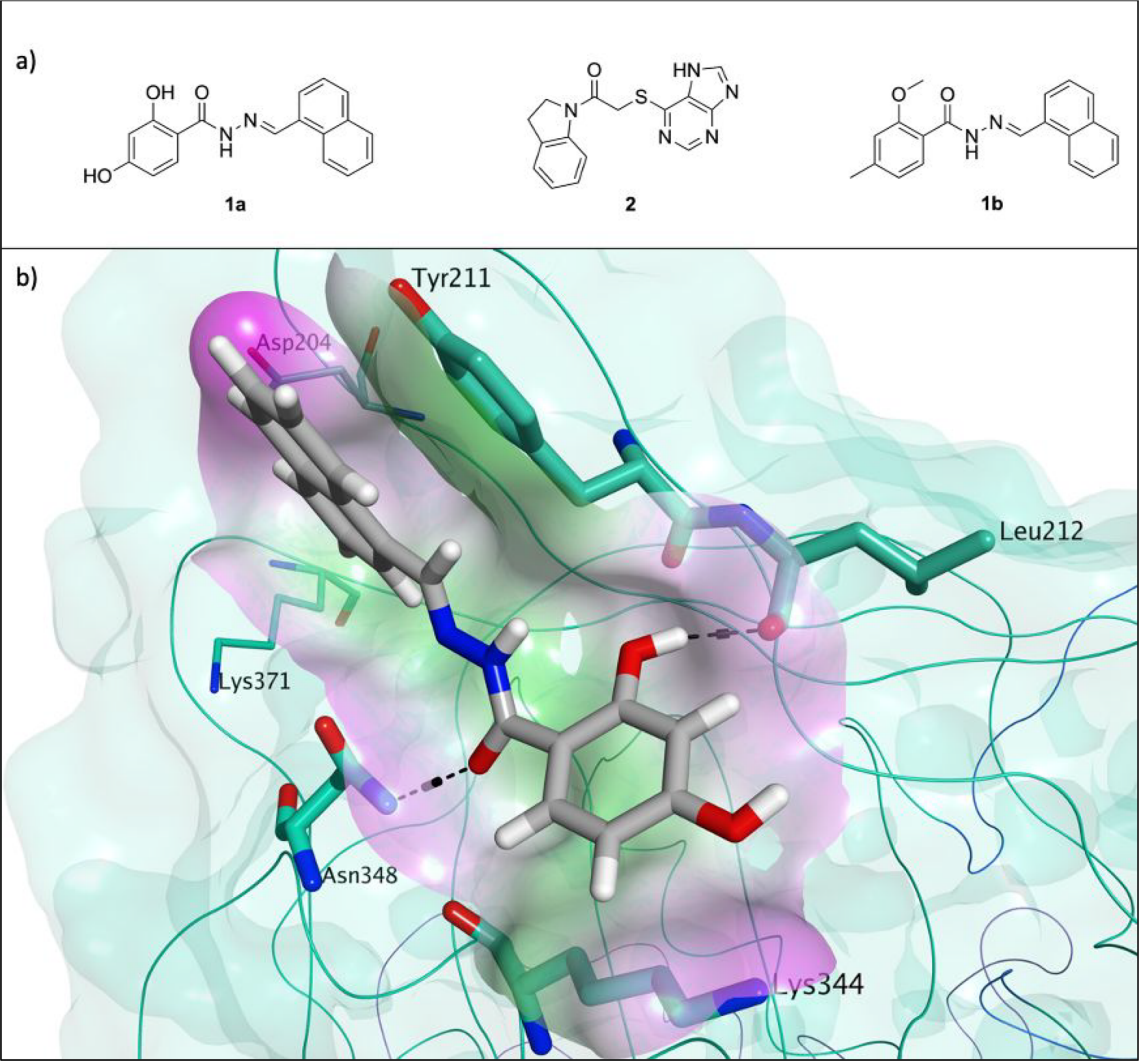
(a) Chemical structures of our initial antiviral hits **1a**, **2**, and **1b**. (b) Predicted binding of **1a** to the target binding pocket of the MACV GP1-hTfR1 complex crystal structure 3KAS, evaluated with the molecular docking program Glide SP. Hit **1a** shows an optimal fitting of the target binding site, with the possibility of forming a direct π–π stacking interaction with key residue Tyr211, a hydrogen bond with the side chain of key residue Asn348, and an additional hydrogen bond with the backbone carbonyl group of Leu212. The apical domain of hTfR1 is represented as a green ribbon and a green molecular surface, with the carbon atoms of interacting residues shown in green. The druggable subpocket area centered around Tyr211 and Asn348 is highlighted with a hydrophilic/lipophilic molecular surface (pink = hydrophilic, green = lipophilic, and white = neutral). Compound **1a** is represented with carbon atoms in gray. Hydrogen bonds between **1a** and hTfR1 are shown as black dotted lines.

Before initiating a full hit-expansion campaign around **1a**, the antiviral potential of this scaffold was further assessed by acquiring six commercially available structural analogues (Supporting Information, Figure S3), selected primarily based on availability from SPECS. These analogues were evaluated in the JUNV VYR assay (Supporting Table S1). Multiple analogues retained low-micromolar antiviral activity. Among these commercial compounds, **1b** (Figure 2a and Supporting Figure S3) showed the most favorable antiviral profile, with a mean EC_90_ value of 3.3 μM.

The Glide SP docking pose obtained for **1a** during the virtual screening workflow is shown in Figure 2b. In this predicted binding mode, **1a** occupies the selected region on hTfR1, positioning the naphthyl ring at a π–π stacking distance from the Tyr211 side chain, and forming two predicted hydrogen-bond interactions: one between the ligand carbonyl oxygen and the Asn348 side chain, and a second between the ortho-hydroxy group and the Leu212 backbone carbonyl. In addition, the side chain of Lys344 lies near the para–substituted region of **1a**. Although no well–defined hydrogen bond is predicted in the docking pose, its cationic ε-amino group may act as hydrogen-bond donor to the para–OH group of **1a**, and may also engage in a cation−π interaction with **1a** aromatic ring, potentially contributing to further ligand stabilization within the pocket.

### 2.2 Design and Synthesis of Hit **1a** Analogues to Explore SAR

Following confirmation of the antiviral potential of scaffold **1a**/**1b**, we designed a focused analogue series to define structure–activity relationships (SAR) and to improve antiviral profiles and predicted pharmacokinetic (PK) properties. The principal structural variations are summarized in Figure 3.

**Figure 3:**
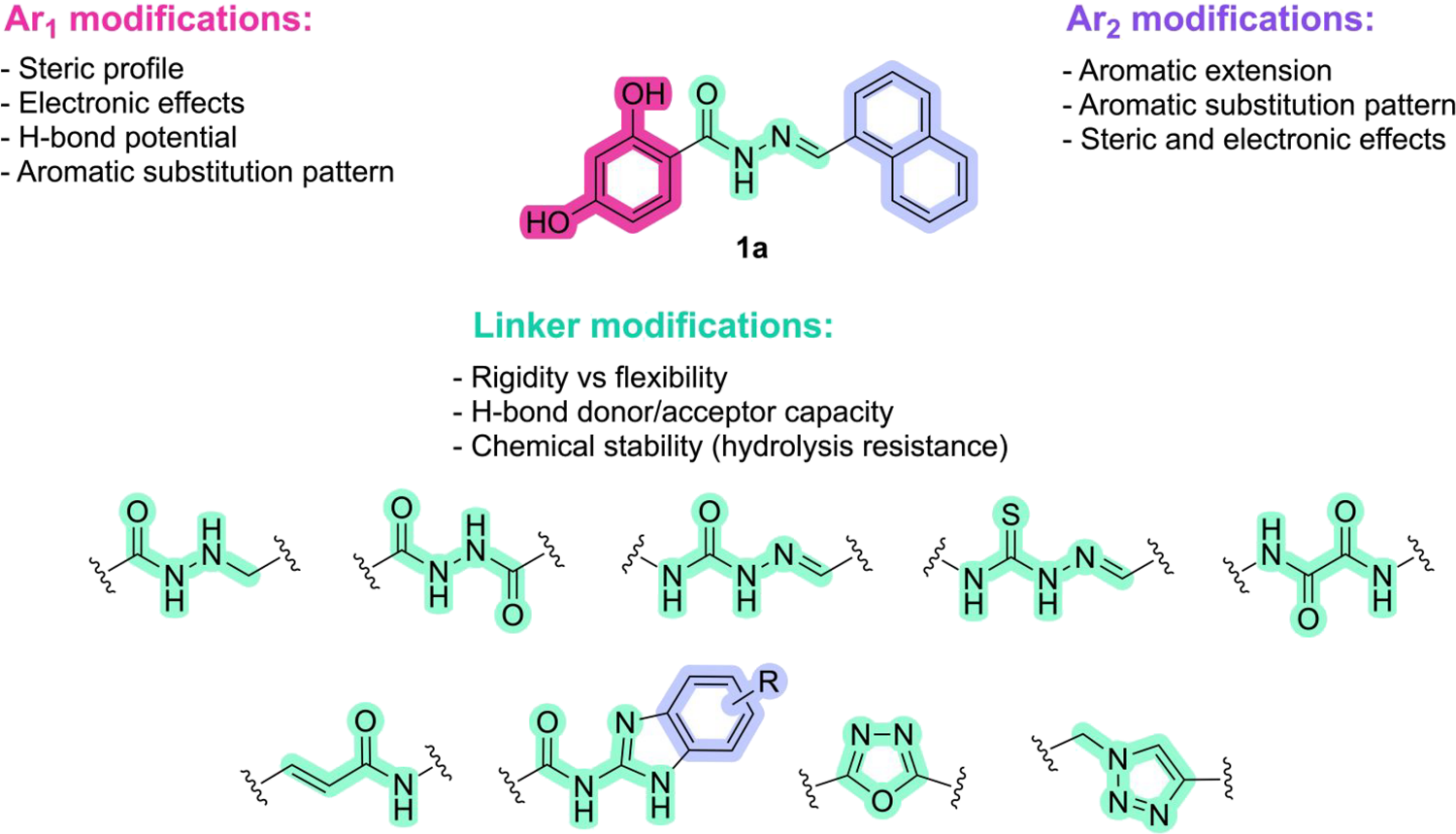
Planned structural modifications to the scaffold of hit **1a**. The sections of **1a** that were systematically modified are highlighted: the 2,4-dihydroxyphenyl group (Ar_1_) in red, the 1-naphthyl group (Ar_2_) in lilac, and the central hydrazone linker in green. While different aromatic rings and aromatic substituents were systematically explored for Ar_1_ and Ar_2_, selected rational modifications were carried out for the linker portion.

Scaffold **1a** comprises three structural elements: a 2,4-dihydroxyphenyl ring (Ar_1_), a central hydrazone linker, and a 1–naphthyl group (Ar_2_). We systematically modified Ar_1_ and Ar_2_ to probe the impact of steric size, polarity, and aromatic extension, and to assess positional effects of substituents on antiviral activity. In parallel, we explored linker replacements, to vary rigidity/flexibility and hydrogen-bonding capacity, and to address potential aqueous instability of the hydrazone motif, which is susceptible to hydrolysis (Figure 3).^40^ These efforts generated families of analogues incorporating reduced linkers, benzoylhydrazides, ureas, thioureas, oxalic amides, acrylamides, and heterocyclic linkers. Hydrazone analogues were prepared via standard ester–to–hydrazide–to–hydrazone formation sequences, while linker–modified derivatives were obtained through established reduction, acylation, coupling, or heterocycle–forming reactions,^41–44^ as described in Schemes S1–S4 in the Supporting Information.

### 2.3 Biological Assays

#### 2.3.1 Virus Yield Reduction (VYR) Antiviral and Cytotoxicity Studies

All the new target molecules, along with resynthesized **1a**, were evaluated in a VYR assay based on JUNV infection of A549 human lung epithelial cells treated with escalating concentrations of compounds and measuring replication by end point titration of infectious virus present in culture supernatants.^45^ Cytotoxicity was determined in parallel in uninfected, compound-treated A549 cells. Ribavirin was used as the positive control for all evaluations.^45^ The results obtained from this screening are shown in Table 1. Data for the most active analogues are in bold.

**Table 1:**
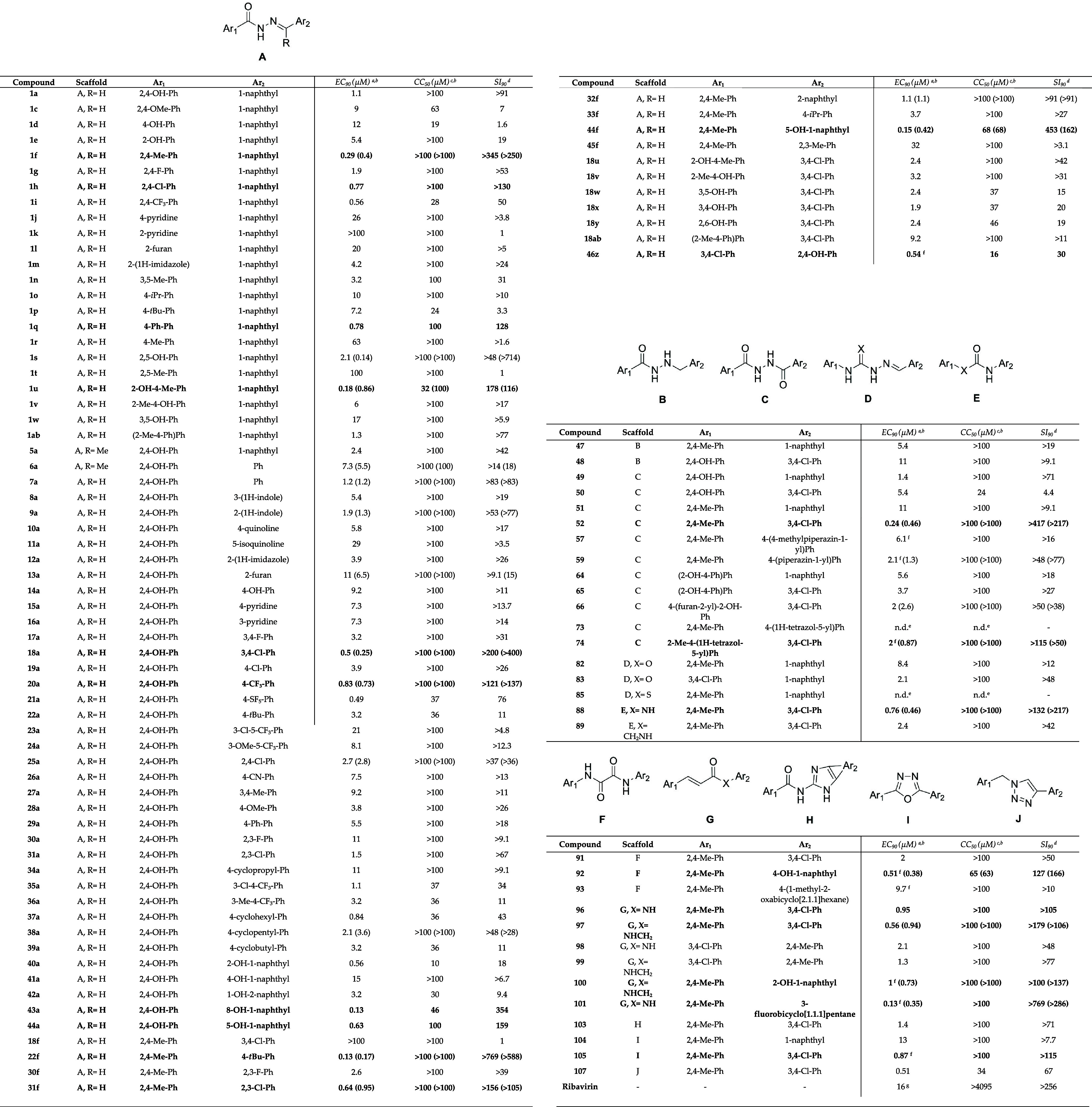
Antiviral and Cytotoxicity Data for the Compounds Synthesized in This Study

a EC_90_ = 90% effective concentration (concentration at which virus yield is reduced by one log_10_).

b Data are largely the results of 4-concentration assays. For selected compounds, a second 8-concentration test was performed to confirm initial results, and the additional testing results are shown in parentheses.

c CC_50_ = 50% cytotoxic concentration (concentration at which 50% adverse effect is observed on the host cell).

d SI_90_ = the ratio of the 50% cytotoxic concentration (CC_50_) to the 90% effective concentration (EC_90_).

e n.d. = not determined due to solubility issues.

f For some of the compounds, an 8-concentration VYR was performed for the initial tests.

g Ribavirin (included as the positive control in each series of compounds tested) data represent the average of 11 test results.

SAR analysis across this series identified clear substitution requirements on both aromatic regions and different non–hydrazone linkers that preserve, and in some cases enhance, sub–micromolar potency (Table 1, Figure 4a). Most analogues showed measurable activity, with many in the low–micromolar range and several achieving sub–micromolar EC_90_ values with SI_90_ > 100.

**Figure 4:**
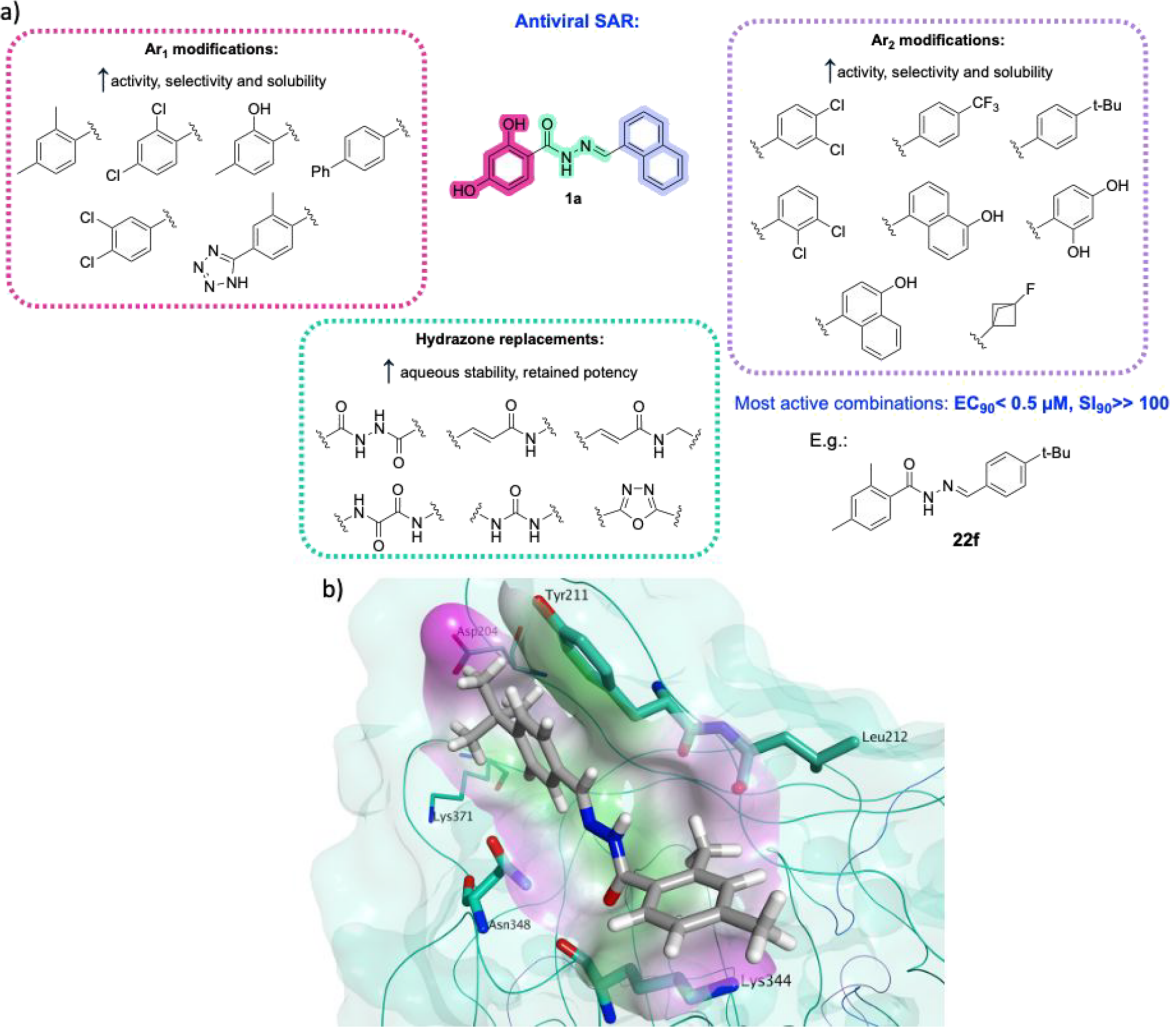
(a) Summary of the best point modifications made to antiviral hit **1a**. The best modifications made to Ar_1_ are shown in the pink dotted box, those to Ar_2_ in the lilac dotted box, and those to the linker region in the green dotted box. (b) Predicted binding of **22f** within the MACV GP1-hTfR1 interface (PDB 3KAS), generated using Glide SP. Compound **22f** shows a binding mode consistent with that of **1a**, retaining the potential for π–π stacking interaction with Tyr211 and for a cation−π interaction with the side chain of Lys344. The hydrazide group of **22f** is positioned within hydrogen–bonding distance of Asn348. The apical domain of hTfR1 is represented as a green ribbon and a green molecular surface, with the carbon atoms of interacting residues shown in green. The druggable subpocket area centered around Tyr211 and Asn348 is highlighted with a hydrophilic/lipophilic molecular surface (pink = hydrophilic, green = lipophilic, and white = neutral). Compound **22f** is represented with carbon atoms in gray.

For the hydrazone scaffold (A in Table 1), the most potent and selective analogues included **22f**, **44f** and **46z**, with additional active compounds **1f**, **1h**, **1q**, **1u**, **18a**, **20a** and **31f**. A consistent trend was an optimal substitution pattern on Ar_1_ centered on the 2,4 positions; importantly, non–hydrogen–bonding groups at these positions could retain or improve potency (e.g., **1f**, **1h**), indicating that steric/electronic effects dominate in this region. Ar_2_ tolerated bulky *para*–substituents (e.g., **22f**, **20a**) and selected aromatic variations, while substitution at other positions was frequently detrimental; notable cooperative effects were observed, where combining individually favorable Ar_1_ and Ar_2_ elements abolished activity (**18f**).

Linker replacements (B–J in Table 1) showed that increased flexibility was generally unfavorable: reduction of the hydrazone to hydrazides (**47**, **48**) reduced potency, consistent with a requirement for rigidity. Several non–hydrazone linkers retained strong activity, including benzoyl–hydrazide (notably **52**), oxalic amide (**92**; SI_90_ = 127), acrylamide (**96**, **97**, **101**), and cyclic replacements such as oxadiazole **105**. Collectively, these data establish multiple viable routes to improve stability and potential for further optimization while preserving antiviral potency (Figure 4a,b).

#### 2.3.2 Viral Entry Inhibition Studies

A subset of four selected compounds (**18a**, **20a**, **22f**, and **44f**) was evaluated at two fixed concentrations, 10 and 50 μM, for their ability to inhibit entry of a murine leukemia virus (MLV) pseudotyped with MACV glycoprotein, encoding an eGFP reporter.^25^ Following a 30 min pretreatment with compounds and a 16-h infection in the presence of compounds, HEK-293T/T17 cells were washed to remove unbound virus, refreshed with media, and incubated for an additional 32 h to allow eGFP expression in infected cells. Viral entry and its inhibition were quantified by flow cytometry detecting eGFP fluorescence (Figure 5a). Among the tested analogues, **22f** exhibited the most potent activity, reducing entry of pseudotyped MLV expressing the MACV GPC by 70% at 10 μM compared to vehicle-treated cells. The positive control ch128.1 IgG1 monoclonal antibody, which competes with pathogenic NWA GPC for the hTfR1 apical domain,^25^ reduced entry by 86% compared to vehicle control cells (Figure 5a). Toxicity was assessed indirectly by measuring flow cytometry acquisition times required to analyze 10,000 cells (Figure 5b). Prolonged acquisition time, indicating reduced cell viability, was only observed with **44f** at 50 μM, consistent with the 68 μM CC_50_ determined in the initial testing in A549 cells. The inhibitory effect of the selected compounds is consistent with targeting the interaction between arenavirus GP1 and the hTfR1 apical domain, and **22f** was identified as the most promising compound for further analysis. Its predicted binding within the hTfR1 apical region is shown in Figure 4b.

**Figure 5:**
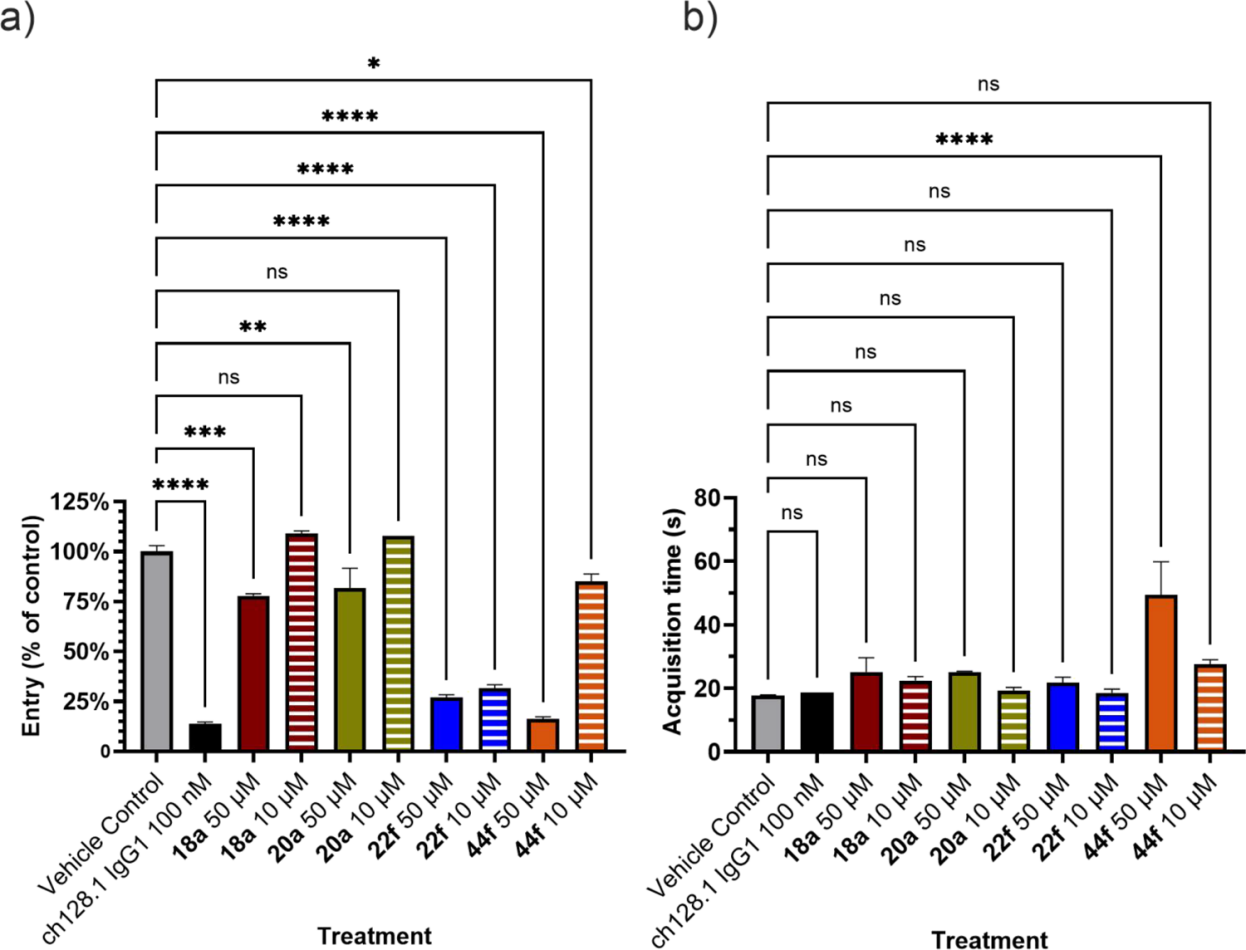
(a) Percent inhibition of entry into HEK 293T/T17 cells by MLV pseudotyped with MACV GPC after treatment with **18a**, **20a**, **22f** and **44f,** compared to cells treated with the vehicle control. (b) Compound toxicity measured indirectly by the length of time required to collect 10,000 events (cells) during flow cytometric analysis, gating FSC and SSC for healthy, intact cells. A delay in acquisition time caused by reduced cell division and dead or dying cells in the fixed-volume suspension indirectly measures compound toxicity. Data shown are the means ± SD from two separate experiments. ****p < 0.0001, ***p < 0.001, **p < 0.01, *p < 0.05.

To assess its breadth of inhibition, **22f** was evaluated for its ability to block viral entry across multiple MLVs pseudotyped with GPCs from several additional Clade B arenaviruses, including JUNV, GTOV, and Tacaribe virus (TCRV). As summarized in Figure 6a, **22f** demonstrated potent inhibition of all tested pseudotyped viruses, with the most dramatic effect observed against JUNV and TCRV pseudotypes. The tested arenavirus pseudotypes exhibit distinct cellular entry pathways: MACV, JUNV, and GTOV rely on hTfR1 for cellular entry, whereas TCRV, despite its close relation to JUNV and MACV, employs a hTfR1-independent mechanism to enter the host cell, as confirmed by the inability of the ch128.1 IgG1 to block entry by the TCRV pseudotype. Notably, previous studies have shown that blocking hTfR1 with specific antibodies unexpectedly enhances TCRV infection,^46^ as was observed with the ch128.1 IgG1 treatment. Because **22f** also inhibited TCRV entry, its antiviral effect is unlikely to be limited to hTfR1 and may reflect interference with a broader entry-associated process. During preparation for flow cytometry, trypsin-detached cell numbers and viability were similar for ch128.1 IgG1 and **22f** (Figure 6b), although both treatments produced slight-to-modest RLU reductions relative to vehicle, consistent with subtle effects on cell growth and/or trypsin sensitivity. Overall, activity across arenaviruses with distinct entry routes supports **22f** as a broadly acting entry-stage inhibitor.^47,48^ Intrigued by **22f**’s inhibitory effect on pseudotyped TCRV, we performed several follow-up experiments evaluating **22f** at a broader range of concentrations against MLV pseudotyped with the LASV GPC. LASV is an Old World (OW) arenavirus with a GP1 subunit that primarily uses host α-dystroglycan rather than TfR1 for cellular entry, so it was surprising to see that **22f** significantly reduced pseudotyped LASV entry at 2, 10, and 50 μM concentration (Supporting Information, Figure S4). The pseudotyped TCRV and LASV entry inhibition data suggest that **22f** also acts by a mechanism distinct from the TfR1 target.

**Figure 6:**
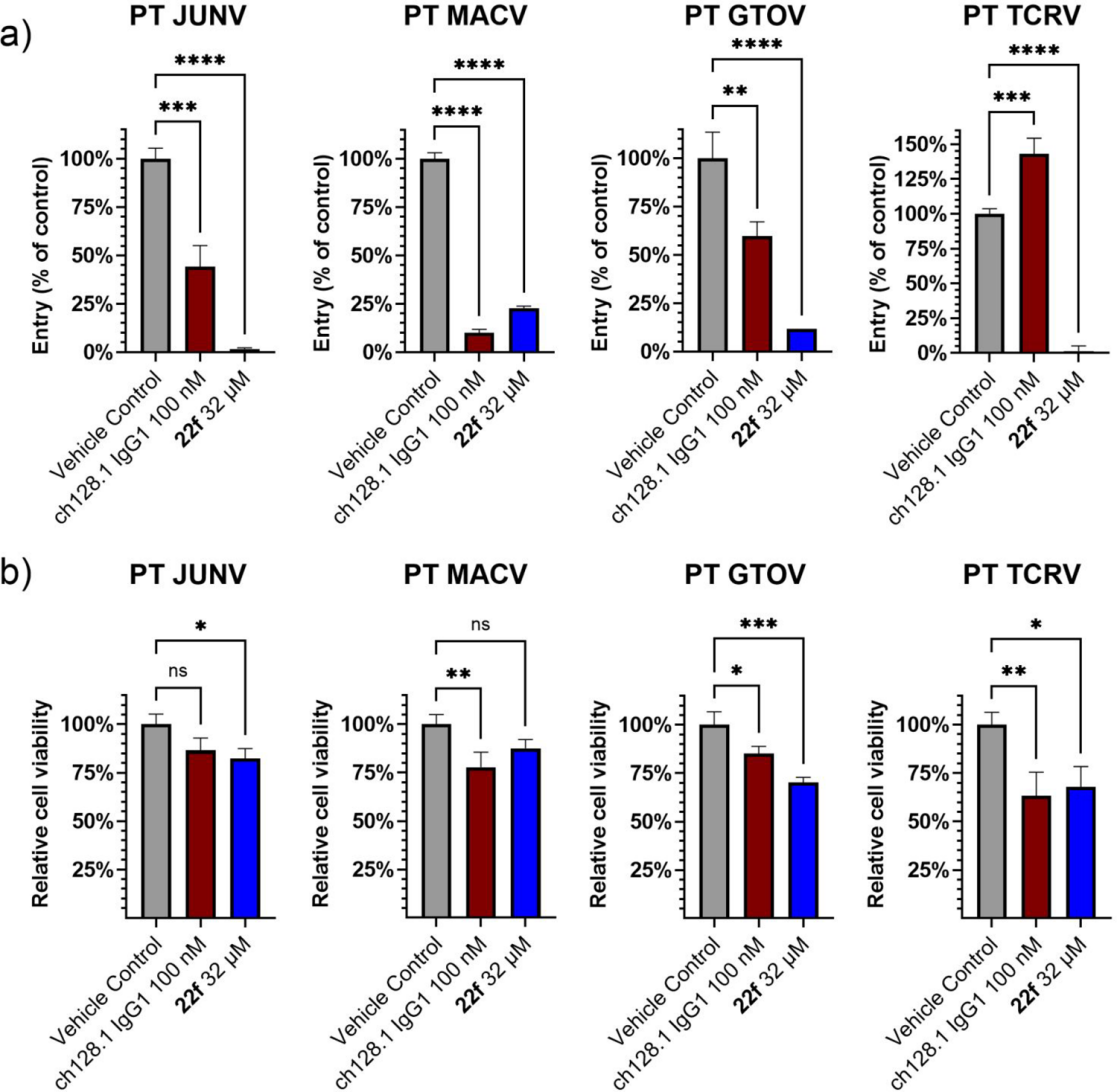
(a) Percent inhibition of entry into HEK 293T/T17 and (b) relative cell viability with **22f** treatment against pseudotyped (PT) MLVs expressing NWA GPCs for JUNV, MACV, GTOV, or TCRV. Viral entry results represent the percent reduction in entry by **22f** compared to the vehicle control. Cytotoxicity was determined in parallel by measuring the viability of treated cells using the CellTiter-Glo Luminescent Cell Viability Assay, with luminescence units normalized to the average vehicle control luminescence for each experiment. The **22f** compound was well tolerated by cells, exhibiting a toxicity profile comparable to that of the ch128.1 positive-control antibody treatment. Data shown are the means ± SD from two separate experiments for each pseudotyped virus. ****p < 0.0001, ***p < 0.001, **p < 0.01, *p < 0.05.

#### 2.3.3 Further Antiviral Assays

To further evaluate its broad-spectrum potential, compound **22f** was assessed by VYR assay in A549 cells, and a cytopathic effect (CPE) reduction assay in Vero cell lines, against native TCRV, Pichindé virus (PICV, a more distantly related Clade A NWA),^49^ and Rift Valley fever virus (RVFV, a phylogenetically unrelated phlebovirus). The results, summarized in Table 2, indicate that **22f** exhibits selective activity toward Clade B NWAs, with weak to no activity against PICV and no detectable inhibition of RVFV replication. While weak activity was observed with PICV by the CPE assay, it was not confirmed by VYR. PICV does not utilize TfR1 for cell entry.^50^ Consistent with previous pseudotyped virus assay results (Figure 6a), **22f** demonstrated low micromolar-range activity against TCRV (Table 2). The potency of **22f** against TCRV was markedly lower than that observed for JUNV (0.13 and 0.17 μM; Table 1), indicating that antiviral performance varies across arenaviruses with different entry routes and is compatible with differing pathway dependence. We confirmed this potency difference in a side-by-side comparison experiment (Supporting Information, Table S2). From an antiviral discovery perspective, the ability of **22f** to inhibit arenaviruses with distinct entry mechanisms supports its further development as a broader-spectrum entry-stage inhibitor.

**Table 2:** Antiviral and Cytotoxicity Data for Compound **22f**

		**TCRV** ^a^			**PICV** ^b^			**RVFV** ^c^		
compound	assay	EC_50/90_(μM)^d^	CC_50_(μM)^e^	SI_50/90_ ^f^	EC_50/90_(μM)^d^	CC_50_(μM)^e^	SI_50/90_ ^f^	EC_50/90_(μM)^d^	CC_50_(μM)^e^	SI_50/90_ ^f^
**22f**	VYR	1.7	>100	>59	100	>100	>1	>100	>100	1
	CPE	3.8	53	14	12	61	5.1	>100	>100	1
ribavirin	VYR	15	>4095	>273	38	>4095	>108	25	>4095	>164
	CPE	36	4095	114	66	4095	63	45	>4095	>91

a Antiviral effect on the replication of TCRV in A549 cells (3-day VYR) and Vero cells (7-day CPE reduction assay).

b Antiviral effect on the replication of PICV in A549 cells (3-day VYR) and Vero cells (7-day CPE reduction assay).

c Antiviral effect on the replication of RVFV in A549 cells (3-day VYR) and Vero 76 cells (5-day CPE reduction assay).

d EC_50/90_ = 50 or 90% effective concentration (concentration at which 50 or 90% antiviral effect is observed by CPE or VYR assay, respectively).

e CC_50_ = 50% cytotoxic concentration (concentration at which 50% adverse effect is observed on the host cell).

f SI_50/90_ = the ratio of the 50% cytotoxic concentration (CC_50_) to the EC_50_ or EC_90_, respectively.

To further define the stage at which **22f** exerts antiviral activity, time–of–addition studies were performed comparing the effects of ch128.1 IgG1 and **22f** at pre– and post–infection (p.i.) time points in A549 cells infected with JUNV (Candid#1). As shown in Figure 7, **22f** was most effective when added 1 h prior to infection, consistent with inhibition at an early step of the entry process. However, **22f** also reduced virus yields when added two or 4 h p.i., indicating that its activity is not limited to the initial attachment phase, and may extend to one or more post–attachment, entry–associated steps. As expected for an hTfR1–blocking antibody, ch128.1 IgG1 was only effective when added before infection. Interestingly, when added p.i., ch128.1 IgG1 increased JUNV progeny, a phenomenon reported previously for TCRV when cells were treated with an hTfR1–targeting antibody.^46^ Overall, our data indicate that **22f** inhibits early stages of JUNV infection and that its antiviral profile is consistent with effects at multiple points along the entry pathway.

**Figure 7:**
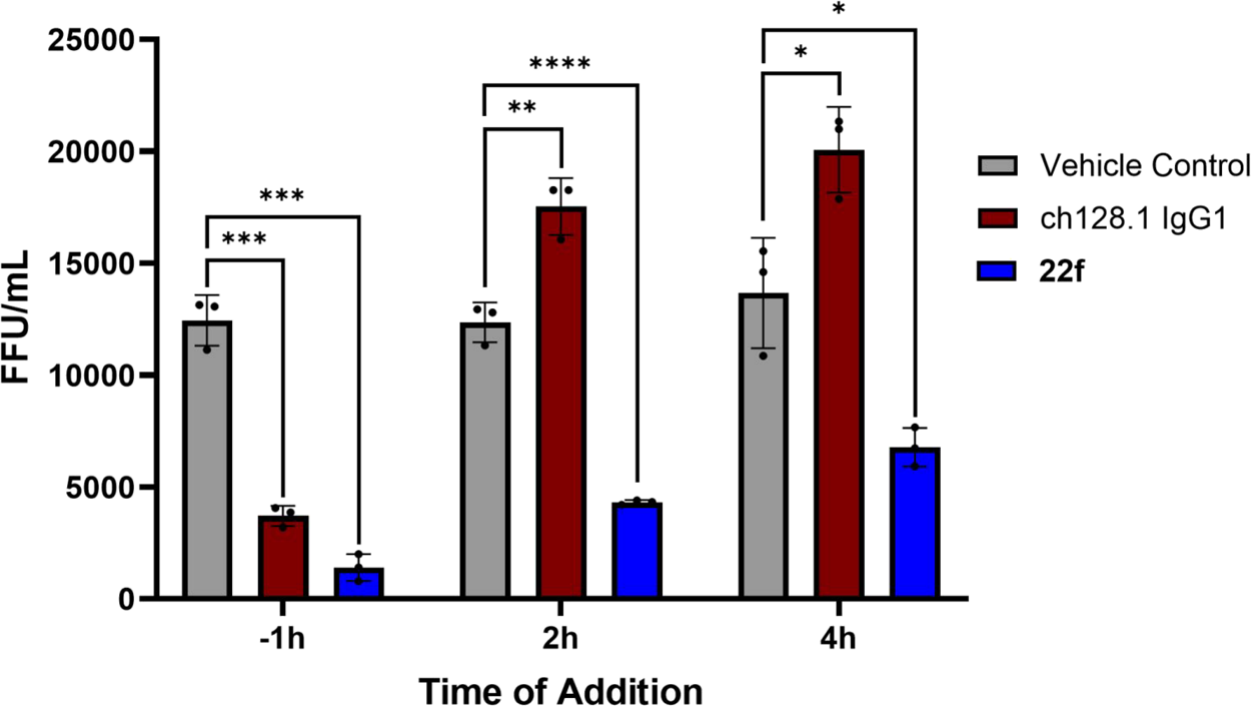
Impact of the time of addition of **22f** on Candid#1 JUNV infection in A549 cells. Cells were treated with a final concentration of 200 nM ch128.1 IgG1 or 50 μM **22f** at the indicated times relative to JUNV infection at a MOI of 0.05. Infected cells exposed to MEM lacking treatment (vehicle control) were included for comparison. Supernatants were collected at 28 h p.i., and virus yields were determined via focus-forming unit (FFU) assay in Vero cells. Depicted are means of biological triplicates ± SD from one experiment, which was representative of two independent experiments. ****p < 0.0001, ***p < 0.001, **p < 0.01, *p < 0.05.

#### 2.3.4 Microsomal Stability and In Vivo Efficacy Studies

To support selection of a candidate for *in vivo* antiviral efficacy evaluation, the metabolic stability of a small subset of active compounds representing key structural modifications (**22f**, **91** and **97**) was assessed *in vitro* using rat liver microsomes. Metabolic half-lives (t_1_/_2_) were determined under standard NADPH-supplemented incubation conditions, with remaining parent compound quantified by LC–MS at selected time points.^51^ This assay provides an estimate of intrinsic clearance and susceptibility to first-pass metabolism, informing the likelihood of achieving adequate systemic exposure. Chromatographic separation was sufficient to quantify parent compound without interference from metabolites. The calculated half-lives are summarized in Table S3. Verapamil was included as a positive control and showed a t_1_/_2_ of 5.48 min, consistent with literature values (typically <10 min),^52^ confirming assay performance.

Among the compounds tested, **22f** showed the highest microsomal stability (t_1_/_2_ > 35 min), whereas **91** and **97** were more rapidly metabolized. The enhanced stability of **22f** may reflect reduced metabolic liability associated with the *tert*-butyl substituent in Ar_2_. Together with its antiviral potency and selectivity profile, these data supported progression of **22f** to animal model studies.

We then explored the antiviral effects of **22f** in the hTfR1 mouse model of infection with the pathogenic JUNV Romero strain. **22f** was prepared in CES (10% cremophor, 10% ethanol, 80% saline) and administered by intraperitoneal (IP) injection for 10 days to 20- to 23-day-old hTfR1 mice challenged with JUNV. At the tested dosing regimen, **22f** was well tolerated in uninfected mice, with only a slight weight decrease and no signs of toxicity or behavioral changes observed throughout the treatment period at the highest dose of 50 mg/kg/day (Supporting Information, Figure S5). As shown in Figure 8a, mice challenged with JUNV and treated with **22f** at 50, 15.8, or 5 mg/kg/day had survival rates similar to or lower than those of mice treated with the CES vehicle placebo (50% survival). Mice treated with the positive control drug favipiravir had a 62.5% survival rate, and the animals that succumbed did so significantly later than those treated with the CES placebo (mean day of death: 19.3 ± 0.6 favipiravir, 14.0 ± 2.2 placebo, *p* = 0.012). Favipiravir is generally given twice daily for optimal effect; however, we chose to treat the animals once daily for consistency with the regimen used for the **22f** and placebo treatments. Mortality in the placebo group was less than expected.^53,54^

**Figure 8:**
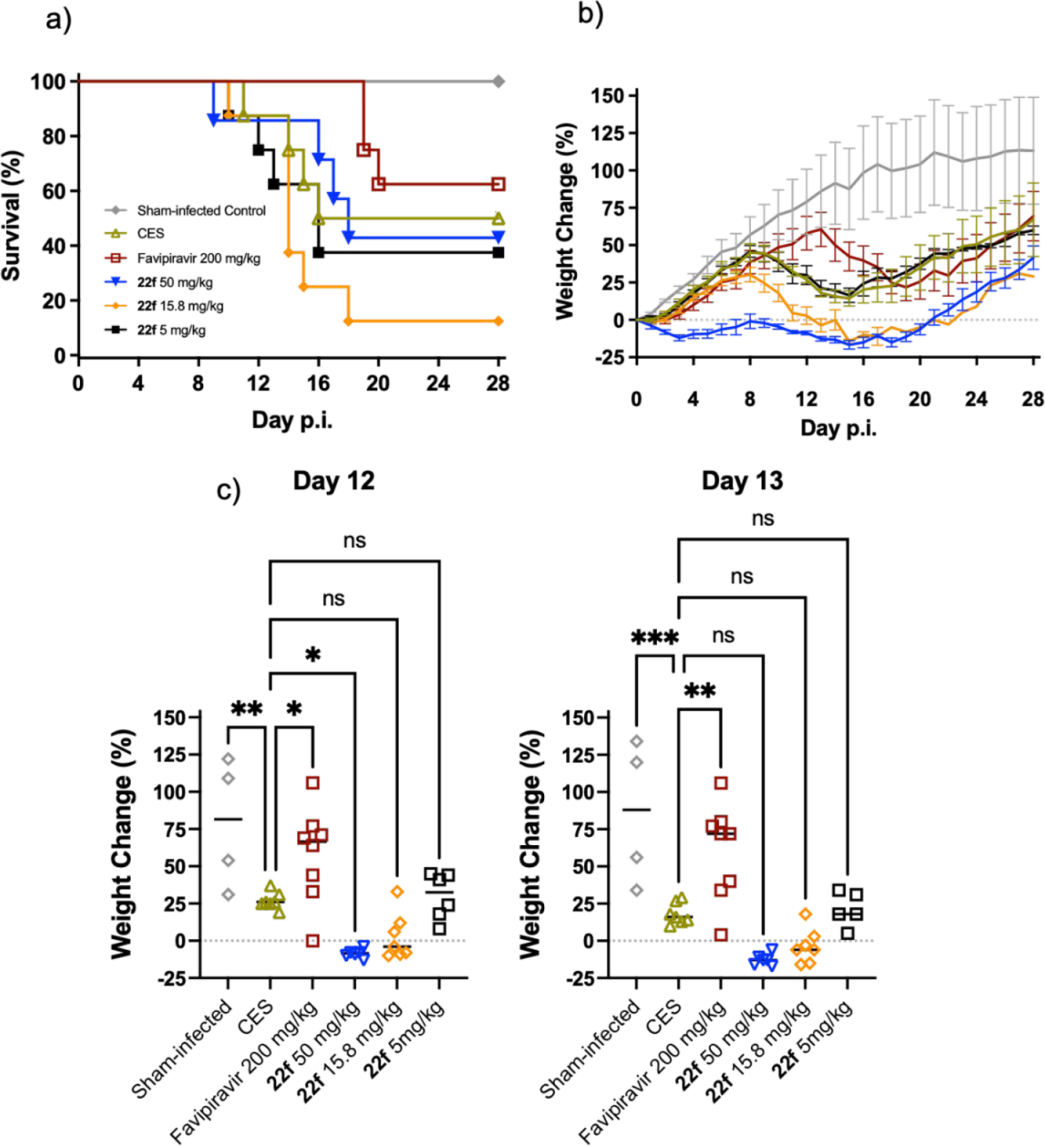
Effect of **22f** treatment on the survival and weight change of hTfR1 mice challenged with JUNV. Animals (*n* = 7 or 8/group) were challenged with 10^4^ CCID_50_ (median cell culture infectious dose) of JUNV Romero and treated once daily with the indicated dosages of **22f** or favipiravir. Sham-infected normal controls (*n* = 4/group) were included for comparison. (a) Survival outcome and (b) longitudinal body weight represented as the group mean and standard error (SEM) of percent change in weight of animals relative to their starting weights on day 0, the day of virus challenge. (c) Weights of individual animals and the group means (lines) are shown for days 12 and 13 p.i. ***p < 0.001, **p < 0.01, *p < 0.5, compared to CES placebo control animals.

Animal weights were measured daily and are reported as the mean percent change in weight for each treatment group relative to day 0 (Figure 8b). Dramatic weight loss was observed early and throughout the study in mice treated with the 50 mg/kg dose of **22f**, with weight gain and recovery beginning toward the end of the third week, after the sick animals had succumbed. Weight loss was also more pronounced with the 15.8 mg/kg dose of **22f** than with the lower 5 mg/kg dose or the vehicle placebo. While differences in overall survival rates between treatment groups were not statistically significant, on days 12 and 13 p.i. (when there were still adequate numbers of mice available to power statistical analysis), only the mice treated with favipiravir had significantly greater weight gain compared to the placebo group (Figure 8c). The weight data suggest that, in the face of JUNV infection, higher doses of **22f** were associated with some degree of toxicity, which was limited in the maximum tolerated dose study in the absence of viral infection (Supporting Information, Figure S5). Overall, no significant improvement in survival or weight gain was detected in **22f**-treated mice compared to placebo-treated controls. While these findings indicate a lack of *in vivo* efficacy under the present experimental conditions, the strong *in vitro* antiviral potency and dual mechanism of action of the scaffold support its continued development. In particular, optimization of PK properties, through further modifications aimed at improving systemic exposure and tissue distribution, may be required to fully translate the promising *in vitro* activity into an *in vivo* effect.

## 3 Conclusions

In this study, we applied a structure–based screening and optimization strategy to identify small–molecule inhibitors with antiviral activity against pathogenic New World arenaviruses (NWAs). From initial virtual screening hits, systematic analogue design and SAR exploration yielded multiple sub–micromolar inhibitors of JUNV, with our current lead compound **22f** also blocking MACV, GTOV, and even OW arenavirus LASV GCP-based entry in cell–based systems, while showing no activity against unrelated RVFV. Although **22f** showed potent antiviral activity across multiple arenaviruses *in vitro*, it did not confer protection in an hTfR1–expressing mouse model of JUNV infection. This outcome likely reflects a combination of pharmacokinetic and pharmacodynamic limitations: despite good microsomal stability, the *in vivo* exposures achieved under the tested dosing regimen were likely insufficient to maintain antiviral concentrations at relevant tissue sites, and the *in vitro* data do not yet establish the precise target engagement or mechanism required for *in vivo* efficacy. Thus, this lack of protection is more consistent with an exposure-response mismatch than with intrinsic potency. Future optimization will therefore require coordinated improvements in both PK and PD properties to translate the favorable *in vitro* profile of this scaffold into *in vivo* antiviral activity. Importantly, while these compounds were designed to target the GP1-hTfR1 interaction interface, the current data do not yet establish direct engagement of hTfR1. Defining the relevant molecular target(s) will be a priority of further work. Collectively, this work delivers a promising expanded-spectrum antiviral chemotype, and a foundation for next–generation arenavirus entry inhibitors.

## 4 Materials and Methods

### 4.1 Molecular Modeling Studies

All molecular modeling experiments were performed on Asus WS X299 PRO Intel i9–10980XE CPU @ 3.00 GHz x 36, running Ubuntu 18.04 (GPU: GeForce RTX 2080 Ti). Molecular Operating Environment (MOE, 2024.0601, Montreal, QC, Canada),^33^ Maestro (Schrödinger Release 2025–2, Schrödinger, LLC, New York, NY, 2025),^35^ PLANTS,^38^ and Seesar (version 14.2.0, Sankt Augustin, Germany)^39^ were used as molecular modeling software.

A library of commercially available compounds was downloaded from the SPECS Web site^34^ in sdf format and prepared using the Maestro LigPrep tool. Structures were energy-minimized using the OPLS_2005 force field, and possible ionization states at pH 7.2 (Epik), tautomers, and low-energy ring conformers were generated. Compounds with chiral centers were considered as racemic mixtures, and up to three stereoisomers per chiral compound were included in the virtual screening. Only the best-performing stereoisomer per molecule was retained for visual inspection.

The crystal structure of hTfR1 in complex with MACV GP1 (PDB code 3KAS) was downloaded from the RCSB Protein Data Bank (http://www.rcsb.org/). The protein was prepared using MOE Protein Preparation tools, retaining GP1-interacting residues Asp114–Val117 as ligand. The resulting protein–ligand complex was saved in *.pdb (for FlexX rescoring), *.mol2 (for PLANTS rescoring after GP1 removal), and *.mae (for Maestro) formats. The *.mae protein structure was further processed with the Schrödinger Protein Preparation Wizard, assigning bond orders, adding hydrogens, and performing restrained minimization of the added hydrogens using the OPLS_2005 force field. A docking grid (inner box 10 Å, outer box 22 Å) was centered on the GP1 ligand.

An initial high-throughput virtual screening (HTVS) of the SPECS library was performed using Glide HTVS precision with default parameters, generating one output pose per ligand. The top 10% of compounds were subsequently redocked using Glide SP precision, generating three poses per ligand. Docking results were rescored using Glide XP, FlexX Score, and CHEMPLP (PLANTS) scoring functions. Values from the three scoring functions were combined to generate a consensus score, and only poses in the top 25% of the score range for all three functions were selected for visual inspection in MOE.

Visual inspection considered the following criteria for final selection of 25 virtual hits:

- Adequate occupation of the target binding site;
- Number and quality of nonbonded interactions (H-bonds, p-p interactions, etc.);
- Chemical diversity, discarding highly similar scaffolds.

### 4.2 Synthetic Chemistry

All solvents and reagents were used as obtained from commercial sources unless otherwise indicated. All solvents used for chromatography were HPLC grade from Fisher Scientific (UK). All reactions were performed under a nitrogen atmosphere. ^1^H, ^13^C, ^19^F and ^31^P NMR spectra were recorded with a Bruker Avance III HD spectrometer operating at 500 MHz for ^1^H and 125 MHz for ^13^C, with Me_4_Si as internal standard. Deuterated chloroform or dimethyl sulfoxide were used as the solvents for NMR experiments, unless otherwise stated. ^1^H chemical shift values (δ) are referenced to the residual nondeuterated components of the NMR solvents (δ = 7.26 ppm for CHCl_3_, etc.). The ^13^C chemical shifts (δ) are referenced to CDCl_3_ (central peak, δ = 77.0 ppm). TLC was performed on silica gel 60 F254 plastic sheets. Flash column chromatography was performed using a Biotage Isolera One automated system. UPLC-MS analysis was conducted to measure the purity of all final compounds, on a Waters UPLC system with both Diode Array detection and Electrospray (+′ve and – ′ve ion) MS detection. The stationary phase was a Waters Acquity UPLC BEH C18 1.7 μm 2.1 50 mm column. The mobile phase was LC-MS grade H_2_O containing 0.1% formic acid (A) and LC-MS grade MeCN containing 0.1% formic acid (B). Column temperature: 40 °C. Sample diluent: MeCN. Sample concentration: 1 μg/mL. Injection volume: 2 μL. A linear gradient standard method was used, unless stated otherwise: 90% A (0.1 min), 90–0% A (1.5 min), 0% A (1.4 min), 90% A (0.1 min); flow rate 0.5 mL/min. High resolution mass spectra (HRMS) were measured in positive mode electrospray ionization (ES+). All compounds tested in biological assays were >95% pure. Purity of intermediates that were not biologically evaluated was >90%, unless otherwise stated. Preparation and characterization of intermediates and all final target products are fully described in the Supporting Information.

### 4.3 Biological and Antiviral Efficacy Studies

#### 4.3.1 Viruses for In Vitro and In Vivo Efficacy Studies

The Candid#1 vaccine strain of JUNV (1 passage in BSC-1, 2 passages in Vero) was kindly provided by Dr. Robert Tesh (World Reference Center for Emerging Viruses and Arboviruses, University of Texas Medical Branch). The molecular clone of the Romero strain of JUNV was generously provided by Dr. Slobodan Paessler (University of Texas Medical Branch).

To test hTfR1-docked entry inhibition, several pseudotyped murine leukemia viruses (MLV)­s were generated using three plasmids: (i) a plasmid encoding the murine leukemia virus *gag* and *pol* genes; (ii) a pCAGGS expression vector encoding arenaviral GPCs (JUNV, GTOV, MACV, TCRV, or LASV); and (iii) the pQCXIX retroviral vector (BD Biosciences) coding for enhanced green fluorescent protein (eGFP). These plasmids were generously provided by Dr. Jonathan Abraham (Harvard University). The production of the recombinant MLVs pseudotyped with arenaviral GPCs has been described previously. ^23^

#### 4.3.2 *In* *Vitro* Antiviral and Cytotoxicity Assays

*In vitro* antiviral activity of virtual anti-NWA hits was evaluated against the Candid#1 vaccine strain of JUNV in a cell culture-based virus yield reduction (VYR) assay.^55^ Compounds, dissolved in dimethyl sulfoxide (DMSO), were serially diluted in Dulbecco’s Modified Eagle Medium (DMEM; Thermo Fisher Scientific), maintaining the DMSO concentration ≤ 0.1% to minimize cytotoxicity. A549 human epithelial lung cells (ATCC CCL-185) were treated with a 100 μL volumes of 4 log_10_ (100, 10, 1, 0.1 μM) or 8 half-log_10_ (100, 32, 10, 3.2, 1, 0.32, 0.1, 0.032 μM) dilutions of each analog, for 30 min in 96-well microplates before the addition of Candid#1 (multiplicity of infection/MOI = 0.01) in cell culture medium. Plates were incubated for 3 days and virus yields were determined by end point dilution of the culture supernatants on Vero African green monkey kidney cells (ATCC CCL-81). Viral cytopathic effect (CPE) was determined 7 days after plating and virus titers (median cell culture infectious dose; CCID_50_) were calculated by the method of Reed and Muench.^56^ As the primary measure of potency, the EC_90_ (the concentration of the compound that reduces the virus yield by 1 log_10_) was calculated by regression analysis. The 50% cell cytotoxic dose (CC_50_) was determined by neutral red dye uptake in uninfected cells treated with the compounds and cultured in parallel. The selectivity index (SI) for each compound was calculated using the formula: SI = CC_50_/EC_90_.

For the pseudotyped MACV entry inhibition assay, HEK293T/T17 cells maintained in high-glucose DMEM supplemented with 10% FBS were plated in 48-well plates in the same medium supplemented with 50 μg/mL gentamicin (DMEM-complete) 24 h prior to infection. Compounds **18a**, **20a**, **22f** and **44f** were dissolved in DMSO and added to DMEM-complete prior to infection with pseudotyped MACV. As a positive control for hTfR1-based entry inhibition, the ch128.1 IgG1 (kindly provided by Dr. Manuel Penichet, University of California, Los Angeles) antibody targeting the apical domain of hTfR1 was diluted to the desired concentration in DMEM-complete. For the vehicle control, an equivalent amount of DMSO was added to DMEM-complete. The controls and tested compounds were combined with the pseudotyped MACV and added to ∼60% confluent HEK293T/T17 cells. The cell cultures were incubated for 16 h at 37 °C and 5% CO_2_, then washed twice before overlaying with fresh DMEM-complete for an additional 32-h incubation. The cells were then trypsinized and washed with flow buffer (PBS, 0.5% bovine serum albumin (BSA), and 2 mM EDTA) before fixation with 1% paraformaldehyde (Electron Microscopy Sciences). The fixed samples were analyzed on a BD Accuri C6 Plus Flow Cytometer. Cells were gated based on FSC and SSC, collecting 10,000 events per sample, and green fluorescence (525 nm) emission from the cells was recorded. Cells that exceeded the background green fluorescence signal present in the uninfected control cells were considered infected. The time required to collect 10,000 events (based on FSC and SSC) was used to measure relative toxicity across treatments.

The pseudotyped JUNV, MACV, GTOV, and TCRV **22f** viral entry inhibition assays were performed using the same protocol as described above, with two variations. First, each compound treatment was tested in biological triplicate in each repeated experiment. Second, toxicity was assessed using the CellTiter-Glo Luminescent Cell Viability Assay (Promega). Following trypsinization and washing with flow buffer, an aliquot of each sample was taken for toxicity assessment, as per the manufacturer’s specifications. Briefly, sample aliquots were diluted 1:10 and added to an equal volume of CellTiter-Glo solution in white, opaque 96-well plate wells. The plates were gently agitated for 2 min to lyse cells, then incubated at room temperature for 10 min. The luminescence emitted from each well was measured using a BioTek Synergy LX multimode microplate reader (Agilent), and relative luminescence units were normalized to the average luminescence of vehicle control wells for each experiment. Follow-up pseudotyped LASV **22f** viral entry inhibition assays and parallel cell viability assays were performed similarly, with the addition of LHF-535 (TargetMol) as a positive control entry inhibitor for OW arenavirus entry, which was prepared in the same vehicle control solution.

Time-of-addition viral yield assays were performed by treating confluent A549 human epithelial lung cells with a final concentration of either 50 μM **22f**, 200 nM ch128.1 IgG1, or the vehicle control Minimum Essential Media (MEM; Thermo Fisher Scientific), all containing 0.25% DMSO. Biological triplicates were treated at 1 h preinfection, 2 h p.i., or 4 h p.i. Cells were infected with JUNV Candid#1 (MOI = 0.05). At 10 h p.i., cells were washed and overlaid with MEM supplemented with 2% FBS and 50 μg/mL gentamicin. At 28 h p.i., culture supernatants were collected and stored at −80 °C until virus yield determination by focus-forming unit (FFU) assay.

To measure FFUs, serial dilutions of the thawed culture supernatants were added to duplicate wells of confluent Vero cells. After a 2 incubation, the cells were overlaid with MEM containing 0.68% microcrystalline cellulose (Sigma-Aldrich), 2% FBS, and 50 μg/mL gentamicin, and incubated for 48 h. After the incubation, the overlay was removed, the cells were washed with PBS, and then fixed in an equal volume solution of methanol and acetone for 20 min at −20 °C. The air-dried plates were rehydrated with a PBS/0.1% TritonX-100 solution and subsequently blocked with PBS/0.1% TritonX-100/1% BSA/5% skim milk before immunohistochemical staining with primary monoclonal antibody AG12 targeting the JUNV nucleoprotein (BEI Resources) and a secondary horseradish peroxidase-conjugated antibody (Thermo Fisher Scientific). The cells were then developed with the ImmPACT NovaRED peroxidase substrate kit (Vector Laboratories), and the foci were quantified to determine FFU/mL.

#### 4.3.3 *In* *Vitro* Rat Liver Microsomal Stability Assay

The metabolic stability of compounds **22f**, **91**, and **97** was evaluated using pooled male Sprague–Dawley rat liver microsomes (0.5 mg/mL protein; Corning, catalog #452501) and an NADPH-regenerating system (Solutions A and B; Corning, catalogs #451220 and #451200), following a protocol adapted from Waters.^57^ Verapamil hydrochloride (Sigma-Aldrich, catalog #V4629) served as a positive control. Incubations were performed at 37 °C in 100 mM potassium phosphate buffer (pH 7.4; Sigma-Aldrich, catalog #P5244) containing 5 μM test compound (final DMSO ≤ 1% v/v) in a final volume of 250 μL. Reactions were initiated by adding microsomes to prewarmed mixtures of the test compound and the NADPH system, and aliquots were collected at 0, 5, 10, 20, and 30 min, then quenched with cold acetonitrile containing the internal standard. Zero-time controls were quenched prior to enzyme addition. Samples were centrifuged (3000 × g, 10 min, 4 °C) and supernatants analyzed by HPLC–MS (Waters ACQUITY BEH C18 column, 2.1 × 50 mm, 1.7 μm; 40 °C; 0.6 mL/min; gradient 5–95% B in 1.3 min, where A = 0.1% formic acid in water and B = 0.1% formic acid in acetonitrile). Detection was by ESI in positive mode on a Waters TQD. Calibration curves for each compound were generated from freshly prepared standards, with manual peak integration in Empower 3 (Version 3, Waters Corporation, Milford, MA, USA, 2022) and regression analysis performed in Microsoft Excel (Version 16.74, Microsoft Corporation, Redmond, WA, USA, 2023). First-order elimination rate constants (k) were derived from semilogarithmic plots of % parent remaining vs time, and half-lives (t_1_/_2_) were calculated as 0.693/k (*n* = 3).

#### 4.3.4 *In* *Vivo* Tolerability and Antiviral Efficacy Studies

All animal procedures complied with guidelines set by the USDA and the Utah State University Institutional Animal Care and Use Committee, and the mice enrolled in the studies were fed Harlan Lab Block and tap water *ad libitum.*

For the **22f** tolerability study, **t**he compound was suspended in CES (10% cremophor, 10% ethanol, 80% saline) at the final dosing concentrations. The hTfR1 knock-in (human *Tfrc* replacing the mouse *Tfrc*) mice on a hybrid C57BL/6 and 129 background were bred at Utah State University.^58^ Male and female 21- to 23-day-old hTfR1 mice were weighed the day of the first treatment and assigned to treatment groups so that sex and weight were evenly distributed across the groups (*n* = 4/group). Mice were administered 200, 100, 50, 25, or 5 mg/kg **22f** or the CES placebo by IP injection, once daily for 10 days. Due to the solubility of **22f**, the 200 mg/kg dose was administered in a total volume of 0.2 mL (placebo also in 0.2 mL). All other treatments were dosed in 0.1 mL. A group of untreated mice was included for comparison. The mice were weighed daily and observed for signs of toxicity for 18 days. Any animals that fell below 70% of their starting body weight were euthanized.

For the **22f** antiviral efficacy study, 21- to 23-day-old hTfR1 mice were weighed and assigned to treatment groups so that sex and weight were evenly distributed across infected groups (*n* = 7–8/group). Animals were challenged IP with 10^4^ CCID_50_ of JUNV Romero. Starting 2 h before infection, mice received 0.1 mL IP treatments of either 50, 15.8, or 5 mg/kg/day of **22f**, 200 mg/kg/day of favipiravir (purchased from TargetMol, Wellesley Hills, MA), or the CES vehicle placebo for 10 days. Uninfected male and female sham-infected control mice (*n* = 4) were included for comparison. Mice were observed for 28 days for morbidity and mortality, and their body weights were recorded daily. Animals with weight loss equal to or greater than 30% of their peak were considered moribund and humanely euthanized.

#### 4.3.5 Statistical Analyses

Pseudotyped virus entry inhibition and time-of-addition assay results were analyzed using one-way analysis of variance (ANOVA) with Dunnett’s posttest to correct for multiple comparisons. For the mouse antiviral efficacy study, the Kaplan–Meier survival plot was analyzed using the log-rank (Mantel-Cox) test. A one-way ANOVA with Dunnett’s posttest was used to compare mouse weights on days 12 and 13 p.i. The mean day of death of placebo- and favipiravir-treated mice was compared using the Welch’s *t*-test. All statistical evaluations were performed with Prism 10 (GraphPad Software).

## Supplementary Material



## Notes

Supplementary figures (Figures S1–S5); supplementary tables (Tables S1–S3); synthetic schemes (Schemes S1–S4); preparation and characterization of final target products and synthetic intermediates; representative ^1^H, ^19^F, and ^13^C NMR spectra (PDF)
