# Supplementary material for: Computer-Aided Discovery of Small-Molecule Inhibitors of Pathogenic New World Arenavirus Entry and Replication
Source: ACS Infect Dis. 2026 May 4;12(6):2046–59. doi: 10.1021/acsinfecdis.6c00138 (PMC13270517; doi:10.1021/acsinfecdis.6c00138)
Supplement: Supplementary file 1 [file id6c00138_si_001.pdf]

Supporting Information

Computer-aided discovery of small-molecule inhibitors of pathogenic New World arenavirus entry and replication

Samantha Rae Wasson [1], Ben Matthew Flude [2], Martina Salerno [2], Kie Hoon Jung [1], Gilda Padalino [3], Salvatore Ferla [3], Dylan Joseph Roche-Dugmore [2], Connor W Bott [2], Andrea Brancale [4], Brian B. Gowen [1]*, and Marcella Bassetto [2,5]*

1 Institute for Antiviral Research, Utah State University, Logan, UT 84322, USA
2 Department of Chemistry, College of Science and Engineering, Swansea University, Swansea SA2 8PP, UK
3 Medical School, Faculty of Medicine, Health and Life Science, Swansea University, Swansea, UK SA2 8PP, UK
4 Department of Organic Chemistry, University of Chemistry and Technology, Prague, Prague, 16628, Czech Republic
5 School of Pharmacy and Pharmaceutical Sciences, Cardiff University, Cardiff CF10 3NB, UK

* Correspondence: M.B., bassettom1@cardiff.ac.uk; B.B.G., brian.gowen@usu.edu

Contents

Figures S1-S5 *Pages S2-S5*

Tables S1-S3 *Pages S6-S8*

Schemes S1-S4 *Pages S9-S12*

Preparation and characterisation of final target products *Page S13*

Preparation and characterisation of synthetic intermediates *Page S28*

Representative ^{1}H, ^{19}F, and ^{13}C-NMR spectra for intermediates and final products *Page S35*

References *Page S72*

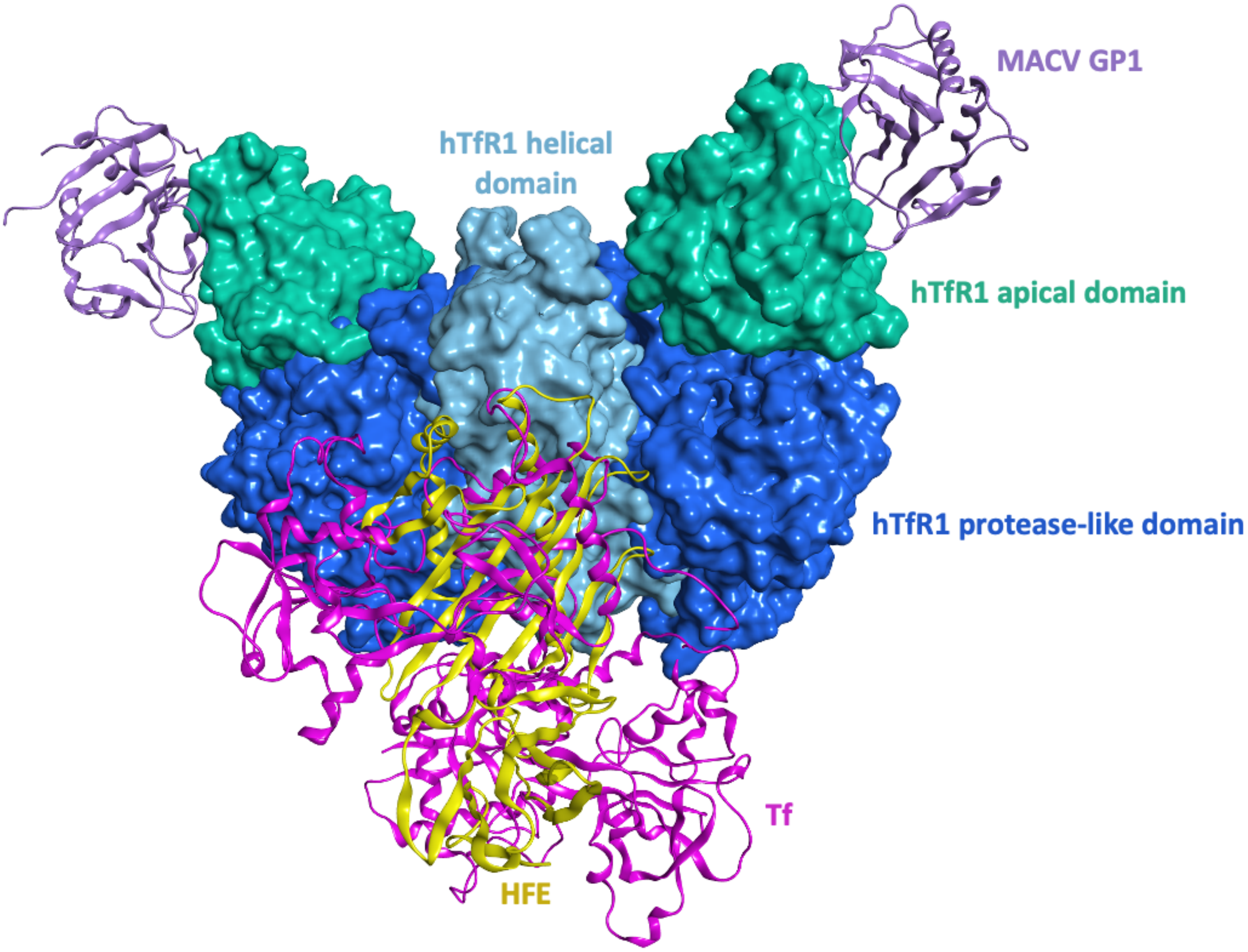

Figure S1. Crystal structure of the hTfR1 dimer in complex with MACV GP1 (PDB ID 3KAS). The hTfR1 extracellular domains are represented as molecular surface (apical domains in green, protease-like domains in blue, helical domains in light blue), while MACV GP1 is represented as a lilac ribbon. The structures of hTfR1 endogenous ligands, Tf (pink ribbon) and HFE (orange ribbon), superposed from the crystal structures of their respective complexes with hTfR1 (PDB ID 1SUV for Tf, and 1DE4 for HFE), highlight how the GP1 binding area in the apical hTfR1 domain is distinct from that of both physiological binders.

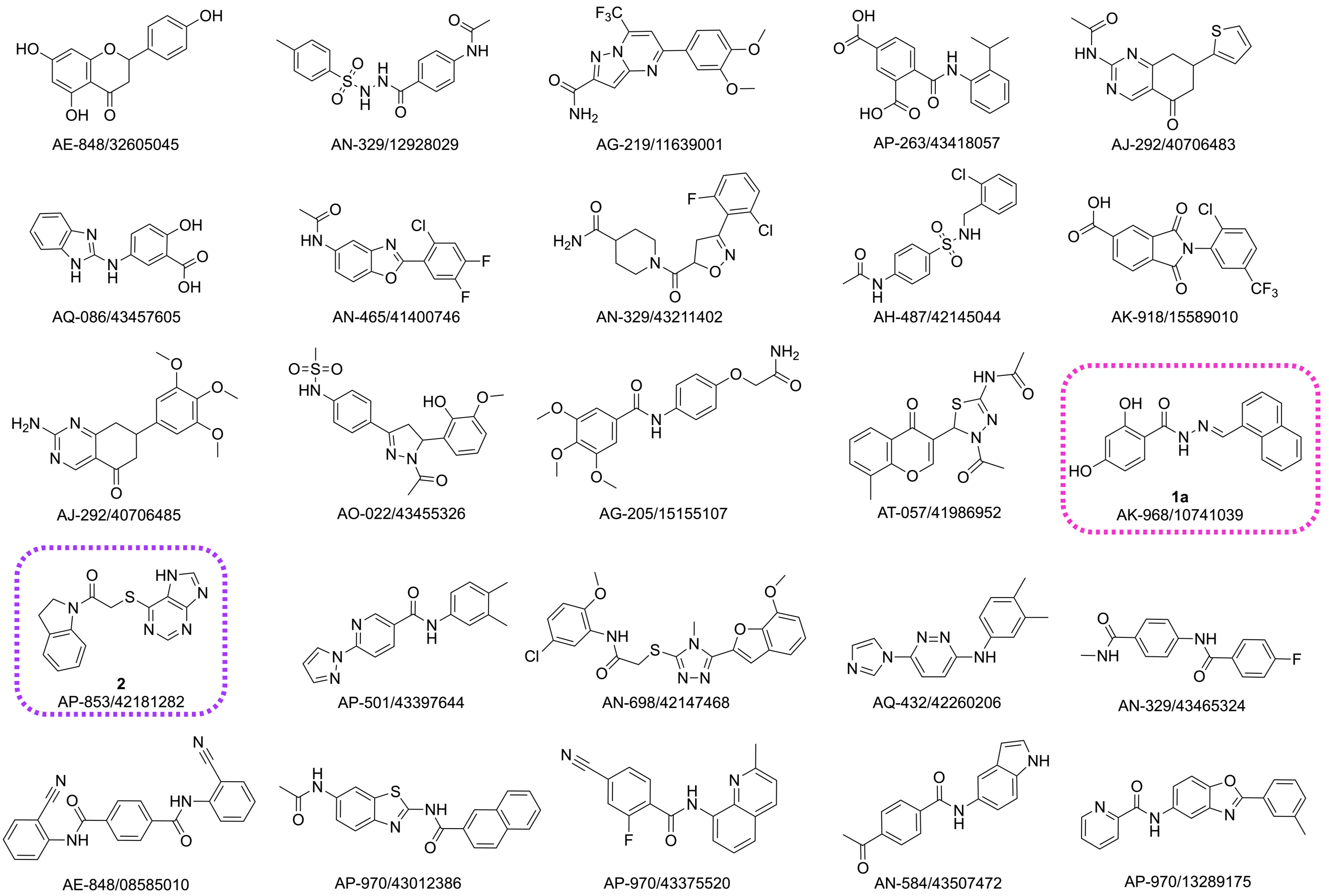

Figure S2. Chemical structure and manufacturer ID code for the virtual hits selected following the structure-based virtual screening on hTfR1 apical domain, and purchased from SPECS.

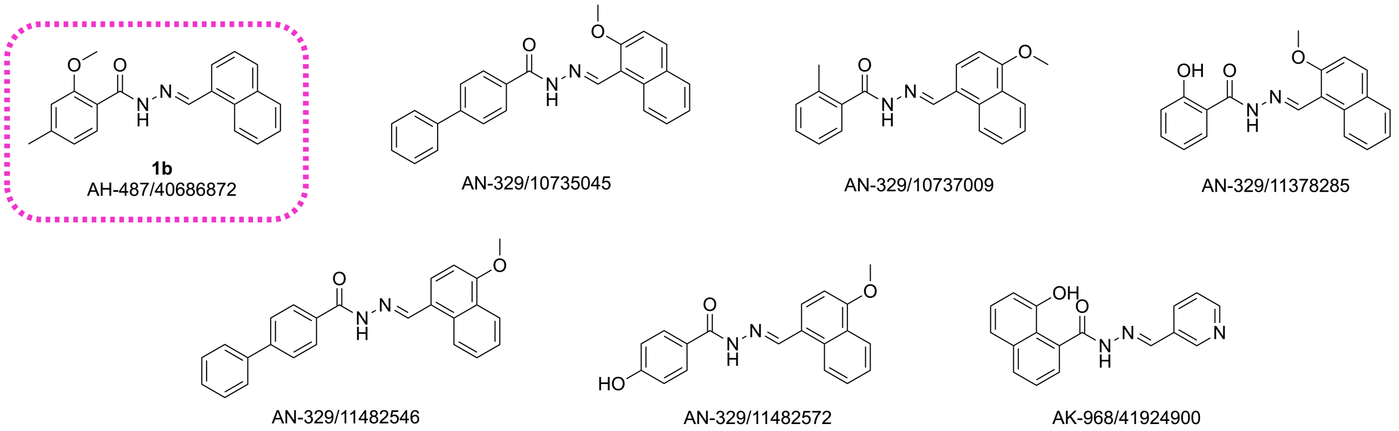

Figure S3. Chemical structure and manufacturer ID code for the analogues of antiviral hit **1** selected to confirm the activity of the scaffold, and purchased from SPECS.

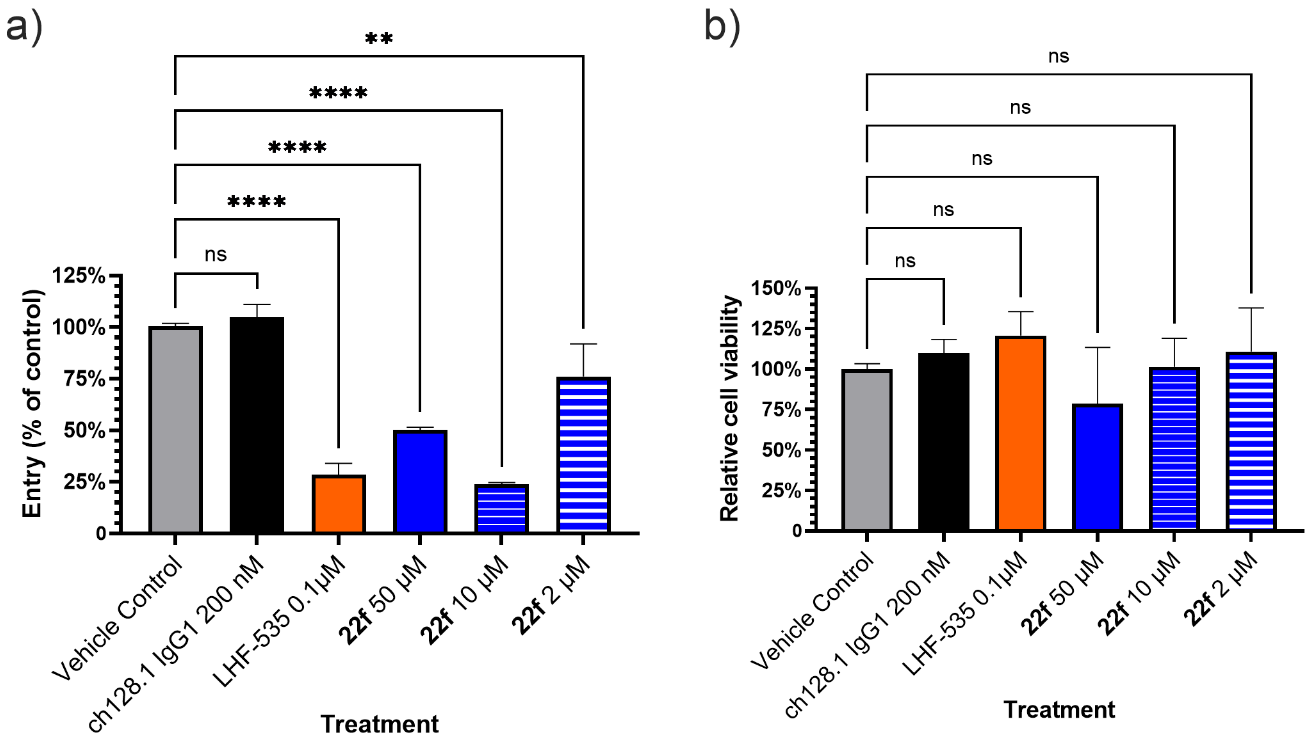

Figure S4. **a)** Inhibition of viral entry by pseudotyped MLV expressing GPC for LASV into HEK 293T/T17 with **22f** (2-50 μM). The TfR1 apical domain-binding ch128.1 antibody was included as a negative control and the fusion inhibitor LHF-535 was included as the positive control, as a known LASV entry inhibitor. Viral entry results represent reduction in entry by **22f** compared to the vehicle control. **b)** Cytotoxicity was determined in parallel by measuring the viability of treated cells using the CellTiter-Glo Luminescent Cell Viability Assay, with luminescence units normalized to average vehicle control luminescence for each experiment. Data shown are the means ± SD from two separate experiments. ****$p < 0.0001$, **$p < 0.01$

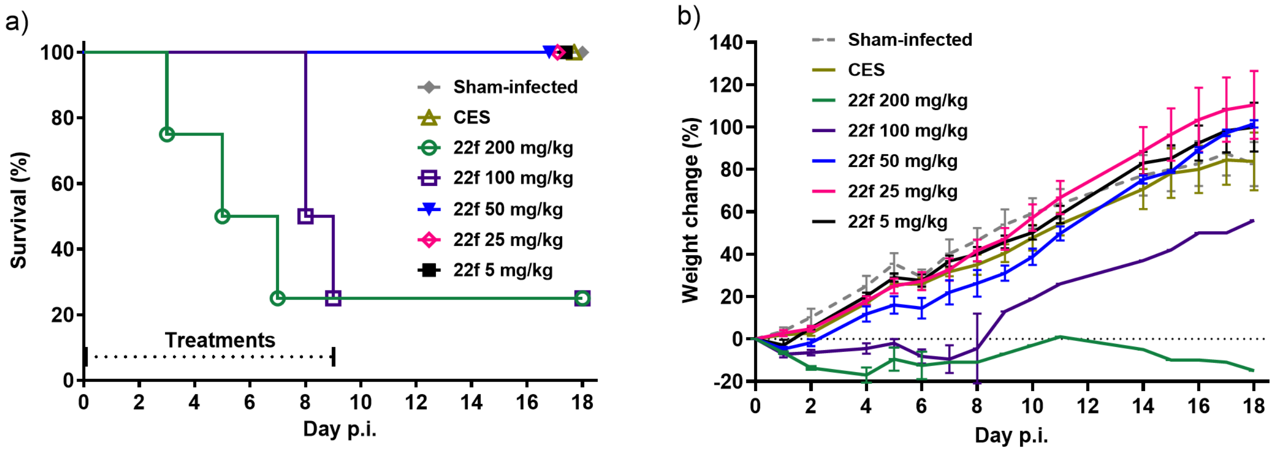

Figure S5. Weanling hTfR1 mouse tolerance of **22f** treatment. Animals in each group (*n* = 4/treatment dose) were treated by intraperitoneal (i.p.) injection with the indicated dose of SW-177 or CES (10% cremophor, 10% ethanol, 80% saline) vehicle placebo for 10 days. a) Survival and b) daily body weights during the 18-day study. The weight data are represented as the group mean and standard error of the percent change in weight of animals relative to their starting weights on the day of treatment initiation. Untreated, normal control animals (*n* = 2) are shown for comparison.

Table S1. Antiviral effect of the virtual hits and hit analogues purchased from SPECS on the replication of JUNV in A549 cells. Virus yield reductions were determined by endpoint dilution of culture supernatants collected from primary and confirmatory 4- and 8-concentration assays, respectively, on Vero cells. Favipiravir was used as the positive control in this assay. [1]

Virtual hits purchased from SPECS

Compound	*EC90 (μM)* [a,b]	*CC50 (μM)* [b,c]	*SI*[d]
AE-848/32605045	>81	81	1
AN-329/12928029	>100	>100	1
AG-219/11639001	>100	>100	1
AP-263/43418057	>100	>100	1
AJ-292/40706483	>100	>100	1
AQ-086/43457605	>100	>100	1
AN-465/41400746	>100	>100	1
AN-329/43211402	>100	>100	1
AH-487/42145044	29	83	2.8
AK-918/15589010	>100	>100	1
AJ-292/40706485	>100	>100	1
AO-022/43455326	>42	42	1
AG-205/15155107	>100	>100	1
AT-057/41986952	9.0<br>19	>100<br>>100	>11<br>>5.2
**1a**<br>AK-968/10741039	**3.2**<br>**2.4**	**51**<br>**>100**	**15**<br>**>42**
**2**<br>AP-853/42181282	**0.68**<br>**1.4**	**>100**<br>**>100**	**>147**<br>**>73**
AP-501/43397644	>100	>100	1
AN-698/42147468	8.9<br>7.0	>100<br>>100	>11<br>>14
AQ-432/42260206	9.1<br>>100	62<br>>100	6.8<br>1
AN-329/43465324	>100	>100	1
AE-848/08585010	>100	>100	1
AP-970/43012386	>100	>100	1
AP-970/43375520	>100	>100	1
AN-584/43507472	>100	>100	1
AP-970/13289175	13	>100	>7.7
Favipiravir (positive control)	1.1<br>1.4	>100<br>>100	>89<br>>71

Commercial analogues (SPECS) of compound 1a

Compound	*EC90 (μM)* [a,b]	*CC50 (μM)* [b,c]	*SI*[d]
**1b**<br>AH-487/40686872	**4.6**<br>**1.9**	**>100**<br>**>320**	**>22**<br>**>171**
AN-329/10735045	11	>100	>9.1
AN-329/10737009	13	17	1.3
AN-329/11378285	58	>100	>1.7
AN-329/11482546	>42	42	1
AN-329/11482572	5.9	52	8.8
AK-968/41924900	42	>100	>2.4
Favipiravir (positive control)	2.6<br>2.7	>100<br>>320	>38<br>>119

[a] EC_{90} = 90% effective concentration (concentration at which virus yield is reduced by one log_{10}).
[b] Data are the results of 4-concentration assays. For selected compounds, a second 8-concentration test was performed to confirm initial results, and the data are shown underneath the initial 4-concentration test results.
[c] CC_{50} = 50% cytotoxic concentration (concentration at which 50% adverse effect is observed on the host cell).
[d] SI = the ratio of CC_{50} to EC_{90}.

Table S2. Additional antiviral activity studies to confirm that the antiviral of compound **22f** versus JUNV and TCRV in A549 cells by 8-concentration VYR assay. The ch128.1 IgG1 antibody was used as the positive control in this assay

Compound	Assay	JUNV			TCRV		
		EC_{90} [a]	*CC_{50}* [b]	*SI_{90}* [c]	*EC_{90}* [a]	*CC_{50}* [b]	*SI_{90}* [c]
**22f** (μM)	**VYR #1**	0.30	>100	>333	1.43	>100	>70
	**VYR #2**	0.24	>100	>417	1.15	>100	>87
**ch128.1 IgG1** (nM)	**VYR #1**	0.13	>200	>1538	>200	>200	1
	**VYR #2**	0.10	>200	>2000	>200	>200	1

[a] EC_{90} = 90% effective concentration (concentration at which virus yield is reduced by one log_{10}).
[b] CC_{50} = 50% cytotoxic concentration (concentration at which 50% adverse effect is observed on the host cell).
[c] SI = the ratio of CC_{50} to EC_{90}.

Table 3. *In vitro* rat liver microsomal stability ($t_1/_2$) of selected antiviral compounds and verapamil as a positive control.

Compound	Scaffold	$t_1/_2$ (min) ± SD [a-c]	Observations
**22f**	A- hydrazone	39.80 ± 1.23	High potency, good stability
**91**	F- oxalic amide	30.26 ± 1.07	Moderate potency, good stability
**97**	G- acrylbenzylamide	20.11 ± 0.69	High potency, moderate stability
**verapamil**	-	5.48 ± 0.65	Literature $t_1/_2$ in rat liver microsomes ~5–6 min[2]

[a] Assay conditions: 0.5 mg/mL rat liver microsomes, NADPH-regenerating system, 37 °C.
[b] Half-life calculated from first-order depletion kinetics over 0–30 min.
[c] SD = standard deviation from *n* = 3 independent determinations.

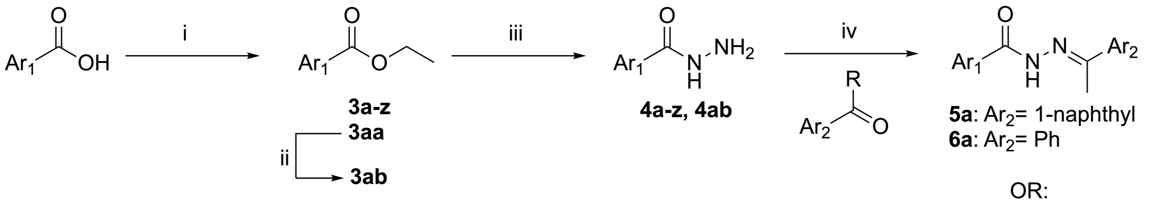

OR:

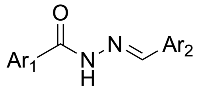

Ar$_1$

**a**= 2,4-OH-Ph	**p**= 4-*t*Bu-Ph
**c**= 2,4-OMe-Ph	**q**= 4-Ph-Ph
**d**= 4-OH-Ph	**r**= 4-Me-Ph
**e**= 2-OH-Ph	**s**= 2,5-OH-Ph
**f**= 2,4-Me-Ph	**t**= 2,5-Me-Ph
**g**= 2,4-F-Ph	**u**= 2-OH-4-Me-Ph
**h**= 2,4-Cl-Ph	**v**= 2-Me-4-OH-Ph
**i**= 2,4-CF_3-Ph	**w**= 3,5-OH-Ph
**j**= 4-pyridine	**x**= 3,4-OH-Ph
**k**= 2-pyridine	**y**= 2,6-OH-Ph
**l**= 2-furan	**z**= 3,4-Cl-Ph
**m**= 2-(1H-imidazole)	**aa**= (2-Me-4-Br)-Ph
**n**= 3,5-Me-Ph	**ab**= (2-Me-4-Ph)-Ph
**o**= 4-*i*Pr-Ph	

Ar$_2$

**1a, 1c-w, 1ab**: 1-naphthyl	**20a**: 4-CF_3-Ph	**34a**: 4-cyclopropyl-Ph
**7a**: Ph	**21a**: 4-SF_5-Ph	**35a**: 3-Cl-4-CF_3-Ph
**8a**: 3-(1H-indole)	**22a, 22f**: 4-*t*Bu-Ph	**36a**: 3-Me-4-CF_3-Ph
**9a**: 2-(1H-indole)	**23a**: 3-Cl-5-CF_3-Ph	**37a**: 4-cyclohexyl-Ph
**10a**: 4-quinoline	**24a**: 3-OMe-5-CF_3-Ph	**38a**: 4-cyclopentyl-Ph
**11a**: 5-isoquinoline	**25a**: 2,4-Cl-Ph	**39a**: 4-cyclobutyl-Ph
**12a**: 2-(1H-imidazole)	**26a**: 4-CN-Ph	**40a**: 2-OH-1-naphthyl
**13a**: 2-furan	**27a**: 3,4-Me-Ph	**41a**: 4-OH-1-naphthyl
**14a**: 4-OH-Ph	**28a**: 4-OMe-Ph	**42a**: 1-OH-2-naphthyl
**15a**: 4-pyridine	**29a**: 4-Ph-Ph	**43a**: 8-OH-1-naphthyl
**16a**: 3-pyridine	**30a, 30f**: 2,3-F-Ph	**44a, 44f**: 5-OH-1-naphthyl
**17a**: 3,4-F-Ph	**31a, 31f**: 2,3-Cl-Ph	**45f**: 2,3-Me-Ph
**18a, 18f, 18u-y, 18ab**: 3,4-Cl-Ph	**32f**: 2-napthtyl	**46z**: 2,4-OH-Ph
**19a**: 4-Cl-Ph	**33f**: 4-*i*Pr-Ph	

Scheme S1. Preparation of hydrazone analogues of **1a**. *Reagents and conditions*: (i) H_2SO_4, EtOH, reflux, 4-12 hours (80-99%); (ii) phenylboronic acid, $PdCl_2$, K_2CO_3, PhMe/MeOH (9:1), 60 °C, 3 hours (76%); (iii) NH_2NH_2*H_2O, EtOH, reflux, o.n. (56-98%); (iv) EtOH, reflux, o.n. (36-94%).

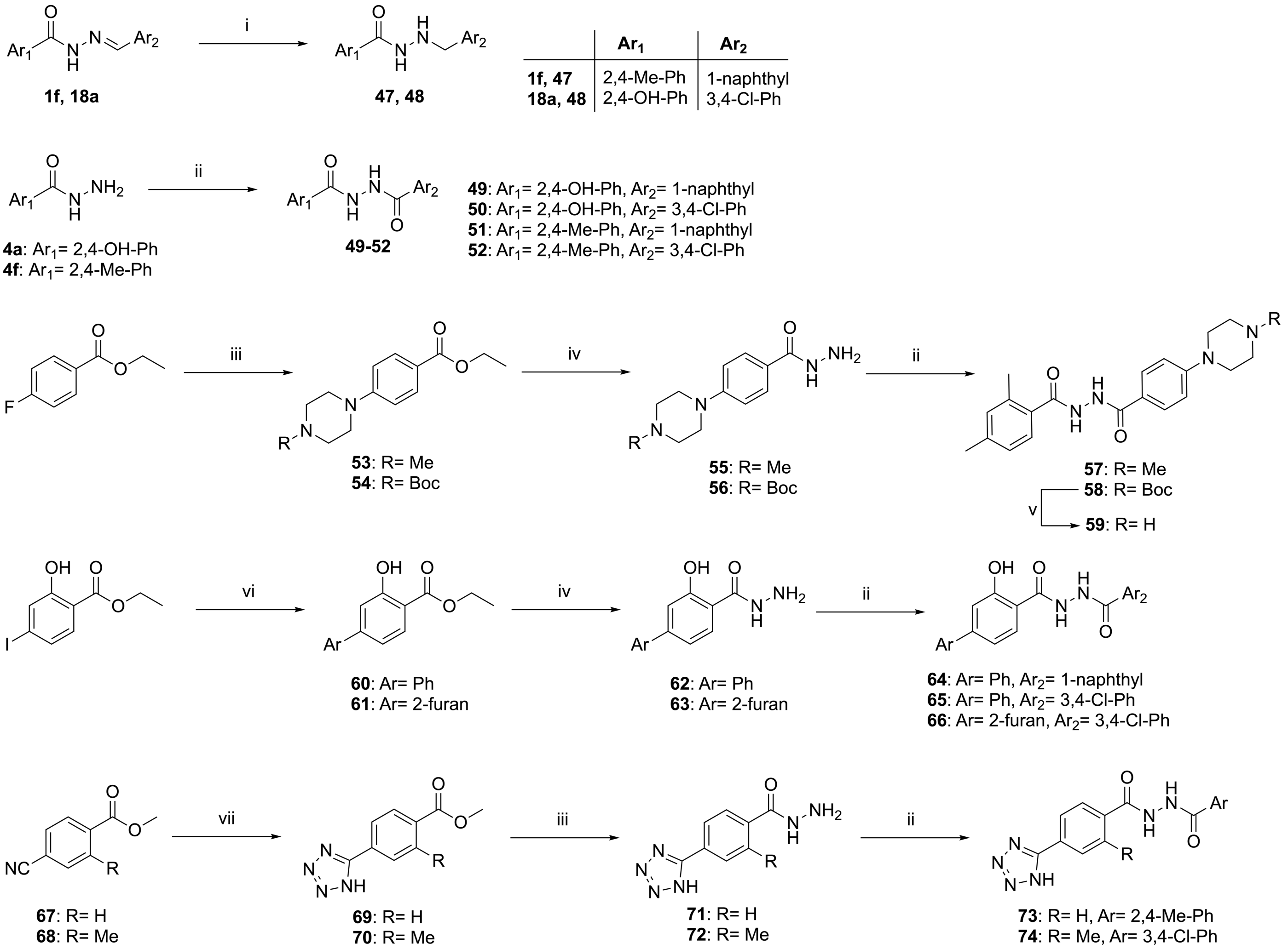

Scheme S2. Preparation of target compounds **47-52**, **57**, **59**, **64-66** and **73-74**. *Reagents and conditions*: (i) triethylsilane, trifluoroacetic acid, 0 °C, 16 hours (48-75%); (ii) appropriate aromatic acyl chloride, Et_3N, THF, 4-16 hours (37-95%); (iii) 1-methylpyperazine or 1-Boc-pyperazine, K_2CO_3, DMSO, 110 °C, 12-24 hours (74-87%); (iv) NH_2NH_2*H_2O, EtOH, reflux, o.n. (56-98%); (v) TFA, DCM, r.t., 3 hours (79%); (vi) phenylboronic acid or 2-furylboronic acid, $PdCl_2$, K_2CO_3, PhMe/MeOH (9:1), 60 °C, 3 hours (64-66%); (vii) NaN_3, NH_4Cl, DMF, reflux, 12-24 hours (85-87%).

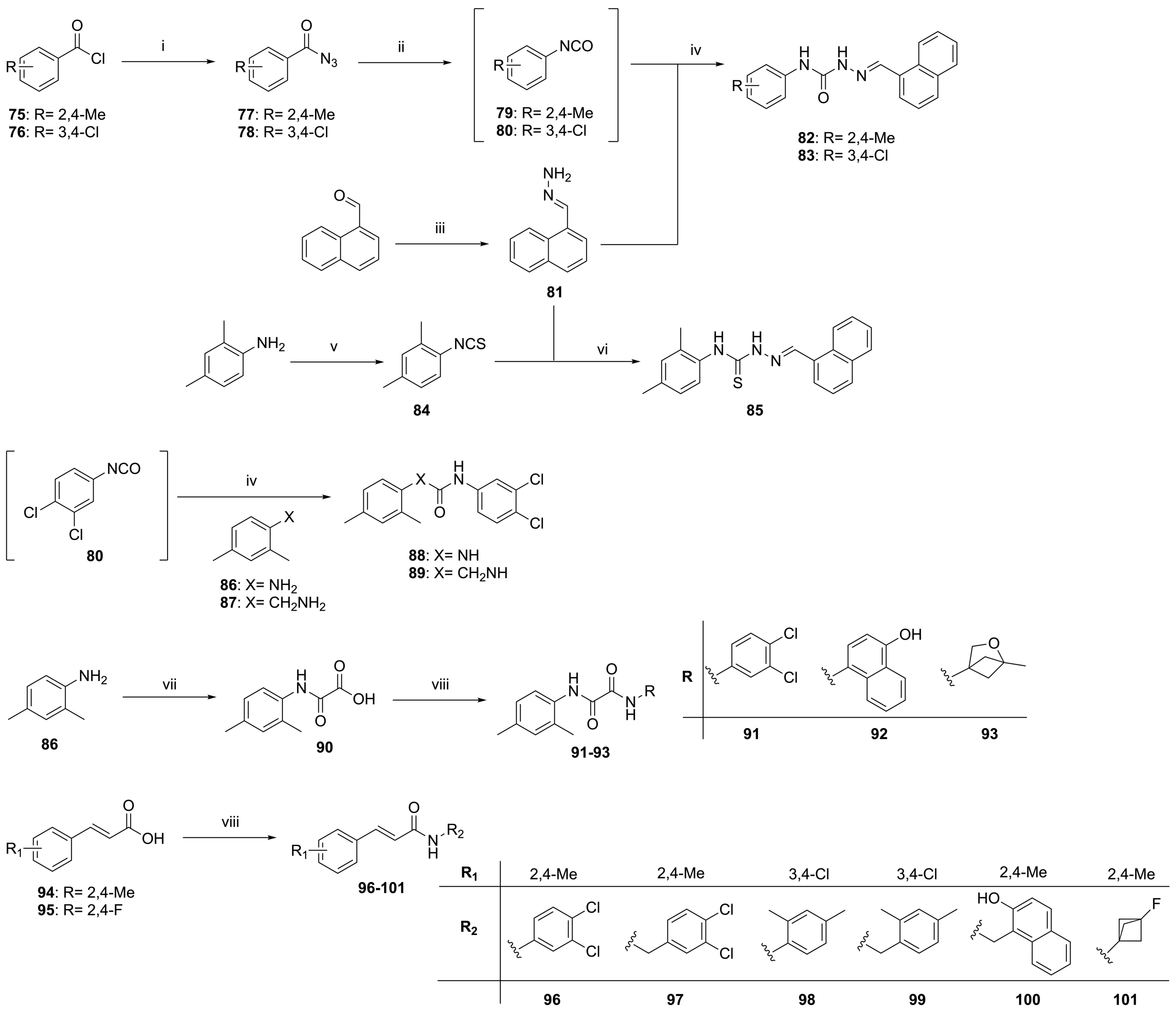

Scheme S3. Preparation of target compounds **82-83**, **85**, **88-89**, **91-93** and **96-101**. *Reagents and conditions*: (i) sodium azide, MeOH/H_2O, 0 °C to r.t., 16 hours (70-78%); (ii) PhMe, reflux, 4 hours; (iii) NH_2NH_2*H_2O, EtOH, reflux, o.n. (83-93%); (iv) r.t., 16 hours (47-99%); (v) $CSCl_2$, Et_3N, THF, 0 °C to r.t., 1 hour (99%); (vi) PhMe, r.t., o.n (84%); (vii) a. ethyl oxalyl chloride, Et_3N, DCM (97%), b. KOH, EtOH/H_2O, r.t., 1 hours (93%); (viii) appropriate amine, TBTU, DiPEA, THF, 4-24 hours (67-94%).

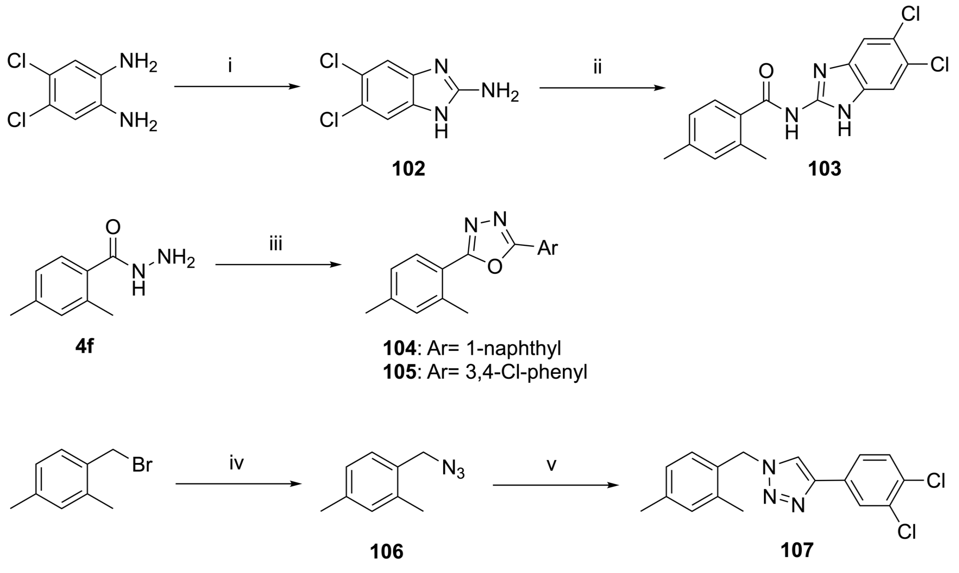

Scheme S4. Preparation of target compounds **103-105** and **107**. *Reagents and conditions*: (i) BrCN, MeOH/H_2O, 50 °C, 1 hours (73%); (ii) 2,4-dimethylbenzoic acid, TBTU, DiPEA, THF, 24 hours (69%); (iii) 1-naphthylaldehyde, NaOCl, *t*-BuOH, reflux, 2 hours, then r.t. to 30 °C, o.n. (33%) OR 3,4-dichlorobenzoic acid, $POCl_3$, reflux, o.n. (46%); (iv) sodium azide, DMF, r.t., 24 hours (95%); (v) 1,2-dichloro-4-ethynylbenzene, CuSO4, Na ascorbate, THF/H_2O (9:1), r.t., o.n. (84%).

Procedures for the preparation and characterisation of final target products

General procedure for the preparation of hydrazones 1a, 1c-w, 1ab, 5a-44a, 18f, 18u-y, 18ab, 30f, 31f, 44f, 45f, 46z

The appropriate aromatic hydrazide **4a-z** or **4ab** (0.5 mmol, 1 eq.) was dissolved in ethanol (5 mL). The appropriate, differently substituted aromatic aldehyde or ketone (0.6 mmol, 1.2 eq.) was then added portion wise with stirring. The solution was heated to reflux until completion (monitored by TLC), then cooled to room temperature and the precipitate formed was isolated by *vacuum* filtration and washed with cold ethanol and 40-60 petroleum ether. The solid product was purified by recrystallisation or flash column chromatography.

- 2,4-Dihydroxy-*N′*-(naphthalen-1-ylmethylene)benzohydrazide **1a**

Purified by recrystallisation from EtOH. Obtained as a white solid in 58% yield. One single species (E/Z isomerism) observed in NMR experiments. ^{1}H-NMR (DMSO-d$_6$), δ: 12.37 (s, 1H), 11.80 (s, 1H), 10.27 (s, 1H), 9.10 (s, 1H), 8.92 (d, J= 8.2 Hz, 1H), 8.03 (app t, J= 7.4 Hz, 2H), 7.95 (d, J= 7.1 Hz, 1H), 7.86 (d, J= 8.7 Hz, 1H), 7.68 (t, J= 7.1 Hz, 1H), 7.64-7.60 (m, 2H), 6.41 (dd, J_1= 8.8 Hz, J_2= 2.3 Hz, 1H), 6.35 (d, J= 2.3 Hz, 1H). ^{13}C-NMR (DMSO-d$_6$), δ: 165.9, 163.2, 162.7, 148.4, 134.1, 131.1, 130.7, 130.1, 129.9, 129.2, 128.5, 127.9, 126.8, 126.1, 124.9, 107.9, 106.7, 103.4. UPLC-MS: t_R 2.01 min, MS [ESI, m/z]: 307.1 [M+H$^+$]. HRMS calculated for $C_{18}H_{15}N_2O_3^+$: 307.1077; found 307.1071.

- 2,4-Dimethoxy-*N′*-(naphthalen-1-ylmethylene)benzohydrazide **1c**

Purified by recrystallisation from EtOH. Obtained as a white solid in 50% yield. Melting point: 163-165 °C. One single species (E/Z isomerism) observed in NMR experiments. ^{1}H-NMR (DMSO-d$_6$), δ: 10.87 (s, 1H), 8.95 (s, 1H), 8.81 (d, J= 8.7 Hz, 1H), 8.34 (d, J= 8.7 Hz, 1H), 8.04 (d, J= 6.9 Hz, 1H), 7.90 (t, J= 6.9 Hz, 2H), 7.64-7.61 (m, 1H), 7.53 (q, J= 7.9, 15.6 Hz, 2H), 6.68 (q, J= 2.3 Hz, 8.8 Hz, 1H), 6.55 (s, 1H), 4.07 (s, 3H), 3.89 (s, 3H). ^{13}C-NMR (DMSO-d$_6$), δ: 164.0, 162.0, 158.6, 146.9, 134.7, 133.8, 130.8, 129.7, 128.8, 127.9, 127.3, 126.1, 125.3, 124.3, 113.4, 105.9, 98.8, 56.2, 55.6. UPLC-MS: t_R 2.16 min, MS [ESI, m/z]: 335.1 [M+H$^+$]. HRMS calculated for $C_{20}H_{19}N_2O_3^+$: 335.1390; found 335.1393.

- 4-Hydroxy-*N′*-(naphthalen-1-ylmethylene)benzohydrazide **1d**[3]

Purified by recrystallisation from EtOH. Obtained as a white solid in 39% yield. Melting point: 230-232 °C. One single species (E/Z isomerism) observed in NMR experiments. ^{1}H-NMR (DMSO-d$_6$), δ: 11.73 (s, 1H), 10.16 (s, 1H), 9.09 (s, 1H), 8.86 (d, J= 6.9 Hz, 2H), 8.02 (d, J= 8.3 Hz, 2H), 7.92 (d, J= 6.91 Hz, 1H), 7.86 (d, J= 8.3 Hz, 1H), 7.67-7.59 (m, 2H), 6.89 (d, J= 8.3 Hz, 2H). ^{13}C-NMR (DMSO-d$_6$), δ: 164.7, 157.4, 146.5, 133.4, 131.7, 130.6, 129.6, 128.0, 127.8, 127.7, 126.4, 125.9, 124.7, 115.7. UPLC-MS: t_R 1.88 min, MS [ESI, m/z]: 291.3 [M+H$^+$]. HRMS calculated for $C_{18}H_{15}N_2O_2^+$: 291.1128; found 291.1136.

- 2-Hydroxy-*N*'-(naphthalen-1-ylmethylene)benzohydrazide **1e**

Purified by flash column chromatography eluting with *n*-hexane-ethyl acetate 90:10 v/v increasing to *n*-hexane-ethyl acetate 30:70 v/v in 15 CV. Obtained as a white solid in 80% yield. Melting point: 204-206 °C. One single species (E/Z isomerism) observed in NMR experiments. ^{1}H-NMR (DMSO-d$_6$), δ: 11.96 (s, 1H), 10.63 (s, 1H), 9.12 (s, 1H), 8.93 (s, 1H), 8.05 (s, 2H), 7.96 (s, 2H), 7.70 (s, 1H), 7.65-7.61 (m, 2H), 7.48 (s, 1H), 7.02-6.99 (m, 2H). ^{13}C-NMR (DMSO-d$_6$), δ: 167.4, 159.8, 146.5, 133.4, 130.6, 129.4, 128.4, 128.0, 127.8, 126.4, 125.9, 124.7, 116.8, 114.1. UPLC-MS: t_R 1.86 min, MS [ESI, m/z]: 291.1 [M+H$^+$]. HRMS calculated for $C_{18}H_{15}N_2O_2^+$: 291.1128; found 291.1125.

- 2,4-Dimethyl-*N*'-(naphthalen-1-ylmethylene)benzohydrazide **1f**

Purified by recrystallisation from EtOH. Obtained as a white solid in 63% yield. Melting point: 208-210 °C. Two species (E/Z isomerism) observed in NMR experiments, in a 1:0.4 ratio. ^{1}H-NMR (DMSO-d$_6$), δ: 11.82 (s, 1.4H), 9.02 (s, 1H), 8.92 (d, J= 8.5 Hz, 1H), 8.65 (s, 0.4H), 8.41 (d, J= 8.5 Hz, 0.4H), 8.09-8.07 (m, 2H), 7.99-7.96 (m, 1H), 7.73 (s, 1.4H), 7.68-7.65 (m, 2.8H), 7.56 (bs, 0.8H), 7.49 (s, 1H), 7.38 (m, 0.8H) 7.19 (m, 2.8H) 2.45 (s, 3H), 2.43 (s, 1.2H), 2.39 (s, 3H), 2.31 (s, 1.2H). ^{13}C-NMR (DMSO-d$_6$), δ: 172.2, 165.6, 147.5, 140.2, 136.7, 134.0, 132.7, 131.9, 130.6, 129.3, 128.1, 127.7, 126.6, 124.7, 21.3, 19.9. UPLC-MS: t_R 2.27 min, MS [ESI, m/z]: 303.1 [M+H$^+$]. HRMS calculated for $C_{20}H_{19}N_2O^+$: 303.1492; found 303.1503.

- 2,4-Difluoro-*N*'-(naphthalen-1-ylmethylene)benzohydrazide **1g**

Purified by recrystallisation from EtOH. Obtained as a white solid in 81% yield. Melting point: 217-219 °C. Two species (E/Z isomerism) observed in NMR experiments, in a 1:0.6 ratio. ^{1}H-NMR (DMSO-d$_6$), δ: 12.07 (s, 0.6H), 11.93 (s, 1H), 8.98 (s, 1H), 8.87 (d, J= 8.6 Hz, 1H), 8.64 (s, 0.6H), 8.42 (d, J= 8.6 Hz, 0.6H), 8.05-8.02 (m, 2H), 7.96-7.93 (m, 2H), 7.86-7.81 (m, 1H), 7.70-7.60 (m, 4.6H), 7.55-7.38 (m, 3H), 7.29-7.24 (m, 1.6 H). ^{13}C-NMR (DMSO-d$_6$), δ: 166.9, 160.1, 148.7, 145.4, 134.0, 121.3, 130.4, 129.7, 129.2, 128.7, 127.5, 126.0, 124.7, 105.3. ^{19}F-NMR (DMSO-d$_6$), δ: -107.26, -114.67. UPLC-MS: t_R 1.89 min, MS [ESI, m/z]: 311.1 [M+H$^+$]. HRMS calculated for $C_{18}H_{13}F_2N_2O^+$: 311.0990; found 311.0984.

- 2,4-Dichloro-*N*'-(naphthalen-1-ylmethylene)benzohydrazide **1h**

Purified by recrystallisation from EtOH. Obtained as a white solid in 87% yield. Melting point: 219-221 °C. One single species (E/Z isomerism) observed in NMR experiments. ^{1}H-NMR (DMSO-d$_6$), δ: 12.10 (s, 1H), 8.90 (s, 1H), 8.30 (d, J= 8.6 Hz, 1H), 8.07-8.03 (m, 2H), 7.96-7.92 (m, 1H), 7.83-7.81 (m, 1H), 7.71-7.68 (m, 1H), 7.64-7.57 (m, 1H), 7.54-7.51 (m, 2H), 7.30-7.27 (m, 1H). ^{13}C-NMR (DMSO-d$_6$), δ: 168.5, 162.1, 148.9, 145.7, 135.9, 134.7, 134.0, 132.3, 131.4, 131.1, 130.1, 129.9, 129.1, 127.9, 127.3, 126.7, 125.9, 125.0. UPLC-MS: t_R 2.36 min, MS [ESI, m/z]: 343.1 [M+H$^+$]. HRMS calculated for $C_{18}H_{13}Cl_2N_2O^+$: 343.0399; found 343.0407.

- *N*'-(Naphthalen-1-ylmethylene)-2,4-bis(trifluoromethyl)benzohydrazide **1i**

Purified by recrystallisation from EtOH. Obtained as a white solid in 36% yield. Melting point: 162-165 °C. One single species (E/Z isomerism) observed in NMR experiments. ^{1}H-NMR (DMSO-d$_6$), δ: 12.29 (s, 1H), 8.91-8.87 (m, 1H), 8.53 (s, 1H), 8.28-8.25 (m, 2H), 8.11-8.03 (m, 1H), 7.94-7.91 (m, 2H), 7.71-7.47 (m, 3H), 7.12 (s, 1H). ^{13}C-NMR (DMSO-d$_6$), δ: 168.7, 146.2, 134.0, 131.3, 130.4, 130.2, 129.2, 127.0, 126.6, 125.9, 124.8. ^{19}F-NMR (DMSO-d$_6$), δ: -58.97, -61.45. UPLC-MS: t_R 2.36 min, MS [ESI, m/z]: 411.1 [M+H$^+$]. HRMS calculated for $C_{20}H_{13}F_6N_2O^+$: 411.0927; found 411.0934.

- *N*'-(Naphthalen-1-ylmethylene)isonicotinohydrazide **1j**[4]

Purified by recrystallisation from EtOH. Obtained as a white solid in 43% yield. Melting point: 188-191 °C. One single species (E/Z isomerism) observed in NMR experiments. ^{1}H-NMR (DMSO-d$_6$), δ: 12.15 (s, 1H), 9.11 (s, 1H), 8.88 (d, J= 7.7 Hz, 1H), 8.83 (d, J= 6.0 Hz, 2H), 8.07-8.03 (m, 2H), 7.96 (d, J= 7.7 Hz, 1H), 7.89 (d, J= 6.0 Hz, 2H), 7.73-7.68 (m, 1H), 7.64-7.61 (m, 2H). ^{13}C-NMR (DMSO-d$_6$), δ: 162.1, 150.9, 149.5, 141.0, 134.0, 131.4, 130.7, 129.7, 129.3, 128.7, 128.0, 126.1, 124.7, 122.0. UPLC-MS: t_R 1.55 min, MS [ESI, m/z]: 276.1 [M+H$^+$]. HRMS calculated for $C_{17}H_{14}N_3O^+$: 276.1131; found 276.1140.

- *N*'-(Naphthalen-1-ylmethylene)picolinohydrazide **1k**

Purified by recrystallisation from EtOH. Obtained as a white solid in 61% yield. Melting point: 176-179 °C. One single species (E/Z isomerism) observed in NMR experiments. ^{1}H-NMR (DMSO-d$_6$), δ: 12.26 (s, 1H), 9.42 (s, 1H), 8.83 (d, J= 8.5 Hz, 1H), 8.75 (d, J= 4.5 Hz, 1H), 8.19 (d, J= 7.7 Hz, 1H), 8.08 (t, J= 7.7 Hz, 1H), 8.01 (q, J= 8.5, 18.7 Hz, 3H), 7.68 (m, 2H), 7.61 (m, 2H). ^{13}C-NMR (DMSO-d$_6$), δ: 160.8, 150.0, 149.2, 149.0, 138.6, 134.0, 131.1, 131.0, 130.2, 127.7, 127.6, 126.7, 126.1, 124.5, 123.2. UPLC-MS: t_R 1.79 min, MS [ESI, m/z]: 276.1 [M+H$^+$]. HRMS calculated for $C_{17}H_{14}N_3O^+$: 276.1131; found 276.1125.

- *N*'-(Naphthalen-1-ylmethylene)furan-2-carbohydrazide **1l**

Purified by recrystallisation from EtOH. Obtained as a white solid in 72% yield. Melting point: 188-192 °C. One single species (E/Z isomerism) observed in NMR experiments. ^{1}H-NMR (DMSO-d$_6$), δ: 11.94 (s, 1H), 9.15 (s, 1H), 8.81 (d, 1H, J= 7.5 Hz), 8.03 (t, J= 7.5 Hz, 2H), 7.99 (s, 1H), 7.95 (d, J= 7.5 Hz, 1H), 7.68 (t, J= 7.5 Hz, 1H), 7.64-7.60 (m, 2H), 7.53 (s, 1H), 6.74 (s, 1H). ^{13}C-NMR (DMSO-d$_6$), δ: 154.7, 148.1, 147.2, 146.4, 134.0, 131.1, 130.7, 130.0, 129.3, 128.0, 127.8, 126.8, 126.1, 124.5, 115.5, 112.7. UPLC-MS: t_R 1.72 min, MS [ESI, m/z]: 265.1 [M+H$^+$]. HRMS calculated for $C_{16}H_{13}N_2O_2^+$: 265.0972; found 265.0975.

- *N*'-(Naphthalen-1-ylmethylene)-1*H*-imidazole-2-carbohydrazide **1m**

Purified by recrystallisation from EtOH. Obtained as a white solid in 74% yield. Melting point: 264-267 °C. One single species (E/Z isomerism) observed in NMR experiments. ^{1}H-NMR (DMSO-d$_6$), δ: 13.33 (s, 1H), 12.08 (s, 1H), 9.34 (s, 1H), 8.72 (d, J= 8.5 Hz, 1H), 8.04-7.98 (m, 3H), 7.67 (t, J= 7.6 Hz, 1H), 7.61 (q, J= 6.8, 13.8 Hz, 2H), 7.30 (bs, 2H). ^{13}C-NMR (DMSO-d$_6$), δ: 155.2, 148.1, 140.4, 134.0, 130.9, 130.1, 129.7, 129.3, 127.7, 127.2, 126.7, 126.1, 124.3, 121.2. UPLC-MS: t_R 1.58 min, MS [ESI, m/z]: 265.1 [M+H$^+$]. HRMS calculated for $C_{15}H_{13}N_4O^+$: 265.1084; found 265.1079.

- 3,5-Dimethyl-*N*'-(naphthalen-1-ylmethylene)benzohydrazide **1n**

Purified by recrystallisation from EtOH. Obtained as a white solid in 38% yield. Melting point: 232-233 °C. One single species (E/Z isomerism) observed in NMR experiments. ^{1}H-NMR (DMSO-d$_6$), δ: 11.86 (s, 1H), 9.13 (s, 1H), 8.85 (d, J= 8.5 Hz, 1H), 8.05-8.02 (m, 2H), 7.95 (d, J= 7.3 Hz, 1H), 7.69 (t, J= 7.6 Hz, 1H), 7.64-7.60 (m, 2H), 7.59 (s, 2H), 7.26 (s, 1H), 2.38 (s, 6H). ^{13}C-NMR (DMSO-d$_6$), δ: 163.7, 147.8, 138.2, 134.0, 133.9, 133.6, 131.0, 130.7, 130.1, 129.3, 128.0, 127.8, 126.8, 126.1, 125.8, 124.6, 21.4. UPLC-MS: t_R 2.47 min, MS [ESI, m/z]: 303.1 [M+H$^+$]. HRMS calculated for $C_{20}H_{19}N_2O^+$: 303.1492; found 303.1496.

- 4-Isopropyl-*N*'-(naphthalen-1-ylmethylene)benzohydrazide **1o**

Purified by recrystallisation from EtOH. Obtained as a white solid in 47% yield. Melting point: 221-221 °C. One single species (E/Z isomerism) observed in NMR experiments. ^{1}H-NMR (DMSO-d$_6$), δ: 11.88 (s, 1H), 9.12 (s, 1H), 8.89 (d, J= 8.4 Hz, 1H), 8.03 (bs, 1H), 7.95-7.91 (m, 4H), 7.69 (t, J= 7.2 Hz, 2H), 7.62 (bs, 1H), 7.44 (d, J= 7.9 Hz, 2H), 3.04-2.95 (m, 1H) 1.26 (s, 3H), 1.25 (s, 3H). ^{13}C-NMR (DMSO-d$_6$), δ: 163.5, 153.0, 147.8, 134.0, 131.0, 129.3, 128.2, 128.1, 127.8, 126.9, 126.8,

126.1, 124.7, 33.9, 31.2, 24.1. UPLC-MS: t_R 2.01 min, MS [ESI, m/z]: 317.0 [M+H$^+$]. HRMS calculated for $C_{21}H_{21}N_2O^+$: 317.1648; found 317.1650.

- 4-(*tert*-Butyl)-*N*'-(naphthalen-1-ylmethylene)benzohydrazide **1p**

Purified by recrystallisation from EtOH. Obtained as a white solid in 81% yield. Melting point: 220-222 °C. One single species (E/Z isomerism) observed in NMR experiments. ^{1}H-NMR (DMSO-d$_6$), δ: 11.89 (s, 1H), 9.12 (s, 1H), 8.89 (d, J= 8.2 Hz, 1H), 8.05-8.02 (m, 2H), 7.95-7.92 (m, 3H), 7.69 (t, J= 8.2 Hz, 1H), 7.64-7.57 (m, 4H), 1.34 (s, 9H). ^{13}C-NMR (DMSO-d$_6$), δ: 163.5, 155.2, 147.9, 134.0, 131.1, 131.0, 130.7, 130.1, 129.3, 128.2, 128.0, 127.8, 126.8, 124.1, 125.8, 124.7, 35.2, 31.4. UPLC-MS: t_R 2.52 min, MS [ESI, m/z]: 331.1 [M+H$^+$]. HRMS calculated for $C_{22}H_{23}N_2O^+$: 331.1805; found 331.1811.

- *N*'-(Naphthalen-1-ylmethylene)-[1,1'-biphenyl]-4-carbohydrazide **1q**

Purified by recrystallisation from EtOH. Obtained as a white solid in 79% yield. Melting point: 223-226 °C. One single species (E/Z isomerism) observed in NMR experiments. ^{1}H-NMR (DMSO-d$_6$), δ: 12.02 (s, 1H), 9.16 (s, 1H), 8.91 (d, J= 8.4 Hz, 2H), 8.10 (d, J= 8.4 Hz, 2H), 8.05 (t, J= 7.0 Hz, 2H), 7.96 (d, J= 7.0 Hz, 2H), 7.89 (d, J= 7.5 Hz, 2H), 7.70 (t, J= 7.5 Hz, 1H), 7.65-7.61 (m, 2H), 7.53 (t, J= 7.5 Hz, 2H), 7.44 (t, J= 7.5 Hz, 1H). ^{13}C-NMR (DMSO-d$_6$), δ: 163.2, 148.2, 143.8, 139.6, 134.1, 132.6, 131.1, 130.7, 130.1, 129.6, 129.3, 128.8, 128.7, 128.3, 127.8, 127.4, 127.2, 126.8, 124.7. UPLC-MS: t_R 2.47 min, MS [ESI, m/z]: 351.1 [M+H$^+$]. HRMS calculated for $C_{24}H_{19}N_2O^+$: 351.1492; found 351.1485.

- 4-Methyl-*N*'-(naphthalen-1-ylmethylene)benzohydrazide **1r**[5]

Purified by recrystallisation from EtOH. Obtained as a white solid in 94% yield. Melting point: 211-215 °C. One single species (E/Z isomerism) observed in NMR experiments. ^{1}H-NMR (DMSO-d$_6$), δ: 11.88 (s, 1H), 9.13 (s, 1H), 8.88 (s, 1H), 8.03-7.91 (m, 5H), 7.62 (bs, 3H), 7.37 (s, 2H), 2.41 (s, 3H). ^{13}C-NMR (DMSO-d$_6$), δ: 163.4, 147.8, 134.0, 131.0, 130.7, 130.1, 129.5, 129.3, 128.1, 127.8, 126.8, 126.2, 124.7, 21.5. UPLC-MS: t_R 2.34 min, MS [ESI, m/z]: 289.0 [M+H$^+$]. HRMS calculated for $C_{19}H_{17}N_2O^+$: 289.1335; found 289.1332.

- 2,5-Dihydroxy-*N*'-(naphthalen-1-ylmethylene)benzohydrazide **1s**

Purified by recrystallisation from EtOH. Obtained as a white solid in 51% yield. Melting point: 230-234 °C. One single species (E/Z isomerism) observed in NMR experiments. ^{1}H-NMR (DMSO-d$_6$), δ: 11.88 (s, 1H), 11.13 (s, 1H), 9.15 (s, 1H), 9.11 (s, 1H), 8.92 (d, J= 8.6 Hz, 1H), 8.04 (t, J= 8.6 Hz, 2H), 7.97 (d, J= 7.3 Hz, 1H), 7.69 (t, J= 7.3 Hz, 1H), 7.64-7.61 (m, 2H), 7.36 (s, 1H), 6.93 (d, J= 6.1 Hz, 1H), 6.86 (d, J= 8.6 Hz, 1H). ^{13}C-NMR (DMSO-d$_6$), δ: 164.8, 151.9, 150.1, 148.8, 134.0, 131.2, 130.7, 130.0, 129.3, 128.5, 127.8, 126.8, 126.0, 124.9, 121.9, 118.4, 116.7, 114.5. UPLC-MS: t_R 1.70 min, MS [ESI, m/z]: 307.0 [M+H$^+$]. HRMS calculated for $C_{18}H_{15}N_2O_3^+$: 307.1077; found 307.1073.

- 2,5-Dimethyl-*N*'-(naphthalen-1-ylmethylene)benzohydrazide **1t**

Purified by recrystallisation from EtOH. Obtained as a white solid in 91% yield. Melting point: 232-235 °C. Two species (E/Z isomerism) observed in NMR experiments, in a ratio 1:0.4. ^{1}H-NMR (DMSO-d$_6$), δ: 11.81 (s, 1.4H), 8.97 (s, 1H), 8.87 (d, J= 8.6 Hz, 1H), 8.57 (s, 0.4H), 8.39 (d, J= 8.6 Hz, 0.4H), 8.05-8.02 (m, 2H), 7.93 (t, J= 7.1 Hz, 1.8H), 7.69 (t, J= 7.1 Hz, 1H), 7.64-7.58 (m, 2.4H), 7.51 (d, J= 7.1 Hz, 0.8H), 7.35 (s, 1H), 7.30-7.21 (m, 3.2H), 7.19 (s, 0.4H), 2.38 (s, 3H), 2.35 (s, 3H), 2.34 (s, 1.2H), 2.25 (s, 1.2H). ^{13}C-NMR (DMSO-d$_6$), δ: 172.2, 165.7, 147.6, 144.7, 135.2, 134.0, 133.4, 131.1, 131.0, 130.6, 130.0, 129.3, 128.4, 128.3, 127.8, 126.8, 126.1, 124.7, 20.9, 19.4. UPLC-MS: t_R 1.96 min, MS [ESI, m/z]: 303.0 [M+H$^+$]. HRMS calculated for $C_{20}H_{19}N_2O^+$: 303.1492; found 303.1495.

- 2-Hydroxy-4-methyl-*N*'-(naphthalen-1-ylmethylene)benzohydrazide **1u**

Purified by recrystallisation from EtOH. Obtained as a white solid in 75% yield. Melting point: 235-239 °C. One single species (E/Z isomerism) observed in NMR experiments. ^{1}H-NMR (DMSO-d$_6$), δ: 12.04 (s, 1H), 11.93 (s, 1H), 9.13 (s, 1H), 8.93 (d, J= 8.6 Hz, 1H), 8.04 (t, J= 8.6 Hz, 2H), 7.97 (d, J= 7.2 Hz, 1H), 7.88 (d, J= 8.6 Hz, 1H), 7.69 (d, J= 7.2 Hz, 1H), 7.65-7.61 (m, 2H), 6.83 (bs, 2H), 2.33 (s, 3H). ^{13}C-NMR (DMSO-d$_6$), δ: 172.1, 165.6, 160.2, 154.2, 149.0, 135.3, 134.0, 133.6, 131.3, 131.4, 130.7, 129.9, 128.7, 128.6, 128.4, 127.9, 126.3, 124.7, 19.5. UPLC-MS: t_R 1.94 min, MS [ESI, m/z]: 305.1 [M+H$^+$]. HRMS calculated for $C_{19}H_{17}N_2O_2^+$: 305.1285; found 305.1287.

- 4-Hydroxy-2-methyl-*N*'-(naphthalen-1-ylmethylene)benzohydrazide **1v**

Purified by recrystallisation from DCM. Obtained as a white solid in 37% yield. Melting point: 237-240 °C. One single species (E/Z isomerism) observed in NMR experiments. ^{1}H-NMR (DMSO-d$_6$), δ: 11.65 (s, 1H), 9.84 (s, 1H), 8.97 (s, 1H), 8.86 (d, J= 7.7 Hz, 1H), 8.03 (d, J= 7.7 Hz, 2H), 7.91 (d, J= 7.7 Hz, 1H), 7.68 (s, 1H), 7.61 (s, 2H), 7.42 (d, J= 7.7 Hz, 1H), 6.71 (bs, 2H), 2.39 (s, 3H). ^{13}C-NMR (DMSO-d$_6$), δ: 165.5, 159.4, 146.9, 139.3, 134.0, 130.8, 130.1, 130.0, 129.3, 128.1, 127.8, 126.7, 126.0, 124.7, 118.0, 112.7, 20.4. UPLC-MS: t_R 1.69 min, MS [ESI, m/z]: 305.0 [M+H$^+$]. HRMS calculated for $C_{19}H_{17}N_2O_2^+$: 305.1285; found 305.1278.

- 3,5-Dihydroxy-*N*'-(naphthalen-1-ylmethylene)benzohydrazide **1w**

Purified by recrystallisation from DCM. Obtained as a white solid in 89% yield. One single species (E/Z isomerism) observed in NMR experiments. ^{1}H-NMR (DMSO-d$_6$), δ: 11.77 (s, 1H); 9.62 (bs, 2H); 9.11 (s, 1H); 8.84 (d, J = 8.5 Hz, 1H); 8.04-8.02 (m, 2H); 7.93 (d, J = 7.1 Hz, 1H); 7.68 (t, J = 7.5 Hz, 1H); 7.63-7.60 (m, 2H); 6.81 (s, 2H), 6.46 (s, 1H). ^{13}C-NMR (DMSO-d$_6$), δ: 163.6, 158.9, 147.7, 135.9, 134.0, 130.9, 130.7, 130.1, 129.2, 128.0, 127.7, 126.7, 126.0, 124.6, 106.2, 110.1. UPLC-MS: t_R 1.58 min, MS [ESI, m/z]: 307.1 [M+H$^+$]. HRMS calculated for $C_{18}H_{15}N_2O_3^+$: 307.1077; found 307.1071.

- 3-Methyl-*N*'-(naphthalen-1-ylmethylene)-[1,1'-biphenyl]-4-carbohydrazide **1ab**

Purified by recrystallisation from DCM. Obtained as a white solid in 74% yield. Two species (E/Z isomerism) observed in NMR experiments, in a ratio 0.7:0.3. ^{1}H-NMR (DMSO-d$_6$), δ: 11.95 (s, 1H); 9.03 (s, 0.7H); 8.95 (d, J = 8.5 Hz, 0.7H); 8.67 (s, 0.3H); 8.45 (d, J = 8.5 Hz, 0.3H); 8.13 – 8.04 (m, 1.3H); 7.97 (t, J = 8.8 Hz, 1.3H); 7.83-7.76 (m, 7.2H); 7.59-7.46 (m, 4H); 7.26 (t, J = 7.7 Hz, 0.3H); 2.58 (s, 2H); 2.44 (s, 1H). ^{13}C-NMR (DMSO-d$_6$), δ: 171.98, 165.44, 164.82, 144.91, 142.22, 141.11, 139.85, 137.43, 135.53, 134.49, 134.05, 131.09, 130.85, 130.64, 129.96, 129.49, 129.29, 128.74, 128.47, 128.40, 128.23, 127.86, 127.31, 127.23, 126.79, 126.60, 126.06, 125.90, 125.20, 124.77, 124.39, 124.05, 48.8, 48.6. UPLC-MS: t_R 2.06 min, MS [ESI, m/z]: 365.2 [M+H$^+$]. HRMS calculated for $C_{25}H_{21}N_2O^+$: 365.1648; found 365.1652.

- 2,4-Dihydroxy-*N*'-(1-(naphthalen-1-yl)ethylidene)benzohydrazide **5a**

Purified by recrystallisation from EtOH. Obtained as a white solid in 79% yield. Melting point: 198-201 °C. Two species (E/Z isomerism) observed in NMR experiments, in a ratio 1:0.6. ^{1}H-NMR (DMSO-d$_6$), δ: 8.25 (s, 0.6H), 8.10-8.04 (m, 2H), 8.00-7.95 (m, 1.3H), 7.89-7.84 (m, 0.6H), 7.70-7.63 (m, 2.1H), 7.61-7.53 (m, 5.7H), 7.51-7.46 (m, 1H), 6.44-6.41 (m, 1.2H), 6.24 (s, 1H), 6.06 (s, 1H), 5.75 (s, 0.4H), 2.45 (s, 2.4H), 2.38 (s, 3H). ^{13}C-NMR (DMSO-d$_6$), δ: 164.7, 162.1, 160.1, 147.1, 133.4, 131.4, 130.4, 128.6, 127.7, 126.5, 126.4, 125.9, 124.7, 115.7, 114.1, 101.1, 13.3. UPLC-MS: t_R 1.90 min, MS [ESI, m/z]: 321.1 [M+H$^+$]. HRMS calculated for $C_{19}H_{17}N_2O_3^+$: 321.1234; found 321.1237.

- 2,4-Dihydroxy-*N*'-(1-phenylethylidene)benzohydrazide **6a**

Purified by recrystallisation from EtOH/H_2O. Obtained as a white solid in 42% yield. One single species (E/Z isomerism) observed in NMR experiments. ^{1}H-NMR (DMSO-d$_6$), δ: 11.87 (s, 1H), 11.18 (s, 1H), 10.17 (s, 1H), 7.92-7.87 (m, 3H), 7.52-7.47 (m, 3H), 6.47-6.44 (m, 2H), 2.38 (s, 3H). ^{13}C-NMR (DMSO-d$_6$), δ: 162.9, 162.5, 159.2, 151.8, 138.5, 132.5, 129.7, 128.8, 126.8, 109.5, 108.4, 103.1, 49.1. UPLC-MS: t_R 1.51 min, MS [ESI, m/z]: 271.1 [M+H$^+$]. HRMS calculated for $C_{15}H_{15}N_2O_3^+$: 271.1077; found 271.1070.

- *N*'-Benzylidene-2,4-dihydroxybenzohydrazide **7a**

Purified by recrystallisation from EtOH/DCM. Obtained as a white solid in 46% yield. One single species (E/Z isomerism) observed in NMR experiments. ^{1}H-NMR (DMSO-d$_6$), δ: 12.34 (s, 1H), 11.70 (s, 1H), 10.23 (s, 1H), 8.44 (s, 1H), 7.81 (d, J=8.79 Hz, 1H), 7.77-7.70 (m, 2H) 7.50-7.41 (m, 3H), 6.37 (dd, J=8.7 Hz, J=2.3 Hz, 1H), 6.33-6.31 (m, 1H). ^{13}C-NMR (DMSO-d$_6$), δ: 165.9, 163.1, 162.7, 148.4, 134.7, 130.6, 130.2, 129.4, 127.6, 107.9, 106.7, 103.3. UPLC-MS: t_R 1.69 min, MS [ESI, m/z]: 257.1 [M+H$^+$]. HRMS calculated for $C_{14}H_{13}N_2O_3^+$: 257.0921; found 257.0927.

- *N*'-((1*H*-Indol-3-yl)methylene)-2,4-dihydroxybenzohydrazide **8a**

Purified by recrystallisation from EtOH. Obtained as a white solid in 67% yield. Melting point: 269-275 °C. One single species (E/Z isomerism) observed in NMR experiments. ^{1}H-NMR (DMSO-d$_6$), δ: 12.76 (s, 1H), 11.62 (s, 1H), 11.46 (s, 1H), 10.17 (s, 1H), 8.62 (s, 1H), 8.30 (d, J= 8.0 Hz, 1H), 7.85-7.83 (m 2H), 7.45 (d, J= 8.0 Hz, 1H), 7.23-7.15 (m, 2H), 6.37 (d, J= 8.0 Hz, 1H), 6.32 (s, 1H). ^{13}C-NMR (DMSO-d$_6$), δ: 170.6, 167.7, 166.0, 162.4, 154.3, 131.7, 129.9, 129.7, 128.1, 126.9, 121.0, 115.7. UPLC-MS: t_R 1.61 min, MS [ESI, m/z]: 296.1 [M+H$^+$]. HRMS calculated for $C_{16}H_{14}N_3O_3^+$: 296.1030; found 296.1034.

- *N*'-((1*H*-Indol-2-yl)methylene)-2,4-dihydroxybenzohydrazide **9a**[6]

Purified by recrystallisation from EtOH. Obtained as a white solid in 53% yield. Melting point: 269-271 °C. One single species (E/Z isomerism) observed in NMR experiments. ^{1}H-NMR (DMSO-d$_6$), δ: 12.44 (s, 1H), 11.69 (s, 1H), 10.24 (s, 1H), 8.49 (s, 1H), 7.83 (d, J= 8.5 Hz, 1H), 7.57 (d, J= 7.6 Hz, 1H), 7.46 (d, J= 8.5 Hz, 1H), 7.17 (app t, J= 7.6 Hz, 2H), 7.02 (app t, J= 8.7 Hz, 1H), 6.86 (s, 1H), 6.40 (d, J= 8.5 Hz, 1H), 6.34 (s, 1H). ^{13}C-NMR (DMSO-d$_6$), δ: 165.8, 163.2, 162.8, 141.3, 138.4, 133.5, 130.1, 128.1, 123.9, 121.2, 120.0, 112.5, 107.9, 107.6, 106.7, 103.3. UPLC-MS: t_R 1.67 min, MS [ESI, m/z]: 296.1 [M+H$^+$]. HRMS calculated for $C_{16}H_{14}N_3O_3^+$: 296.1030; found 296.1037.

- 2,4-Dihydroxy-*N*'-(quinolin-4-ylmethylene)benzohydrazide **10a**

Purified by recrystallisation from EtOH. Obtained as a white solid in 73% yield. Melting point: 223-225 °C. One single species (E/Z isomerism) observed in NMR experiments. ^{1}H-NMR (DMSO-d$_6$), δ: 12.13 (s, 1H), 11.99 (s, 1H), 10.13 (s, 1H), 9.08 (s, 1H), 9.00 (d, J= 6.0 Hz, 1H), 8.82 (d, J= 8.8 Hz, 1H), 8.11 (d, J= 8.8 Hz, 1H), 7.85-7.83 (m, 3H), 7.75 (app t, J= 6.0 Hz, 1H), 6.42 (d, J= 8.8 Hz, 1H), 6.37 (s, 1H). ^{13}C-NMR (DMSO-d$_6$), δ: 162.4, 150.1, 148.9, 137.8, 130.3, 128.1, 125.1, 120.6, 108.2, 103.3. UPLC-MS: t_R 1.61 min, MS [ESI, m/z]: 308.1 [M+H$^+$]. HRMS calculated for $C_{17}H_{14}N_3O_3^+$: 308.1030; found 308.1028.

- 2,4-Dihydroxy-*N'*-(isoquinolin-5-ylmethylene)benzohydrazide **11a**

Purified by recrystallisation from EtOH. Obtained as a white solid in 61% yield. Melting point: 268-270 °C. One single species (E/Z isomerism) observed in NMR experiments. ^{1}H-NMR (DMSO-d$_6$), δ: 12.29 (s, 1H), 11.85 (s, 1H), 10.27 (s, 1H), 9.40 (s, 1H), 8.99 (s, 1H), 8.84 (d, J= 5.8 Hz, 1H), 8.65 (d, J= 5.8 Hz, 1H), 8.22 (d, J= 7.9 Hz, 1H), 8.15 (d, J= 7.9 Hz, 1H), 7.85 (d, J= 8.7 Hz, 1H), 7.78 (app t, J= 7.9 Hz, 1H), 6.41 (d, J= 8.7 Hz, 1H), 6.36 (s, 1H). ^{13}C-NMR (DMSO-d$_6$), δ: 165.9, 163.3, 162.6, 153.7, 147.5, 144.8, 123.9, 130.5, 129.2, 127.7, 118.1, 108.0, 106.8, 103.4. UPLC-MS: t_R 1.29 min, MS [ESI, m/z]: 308.1 [M+H$^+$]. HRMS calculated for $C_{17}H_{14}N_3O_3^+$: 308.1030; found 308.1033.

- *N'*-((1*H*-Imidazol-2-yl)methylene)-2,4-dihydroxybenzohydrazide **12a**

Purified by recrystallisation from EtOH/H_2O. Obtained as a white solid in 39% yield. One single species (E/Z isomerism) observed in NMR experiments. ^{1}H-NMR (DMSO-d$_6$), δ: 12.89 (s, 1H), 12.30 (s, 1H), 11.69 (s, 1H), 10.26 (s, 1H), 8.35 (s, 1H), 7.79- 7.77 (d, J=8.6 Hz, 1H), 7.19 (s, 2H), 6.37 (dd, J_1= 8.9 Hz, J_2= 2.3 Hz, 1H), 6.32 (d, J= 2.3 Hz, 1H). ^{13}C-NMR (DMSO-d$_6$), δ: 165.8, 163.2, 162.7, 142.8, 140.3, 130.2, 128.8, 107.9, 107.5, 106.6, 103.3. UPLC-MS: t_R 1.28 min, MS [ESI, m/z]: 247.1 [M+H$^+$]. HRMS calculated for $C_{11}H_{11}N_4O_3^+$: 247.0826; found 247.0831.

- *N'*-(Furan-2-ylmethylene)-2,4-dihydroxybenzohydrazide **13a**

Purified by recrystallisation from EtOH/H_2O. Obtained as a pale yellow solid in 41% yield. One single species (E/Z isomerism) observed in NMR experiments. ^{1}H-NMR (DMSO-d$_6$), δ: 12.38 (s, 1H), 11.68 (s, 1H), 10.28 (s, 1H), 8.38 (s, 1H), 7.92-7.90 (m, 1H), 7.84-7.77 (d, J= 8.4 Hz, 1H), 7.00-6.98 (m, 1H), 6.71-6.69 (m, 1H), 6.42 (app q, J= 2.3 Hz, 1H), 6.36 (d, J= 2.3 Hz, 1H). ^{13}C-NMR (DMSO-d$_6$), δ: 165., 163.2, 162.7, 149.8, 145.8, 138.2, 130.0, 114.2, 112.7, 107.9, 106.6, 103.3. UPLC-MS: t_R 1.39 min, MS [ESI, m/z]: 247.1 [M+H$^+$]. HRMS calculated for $C_{12}H_{11}N_2O_4^+$: 247.0713; found 247.0710.

- 2,4-Dihydroxy-*N'*-(4-hydroxybenzylidene)benzohydrazide **14a**

Purified by recrystallisation from EtOH/DCM. Obtained as a pale yellow solid in 49% yield. One single species (E/Z isomerism) observed in NMR experiments. ^{1}H-NMR (DMSO-d$_6$), δ: 12.49 (s, 1H), 11.53 (s, 1H), 10.20 (s, 1H), 9.95 (s, 1H), 8.33 (s, 1H), 7.78 (d, J= 8.8 Hz, 1H), 7.56 (d, J= 8.5 Hz, 2H), 6.84 (m, 2H) 6.35 (dd, J_1= 8.7 Hz, J_2= 2.3 Hz, 1H), 6.30 (d, J= 2.3 Hz, 1H). ^{13}C-NMR (DMSO-d$_6$), δ: 165.8, 163.0, 162.9, 159.9, 148.8, 129.9, 129.4, 125.7, 116.2, 107.7, 106.6, 103.3. UPLC-MS: t_R 1.37 min, MS [ESI, m/z]: 273.1 [M+H$^+$]. HRMS calculated for $C_{14}H_{13}N_2O_4^+$: 273.0870; found 273.0864.

- 2,4-Dihydroxy-*N'*-(pyridin-4-ylmethylene)benzohydrazide **15a**

Purified by recrystallisation from EtOH. Obtained as an off-white solid in 37% yield. Melting point: 277-280 °C. One single species (E/Z isomerism) observed in NMR experiments. ^{1}H-NMR (DMSO-d$_6$), δ: 12.13 (s, 1H), 11.89 (s, 1H), 10.28 (s, 1H), 8.86 (d, J= 4.6 Hz, 2H), 8.42 (s, 1H), 7.81 (d, J= 8.6 Hz, 1H), 7.67 (d, J= 5.5 Hz, 2H), 6.38 (d, J= 8.6 Hz, 1H), 6.34 (s, 1H). ^{13}C-NMR (DMSO-d$_6$), δ: 165.9, 163.4, 162.4, 150.8, 145.8, 141.9, 130.6, 121.5, 108.1, 106.8, 103.3. UPLC-MS: t_R 1.21 min, MS [ESI, m/z]: 258.0 [M+H$^+$]. HRMS calculated for $C_{13}H_{12}N_3O_3^+$: 258.0873; found 258.0875.

- 2,4-Dihydroxy-*N'*-(pyridin-3-ylmethylene)benzohydrazide **16a**

Purified by recrystallisation from EtOH. Obtained as a white solid in 94% yield. Melting point: 289-290 °C. One single species (E/Z isomerism) observed in NMR experiments. ^{1}H-NMR (DMSO-d$_6$), δ: 12.24 (s, 1H), 11.82 (s, 1H), 10.26 (s, 1H), 8.86 (s, 1H), 8.62 (d, J= 7.1 Hz, 1H), 8.48 (s, 1H), 8.14 (d, J= 7.1 Hz, 1H), 7.81 (d, J= 8.7 Hz, 1H), 7.50 (q, J= 4.8, 7.9 Hz, 1H), 6.38 (d, J= 8.7 Hz, 1H), 6.33 (s, 1H). ^{13}C-NMR (DMSO-d$_6$), δ: 165.9, 163.3, 162.6, 151.2, 149.3, 145.6, 133.9, 124.5, 108.0, 103.3. UPLC-MS: t_R 1.06 min, MS [ESI, m/z]: 258.0 [M+H$^+$]. HRMS calculated for $C_{13}H_{12}N_3O_3^+$: 258.0873; found 258.0877.

- *N'*-(3,4-Difluorobenzylidene)-2,4-dihydroxybenzohydrazide **17a**

Purified by recrystallisation from EtOH. Obtained as a white solid in 74% yield. Melting point: 275-280 °C. One single species (E/Z isomerism) observed in NMR experiments. ^{1}H-NMR (DMSO-d$_6$), δ: 12.25 (s, 1H), 11.79 (s, 1H), 10.26 (s, 1H), 8.41 (s, 1H), 7.81-7.75 (m, 2H), 7.60 (bs, 1H), 7.57-7.52 (m, 1H), 6.38 (d, J= 8.8 Hz, 1H), 6.33 (s, 1H). ^{13}C-NMR (DMSO-d$_6$), δ: 165.9, 163.3, 162.6, 151.3, 149.2, 146.1, 132.6, 130.4, 124.8, 118.7, 115.8, 108.0, 106.7, 103.3. ^{19}F-NMR (DMSO-d$_6$), δ: -136.05, -137.85. UPLC-MS: t_R 1.61 min, MS [ESI, m/z]: 293.0 [M+H$^+$]. HRMS calculated for $C_{14}H_{11}F_2N_2O_3^+$: 293.0732; found 293.0727.

- *N′*-(3,4-Dichlorobenzylidene)-2,4-dihydroxybenzohydrazide **18a**

Purified by recrystallisation from EtOH. Obtained as a white solid in 71% yield. Melting point: 275-276 °C. One single species (E/Z isomerism) observed in NMR experiments. ^{1}H-NMR (DMSO-d$_6$), δ: 12.22 (s, 1H), 11.84 (s, 1H), 10.27 (s, 1H), 8.41 (s, 1H), 7.96 (s, 1H), 7.81 (d, J= 8.7 Hz, 1H), 7.73 (s, 2H), 6.38 (d, J= 8.7 Hz, 1H), 6.33 (s, 1H). ^{13}C-NMR (DMSO-d$_6$), δ: 165.9, 163.3, 162.5, 145.6, 132.7, 132.2, 131.6, 130.5, 129.0, 127.4, 108.0, 106.8, 103.3. UPLC-MS: t_R 1.78 min, MS [ESI, m/z]: 325.1 [M+H$^+$]. HRMS calculated for $C_{14}H_{11}Cl_2N_2O_3^+$: 325.0141; found 325.0147.

- *N'*-(3,4-dichlorobenzylidene)-2,4-dimethylbenzohydrazide **18f**

Purified by recrystallisation from EtOH. Obtained as a white solid in 88% yield. Melting point: 194-195 °C. Two species (E/Z isomerism) observed in NMR experiments, in a ratio 1:0.2. ^{1}H-NMR (DMSO-d$_6$), δ: 11.88 (s, 1.2H), 8.28 (s, 1H), 8.03 (s, 0.2H), 7.95 (s, 1H), 7.72 (s, 2H), 7.62 (s, 0.8H), 7.36 (d, J= 7.5 Hz, 1.2H), 7.21 (d, J= 7.5 Hz, 0.2H), 7.14 (s, 1H), 7.11 (d, J= 7.5 Hz, 1H), 7.08 (s, 0.2H), 2.36 (s, 3H), 2.33 (s, 3.6H), 2.23 (s, 0.6H). ^{13}C-NMR (DMSO-d$_6$), δ: 165.8, 144.8, 140.3, 136.6, 135.7, 132.6, 132.5, 132.2, 131.8, 131.6, 128.9, 128.1, 127.3, 126.6, 21.3, 19.8. UPLC-MS: t_R 1.99 min, MS [ESI, m/z]: 321.1 [M+H$^+$]. HRMS calculated for $C_{16}H_{15}Cl_2N_2O^+$: 321.0556; found 321.0560.

- *N*'-(3,4-Dichlorobenzylidene)-2-hydroxy-4-methylbenzohydrazide **18u**

Purified by recrystallisation from EtOH. Obtained as a white solid in 79% yield. Melting point: 235-239 °C. One single species (E/Z isomerism) observed in NMR experiments. ^{1}H-NMR (DMSO-d$_6$), δ: 11.96 (s, 1H), 8.44 (s, 1H), 7.98 (s, 1H), 7.82 (d, J= 7.91 Hz, 1H), 7.75 (m, 3H), 6.81 (bs, 2H), 2.31 (s, 3H). ^{13}C-NMR (DMSO-d$_6$), δ: 165.5, 159.8, 146.2, 145.2, 135.5, 132.9, 132.2, 131.6, 129.1, 128.9, 127.5, 120.5, 118.0, 113.1, 21.6. UPLC-MS: t_R 1.94 min, MS [ESI, m/z]: 323.0 [M+H$^+$]. HRMS calculated for $C_{15}H_{13}Cl_2N_2O_2^+$: 323.0349; found 323.0352.

- *N*'-(3,4-Dichlorobenzylidene)-4-hydroxy-2-methylbenzohydrazide **18v**

Purified by recrystallisation from EtOH. Obtained as a white solid in 79% yield. Melting point: 235-239 °C. One single species (E/Z isomerism) observed in NMR experiments. ^{1}H-NMR (DMSO-d$_6$), δ: 11.76 (s, 1H), 9.83 (s, 1H), 8.27 (s, 1H), 7.94 (s, 1H), 7.71 (bs, 2H), 7.33 (s, 1H), 6.68 (bs, 2H), 2.34 (s, 3H). ^{13}C-NMR (DMSO-d$_6$), δ: 165.7, 159.5, 144.3, 139.3, 135.8, 132.1, 128.8, 118.0, 112.6, 20.3. UPLC-MS: t_R 1.70 min, MS [ESI, m/z]: 323.0 [M+H$^+$]. HRMS calculated for $C_{15}H_{13}Cl_2N_2O_2^+$: 323.0349; found 323.0342.

- *N*'-(3,4-Dichlorobenzylidene)-3,5-dihydroxybenzohydrazide **18w**

Purified by recrystallisation from DCM. Obtained as a white solid in 90% yield. One single species (E/Z isomerism) observed in NMR experiments. ^{1}H-NMR (DMSO-d$_6$), δ: 11.89 (s, 1H), 9.60 (bs, 2H), 8.40 (s, 1H), 7.94 (s, 1H), 7.72 (s, 2H), 6.75 (s, 2H), 6.44 (s, 1H). ^{13}C-NMR (DMSO-d$_6$), δ: 163.9, 158.8, 145.1, 135.7, 135.6, 132.5, 132.1, 131.5, 128.8, 127.3, 106.2. UPLC-MS: t_R 1.62 min, MS [ESI, m/z]: 325.1 [M+H$^+$]. HRMS calculated for $C_{14}H_{11}Cl_2N_2O_3^+$: 325.0141; found 325.0139.

- *N*'-(3,4-Dichlorobenzylidene)-3,4-dihydroxybenzohydrazide **18x**

Purified by recrystallisation from DCM. Obtained as a white solid in 85% yield. One single species (E/Z isomerism) observed in NMR experiments. ^{1}H-NMR (DMSO-d$_6$), δ: 11.79 (s, 1H), 9.64 (s, 1H), 9.29 (s, 1H), 8.39 (s, 1H), 7.93 (s, 1H), 7.71 (s, 2H), 7.37 (d, J = 1.9 Hz, 1H), 7.31 (dd, J_1= 8.2 Hz, J_2= 1.9 Hz, 1H), 6.83 (d, J= 8.2 Hz, 1H). ^{13}C-NMR (DMSO-d$_6$), δ: 163.4, 149.6, 145.5, 144.3, 135.9, 132.3, 132.1, 131.5, 128.7, 127.2, 124.5, 120.2, 115.8, 115.4. UPLC-MS: t_R 1.63 min, MS [ESI, m/z]: 325.1 [M+H$^+$]. HRMS calculated for $C_{14}H_{11}Cl_2N_2O_3^+$: 325.0141; found 325.0135.

- *N*'-(3,4-Dichlorobenzylidene)-2,6-dihydroxybenzohydrazide **18y**

Purified by recrystallisation from DCM. Obtained as a white solid in 58% yield. One single species (E/Z isomerism) observed in NMR experiments. ^{1}H-NMR (DMSO-d$_6$), δ: 12.04 (s, 2H), 11.92 (s, 1H), 8.46 (s, 1H), 8.01 (s, 1H), 7.80 (s, 2H), 7.27 (app t, J= 8.2 Hz, 1H), 6.49 (d, J= 8.2 Hz, 2H). ^{13}C-NMR (DMSO-d$_6$), δ: 166.5, 160.0, 146.9, 135.3, 134.1, 132.9, 132.1, 131.6, 129.1, 127.5, 107.6, 104.0. UPLC-MS: t_R 1.91 min, MS [ESI, m/z]: 325.0 [M+H$^+$]. HRMS calculated for $C_{14}H_{11}Cl_2N_2O_3^+$: 325.0141; found 325.0144.

- *N'*-(3,4-Dichlorobenzylidene)-3-methyl-[1,1'-biphenyl]-4-carbohydrazide **18ab**

Purified by recrystallisation from DCM. Obtained as a white solid in 86% yield. One single species (E/Z isomerism) observed in NMR experiments. ^{1}H-NMR (DMSO-d$_6$), δ: 12.06 (s, 1H), 8.35 (s, 1H), 8.02 (s, 1H), 7.78 (app d, J= 5.6 Hz, 2H), 7.76 (s, 1H), 7.69-7.60 (m, 3H), 7.55 (app t, J= 7.6 Hz, 2H), 7.46 (app t, J= 7.6 Hz, 2H), 2.52 (s, 3H). ^{13}C-NMR (DMSO-d$_6$), δ: 165.6, 145.1, 142.2, 139.8, 137.3, 135.6, 134.2, 132.7, 132.2, 131.5, 129.4, 129.0, 128.7, 128.4, 127.3, 127.2, 124.3, 20.0. UPLC-MS: t_R 2.10 min, MS [ESI, m/z]: 383.1 [M+H$^+$]. HRMS calculated for $C_{21}H_{17}Cl_2N_2O^+$: 383.0712; found 383.0709.

- *N*'-(4-Chlorobenzylidene)-2,4-dihydroxybenzohydrazide **19a**

Purified by recrystallisation from EtOH. Obtained as a white solid in 61% yield. Melting point: 253-257 °C. One single species (E/Z isomerism) observed in NMR experiments. ^{1}H-NMR (DMSO-d$_6$), δ: 12.28 (s, 1H), 11.74 (s, 1H), 10.25 (s, 1H), 8.43 (s, 1H), 7.80 (d, J= 8.6 Hz, 1H), 7.76 (d, J= 8.6 Hz, 2H), 7.54 (d, J= 8.6 Hz, 2H), 6.83 (d, J= 8.6 Hz, 1H), 6.33 (s, 1H). ^{13}C-NMR (DMSO-d$_6$), δ: 165.9, 163.2, 162.6, 147.1, 135.0, 133.7, 130.3, 129.4, 129.2, 108.0, 106.7, 103.3. UPLC-MS: t_R 1.65 min, MS [ESI, m/z]: 291.0 [M+H$^+$]. HRMS calculated for $C_{14}H_{12}ClN_2O_3^+$: 291.0531; found 291.0537.

- 2,4-Dihydroxy-*N*'-(4-(trifluoromethyl)benzylidene)benzohydrazide **20a**

Purified by recrystallisation from EtOH. Obtained as a white solid in 37% yield. Melting point: 266-270 °C. One single species (E/Z isomerism) observed in NMR experiments. ^{1}H-NMR (DMSO-d$_6$), δ: 12.21 (s, 1H), 11.85 (s, 1H), 10.28 (s, 1H), 8.51 (s, 1H), 7.95 (d, J= 7.8 Hz, 2H), 7.83 (m, 3H), 6.39 (d, J= 7.8 Hz, 1H), 6.34 (s, 1H). ^{13}C-NMR (DMSO-d$_6$), δ: 165.9, 163.3,

162.5, 146.5, 138.7, 130.5, 128.2, 126.2, 125.7, 123.5, 108.0, 106.8, 103.3. ^{19}F-NMR (DMSO-d$_6$), δ: -61.16. UPLC-MS: t$_R$ 1.73 min, MS [ESI, m/z]: 325.1 [M+H$^+$]. HRMS calculated for $C_{15}H_{12}F_3N_2O_3^+$: 325.0795; found 325.0797.

- 2,4-Dihydroxy-*N*'-(4-(pentafluoro-*λ*6-sulfaneyl)benzylidene)benzohydrazide **21a**

Purified by flash column chromatography eluting with *n*-hexane: ethyl acetate 40:60 v/v increasing to *n*-hexane: ethyl acetate 0:100 v/v in 10 CV. Obtained as a white solid in 43% yield. Melting point: 254-256 °C. One single species (E/Z isomerism) observed in NMR experiments. ^{1}H-NMR (DMSO-d$_6$), δ: 12.17 (s, 1H), 11.88 (s, 1H), 10.29 (s, 1H), 8.50 (s, 1H), 7.98 (app q, J= 8.7 Hz, 4H), 7.82 (d, J= 8.7 Hz, 1H), 6.39 (d, J= 8.7 Hz, 1H), 6.35 (s, 1H). ^{13}C-NMR (DMSO-d$_6$), δ: 164.7, 162.1, 160.1, 157.7, 039.5, 133.8, 131.4, 127.8, 127.3, 115.7, 114.1, 101.1. ^{19}F-NMR (DMSO-d$_6$), δ: 87.1 (quintet, J= 150.5 Hz, 1F), 64.1 (d, J= 150.5 Hz, 4F). UPLC-MS: t$_R$ 1.80 min, MS [ESI, m/z]: 383.2 [M+H$^+$]. HRMS calculated for $C_{14}H_{12}F_5N_2O_3S^+$: 383.0483; found 383.0479.

- *N*'-(4-(*tert*-Butyl)benzylidene)-2,4-dihydroxybenzohydrazide **22a**

Purified by recrystallisation from EtOH. Obtained as a white solid in 84% yield. Melting point: 225-229 °C. One single species (E/Z isomerism) observed in NMR experiments. ^{1}H-NMR (DMSO-d$_6$), δ: 12.39 (s, 1H), 11.66 (s, 1H), 10.24 (s, 1H), 8.34 (s, 1H), 7.84-7.81 (m, 1H), 7.66 (bs, 2H), 7.50 (bs, 2H), 6.37 (d, J= 7.2 Hz, 1H), 6.32 (s, 1H), 1.31 (s, 9H). ^{13}C-NMR (DMSO-d$_6$), δ: 165.9, 163.1, 162.7, 156.4, 148.5, 132.0, 130.1, 127.5, 126.1, 107.9, 106.7, 103.3, 35.1, 31.4. UPLC-MS: t$_R$ 1.85 min, MS [ESI, m/z]: 313.1 [M+H$^+$]. HRMS calculated for $C_{18}H_{21}N_2O_3^+$: 313.1547; found 313.1550.

- *N*'-(4-(*tert*-Butyl)benzylidene)-2,4-dimethylbenzohydrazide **22f**

Purified by recrystallisation from DCM. Obtained as a white solid in 65% yield. Melting point: 212-216 °C. Two species (E/Z isomerism) observed in NMR experiments, in a ratio 1:0.3. ^{1}H-NMR (DMSO-d$_6$), δ: 11.64 (s, 0.3H), 11.60 (s, 1H), 8.28 (s, 1H), 7.64 (d, J= 8.4 Hz, 2H), 7.54 (d, J= 8.4 Hz, 0.3H) 7.49 (d, J= 8.2 Hz, 2H), 7.39-7.32 (m, 2.2H), 7.19 (d, J= 7.5 Hz, 0.3H), 7.13-7.10 (m, 2.3H), 7.06 (d, J= 7.5 Hz, 0.3H), 2.36 (s, 3H), 2.33 (s, 3.9H), 2.23 (s, 3.9H), 1.31 (s, 9H), 1.25 (s, 2.7H). ^{13}C-NMR (DMSO-d$_6$), δ: 163.2, 153.6, 146.8, 138.0, 137.1, 132.4, 130.7, 128.8, 126.7, 125.1, 34.2, 32.3, 21.6, 19.3. UPLC-MS: t$_R$ 2.06 min, MS [ESI, m/z]: 309.1 [M+H$^+$]. HRMS calculated for $C_{20}H_{25}N_2O^+$: 309.1961; found 309.1955.

- *N*'-(3-Chloro-5-(trifluoromethyl)benzylidene)-2,4-dihydroxybenzohydrazide **23a**

Purified by recrystallisation from EtOH. Obtained as a white solid in 94% yield. Melting point: 275-276 °C. One single species (E/Z isomerism) observed in NMR experiments. ^{1}H-NMR (DMSO-d$_6$), δ: 12.15 (s, 1H), 11.95 (s, 1H), 10.29 (s, 1H), 8.48 (s, 1H), 8.08 (bs, 1H), 8.04 (bs, 1H), 7.92-7.89 (m, 1H), 7.82 (d, J= 8.8 Hz, 1H), 6.39 (d, J= 8.8 Hz, 1H), 6.34 (s, 1H). ^{13}C-NMR (DMSO-d$_6$), δ: 165.9, 163.4, 162.4, 144.9, 138.2, 135.2, 131.9, 131.7, 130.9, 126.5, 124.7, 122.3, 108.1 ,106.8, 103.3. ^{19}F-NMR (DMSO-d$_6$), δ: -61.44. UPLC-MS: t$_R$ 1.87 min, MS [ESI, m/z]: 359.1 [M+H$^+$]. HRMS calculated for $C_{15}H_{11}ClF_3N_2O_3^+$: 359.0405; found 359.0409.

- 2,4-Dihydroxy-*N*'-(3-methoxy-5-(trifluoromethyl)benzylidene)benzohydrazide **24a**

Purified by recrystallisation from EtOH. Obtained as a white solid in 77% yield. Melting point: 264-268 °C. One single species (E/Z isomerism) observed in NMR experiments. ^{1}H-NMR (DMSO-d$_6$), δ: 12.91 (s, 1H), 11.86 (s, 1H), 10.27 (s, 1H), 8.47 (s, 1H), 7.82 (d, J= 8.7 Hz, 1H), 7.65 (s, 1H), 7.55 (s, 1H), 7.31 (s, 1H), 6.39 (d, J= 8.7 Hz, 1H), 6.34 (s, 1H), 3.90 (s, 3H). ^{13}C-NMR (DMSO-d$_6$), δ: 165.9, 163.3, 162.4, 160.5, 146.4, 137.5, 130.5, 125.3, 123.2, 116.6, 115.8, 112.4, 108.0, 106.8, 103.3, 56.3. ^{19}F-NMR (DMSO-d$_6$), δ: -61.33. UPLC-MS: t$_R$ 1.77 min, MS [ESI, m/z]: 355.1 [M+H$^+$]. HRMS calculated for $C_{16}H_{14}F_3N_2O_4^+$: 355.0900; found 355.0897.

- *N*'-(2,4-Dichlorobenzylidene)-2,4-dihydroxybenzohydrazide **25a**

Purified by recrystallisation from EtOH. Obtained as an off-white solid in 75% yield. Melting point: 273-275 °C. One single species (E/Z isomerism) observed in NMR experiments. ^{1}H-NMR (DMSO-d$_6$), δ: 12.29 (s, 1H), 11.96 (s, 1H), 10.30 (s, 1H), 8.79 (s, 1H), 8.02 (d, J= 8.5 Hz, 1H), 7.81 (d, J= 8.8 Hz, 1H), 7.74 (s, 1H), 7.54 (d, J= 8.5 Hz, 1H), 6.49 (d, J= 8.8 Hz, 1H), 6.33 (s, 1H). ^{13}C-NMR (DMSO-d$_6$), δ: 166.3, 163.4, 163.1, 143.2, 135.6, 134.4, 131.1, 130.1, 129.99, 128.6, 128.5, 108.0, 106.4, 103.3. UPLC-MS: t$_R$ 1.81 min, MS [ESI, m/z]: 325.1 [M+H$^+$]. HRMS calculated for $C_{14}H_{11}Cl_2N_2O_3^+$: 325.0141; found 325.0144.

- *N*'-(4-Cyanobenzylidene)-2,4-dihydroxybenzohydrazide **26a**

Purified by recrystallisation from EtOH. Obtained as a pale yellow solid in 87% yield. Melting point: 274-276 °C. One single species (E/Z isomerism) observed in NMR experiments. ^{1}H-NMR (DMSO-d$_6$), δ: 12.17 (s, 1H), 11.88 (s, 1H), 10.28 (s, 1H), 8.49 (s, 1H), 7.91 (s, 4H), 7.81 (d, J= 8.8 Hz, 1H,), 6.39 (d, J= 8.8 Hz, 1H), 6.34 (s, 1H). ^{13}C-NMR (DMSO-d$_6$), δ: 165.7, 163.1, 162.9, 145.8, 137.5, 131.1, 129.6, 124.8, 123.1, 122.5, 120.9, 112.3, 107.6, 106.6, 103.4. UPLC-MS: t$_R$ 1.48 min, MS [ESI, m/z]: 282.0 [M+H$^+$]. HRMS calculated for $C_{15}H_{12}N_3O_3^+$: 282.0873; found 282.0867.

- *N*'-(3,4-Dimethylbenzylidene)-2,4-dihydroxybenzohydrazide **27a**

Purified by recrystallisation from EtOH. Obtained as a white solid in 43% yield. Melting point: 230-234 °C. One single species (E/Z isomerism) observed in NMR experiments. ^{1}H-NMR (DMSO-d$_6$), δ: 12.40 (s, 1H), 11.62 (s, 1H), 10.23 (s, 1H), 8.37 (s, 1H), 7.80 (d, J= 8.7 Hz, 1H), 7.52 (s, 1H), 7.44 (d, J= 7.8 Hz, 1H), 7.23 (d, J= 7.8 Hz, 1H), 6.37 (d, J= 8.7 Hz, 1H), 6.32 (s, 1H), 2.28 (s, 3H), 2.26 (s, 3H). ^{13}C-NMR (DMSO-d$_6$), δ: 165.9, 163.4, 163.3, 162.5, 146.3, 139.2, 133.2, 130.5, 128.1, 119.1, 112.4, 108.1, 106.8, 103.3, 20.8, 20.6. UPLC-MS: t$_R$ 1.70 min, MS [ESI, m/z]: 285.1 [M+H$^+$]. HRMS calculated for $C_{16}H_{17}N_2O_3^+$: 285.1234; found 285.1230.

- 2,4-Dihydroxy-*N*'-(4-methoxybenzylidene)benzohydrazide **28a**[7]

Purified by recrystallisation from EtOH. Obtained as a white solid in 85% yield. Melting point: 270-271 °C. One single species (E/Z isomerism) observed in NMR experiments. ^{1}H-NMR (DMSO-d$_6$), δ: 12.43 (s, 1H), 11.58 (s, 1H), 10.21 (s, 1H), 8.38 (s, 1H), 7.80 (d, J= 8.7 Hz, 1H), 7.68 (d, J= 8.7 Hz, 2H), 7.03 (d, J= 8.7 Hz, 2H), 6.36 (d, J= 8.7 Hz, 1H), 6.32 (s, 1H), 3.82 (s, 3H). ^{13}C-NMR (DMSO-d$_6$), δ: 165.8, 163.1, 162.8, 161.4, 148.4, 130.0, 129.2, 127.2, 114.8, 107.8, 106.6, 103.3, 55.8. UPLC-MS: t$_R$ 1.51 min, MS [ESI, m/z]: 287.1 [M+H$^+$]. HRMS calculated for $C_{15}H_{15}N_2O_4^+$: 287.1026; found 287.1021.

- *N*'-([1,1'-Biphenyl]-4-ylmethylene)-2,4-dihydroxybenzohydrazide **29a**

Purified by recrystallisation from EtOH/H_2O. Obtained as an off-white solid in 36% yield. One single species (E/Z isomerism) observed in NMR experiments. ^{1}H-NMR (DMSO-d$_6$), δ: 12.36 (s, 1H), 11.76 (s, 1H), 10.24 (s, 1H), 8.48 (s, 1H), 7.85-7.76 (m, 5H), 7.73 (d, J= 7.4 Hz, 2H), 7.52-7.47 (m, 2H), 7.42-7.38 (m, 1H), 6.38 (dd, J_1= 8.7 Hz, J_2= 2.3 Hz, 1H), 6.33 (d, J= 2.3 Hz, 1H). ^{13}C-NMR (DMSO-d$_6$), δ: 165.9, 163.2, 162.8, 147.9, 142.1, 139.8, 133.8, 130.2, 129.5, 128.4, 128.2, 127.6, 127.1, 112.7, 107.9, 106.8, 103.4. UPLC-MS: t$_R$ 2.12 min, MS [ESI, m/z]: 333.1 [M+H$^+$]. HRMS calculated for $C_{20}H_{17}N_2O_3^+$: 333.1234; found 333.1239.

- *N*'-(2,3-Difluorobenzylidene)-2,4-dihydroxybenzohydrazide **30a**

Purified by recrystallisation from EtOH. Obtained as a white solid in 57% yield. Melting point: 249-252 °C. One single species (E/Z isomerism) observed in NMR experiments. ^{1}H-NMR (DMSO-d$_6$), δ: 12.33 (s, 1H), 11.89 (s, 1H), 10.28 (s, 1H), 8.67 (s, 1H), 7.80 (d, J= 8.7 Hz, 1H), 7.73 (s, 1H), 7.52 (d, J= 8.7 Hz, 1H), 7.32 (d, J= 5.0 Hz, 1H), 6.39 (d, J= 8.7 Hz, 1H), 6.33 (s, 1H). ^{13}C-NMR (DMSO-d$_6$), δ: 162.9, 140.1, 130.2, 125.7, 124.7, 124.6, 122.1, 119.1, 118.9, 108.2, 106.5, 103.3. ^{19}F-NMR (DMSO-d$_6$), δ: -138.91, -146.34. UPLC-MS: t$_R$ 1.61 min, MS [ESI, m/z]: 293.0 [M+H$^+$]. HRMS calculated for $C_{14}H_{11}F_2N_2O_3^+$: 293.0732; found 293.0735.

- *N*'-(2,3-Difluorobenzylidene)-2,4-dimethylbenzohydrazide **30f**

Purified by recrystallisation from EtOH. Obtained as a white solid in 40% yield. Melting point: 203-205 °C. Two species (E/Z isomerism) observed in NMR experiments, in a ratio 1:0.3. ^{1}H-NMR (DMSO-d$_6$), δ: 11.90 (s, 1.3H), 8.54 (s, 1H), 8.26 (s, 0.3H), 7.74 (app t, J= 6.1 Hz, 1H), 7.51 (d, J= 8.5 Hz, 1H), 7.39 (d, J= 8.5 Hz, 1H), 7.32 (d, J= 6.1 Hz, 1H), 7.22-7.06 (m, 3.5H), 2.37 (s, 3H), 2.33 (s, 3.9H), 2.24 (s, 0.9H). ^{13}C-NMR (DMSO-d$_6$), δ: 165.7, 140.4, 139.2, 136.7, 131.9, 128.1, 126.6, 125.7, 122.0, 21.3, 19.9. ^{19}F-NMR (DMSO-d$_6$), δ: -138.91, -146.72. UPLC-MS: t$_R$ 1.85 min, MS [ESI, m/z]: 289.1 [M+H$^+$]. HRMS calculated for $C_{16}H_{15}F_2N_2O^+$: 289.1147; found 289.1151.

- *N*'-(2,3-Dichlorobenzylidene)-2,4-dihydroxybenzohydrazide **31a**

Purified by recrystallisation from DCM. Obtained as a white solid in 70% yield. Melting point: 242-243 °C. One single species (E/Z isomerism) observed in NMR experiments. ^{1}H-NMR (DMSO-d$_6$), δ: 12.27 (s, 1H), 11.99 (s, 1H), 10.30 (s, 1H), 8.87 (s, 1H), 7.99 (d, J= 8.8 Hz, 1H), 7.81 (d, J= 7.9 Hz, 1H), 7.73 (d, J= 7.9 Hz, 1H), 7.47 (app t, J= 7.9 Hz, 1H), 6.39 (d, J= 8.8 Hz, 1H), 6.33 (s, 1H). ^{13}C-NMR (DMSO-d$_6$), δ: 163.0, 144.0, 134.5, 132.9, 132.1, 131.5, 129.0, 126.0, 108.0, 103.3. UPLC-MS: t$_R$ 1.77 min, MS [ESI, m/z]: 325.0 [M+H$^+$]. HRMS calculated for $C_{14}H_{11}Cl_2N_2O_3^+$: 325.0141; found 325.0133.

- *N*'-(2,3-Dichlorobenzylidene)-2,4-dimethylbenzohydrazide **31f**

Purified by recrystallisation from EtOH. Obtained as a white solid in 37% yield. Melting point: 199-200 °C. Two species (E/Z isomerism) observed in NMR experiments, in a ratio 1:0.3. ^{1}H-NMR (DMSO-d$_6$), δ: 11.99 (s, 1.3H), 8.76 (s, 1H), 8.47 (s, 0.3H), 7.99 (d, J= 7.8 Hz, 1H), 7.72 (d, J= 7.8 Hz, 1H), 7.65 (s, 0.3H), 7.47 (app t, J= 7.8 Hz, 1H), 7.40 (d, J= 7.8 Hz, 1H), 7.33 (s, 0.3H), 7.23 (s, 0.3H), 7.14-7.06 (m, 2.7H), 2.37 (s, 3H), 2.33 (s, 3.9H), 2.24 (s, 0.9H). ^{13}C-NMR (DMSO-d$_6$), δ: 165.8, 143.3, 140.4, 136.8, 134.6, 132.8, 132.2, 132.0, 131.9, 129.0, 128.1, 126.6, 125.9, 21.2, 19.9. UPLC-MS: t$_R$ 1.90 min, MS [ESI, m/z]: 320.9 [M+H$^+$]. HRMS calculated for $C_{16}H_{15}Cl_2N_2O^+$: 321.0556; found 321.0549.

- 2,4-Dimethyl-*N*'-(naphthalen-2-ylmethylene)benzohydrazide **32f**

Purified by recrystallisation from EtOH. Obtained as a white solid in 55% yield. Melting point: 232-235 °C. Two species (E/Z isomerism) observed in NMR experiments, in a ratio 1:0.2. ^{1}H-NMR (DMSO-d$_6$), δ: 11.79 (s, 1.2H), 8.17 (s, 1H), 8.23 (s, 0.2H), 8.13 (s, 1H), 8.02-8.00 (m, 1H), 7.99 (s, 2H), 7.97-7.95 (m, 1.2H), 7.87 (s, 0.2H), 7.83 (d, J= 8.5 Hz, 0.2H), 7.58 (bs, 2H), 7.53 (s, 0.6H), 7.49 (s, 0.2H), 7.39 (d, J= 7.6 Hz, 1H), 7.25 (d, J= 7.6 Hz, 0.2H), 7.14-7.09 (m, 2.4H), 2.38 (s, 3H), 2.35 (s, 0.6H), 2.34 (s, 3H), 2.27 (s, 0.6H). ^{13}C-NMR (DMSO-d$_6$), δ: 165.8, 147.5, 140.0, 136.5, 134.2, 133.4, 132.9, 132.6, 131.8, 129.1,

129.0, 128.8, 128.2, 128.1, 127.6, 127.2, 126.6, 123.1, 21.3, 19.9. UPLC-MS: t_R 1.91 min, MS [ESI, m/z]: 303.0 [M+H$^+$]. HRMS calculated for $C_{20}H_{19}N_2O^+$: 303.1492; found 303.1296.

- *N'*-(4-Isopropylbenzylidene)-2,4-dimethylbenzohydrazide **33f**

Purified by flash column chromatography, eluting with *n*-hexane:ethyl acetate 100:0 v/v increasing to *n*-hexane:ethyl acetate 0:100 v/v in 10 CV. Obtained as a white solid in 91% yield. Melting point: 195-197 °C. Two species (E/Z isomerism) observed in NMR experiments, in a ratio 1:0.2. ^{1}H-NMR (DMSO-d$_6$), δ: 11.64 (s, 0.3H), 11.61 (s, 1H), 8.28 (s, 1H), 8.03 (s, 0.3H), 7.64 (d, J= 9.1 Hz, 2H), 7.36-7.31 (m, 3H), 7.24-7.19 (m, 0.9H), 7.13 (s, 1H), 7.11 (d, J= 8.1 Hz, 1.3H), 7.06 (d, J= 7.6 Hz, 0.3H), 2.98-2.89 (m, 1H), 2.88-2.81 (m, 0.3H), 2.36 (s, 3H), 2.33 (s, 3.9H), 2.23 (s, 0.9H). ^{13}C-NMR (DMSO-d$_6$), δ: 165.6, 151.1, 147.6, 140.0, 136.5, 132.9, 132.5, 131.8, 128.0, 127.6, 127.3, 126.6, 24.1, 21.3, 19.8. UPLC-MS: t_R 1.99 min, MS [ESI, m/z]: 295.0 [M+H$^+$]. HRMS calculated for $C_{19}H_{23}N_2O^+$: 295.1805; found 295.1808.

- *N'*-(4-Cyclopropylbenzylidene)-2,4-dihydroxybenzohydrazide **34a**

Purified by recrystallisation from DCM. Obtained as a white solid in 73% yield. One single species (E/Z isomerism) observed in NMR experiments. ^{1}H-NMR (DMSO-d$_6$), δ: 12.39 (s, 1H), 11.63 (s, 1H), 10.22 (s, 1H), 8.39 (s, 1H), 7.80 (d, J = 8.6 Hz, 1H), 7.61 (d, J = 7.9 Hz, 2H), 7.16 (d, J = 7.9 Hz, 2H), 6.37 (dd, J_1= 8.6 Hz, J_2= 2.1 Hz, 1H), 6.32 (d, J= 2.1 Hz, 1H), 1.99-1.94 (m, 1H), 1.09-0.99 (m, 2H), 0.75-0.75 (m, 2H). ^{13}C-NMR (DMSO-d$_6$), δ: 165.8, 163.1, 162.7, 148.5, 146.9, 131.7, 130.0, 127.6, 126.1, 107.8, 106.6, 103.3, 15.9, 10.4. UPLC-MS: t_R 1.72 min, MS [ESI, m/z]: 297.0 [M+H$^+$]. HRMS calculated for $C_{17}H_{17}N_2O_3^+$: 297.1234; found 297.1239.

- *N'*-(3-Chloro-4-(trifluoromethyl)benzylidene)-2,4-dihydroxybenzohydrazide **35a**

Purified by recrystallisation from DCM. Obtained as a white solid in 81% yield. One single species (E/Z isomerism) observed in NMR experiments. ^{1}H-NMR (DMSO-d$_6$), δ: 12.14 (s, 1H), 11.95 (s, 1H), 10.29 (s, 1H), 8.47 (s, 1H), 8.03 (s, 1H), 7.95 (d, J= 8.3 Hz, 1H), 7.90 (d, J= 8.3 Hz, 1H), 7.82 (d, J= 8.8 Hz, 1H), 6.39 (dd, J_1= 8.8 Hz, J_2= 2.2 Hz, 1H), 6.34 (d, J= 2.2 Hz, 1H). ^{13}C-NMR (DMSO-d$_6$), δ: 165.9, 163.4, 162.4, 144.8, 140.6, 131.7, 130.6, 129.7, 128.9, 127.5, 126.3, 122.1, 108.0, 106.8, 103.3. ^{19}F-NMR (DMSO-d$_6$), δ: -61.08. UPLC-MS: t_R 1.83 min, MS [ESI, m/z]: 359.1 [M+H$^+$]. HRMS calculated for $C_{15}H_{11}ClF_3N_2O_3^+$: 359.0405; found 359.0403.

- 2,4-Dihydroxy-*N'*-(3-methyl-4-(trifluoromethyl)benzylidene)benzohydrazide **36a**

Purified by recrystallisation from DCM. Obtained as a white solid in 97% yield. One single species (E/Z isomerism) observed in NMR experiments. ^{1}H-NMR (DMSO-d$_6$), δ: 12.23 (s, 1H), 11.84 (s, 1H), 10.27 (s, 1H), 8.46 (s, 1H), 7.83-7.72 (m, 4H), 6.39 (dd, J_1= 8.7 Hz, J_2= 1.7 Hz, 1H), 6.34 (d, J= 1.6 Hz, 1H), 2.51 (s, 3H). ^{13}C-NMR (DMSO-d$_6$), δ: 165.9, 163.3, 162.2, 146.5, 138.4, 130.7, 130.4, 128.7, 128.5, 126.7, 125.9, 123.8, 108.0, 106.7, 103.3, 19.2. ^{19}F-NMR (DMSO-d$_6$), δ: -119.16. UPLC-MS: t_R 1.80 min, MS [ESI, m/z]: 339.1 [M+H$^+$]. HRMS calculated for $C_{16}H_{14}F_3N_2O_3^+$: 339.0951; found 339.0948.

- *N'*-(4-Cyclohexylbenzylidene)-2,4-dihydroxybenzohydrazide **37a**

Purified by recrystallisation from DCM. Obtained as a white solid in 89% yield. One single species (E/Z isomerism) observed in NMR experiments. ^{1}H-NMR (DMSO-d$_6$), δ: 12.40 (s, 1H), 11.64 (s, 1H), 10.22 (s, 1H), 8.41 (s, 1H), 7.81 (d, J= 8.7 Hz, 1H), 7.65 (d, J= 8.2 Hz, 2H), 7.32 (d, J= 8.2 Hz, 2H), 6.37 (dd, J_1= 8.7 Hz, J_2= 2.3 Hz, 1H), 6.32 (d, J= 2.3 Hz, 1H), 2.54 (t, J= 11.4 Hz, 1H), 1.80 (d, J= 11.4 Hz, 4H), 1.71 (d, J= 11.4 Hz, 1H), 1.46-1.34 (m, 4H), 1.29-1.22 (m, 1H). ^{13}C-NMR (DMSO-d$_6$), δ: 165.8, 163.1, 162.7, 150.4, 148.5, 132.3, 130.0, 127.6, 127.6, 107.8, 106.6, 103.3, 44.1, 34.2, 26.7, 26.0. UPLC-MS: t_R 1.98 min, MS [ESI, m/z]: 339.2 [M+H$^+$]. HRMS calculated for $C_{20}H_{23}N_2O_3^+$: 339.1703; found 339.1709.

- *N'*-(4-Cyclopentylbenzylidene)-2,4-dihydroxybenzohydrazide **38a**

Purified by recrystallisation from DCM. Obtained as a white solid in 78% yield. One single species (E/Z isomerism) observed in NMR experiments. ^{1}H-NMR (DMSO-d$_6$), δ: 12.39 (s, 1H), 11.64 (s, 1H), 10.22 (s, 1H), 8.41 (s, 1H), 7.81 (d, J= 8.6 Hz, 1H), 7.65 (d, J= 8.0 Hz, 2H), 7.34 (d, J= 8.0 Hz, 2H), 6.37 (dd, J_1= 8.6 Hz, J_2= 2.2 Hz, 1H), 6.32 (d, J= 2.2 Hz, 1H), 3.05-2.98 (m, 1H), 2.06-2.01 (m, 2H), 1.81-1.75 (m, 2H), 1.70-1.62 (m, 2H), 1.59-1.51 (m, 2H). ^{13}C-NMR (DMSO-d$_6$), δ: 165.8, 163.1, 162.7, 148.9, 148.5, 132.2, 130.0, 127.9, 127.6, 107.8, 106.6, 103.3, 45.7, 34.6, 25.5. UPLC-MS: t_R 1.90 min, MS [ESI, m/z]: 325.2 [M+H$^+$]. HRMS calculated for $C_{19}H_{21}N_2O_3^+$: 325.1547; found 325.1550.

- *N'*-(4-Cyclobutylbenzylidene)-2,4-dihydroxybenzohydrazide **39a**

Purified by recrystallisation from DCM. Obtained as a white solid in 76% yield. One single species (E/Z isomerism) observed in NMR experiments. ^{1}H-NMR (DMSO-d$_6$), δ: 12.39 (s, 1H), 11.65 (s, 1H), 10.23 (s, 1H), 8.41 (s, 1H), 7.81 (d, J= 8.7 Hz, 1H), 7.67 (d, J= 8.0 Hz, 2H), 7.32 (d, J= 8.0 Hz, 2H), 6.38 (dd, J_1= 8.7 Hz, J_2= 2.1 Hz, 1H), 6.32 (d, J= 2.1 Hz, 1H), 3.56 (quintet, J= 8.7 Hz, 1H), 2.35-2.25 (m, 2H), 2.18-2.05 (m, 2H), 1.85-1.80 (m, 2H). ^{13}C-NMR (DMSO-d$_6$), δ: 165.8, 163.1, 162.7, 148.4, 132.3, 130.1, 127.6, 127.2, 107.8, 106.6, 103.3, 41.4, 29.7, 18.2. UPLC-MS: t_R 1.85 min, MS [ESI, m/z]: 311.1 [M+H$^+$]. HRMS calculated for $C_{18}H_{19}N_2O_3^+$: 311.1390; found 311.1394.

- 2,4-Dihydroxy-*N*'-((2-hydroxynaphthalen-1-yl)methylene)benzohydrazide **40a**

Purified by recrystallisation from EtOH. Obtained as a light pink solid in 87% yield. Melting point: 280-284 °C. One single species (E/Z isomerism) observed in NMR experiments. ^{1}H-NMR (DMSO-d_6), δ: 12.77 (s, 1H), 12.12 (s, 1H), 11.97 (s, 1H), 10.30 (s, 1H), 9.52 (s, 1H), 8.30 (d, J= 8.8 Hz, 1H), 7.95-7.90 (m, 2H), 7.83 (d, J= 8.8 Hz, 1H), 7.62 (d, J= 7.5 Hz, 1H), 7.42 (d, J= 7.5 Hz, 1H), 7.24 (d, J= 8.8 Hz, 1H), 6.43 (d, J= 8.8 Hz, 1H), 6.38 (s, 1H). ^{13}C-NMR (DMSO-d_6), δ: 165.0, 163.4, 162.3, 158.5, 147.4, 133.2, 132.1, 130.4, 129.4, 128.4, 128.2, 124.0, 121.4, 119.4, 109.1, 108.2, 106.6, 103.3. UPLC-MS: t_R 1.80 min, MS [ESI, m/z]: 323.1 [M+H$^+$]. HRMS calculated for $C_{18}H_{15}N_2O_4^+$: 323.1026; found 323.1023.

- 2,4-Dihydroxy-*N*'-((4-hydroxynaphthalen-1-yl)methylene)benzohydrazide **41a**[3]

Purified by recrystallisation from EtOH. Obtained as a yellow solid in 74% yield. Melting point: 264-267 °C. One single species (E/Z isomerism) observed in NMR experiments. ^{1}H-NMR (DMSO-d_6), δ: 12.54 (s, 1H), 11.60 (s, 1H), 10.82 (s, 1H), 10.22 (s, 1H), 9.01 (d, J= 8.5 Hz, 1H), 8.91 (s, 1H), 8.25 (d, J= 8.5 Hz, 1H), 7.84 (d, J= 8.7 Hz, 1H), 7.75 (d, J= 8.0 Hz, 1H), 7.66 (d, J= 7.4 Hz, 1H), 7.55 (d, J= 7.4 Hz, 1H), 6.98 (d, J= 8.0 Hz, 1H), 6.39 (d, J= 8.7 Hz, 1H), 6.34 (s, 1H). ^{13}C-NMR (DMSO-d_6), δ: 165.8, 162.9, 156.3, 149.6, 132.1, 131.1, 128.2, 125.6, 125.2, 125.1, 123.1, 120.8, 108.5, 107.8, 103.4. UPLC-MS: t_R 1.56 min, MS [ESI, m/z]: 323.1 [M+H$^+$]. HRMS calculated for $C_{18}H_{15}N_2O_4^+$: 323.1026; found 323.1028.

- 2,4-Dihydroxy-*N*'-((1-hydroxynaphthalen-2-yl)methylene)benzohydrazide **42a**

Purified by recrystallisation from EtOH. Obtained as a white solid in 45% yield. Melting point: 223-226 °C. One single species (E/Z isomerism) observed in NMR experiments. ^{1}H-NMR (DMSO-d_6), δ: 12.89 (s, 1H), 12.16 (s, 1H), 12.08 (s, 1H), 10.31 (s, 1H), 8.75 (s, 1H), 8.31 (d, J= 8.0 Hz, 1H), 7.89 (d, J= 8.0 Hz, 1H), 7.83 (d, J= 6.9 Hz, 1H), 7.62-7.53 (m, 3H), 7.46 (s, 1H), 6.42 (d, J= 8.7 Hz, 1H), 6.37 (s, 1H). ^{13}C-NMR (DMSO-d_6), δ: 165.3, 163.4, 162.5, 155.4, 150.5, 135.0, 130.4, 128.5, 128.0, 127.2, 126.2, 124.8, 123.1, 119.3, 111.9, 108.2, 106.4, 103.4. UPLC-MS: t_R 1.85 min, MS [ESI, m/z]: 323.1 [M+H$^+$]. HRMS calculated for $C_{18}H_{15}N_2O_4^+$: 323.1026; found 323.1033.

- 2,4-Dihydroxy-*N*'-((8-hydroxynaphthalen-1-yl)methylene)benzohydrazide **43a**

Purified by recrystallisation from DCM. Obtained as a yellow solid in 85% yield. One single species (E/Z isomerism) observed in NMR experiments. ^{1}H-NMR (DMSO-d_6), δ: 12.57 (s, 1H), 11.86 (s, 1H), 10.60 (s, 1H), 10.23 (s, 1H), 6.67 (s, 1H), 7.93 (d, J= 8.1 Hz, 2H), 7.89 (d, J= 8.6 Hz, 1H), 7.52 (app t, J= 7.7 Hz, 1H), 7.43 (d, J= 8.1 Hz, 1H), 7.36 (app t, J= 7.7 Hz, 1H), 7.00 (d, J= 7.4 Hz, 1H), 6.38 (d, J= 8.6 Hz, 1H), 6.33 (s, 1H). ^{13}C-NMR (DMSO-d_6), δ: 166.3, 163.1, 163.1, 154.8, 152.0, 136.1, 131.2, 130.8, 130.0, 127.0, 126.2, 125.9, 122.4, 120.1, 111.2, 107.7, 106.4, 103.3. UPLC-MS: t_R 1.64 min, MS [ESI, m/z]: 323.1 [M+H$^+$]. HRMS calculated for $C_{18}H_{15}N_2O_4^+$: 323.1026; found 323.1019.

- 2,4-Dihydroxy-*N*'-((5-hydroxynaphthalen-1-yl)methylene)benzohydrazide **44a**

Purified by recrystallisation from DCM. Obtained as a light brown solid in 93% yield. One single species (E/Z isomerism) observed in NMR experiments. ^{1}H-NMR (DMSO-d_6), δ: 12.40 (s, 1H), 11.77 (s, 1H), 10.32 (s, 1H), 10.26 (s, 1H), 6.67 (s, 1H), 9.09 (s, 1H), 8.29 (d, J= 8.3 Hz, 1H), 8.22 (d, J= 8.3 Hz, 1H), 7.94 (d, J= 7.3 Hz, 1H), 7.85 (d, J= 8.3 Hz, 1H), 7.54 (app t, J= 7.9 Hz, 1H), 7.46 (t, J= 7.9 Hz, 1H), 6.97 (d, J= 7.3 Hz, 1H), 6.35 (s, 1H). ^{13}C-NMR (DMSO-d_6), δ: 165.9, 136.2, 162.8, 154.2, 148.3, 132.2, 130.0, 129.5, 128.3, 127.9, 125.5, 125.0, 124.5, 115.0, 108.9, 107.9, 106.6, 103.3. UPLC-MS: t_R 1.52 min, MS [ESI, m/z]: 323.1 [M+H$^+$]. HRMS calculated for $C_{18}H_{15}N_2O_4^+$: 323.1026; found 323.1034.

- *N*'-((5-Hydroxynaphthalen-1-yl)methylene)-2,4-dimethylbenzohydrazide **44f**

Purified by recrystallisation from DCM. Obtained as a yellow solid in 37% yield. Two species (E/Z isomerism) observed in NMR experiments, in a ratio 0.7:0.3. ^{1}H-NMR (DMSO-d_6), δ: 11.76 (s, 1H), 10.35 (s, 1H), 9.02 (s, 0.7H), 8.67 (s, 0.3H), 8.33 (d, J= 8.5 Hz, 0.7H), 8.20 (d, J= 8.5 Hz, 1H), 7.97 (d, J= 7.1 Hz, 1H), 7.80 (d, J= 8.5 Hz, 0.3H), 7.64-7.54 (m, 1.3H), 7.53-7.45 (m, 1.7H), 7.30 (d, J= 7.5 Hz, 0.3H), 7.20-7.15 (m, 2.3H), 7.01 (d, J= 7.5 Hz, 0.7H), 6.93 (d, J= 7.5 Hz, 0.3H), 2.45 (s, 2.1H), 2.42 (s, 0.9H), 2.39 (s, 2.1H), 2.32 (s, 0.9H). ^{13}C-NMR (DMSO-d_6), δ: 165.5, 154.1, 147.4, 140.1, 136.6, 132.7, 132.1, 131.8, 129.6, 128.3, 128.0, 127.7, 126.5, 125.5, 124.9, 124.5, 114.8, 108.9, 21.3, 19.9. UPLC-MS: t_R 1.73 min, MS [ESI, m/z]: 319.1 [M+H$^+$]. HRMS calculated for $C_{20}H_{19}N_2O_2^+$: 319.1441; found 319.1439.

- *N*'-(2,3-Dimethylbenzylidene)-2,4-dimethylbenzohydrazide **45f**

Purified by recrystallisation from EtOH. Obtained as a white solid in 39% yield. Melting point: 197-199 °C. Two species (E/Z isomerism) observed in NMR experiments, in a ratio 1:0.3. ^{1}H-NMR (DMSO-d_6), δ: : 11.59 (s, 1.3H), 8.70 (s, 1H), 8.42 (s, 0.3H), 7.71 (d, J=7.7 Hz, 1H), 7.38 (d, J= 7.7 Hz, 1H), 7.23 (d, J= 7.7 Hz, 1H), 7.20-7.01 (m, 5.3H), 2.37 (s, 3H), 2.33 (s, 3H), 2.32 (s, 0.9H), 2.30 (s, 3H), 2.28 (s, 3H), 2.24 (s, 0.9H), 2.23 (s, 0.9H), 2.19 (s, 0.9H). ^{13}C-NMR (DMSO-d_6), δ: 165.5, 146.8, 140.1, 137.6, 125.9, 132.7, 131.8, 131.6, 128.0, 126.5, 126.1, 124.2, 21.3, 20.5, 19.2, 15.6. UPLC-MS: t_R 1.62 min, MS [ESI, m/z]: 281.0 [M+H$^+$]. HRMS calculated for $C_{18}H_{21}N_2O^+$: 281.1648; found 281.1643.

- 3,4-Dichloro-*N*'-(2,4-dihydroxybenzylidene)benzohydrazide **46z**

Purified by recrystallisation from EtOH. Obtained as an off-white solid in 83% yield. One single species (E/Z isomerism) observed in NMR experiments. ^{1}H-NMR (DMSO-d$_6$), δ: : 12.02 (s, 1H), 11.30 (s, 1H), 9.99 (s, 1H), 8.51 (s, 1H), 8.17 (s, 1H), 7.91 (d, J= 8.4 Hz, 1H), 7.84 (d, J= 8.4 Hz, 1H), 7.35 (d, J= 8.4 Hz, 1H), 6.37 (d, J= 8.4 Hz, 1H), 6.33 (s, 1H). ^{13}C-NMR (DMSO-d$_6$), δ: 160.0, 150.0, 133.9, 131.7, 131.4, 129.9, 128.4, 111.0, 108.3, 103.1. UPLC-MS: t_R 1.74 min, MS [ESI, m/z]: 325.0 [M+H$^+$]. HRMS calculated for $C_{14}H_{11}Cl_2N_2O_3^+$: 325.0141; found 325.0137.

General procedure for the preparation of hydrazides 47 and 48

The appropriate hydrazone **1f** or **18a** (0.5 mmol, 1 eq.) was dissolved in TFA (20 mmol, 40 eq.) followed by triethylsilane (1 mmol, 2 eq.) at 0 °C. This solution was stirred at 0 °C until completion (monitored by TLC), before being diluted with 10% aq. HCl (15 mL). The precipitate formed was isolated by vacuum filtration, washed thoroughly with cold water and 40-60 petroleum ether, and then dried under *vacuum*. The solid product was purified by recrystallisation from DCM.

- 2,4-Dimethyl-N'-(naphthalen-1-ylmethyl)benzohydrazide **47**

Obtained as a white solid in 48% yield. Melting point: 192-194 °C. ^{1}H-NMR (DMSO-d$_6$), δ: 9.70 (s, 1H), 8.35 (d, J= 8.2 Hz, 1H), 7.95 (d, J= 7.9 Hz, 1H), 7.87 (d, J= 8.2 Hz, 1H), 7.59-7.52 (m, 3H), 7.48 (app t, J= 7.9 Hz, 1H), 7.17 (d, J= 7.7 Hz, 1H), 7.04 (s, 1H), 7.01 (d, J= 7.7 Hz, 1H), 5.48 (bs, 1H), 4.44 (s, 2H), 2.29 (s, 3H), 2.28 (s, 3H). ^{13}C-NMR (DMSO-d$_6$), δ: 168.9, 139.5, 136.2, 134.5, 133.9, 132.9, 132.3, 131.6, 128.8, 128.3, 127.9, 128.8, 126.5, 126.4, 126.1, 125.9, 124.8, 53.0, 21.2, 19.8. UPLC-MS: t_R 1.89 min, MS [ESI, m/z]: 305.2 [M+H$^+$]. HRMS calculated for $C_{20}H_{21}N_2O^+$: 305.1648; found 305.1652.

- *N*'-(3,4-Dichlorobenzyl)-2,4-dihydroxybenzohydrazide **48**

Obtained as a white solid in 75% yield. Melting point: 205-209 °C. ^{1}H-NMR (DMSO-d$_6$), δ: 12.45 (s, 1H), 10.08 (s, 1H), 9.92 (d, J= 7.4 Hz, 1H), 7.66 (s, 1H), 7.60-7.57 (m, 2H), 7.36 (d, J= 6.5 Hz, 1H), 6.27-6.23 (m, 2H), 5.71 (t, J= 4.9 Hz, 1H), 3.99 (d, J= 4.9 Hz, 2H). ^{13}C-NMR (DMSO-d$_6$), δ: 168.9, 162.7, 162.4, 140.6, 130.8, 130.8, 129.2, 107.6, 106.2, 103.2, 53.6. UPLC-MS: t_R 1.76 min, MS [ESI, m/z]: 327.1 [M+H$^+$]. HRMS calculated for $C_{14}H_{13}Cl_2N_2O_3^+$: 327.0298; found 327.0301.

General procedure for the preparation of benzoylhydrazides 49-52, 57

The appropriate benzohydrazide **4a**, **4f**, **55**, **56**, **62**, **63**, **71** or **72** (0.6 mmol, 1 eq.) and the appropriately substituted aromatic acyl chloride (0.6 mmol, 1 eq.) were dissolved in separate solutions of anhydrous THF (3 mL and 4 mL, respectively). The acyl chloride solution was cooled to 0 °C and triethylamine (1.20 mmol, 2 eq.) was added, followed by the dropwise addition of the hydrazide solution. The resulting solution was stirred at room temperature until complete (monitored by TLC), before being poured into water (25 mL). The resulting precipitate was isolated by *vacuum* filtration and washed thoroughly with 40-60 petroleum ether. The solid was then purified by recrystallisation or flash column chromatography.

- *N'*-(2,4-Dihydroxybenzoyl)-1-naphthohydrazide **49**

Purified by recrystallisation from EtOH. Obtained as a white solid in 78% yield. ^{1}H-NMR (DMSO-d$_6$), δ: 12.36 (s, 1H), 10.61 (s, 1H), 10.57 (s, 1H), 10.30 (s, 1H), 8.44 (d, J= 8.3 Hz, 1H), 8.13 (d, J= 8.2 Hz, 1H), 8.10-8.00 (m, 1H), 7.88 (d, J= 8.7 Hz, 1H), 7.80-7.72 (m, 1H), 7.70-7.63 (m, 3H), 6.44 (dd, J_1= 8.7, J_2= 2.3 Hz, 1H), 6.39 (d, J= 2.3 Hz, 1H). ^{13}C-NMR (DMSO-d$_6$), δ: 169.0, 168.3, 163.2, 162.6, 133.5, 132.9, 130.9, 130.3, 130.0, 128.7, 127.4, 126.9, 126.1, 125.8, 125.4, 108.0, 106.0, 103.2. UPLC-MS: t_R 1.55 min, MS [ESI, m/z]: 323.1 [M+H$^+$]. HRMS calculated for $C_{18}H_{15}N_2O_4^+$: 323.1026; found 323.1021.

- *N'*-(3,4-Dichlorobenzoyl)-2,4-dihydroxybenzohydrazide **50**

Purified by recrystallisation from EtOH. Obtained as a white solid in 89% yield. ^{1}H-NMR (DMSO-d$_6$), δ: 12.12 (s, 1H), 10.78 (s, 1H), 10.50 (s, 1H), 10.24 (s, 1H), 8.15 (d, J= 2.0 Hz, 1H), 7.90 (dd, J_1= 8.4 Hz, J_2= 2.0 Hz, 1H), 7.84 (d, J= 8.4 Hz, 1H), 7.79 (d, J= 8.7 Hz, 1H), 6.37 (dd, J_1= 8.7 Hz, J_2 = 2.3 Hz, 1H), 6.32 (d, J= 2.3 Hz, 1H). ^{13}C-NMR (DMSO-d$_6$), δ: 168.8, 163.9, 163.2, 162.3, 135.3, 133.1, 132.0, 131.5, 130.1, 129.8, 128.2, 108.0, 106.0, 103.2. UPLC-MS: t_R 1.61 min, MS [ESI, m/z]: 341.0 [M+H$^+$]. HRMS calculated for $C_{14}H_{11}Cl_2N_2O_4^+$: 341.0090; found 341.0083.

- *N'*-(2,4-Dimethylbenzoyl)-1-naphthohydrazide **51**

Purified by flash column chromatography, eluting with DCM:MeOH 100:0 v/v, increasing to DCM:MeOH 90:10 v/v in 10 CV. Obtained as a white solid in 95% yield. Melting point: 204-207 °C. ^{1}H-NMR (DMSO-d$_6$), δ: 10.43 (s, 1H), 10.21 (s, 1H), 8.43 (d, J= 7.6 Hz, 1H), 8.09 (d, J= 8.1 Hz, 1H), 8.02 (d, J= 7.6 Hz, 1H), 7.71 (d, J= 6.1 Hz, 1H), 7.65-7.29 (m, 3H), 7.41 (d, J= 7.6 Hz, 1H), 7.14 (d, J= 7.6 Hz, 1H), 7.12 (d, J= 8.1 Hz, 1H), 2.45 (s, 3H), 2.34 (s, 3H). ^{13}C-NMR (DMSO-d$_6$), δ: 169.0, 168.4, 140.0, 136.6, 133.2, 132.3, 131.8, 130.8, 130.4, 128.7, 128.1, 127.3, 126.9, 126.5, 126.1, 126.0, 125.5, 21.3, 19.9. UPLC-MS: t_R 2.01 min, MS [ESI, m/z]: 319.4 [M+H$^+$]. HRMS calculated for $C_{20}H_{19}N_2O_2^+$: 319.1441; found 319.1444.

- *N′*-(3,4-Dichlorobenzoyl)-2,4-dimethylbenzohydrazide **52**

Purified by recrystallisation from EtOH. Obtained as a white solid in 37% yield. Melting point: 225-227 °C. ^{1}H-NMR (DMSO-d$_6$), δ: 10.43 (s, 1H), 10.22 (s, 1H), 8.43 (d, J= 8.1 Hz, 1H), 8.09 (d, J= 8.1 Hz, 1H), 7.71 (d, J= 7.2 Hz, 1H), 7.42 (d, J= 8.1 Hz, 1H), 7.14 (s, 1H), 7.12 (d, J= 8.1 Hz, 1H), 2.45 (s, 3H), 2.34 (s, 3H). ^{13}C-NMR (DMSO-d$_6$), δ: 169.0, 168.4, 136.6, 133.6, 133.2, 132.3, 130.8, 128.7, 128.1, 127.3, 126.7, 126.5, 126.1, 125.5, 21.3, 19.9. UPLC-MS: t$_R$ 1.98 min, MS [ESI, m/z]: 337.1 [M+H$^+$]. HRMS calculated for $C_{16}H_{15}Cl_2N_2O_2^+$: 337.0505; found 337.0501.

- 2,4-Dimethyl-*N*′-(4-(4-methylpiperazin-1-yl)benzoyl)benzohydrazide **57**

Purified by recrystallisation from EtOH. Obtained as a white solid in 45% yield. Melting point: 238-242 °C. ^{1}H-NMR (DMSO-d$_6$), δ: 10.17 (s, 1H), 9.95 (s, 1H), 7.82 (d, J= 6.8 Hz, 2H), 7.37-7.34 (m, 1H), 7.10 (bs, 2H), 7.00 (d, J= 6.8 Hz, 2H), 3.21 (bs, 4H), 2.45 (bs, 4H), 2.40 (s, 3H), 2.32(s, 3H), 2.30 (s, 3H). ^{13}C-NMR (DMSO-d$_6$), δ: 169.1, 165.9, 153.5, 139.9, 136.5, 132.6, 131.7, 129.3, 128.0, 126.5, 121.9, 114.0, 54.8, 47.3, 46.2, 21.3, 19.8. UPLC-MS: t$_R$ 0.97 min, MS [ESI, m/z]: 367.3 [M+H$^+$]. HRMS calculated for $C_{21}H_{27}N_4O_2^+$: 367.2129; found 367.2135.

- *N*′-(3-Hydroxy-[1,1′-biphenyl]-4-carbonyl)-1-naphthohydrazide **64**

Purified by flash column chromatography eluting with *n*-hexane:EtOAc 100:0 v/v increasing to *n*-hexane:EtOAc 0:100 v/v in 9 CV. Obtained as a white solid in 68% yield. ^{1}H-NMR (DMSO-d$_6$), δ: 12.24 (s, 1H), 10.91 (s, 1H), 10.74 (s, 1H), 8.47 (d, J= 8.3 Hz, 1H), 8.15 (dd, J$_1$= 12.1 Hz, J$_2$= 8.3 Hz, 2H), 8.09 (d, J= 7.5 Hz, 1H), 7.82-7.80 (m, 3H), 7.72-7.65 (m, 3H), 7.57 (app t, J= 7.5 Hz, 2H), 7.49 (app t, J= 7.5 Hz, 1H), 7.38 (dd, J$_1$= 8.3 Hz, J$_2$= 1.7 Hz, 1H), 7.35 (d, J= 1.7 Hz, 1H). ^{13}C-NMR (DMSO-d$_6$), δ: 168.2, 168.1, 160.3, 146.2, 139.1, 133.6, 132.8, 130.9, 130.4, 129.5, 129.4, 128.9, 128.7, 127.4, 127.3, 126.9, 126.2, 125.8, 125.4, 118.0, 115.5, 113.7. UPLC-MS: t$_R$ 1.88 min, MS [ESI, m/z]: 383.2 [M+H$^+$]. HRMS calculated for $C_{24}H_{19}N_2O_3^+$: 383.1390; found 383.1394.

- *N*′-(3,4-Dichlorobenzoyl)-3-hydroxy-[1,1′-biphenyl]-4-carbohydrazide **65**

Purified by recrystallisation from DCM. Obtained as a white solid in 76% yield. ^{1}H-NMR (DMSO-d$_6$), δ: 12.06 (s, 1H), 11.00 (s, 1H), 10.84 (s, 1H), 8.23 (d, J= 1.5 Hz, 1H), 8.08 (d, J= 8.3 Hz, 1H), 7.98 (dd, J$_1$= 8.3, J$_2$= 1.5 Hz, 1H), 7.90 (d, J= 8.3 Hz, 1H), 7.78 (d, J= 7.5 Hz, 2H), 7.56 (app t, J= 7.5 Hz, 2H), 7.49 (t, J= 7.5 Hz, 1H), 7.35 (d, J= 8.3 Hz, 1H), 7.32 (s, 1H). ^{13}C-NMR (DMSO-d$_6$), δ: 167.7, 163.8, 160.0, 146.2, 139.1, 135.3, 133.0, 132.0, 131.5, 129.9, 129.5, 128.9, 128.2, 127.3, 118.1, 115.5, 113.9. UPLC-MS: t$_R$ 1.95 min, MS [ESI, m/z]: 401.1 [M+H$^+$]. HRMS calculated for $C_{20}H_{15}Cl_2N_2O_3^+$: 401.0454; found 401.0560.

- *N*′-(3,4-Dichlorobenzoyl)-4-(furan-2-yl)-2-hydroxybenzohydrazide **66**

Purified by recrystallisation from DCM. Obtained as a white solid in 64% yield. ^{1}H-NMR (DMSO-d$_6$), δ: 12.03 (s, 1H), 10.93 (s, 1H), 10.74 (s, 1H), 8.16 (dd, J$_1$= 7.4 Hz, J$_2$= 1.9 Hz, 1H), 7.98 (d, J= 8.3 Hz, 1H), 7.91 (td, J$_1$= 8.3 Hz, J$_2$= 1.9 Hz, 1H), 7.86-7.83 (m, 1H), 7.65 (d, J= 8.3 Hz, 1H), 7.40-7.29 (m, 2H), 7.14 (d, J= 3.3 Hz, 1H), 6.66-6.65 (m, 1H). ^{13}C-NMR (DMSO-d$_6$), δ: 166.7, 163.8, 160.0, 159.2, 152.2, 144.6, 135.7, 133.0, 132.0, 131.5, 130.8, 129.9, 128.5, 126.2, 115.6, 114.9, 112.9, 111.7. UPLC-MS: t$_R$ 1.87 min, MS [ESI, m/z]: 391.1 [M+H$^+$]. HRMS calculated for $C_{18}H_{13}Cl_2N_2O_4^+$: 391.0247; found 391.0244.

- *N*′-(4-(1*H*-Tetrazol-5-yl)benzoyl)-2,4-dimethylbenzohydrazide **73**

Purified by recrystallisation from EtOH. Obtained as a white solid in 67% yield. Melting point: 246-249 °C. ^{1}H-NMR (CDCl$_3$), δ: 10.63 (s, 1H), 10.15 (s, 1H), 8.16 (d, J= 8.3 Hz, 2H), 8.11 (d, J= 8.3 Hz, 2H), 7.35 (d, J= 7.8 Hz, 1H), 7.09 (s, 1H), 7.07 (d, J= 7.8 Hz, 1H), 2.38 (s, 3H), 2.29 (s, 3H). ^{13}C-NMR (CDCl$_3$), δ: 168.9, 165.4, 140.1, 136.6, 135.2, 132.3, 131.8, 129.0, 128.0, 127.6, 126.5, 21.3, 19.9. UPLC-MS: t$_R$ 1.43 min, MS [ESI, m/z]: 337.2 [M+H$^+$]. HRMS calculated for $C_{17}H_{17}N_6O_2^+$: 337.1408; found 337.1406.

- *N*′-(3,4-Dichlorobenzoyl)-2-methyl-4-(1*H*-tetrazol-5-yl)benzohydrazide **74**

Purified by recrystallisation from EtOH. Obtained as a white solid in 39% yield. Melting point: 182-183 °C. ^{1}H-NMR (CDCl$_3$), δ: 10.80 (s, 1H), 10.47 (s, 1H), 8.18 (d, J= 2.0 Hz, 1H), 8.00 (s, 1H), 7.97 (d, J= 7.9 Hz, 1H), 7.93 (dd, J$_1$= 8.4 Hz, J$_2$= 2.0 Hz, 1H), 7.89 (dd, J$_1$= 8.4 Hz, J$_2$= 2.0 Hz, 1H), 7.86 (d, J= 8.4 Hz, 1H), 7.65 (d, J= 7.9 Hz, 1H), 2.54 (s, 3H). ^{13}C-NMR (CDCl$_3$), δ: 164.8, 155.2, 137.7, 136.8, 135.4, 133.8, 130.4, 128.0, 124.6, 19.3. UPLC-MS: t$_R$ 1.59 min, MS [ESI, m/z]: 391.1 [M+H$^+$]. HRMS calculated for $C_{16}H_{13}Cl_2N_6O_2^+$: 391.0472; found 391.0469.

Synthesis of 2,4-dimethyl-*N*′-(4-(piperazin-1-yl)benzoyl)benzohydrazide 59

The boc-protected compound **58** (0.12 mmol, 1 eq.) was dissolved in DCM (5 mL) before TFA (0.50 mL, 55 eq.) was added dropwise with stirring. This solution was stirred at room temperature until completion (monitored by TLC), and the solvent was then removed under reduced pressure. The resulting slurry was stirred in diethyl ether (5 mL) until a precipitate formed. The solid was isolated by *vacuum* filtration and washed with cold diethyl ether and 40-60 petroleum ether. The crude reside was purified by recrystallisation from DCM/*n*-hexane to give the title compound as a white solid in 79% yield. Melting point: 231-234 °C. ^{1}H-NMR (DMSO-d$_6$), δ: 10.27 (s, 1H), 10.03 (s, 1H), 8.87 (bs, 2H), 7.87 (d,

J= 8.8 Hz, 2H), 7.35 (d, J= 7.7 Hz, 2H), 7.11-7.08 (m, 1H), 3.51(bs, 4H), 3.25 (bs, 4H), 2.40 (s, 3H), 2.32 (s, 3H). ^{13}C-NMR (DMSO-d_6), δ: 169.1, 165.7, 152.5, 139.9, 136.5, 132.5, 131.7, 129.4, 128.0, 126.5, 123.2, 114.7, 44.8, 43.0, 21.3, 19.8. UPLC-MS: t_R 1.20 min, MS [ESI, m/z]: 353.2 [M+H$^+$]. HRMS calculated for $C_{20}H_{25}N_4O_2^+$: 353.1972; found 353.1765.

General procedure for the preparation of substituted urea or thiourea compounds 82, 83, 85, 88, 89

The appropriately substituted isocyanate or isothiocyanate **79**, **80** or **84** (0.50 mmol, 1 eq.), suspended in toluene (8 mL) at room temperature, was added portion-wise of the appropriate aromatic amine or hydrazine **81**, **86** or **87** (0.50 mmol, 1 eq.). The reaction was allowed to stir at room temperature for 16 hours or until completion (monitored by TLC). The precipitate formed was isolated by *vacuum* filtration and washed with cold 40-60 petroleum ether. The crude solid was purified by recrystallisation or flash column chromatography.

- *N*-(2,4-Dimethylphenyl)-2-(naphthalen-1-ylmethylene)hydrazine-1-carboxamide **82**

Purified by recrystallisation from EtOH. Obtained as a white solid in 47% yield. Melting point: 194-196 °C. ^1H-NMR (DMSO-d_6), δ: 8.65 (d, J= 8.2 Hz, 1H), 8.42 (s, 1H), 7.93 (d, J= 7.3 Hz, 1H), 7.82 (d, J= 8.2 Hz, 1H), 7.71 (d, J= 7.3 Hz, 1H), 7.57-7.47 (m, 4H), 6.98 (bs, 2H), 6.86 (bs, 2H), 2.45 (s, 3H), 2.23 (s, 3H). ^{13}C-NMR (DMSO-d_6), δ: 162.5, 138.0, 131.1, 129.7, 129.0, 128.1, 126.7, 126.3, 126.1, 125.6, 124.7, 124.6, 21.7, 21.2. UPLC-MS: t_R 1.97 min, MS [ESI, m/z]: 318.2 [M+H$^+$]. HRMS calculated for $C_{20}H_{20}N_3O^+$: 318.1601; found 318.1605.

- *N*-(3,4-Dichlorophenyl)-2-(naphthalen-1-ylmethylene)hydrazine-1-carboxamide **83**

Purified by recrystallisation from EtOH. Obtained as a white solid in 89% yield. Melting point: 223-226 °C. ^1H-NMR (DMSO-d_6), δ: 10.99 (s, 1H), 9.29 (s, 1H), 8.83 (s, 1H), 8.34 (app t, J= 6.6 Hz, 2H), 8.09 (s, 1H), 8.02 (d, J= 8.2 Hz, 2H), 7.74 (d, J= 6.6 Hz, 1H), 7.68-7.51 (m, 4H). ^{13}C-NMR (DMSO-d_6), δ: 153.3, 140.0, 133.8, 131.2, 130.9, 130.8, 130.4, 129.9, 129.3, 127.6, 126.6, 126.0, 124.2, 123.1, 121.3, 120.2. UPLC-MS: t_R 2.14 min, MS [ESI, m/z]: 358.1 [M+H$^+$]. HRMS calculated for $C_{18}H_{14}Cl_2N_3O^+$: 358.0508; found 358.0503.

- *N*-(2,4-Dimethylphenyl)-2-(naphthalen-1-ylmethylene)hydrazine-1-carbothioamide **85**

Purified by flash column chromatography eluting with DCM:MeOH 95:5 v/v. Obtained as golden crystals in 49% yield. ^1H-NMR ($CDCl_3$), δ: 10.22 (s, 1H), 9.10 (s, 1H), 8.63 (s, 1H), 8.33 (d, J= 8.1 Hz, 1H), 7.88-7.82 (m, 3H), 7.52-7.43 (m, 3H), 7.23 (s, 2H), 6.82 (s, 1H), 2.27 (s, 6H). ^{13}C-NMR ($CDCl_3$), δ: 175.7, 141.9, 138.5, 137.6, 133.8, 131.2, 130.9, 129.0, 128.8, 128.1, 127.4, 126.9, 126.3, 125.3, 123.2, 122.3, 21.3. UPLC-MS: t_R 2.16 min, MS [ESI, m/z]: 334.1 [M+H$^+$]. HRMS calculated for $C_{20}H_{20}N_3S^+$: 334.1372; found 334.1370.

- 1-(3,4-Dichlorophenyl)-3-(2,4-dimethylphenyl)urea **88**

Purified by recrystallisation from EtOH. Obtained as a white solid in 99% yield. Melting point: 202-206 °C. ^1H-NMR (DMSO-d_6), δ: 9.21 (s, 1H), 7.95 (s, 1H), 7.59 (d, J= 8.1 Hz, 1H), 7.50 (d, J= 8.8 Hz, 1H), 7.29 (d, J= 8.8 Hz, 1H), 7.27-7.13 (m, 1H), 7.00 (s, 1H), 6.96 (d, J= 8.1 Hz, 1H), 2.23 (s, 3H), 2.20 (s, 3H). ^{13}C-NMR (DMSO-d_6), δ: 153.0, 140.7, 134.7, 132.8, 131.5, 131.2, 131.0, 129.0, 127.1, 123.3, 122.5, 119.5, 118.5, 20.8, 18.2. UPLC-MS: t_R 2.06 min, MS [ESI, m/z]: 309.1 [M+H$^+$]. HRMS calculated for $C_{15}H_{15}Cl_2N_2O^+$: 309.0556; found 309.0548.

- *N*-(3,4-Dichlorophenyl)-2-((2,4-dimethylphenyl)amino)acetamide **89**

Purified by recrystallisation from EtOH. Obtained as a white solid in 75% yield. Melting point: 206-209 °C. ^1H-NMR (DMSO-d_6), δ: 8.81 (s, 1H), 7.86 (d, J= 2.5 Hz, 1H), 7.46 (d, J= 8.8 Hz, 1H), 7.25 (m, 1H), 7.12 (d, J= 7.8 Hz, 1H), 6.99 (s, 1H), 6.97 (d, J= 7.8 Hz, 1H), 6.61 (t, J= 5.6 Hz, 1H), 4.23 (d, J= 5.6 Hz, 2H), 2.26 (s, 3H), 2.25 (s, 3H). ^{13}C-NMR (DMSO-d_6), δ: 155.2, 141.2, 136.3, 135.8, 134.9, 131.4, 131.2, 130.9, 128.1, 126.8, 122.7, 119.1, 118.6, 41.1, 21.0, 19.0. UPLC-MS: t_R 2.08 min, MS [ESI, m/z]: 323.1 [M+H$^+$]. HRMS calculated for $C_{16}H_{17}Cl_2N_2O^+$: 323.0712; found 323.0709.

General procedure for the preparation of substituted amides 91-93, 96-101 and 103

The appropriately substituted carboxylic acid **90**, **94**, **95** or 2,4-dimethylbenzoic acid (0.85 mmol, 1 eq.) and TBTU (0.85 mmol, 1 eq.) were dissolved in THF (5 mL) under a N_2 atmosphere, and added of DiPEA (1.79 mmol, 2.1 eq.). The appropriate aromatic or aliphatic amine (0.85 mmol, 1 eq.), dissolved in THF (2 mL), was then added to the solution, and the reaction was stirred at room temperature until completion (monitored by TLC). The solvent was then removed under reduced pressure and the resulting residue was dissolved in ethyl acetate (20 mL). The organic layer was then washed with sat. aq. $NaHCO_3$ solution (20 mL) and brine (20 mL), dried over Na_2SO_4, and the solvent removed under reduced pressure. This crude solid was purified by recrystallisation or flash column chromatography.

- *N*^{1}-(3,4-Dichlorophenyl)-*N*^{2}-(2,4-dimethylphenyl)oxalamide **91**

Purified by recrystallisation from DCM. Obtained as a white solid in 67% yield. Melting point: 197-201 °C. ^{1}H-NMR (DMSO-d$_6$), δ: 11.14 (s, 1H), 10.28 (s, 1H), 8.23 (d, J= 2.5 Hz, 1H), 7.90 (q, J= 2.5, 8.8 Hz, 1H), 7.66 (d, J= 8.8 Hz, 1H), 7.31 (d, J= 8.0 Hz, 1H), 7.10 (s, 1H), 7.05 (d, J= 8.0 Hz, 1H), 2.28 (s, 3H), 2.20 (s, 3H). ^{13}C-NMR (DMSO-d$_6$), δ: 159.5, 158.5, 138.3, 136.0, 132.8, 132.7, 131.5, 131.4, 131.1, 127.2, 126.8, 125.4, 122.3, 121.1, 21.0, 18.0. UPLC-MS: t$_R$ 2.17 min, MS [ESI, m/z]: 337.1 [M+H$^+$]. HRMS calculated for $C_{16}H_{15}Cl_2N_2O_2^+$: 337.0505; found 337.0510.

- *N*^{1}-(2,4-Dimethylphenyl)-*N*^{2}-(4-hydroxynaphthalen-1-yl)oxalamide **92**

Purified by flash column chromatography eluting with DCM:MeOH 100:0 v/v increasing to DCM:MeOH 90:10 v/v in 10 CV. Obtained as a light pink solid in 70% yield. ^{1}H-NMR (CDCl$_3$), δ: 9.71 (s, 1H), 9.32 (s, 1H), 8.27 (d, J= 8.3 Hz, 1H), 7.98-7.96 (m, 1H), 7.92-7.89 (m, 2H), 7.62-7.56 (m, 3H), 7.11, (d, J= 8.3 Hz, 1H), 7.07 (s, 1H), 2.35 (s, 3H), 2.34 (s, 3H). ^{13}C-NMR (CDCl$_3$), δ: 155.7, 151.4, 142.6, 140.1, 133.1, 131.5, 131.4, 127.5, 122.7, 120.5, 117.3, 115.6, 105.0, 104.9, 102.7, 101.9, 99.2, 94.2, 28.3, 25.6. UPLC-MS: t$_R$ 1.95 min, MS [ESI, m/z]: 335.2 [M+H$^+$]. HRMS calculated for $C_{20}H_{19}N_2O_3^+$: 335.1390; found 335.1388.

- *N*^{1}-(2,4-Dimethylphenyl)-*N*^{2}-(1-methyl-2-oxabicyclo[2.1.1]hexan-4-yl)oxalamide **93**

Purified by recrystallisation from DCM. Obtained as a white solid in 77% yield. Melting point: 233-235 °C. ^{1}H-NMR (DMSO-d$_6$), δ: 9.13 (s, 1H), 8.03 (s, 1H), 7.84 (d, J= 8.1 Hz, 1H), 7.05 (m, 2H), 3.92 (s, 2H), 2.31 (s, 3H), 2.29 (s, 3H), 2.04 (app q, J= 6.3, 4H), 1.49 (s, 3H). ^{13}C-NMR (DMSO-d$_6$), δ: 160.3, 157.0, 131.4, 127.5, 121.5, 69.2, 56.9, 46.9, 21.0, 17.8, 17.5. UPLC-MS: t$_R$ 1.72 min, MS [ESI, m/z]: 289.1 [M+H$^+$]. HRMS calculated for $C_{16}H_{21}N_2O_3^+$: 289.1547; found 289.1543.

- (*E*)-*N*-(3,4-Dichlorophenyl)-3-(2,4-dimethylphenyl)acrylamide **96**

Purified by recrystallisation from EtOAc. Obtained as a white solid in 73% yield. Melting point: 172-175 °C. ^{1}H-NMR (CDCl$_3$), δ: 8.54 (d, J= 8.4 Hz, 1H), 8.40 (d, J= 15.7 Hz, 1H), 8.05 (d, J= 8.4 Hz, 1H), 7.81 (app t, J= 7.8 Hz, 1H), 7.70 (d, J= 7.8 Hz, 1H), 7.63-7.58 (m, 2H), 7.11 (d, J= 8.1 Hz, 1H), 7.09 (s, 1H), 2.44 (s, 3H), 2.31 (s, 3H). ^{13}C-NMR (CDCl$_3$), δ: 162.1, 146.7, 142.3, 139.0, 133.0, 131.9, 130.0, 127.6, 127.1, 126.9, 116.5, 115.6, 114.2, 21.6, 19.8. UPLC-MS: t$_R$ 2.01 min, MS [ESI, m/z]: 320.1 [M+H$^+$]. HRMS calculated for $C_{17}H_{16}Cl_2NO^+$: 320.0603; found 320.0609.

- (*E*)-*N*-(3,4-Dichlorobenzyl)-3-(2,4-dimethylphenyl)acrylamide **97**

Purified by recrystallisation from EtOH. Obtained as a white solid in 78% yield. Melting point: 173-177 °C. ^{1}H-NMR (CDCl$_3$), δ: 7.95 (d, J= 15.4 Hz, 1H), 7.42-7.38 (m, 3H), 7.16 (m, 1H), 7.02 (s, 1H), 6.99 (d, J= 8.1 Hz, 1H), 6.30 (d, J= 15.4 Hz, 1H), 6.04 (bs, 1H), 4.52 (d, J= 6.1 Hz, 2H), 2.40 (s, 3H), 2.32 (s, 3H). ^{13}C-NMR (CDCl$_3$), δ: 166.2, 140.0, 139.8, 138.7, 137.7, 132.7, 131.6, 131.5, 130.7, 129.7, 127.2, 127.1, 126.0, 119.7, 42.6, 21.3, 19.8. UPLC-MS: t$_R$ 2.07 min, MS [ESI, m/z]: 334.1 [M+H$^+$]. HRMS calculated for $C_{18}H_{18}Cl_2NO^+$: 334.0760; found 334.0766.

- (*E*)-3-(3,4-Dichlorophenyl)-*N*-(2,4-dimethylphenyl)acrylamide **98**

Purified by recrystallisation from EtOH. Obtained as a white solid in 88% yield. ^{1}H-NMR (DMSO-d$_6$), δ: 9.40 (s, 1H), 7.91 (s, 1H), 7.71 (d, J= 8.3 Hz, 1H), 7.63 (d, J= 8.0 Hz, 1H), 7.54 (d, J= 15.8 Hz, 1H), 7.47 (d, J= 8.0 Hz, 1H), 7.06-6.99 (m, 3H), 2.26 (s, 3H), 2.21 (s, 3H). ^{13}C-NMR (DMSO-d$_6$), δ: 163.5, 137.5, 136.3, 134.6, 132.2, 132.1, 131.6, 131.5, 129.9, 127.9, 126.9, 125.2, 124.7, 20.9, 18.3. UPLC-MS: t$_R$ 2.06 min, MS [ESI, m/z]: 320.0 [M+H$^+$]. HRMS calculated for $C_{17}H_{16}Cl_2NO^+$: 320.0603; found 320.0605.

- (*E*)-3-(3,4-Dichlorophenyl)-*N*-(2,4-dimethylbenzyl)acrylamide **99**

Purified by recrystallisation from EtOH. Obtained as a white solid in 91% yield. ^{1}H-NMR (DMSO-d$_6$), δ: 8.43 (t, J= 5.5 Hz, 1H), 7.84 (d, J= 1.8 Hz, 1H), 7.68 (d, J= 8.4 Hz, 1H), 7.56 (dd, J_1= 8.4 Hz, J_2= 1.8 Hz, 1H), 7.44 (d, J= 15.8 Hz, 1H), 7.12 (d, J= 7.7 Hz, 1H), 7.0-6.96 (m, 2H), 6.78 (d, J= 15.8 Hz, 1H), 4.34 (d, J= 5.5 Hz, 2H), 2.25 (s, 3H), 2.20 (s, 3H). ^{13}C-NMR (DMSO-d$_6$), δ: 164.7, 136.6, 136.5, 136.3, 136.0, 134.1, 132.1, 132.0, 131.5, 131.1, 129.8, 128.5, 127.7, 126.7, 124.8, 40.8, 21.0, 19.0. UPLC-MS: t$_R$ 2.04 min, MS [ESI, m/z]: 334.1 [M+H$^+$]. HRMS calculated for $C_{18}H_{18}Cl_2NO^+$: 334.0760; found 334.0753.

- (*E*)-3-(2,4-Dimethylphenyl)-*N*-((2-hydroxynaphthalen-1-yl)methyl)acrylamide **100**

Purified by recrystallisation from DCM. Obtained as a light pink solid in 74% yield. ^{1}H-NMR (CDCl$_3$), δ: 10.26 (s, 1H), 7.96 (s, 1H), 7.85 (d, J= 8.5 Hz, 1H), 7.80 (d, J= 8.0 Hz, 1H), 7.74 (d, J= 8.5 Hz, 1H), 7.52 (app t, J= 7.5 Hz, 1H), 7.36 (s, 1H), 7.34 (t, J= 6.5 Hz, 1H), 7.00 (s, 1H), 6.97 (d, J= 8.0 Hz, 1H), 6.59-6.54 (m, 2H), 6.24 (d, J= 15.5 Hz, 1H), 4.92 (d, J= 6.5 Hz, 2H), 2.38 (s, 3H), 2.31 (s, 3H). ^{13}C-NMR (CDCl$_3$), δ: 173.4, 155.7, 139.6, 136.3, 134.7, 134.6, 134.5, 124.7, 124.6, 124.5, 130.0, 128.2, 128.1, 128.0, 126.5, 125.0, 123.2, 122.3, 115.5, 45.6, 20.6, 19.7. UPLC-MS: t$_R$ 2.08 min, MS [ESI, m/z]: 332.2 [M+H$^+$]. HRMS calculated for $C_{22}H_{22}NO_2^+$: 332.1645; found 332.1641.

- (*E*)-3-(2,4-Dimethylphenyl)-*N*-(3-fluorobicyclo[1.1.1]pentan-1-yl)acrylamide **101**

Purified by recrystallisation from EtOH. Obtained as a white solid in 94% yield. ^{1}H-NMR (DMSO-d$_6$), δ: 8.81 (s, 1H), 7.62 (d, J= 15.7 Hz, 1H), 7.41 (d, J= 7.8 Hz, 1H), 7.06-7.04 (m, 2H), 6.42 (d, J= 15.7 Hz, 1H), 2.39 (s, 4H), 2.35 (s, 2H), 2.33

(s, 3H), 2.28 (s, 3H). ^{13}C-NMR (DMSO-d$_6$), δ: 166.1, 139.4, 137.2, 137.1, 131.8, 131.1, 127.5, 126.4, 122.1, 77.8, 75.6, 55.3, 55.2, 21.2, 19.7. ^{19}F-NMR (DMSO-d$_6$), δ: -163.77. UPLC-MS: t_R 1.88 min, MS [ESI, m/z]: 260.1 [M+H$^+$]. HRMS calculated for $C_{16}H_{18}FNO^+$: 260.1445; found 260.1443.

- *N*-(5,6-Dichloro-1*H*-benzo[*d*]imidazol-2-yl)-2,4-dimethylbenzamide **103**

Purified by recrystallisation from EtOH. Obtained as a white solid in 69% yield. Melting point: 176-180 °C. ^{1}H-NMR (DMSO-d$_6$), δ: 7.22 (s, 2H), 7.50 (d, J= 7.8 Hz, 1H), 7.46 (s, 1H), 7.38 (s, 1H), 7.31 (d, J= 7.8 Hz, 1H), 5.82 (s, 1H), 2.47 (s, 3H), 2.24 (s, 3H). ^{13}C-NMR (DMSO-d$_6$), δ: 169.7, 156.4, 144.1, 142.4, 135.7, 132.3, 131.4, 130.7, 128.0, 127.96, 126.9, 121.1, 116.9, 113.8, 21.5, 18.9. UPLC-MS: t_R 2.05 min, MS [ESI, m/z]: 334.1 [M+H$^+$]. HRMS calculated for $C_{16}H_{14}Cl_2N_3O^+$: 334.0508; found 334.0505.

Synthesis of 2-(2,4-dimethylphenyl)-5-(naphthalen-1-yl)-1,3,4-oxadiazole 104

2,4-Diethylbenzohydrazide **4f** (0.120 g, 0.73 mmol, 1 eq.) and 1-naphthylaldehyde (0.099 mL, 0.73 mmol, 1 eq.) were refluxed in tert-BuOH (7 mL) for 2 hours. NaOCl (0.226 mL, 3.65 mmol, 5 eq.) was then added at 0°C and the mixture was stirred at 30°C overnight. Additional NaOCl (0.226 mL, 3.65 mmol, 5 eq.) was added and the reaction was stirred at room temperature fro further 5 hours. The solvent was evaporated under *vacuum* and the crude residue was purified by flash column chromatography eluting with DCM:MeOH 100:0 v/v increasing to DCM:MeOH 90:10 v/v in 15 CV to give the title compound as a light orange solid in 33% yield. ^{1}H-NMR (DMSO-d$_6$), δ: 9.26 (d, J= 8.6 Hz, 1H), 8.16 (d, J= 7.6 Hz, 1H), 7.94 (d, J= 8.1 Hz, 1H), 7.90 (d, J= 8.1 Hz, 1H), 7.84 (d, J= 8.1 Hz, 1H), 7.61 app (t, J= 7.6 Hz, 1H), 7.52-7.49 (m, 2H), 7.11-7.08 (m, 2H), 2.70 (s, 3H), 2.32 (s, 3H). ^{13}C-NMR (DMSO-d$_6$), δ: 164.5, 163.9, 141.7, 138.4, 133.9, 132.6, 132.4, 130.1, 129.0, 128.6, 128.2, 128.1, 127.0, 126.7, 126.3, 124.9, 120.6, 120.1, 22.2, 21.4. UPLC-MS: t_R 2.28 min, MS [ESI, m/z]: 301.0 [M+H$^+$]. HRMS calculated for $C_{20}H_{17}N_2O^+$: 301.1335; found 301.1342.

Synthesis of 2-(3,4-dichlorophenyl)-5-(2,4-dimethylphenyl)-1,3,4-oxadiazole 105

2,4-Diethylbenzohydrazide **4f** (0.300g, 1.82 mmol, 1 eq.) and 3,4-dichlorobenzoic acid (0.350 g, 1.82 mmol, 1 eq.) were refluxed in $POCl_3$ (10 mL) overnight. The mixture was concentrated under *vacuum* and the residue was poured onto ice. The mixture was extracted with EtOAc (3x 30 mL) and the combined organic extracts were washed with sat. aq. $NaHCO_3$ solution (2x 80 mL). The organic layer was dried over Na_2SO_4 and concentrated under *vacuum*. The residue was purified by flash column chromatography eluting with DCM:MeOH 100:0 v/v increasing to DCM:MeOH 90:10 v/v in 15 CV to give the title compound as a white solid in 46% yield. ^{1}H-NMR (DMSO-d$_6$), δ: 8.24 (s, 1H), 8.01 (d, J= 8.4 Hz, 1H), 7.95 (d, J= 8.4 Hz, 1H), 7.87-7.82 (m, 1H), 7.21-7.17 (m, 2H), 2.59 (s, 3H), 2.30 (s, 3H). ^{13}C-NMR (DMSO-d$_6$), δ: 165.2, 162.1, 142.2, 138.2, 135.1, 132.8, 132.2, 129.6, 128.7, 127.6, 127.2, 124.4, 119.9, 21.9, 21.4. UPLC-MS: t_R 2.31 min, MS [ESI, m/z]: 319.0 [M+H$^+$]. HRMS calculated for $C_{16}H_{13}Cl_2N_2O^+$: 319.0399; found 319.0393.

Synthesis of 4-(3,4-dichlorophenyl)-1-(2,4-dimethylbenzyl)-1*H*-1,2,3-triazole 107

1-Azidomethyl-2,4-dimethylbenzene **106** (0.384 g, 2.38 mmol, 1.6 eq.) and 1,2-dichloro-4-ethynylbenzene (0.313 g, 1.83 mmol, 1 eq.) were dissolved in THF: water 9:1 (5 mL). $CuSO_4$ (0.014 g, 0.9 mmol, 0.5 eq.) and sodium ascorbate (0.145 g, 0.73 mmol, 0.3 eq.) were added to the mixture, and the solution was stirred at room temperature overnight. The solution was then poured over crushed ice and the formed precipitate was dissolved in EtOAc (15 mL). The organic layer was washed with water (15 mL) and brine (15 mL), dried over Na_2SO_4 and concentrated under reduced pressure. The crude residue was purified by flash column chromatography eluting with *n*-hexane:EtOAc 100:0 v/v increasing to *n*-hexane:EtOAc 40:60 v/v in 10 CV to give the title compound as a white solid in 84% yield. ^{1}H-NMR (DMSO-d$_6$), δ: 8.63 (s, 1H), 8.12 (d, J= 2.0 Hz, 1H), 7.87 (dd, J_1= 8.4 Hz, J_2= 2.0 Hz, 1H), 7.70 (d, J= 8.4 Hz, 1H), 7.12 (d, J= 7.7 Hz, 1H), 7.09 – 6.99 (m, 2H), 5.61 (s, 2H), 2.29 (s, 3H), 2.27 (s, 3H). ^{13}C-NMR (DMSO-d$_6$), δ: 144.7, 138.2, 136.8, 132.1, 131.8, 131.6, 131.6, 131.1, 130.5, 129.7, 127.3, 127.2, 125.6, 122.8, 51.6, 21.1, 19.0. UPLC-MS: t_R 2.18 min, MS [ESI, m/z]: 332.1 [M+H$^+$]. HRMS calculated for $C_{17}H_{16}Cl_2N_3^+$: 332.0716; found 332.0720.

Procedures for the preparation and characterisation of synthetic intermediates

General procedure for the preparation of ethyl esters

Intermediate esters **3n**, **3p-3s**, **3u-3z** were purchased from commercial sources (Sigma-Aldrich, Fisher Scientific, Alfa-Aesar or Fluorochem). For the remaining ethyl esters used, the appropriately substituted benzoic acid (1 g) was suspended in ethanol (5 mL) before concentrated sulfuric acid (1 mL) was added dropwise with stirring. The solution was heated to reflux until completion (monitored by T.L.C.). The solution was then cooled to room temperature and the pH adjusted to ~5 using sat. aq. $NaHCO_3$, before being extracted with ethyl acetate (3x 15 mL). The combined organic layers were dried over Na_2SO_4 and the solvent removed under reduced pressure, to afford the desired intermediate which did not require further purification unless stated otherwise.

- Ethyl 2,4-dihydroxybenzoate **3a**

Obtained as a dark orange oil in 81% yield. ^{1}H-NMR (DSMO-d_6), δ: 11.55 (s, 1H), 8.18 (d, J= 8.7 Hz, 1H), 7.49 (m, 2H), 4.81 (q, J= 14.3, 7.1 Hz, 2H), 1.83 (t, J= 7.1 Hz, 3H).

- Ethyl 2,4-dimethoxybenzoate **3c**

Obtained as an orange oil in 89% yield. ^{1}H-NMR ($CDCl_3$), δ: 7.85-7.83 (m, 1H), 6.49-6.47 (m, 2H), 4.31 (q, J=7.1 Hz, 2H), 3.88 (s, 3H), 3.84 (s, 3H), 1.35 (t, J=7.1 Hz, 3H). ^{13}C-NMR ($CDCl_3$), δ: 165.6, 164.1, 161.4, 133.7, 112.7, 104.5, 99.0, 60.4, 56.0, 55.5, 14.4.

- Ethyl 4-hydroxybenzoate **3d**

Obtained as a white solid in 83% yield. ^{1}H-NMR ($CDCl_3$), δ: 7.98-7.95 (m, 2H), 6.89-6.87 (m, 2H), 6.15 (s, 2H), 4.63 (q, J= 7.1 Hz, 2H), 1.35 (t, J= 7.1 Hz, 3H). ^{13}C-NMR ($CDCl_3$), δ: 166.6, 159.8, 131.9, 123.0, 115.2, 60.9, 14.4.

- Ethyl 2-hydroxybenzoate **3e**

Obtained as a white solid in 80% yield. ^{1}H-NMR ($CDCl_3$), δ: 10.84 (s, 1H), 7.85 (d, J= 7.9 Hz, 1H), 7.44 (app t, J= 7.9 Hz, 1H), 6.97 (d, J= 8.1 Hz, 1H), 6.87 (app t, J= 8.1 Hz, 1H), 4.41 (q, J= 7.13 Hz, 2H), 1.41 (t, J= 7.1 Hz, 3H). ^{13}C-NMR ($CDCl_3$), δ: 170.2, 161.7, 135.6, 129.9, 119.1, 117.5, 112.6, 61.4, 14.2.

- Ethyl 2,4-dimethylbenzoate **3f**

Obtained as a yellow oil in 84% yield. ^{1}H-NMR ($CDCl_3$), δ: 7.75-7.70 (m, 1H), 6.97-6.91 (m, 2H), 4.24 (q, J= 7.1Hz, 2H), 2.47 (s, 3H), 2.24 (s, 3H), 1.15 (t, J= 7.1 Hz, 3H). ^{13}C-NMR ($CDCl_3$), δ: 171.1, 167.6, 142.3, 140.2, 132.4, 130.7, 126.4, 60.5, 21.7, 21.3, 14.3.

- Ethyl 2,4-difluorobenzoate **3g**

Obtained as a yellow oil in 92% yield. ^{1}H-NMR (DMSO-d_6), δ: 7.96 (s, 1H), 6.93-6.83 (m, 2H), 4.37 (q, J= 7.2 Hz, 2H), 1.38 (t, J= 7.2 Hz, 3H). ^{13}C-NMR (DMSO-d_6), δ: 171.2, 166.6, 164.6, 163.6, 161.7, 133.8, 111.6, 105.2, 61.4, 14.2.

- Ethyl 2,4-dichlorobenzoate **3h**

Obtained as a yellow oil in 94% yield. ^{1}H-NMR ($CDCl_3$), δ: 7.79 (s ,1H), 7.46 (s, 1H), 7.29 (s, 1H), 4.40 (bs, 2H), 1.40 (bs, 3H). ^{13}C-NMR ($CDCl_3$), δ: 171.1, 164.7, 138.1, 134.8, 132.4, 130.9, 126.9, 61.7, 14.1.

- Ethyl 2,4-bis(trifluoromethyl)benzoate **3i**

Obtained as a yellow oil in 81% yield. ^{1}H-NMR (DMSO-d_6), δ: 7.98 (s, 1H), 7.89 (s, 2H), 4.44-4.40 (m, 2H), 1.43-1.37 (m, 3H). ^{13}C-NMR (DMSO-d_6), δ: 171.2, 165.7, 130.8, 62.6, 13.8. ^{19}F-NMR (DMSO-d_6), δ: -58.44, -61.33.

- Ethyl isonicotinate **3j**

Obtained as a yellow oil in 87% yield. ^{1}H-NMR (DMSO-d_6), δ: 8.71 (s, 2H), 7.79 (s, 2H), 4.35 (s, 2H), 1.39-1.33 (m, 3H). ^{13}C-NMR (DMSO-d_6), δ: 171.1, 165.1, 150.5, 122.8, 61.7, 14.1.

- Ethyl picolinate **3k**

Obtained as a pale yellow oil in 83% yield. ^{1}H-NMR (DMSO-d_6), δ: 8.70 (s, 1H), 8.07 (s, 1H), 7.80 (s, 1H), 7.42 (s, 1H), 4.48-4.39 (m, 2H), 1.43-1.36 (m, 3H). ^{13}C-NMR (DMSO-d_6), δ: 160.4, 145.1, 132.2, 122.0, 120.3, 57.2, 9.5.

- Ethyl furan-2-carboxylate **3l**

Obtained as an orange oil in 81% yield. ^{1}H-NMR ($CDCl_3$), δ: 7.50 (s, 1H), 7.10 (s, 1H), 6.43 (s, 1H), 4.28 (m, 2H), 1.30 (m, 3H). ^{13}C-NMR ($CDCl_3$), δ: 170.9, 158.6, 146.1, 117.6, 111.7, 60.7, 14.1.

- Ethyl 1*H*-imidazole-2-carboxylate **3m**

Obtained as a white solid in 87% yield. ^{1}H-NMR (DMSO-d_6), δ: 11.27 (bs, 1H), 7.22 (s, 2H), 4.35 (bs, 2H), 1.32 (bs, 3H). ^{13}C-NMR (DMSO-d_6), δ: 168.7, 159.1, 139.0, 125.6, 61.8, 14.2.

- Ethyl 4-isopropylbenzoate **3o**

Obtained as a colourless oil in 91% yield. ^{1}H-NMR ($CDCl_3$), δ: 7.87 (d, J= 8.3 Hz, 2H), 7.16 (d, J= 8.3 Hz, 2H), 4.25 (q, J= 7.2 Hz, 2H), 2.87-2.79 (m, 1H), 1.26 (t, J= 7.2 Hz, 3H), 1.14 (, J= 6.8 Hz, 6H). ^{13}C-NMR ($CDCl_3$), δ: 170.5, 166.0, 129.0, 125.7, 60.0, 33.5, 23.7, 14.3.

- Ethyl 2,5-dimethylbenzoate **3t**

Obtained as a colourless oil in 99% yield. ^{1}H-NMR ($CDCl_3$), δ: 7.56 (s, 1H), 7.02 (d, J= 7.2 Hz, 1H), 6.94 (d, J= 7.2 Hz, 1H), 4.19 (q, J= 7.2 Hz, 2H), 2.38 (s, 3H), 2.17 (s, 3H), 1.22 (t, J= 7.2 Hz, 3H). ^{13}C-NMR ($CDCl_3$), δ: 167.8, 136.8, 135.1, 132.6, 131.6, 130.9, 129.7, 60.6, 21.2, 20.7, 14.3.

- Ethyl 4-bromo-2-methylbenzoate **3aa**

Obtained as an orange oil in 87% yield. ^{1}H-NMR (DMSO-d_6), δ: 7.74 (d, J= 8.4 Hz, 1H), 7.59 (s, 1H), 7.52 (dd, J_1= 8.4 Hz, J_2= 1.7 Hz, 1H), 4.29 (q, J= 7.1 Hz, 2H), 2.52 (s, 3H), 1.31 (t, J= 7.1 Hz, 3H). ^{13}C-NMR (DMSO-d_6), δ: 166.6, 142.1, 134.5, 132.4, 129.4, 129.2, 126.1, 61.2, 21.1, 14.5.

- Ethyl 2-hydroxy-4-iodobenzoate

Obtained as an orange oil in 83% yield. ^{1}H-NMR (DMSO-d_6), δ: 10.69 (s, 1H), 7.38 (dd, J_1= 8.3 Hz, J_2= 1.6 Hz), 7.28 (d, J= 1.6 Hz, 1H), 7.20 (dd, J_1= 8.3 Hz, J_2= 1.6 Hz, 1H), 4.40 (q, J= 7.1 Hz, 2H), 1.38 (t, J= 7.1 Hz, 3H). ^{13}C-NMR (DMSO-d_6), δ: 168.6, 160.9, 131.8, 128.2, 126.7, 113.3, 103.2, 61.7, 14.4.

General procedure for the Suzuki coupling reaction to prepare intermediates 3ab, 60 and 61

The appropriate ester **3aa** or ethyl 2-hydroxy-4-iodobenzoate (0.60 mmol, 1 eq.) was dissolved in a 9:1 mix of toluene:MeOH (5 mL), then the proper boronic acid (0.71 mmol, 1.2 eq.) was added, followed by $PdCl_2$ (0.23 mmol, 0.4 eq.) and K_2CO_3 (1.8 mmol, 3 eq.). The reaction was stirred at 60 °C for 3 hours, then cooled to room temperature and filtered over a celite pad. The filtrate was concentrated under reduced pressure, and the residue was purified by flash column chromatography eluting with *n*-hexane:EtOAc 100:0 v/v increasing to *n*-hexane:EtOAc 40:60 v/v in 10 CV, to afford the desired intermediate **3ab**, **60** or **61**.

- Ethyl 3-methyl-[1,1'-biphenyl]-4-carboxylate **3ab**

Obtained as a colourless oil in 76% yield. ^{1}H-NMR (DSMO-d_6), δ: 7.84 (d, J= 8.1 Hz, 1H), 7.67-7.65 (m, 2H), 7.59-7.58 (m, 1H), 7.54 (dd, J_1= 8.1 Hz, J_2= 1.7 Hz, 1H), 7.44-7.40 (m, 2H), 7.39-7.34 (m, 1H), 4.24 (q, J= 7.1 Hz, 2H), 2.53 (s, 3H), 1.27 (t, J= 7.1 Hz, 3H). ^{13}C-NMR (DMSO-d_6), δ: 167.1, 143.9, 140.3, 139.3, 131.3, 130.2, 129.4, 128.8, 128.7, 127.3, 124.6, 60.9, 21.7, 14.6.

- Ethyl 3-hydroxy-[1,1'-biphenyl]-4-carboxylate **60**

Obtained as a colourless oil in 66% yield. ^{1}H-NMR (DSMO-d_6), δ: 10.57 (s, 1H), 7.80 (d, J= 8.7 Hz, 1H), 7.65 (d, J= 7.3 Hz, 2H), 7.43 (app t, J= 7.3 Hz, 2H), 7.38-7.34 (m, 1H), 7.22-7.20 (m, 1H), 4.32 (q, J= 7.1 Hz, 2H), 1.29 (t, J= 7.1 Hz, 3H). ^{13}C-NMR (DMSO-d_6), δ: 169.3, 161.1, 147.6, 138.9, 131.0, 130.9, 129.4, 129.1, 127.4, 118.3, 115.4, 115.4, 112.2, 61.8, 14.4.

- Ethyl 4-(furan-2-yl)-2-hydroxybenzoate **61**

Obtained as an orange oil in 64% yield. ^{1}H-NMR (DSMO-d_6), δ: 10.63 (s, 1H), 7.84 (d, J= 1.5 Hz, 1H), 7.49 (d, J= 8.3 Hz, 1H), 7.41 (d, J= 1.5 Hz, 1H), 7.30 (t, J= 1.5 Hz, 1H), 7.18 (d, J= 3.3 Hz, 1H), 6.66 (dd, J_1= 3.3 Hz, J_2= 1.5 Hz, 1H), 3.90 (s, 2H), 0.85 (t, J= 7.1 Hz, 3H). ^{13}C-NMR (DMSO-d_6), δ: 169.4, 161.0, 152.0, 144.8, 137.0, 131.8, 131.2, 128.8, 126.5, 111.6, 109.6, 52.9, 22.9.

General procedure for the preparation of hydrazides

Intermediate hydrazides **4n**, **4p-r**, **4w-4x**, **4z** were purchased from commercial sources (Sigma-Aldrich, Fisher Scientific, Alfa-Aesar or Fluorochem). For the remaining hydrazides used, the appropriately substituted ethyl or methyl ester (1.00 mmol, 1 eq.) was dissolved in ethanol (5 mL) before hydrazine monohydrate (20.00 mmol, 20 eq.) was added dropwise with stirring. The solution was heated to reflux over night or until completion (monitored by TLC), then cooled to room

temperature and then further cooled on an ice bath until a precipitate formed, which was isolated by *vacuum* filtration and washed with cold 40-60 petroleum ether. In those cases where a precipitate did not form, the solution was partitioned between water (15 mL) and ethyl acetate (15 mL). The water layer was extracted twice further with fresh ethyl acetate (2x 15 mL), and the combined organic layers were dried over Na_2SO_4 before the solvent was removed under reduced pressure, to afford the desired intermediate which did not require further purification unless stated otherwise.

- 2,4-Dihydroxybenzohydrazide **4a**

Obtained as a white solid in 58% yield. ^{1}H-NMR (DSMO-d_6), δ: 12.74 (s, 1H), 10.02 (s, 1H), 9.74 (s, 1H), 7.62 (d, J= 8.7 Hz, 1H), 6.26-6.22 (m, 2H), 4.49 (bs, 2H). ^{13}C-NMR (DMSO-d_6), δ: 169.1, 162.5, 128.8, 107.5, 106.1, 103.2.

- 2,4-Dimethoxybenzohydrazide **4c**

Obtained as an orange oil in 59% yield. ^{1}H-NMR (DSMO-d_6), δ: 8.00 (bs, 1H), 7.76-7.74 (m, 1H), 6.62-6.60 (m, 2H), 4.51 (bs, 2H), 3.87 (s, 3H), 3.80 (s, 3H). ^{13}C-NMR (DMSO-d_6), δ: 164.8, 163.1, 158.9, 132.4, 114.5, 106.0, 98.8, 56.3, 55.9.

- 4-Hydroxybenzohydrazide **4d**

Obtained as a white solid in 60% yield. ^{1}H-NMR (DSMO-d_6), δ: 9.47 (s, 1H), 7.72-7.63 (m, 2H), 6.22-6.72 (m, 2H), 4.35 (bs, 2H). ^{13}C-NMR (DMSO-d_6), δ: 166.4, 160.5, 129.3, 124.4, 115.3.

- 2-Hydroxybenzohydrazide **4e**

Obtained as a white solid in 64% yield. ^{1}H-NMR (DSMO-d_6), δ: 10.05 (s, 1H), 8.92 (s, 1H), 7.79 (m, 1H), 7.73 (app t, J= 8.2 Hz, 1H), 6.90-6.83 (m, 2H), 6.63 (bs, 2H). ^{13}C-NMR (DMSO-d_6), δ: 169.1, 160.0, 133.9, 127.5, 119.1, 117.7.

- 2,4-Dimethylbenzohydrazide **4f**

Obtained as a white solid in 59% yield. ^{1}H-NMR (DSMO-d_6), δ: 9.31 (s, 1H), 7.18 (d, J= 7.9 Hz, 1H), 7.04 (s, 1H), 7.00 (d, J= 7.9 Hz, 1H), 4.41 (bs, 2H), 2.30 (s, 3H), 2.28 (s, 3H). ^{13}C-NMR (DMSO-d_6), δ: 169.0, 139.4, 136.1, 131.6, 127.8, 126.4, 21.2, 19.9.

- 2,4-Difluorobenzohydrazide **4g**

Obtained as a white solid in 66% yield. ^{1}H-NMR (DSMO-d_6), δ: 9.53 (s, 1H), 7.63 (m, 1H), 7.33 (m, 1H), 7.16 (m, 1H), 4.55 (bs, 2H). ^{13}C-NMR (DMSO-d_6), δ: 163.0, 132.0, 120.2, 112.2, 105.0.

- 2,4-Dichlorobenzohydrazide **4h**

Obtained as a white solid in 72% yield. ^{1}H-NMR (DSMO-d_6), δ: 9.62 (s, 1H), 7.67 (s, 1H), 7.47-7.42 (m, 2H), 4.52 (bs, 2H). ^{13}C-NMR (DMSO-d_6), δ: 165.2, 135.1, 132.1, 131.0, 129.6, 127.7.

- 2,4-bis(Trifluoromethyl)benzohydrazide **4i**

Obtained as a white solid in 74% yield. ^{1}H-NMR (DSMO-d_6), δ: 9.75 (s, 1H), 8.13 (m, 2H), 7.75 (m, 1H), 4.42 (bs, 2H). ^{13}C-NMR (DMSO-d_6), δ: 165.6, 139.5, 130.9, 130.0, 128.0, 127.8, 124.7, 123.8, 122.2. ^{19}F-NMR (DMSO-d_6), δ: -56.97, -60.81.

- Isonicotinohydrazide **4j**

Obtained as a white solid in 81% yield. ^{1}H-NMR (DSMO-d_6), δ: 10.09 (s, 1H), 8.69 (s, 2H), 7.73 (s, 2H), 4.62 (bs, 2H). ^{13}C-NMR (DMSO-d_6), δ: 164.4, 150.7, 140.7, 121.5.

- Picolinohydrazide **4k**

Obtained as a white solid in 90% yield. ^{1}H-NMR (DSMO-d_6), δ: 9.87 (s, 1H), 8.60 (m, 1H), 8.00-7.95 (m, 2H), 7.56 (m, 1H), 4.56 (bs, 2H). ^{13}C-NMR (DMSO-d_6), δ: 169.1, 163.1, 149.0, 138.1, 126.7, 122.2.

- Furan-2-carbohydrazide **4l**

Obtained as a pale yellow oil in 88% yield. ^{1}H-NMR (DSMO-d_6), δ: 9.64 (s, 1H), 7.80 (m, 1H), 7.08 (m, 1H), 6.59 (m, 1H), 4.44 (bs, 2H). ^{13}C-NMR (DMSO-d_6), δ: 169.2, 158.4, 145.3, 113.3, 112.0.

- 1*H*-Imidazole-2-carbohydrazide **4m**

Obtained as a white solid in 98% yield. ^{1}H-NMR (DSMO-d_6), δ: 10.02 (s, 1H), 7.64 (bs, 1H) 7.14 (bs, 2H), 4.67 (bs, 2H). ^{13}C-NMR (DMSO-d_6), δ: 168.7, 158.4, 140.6, 124.4.

- 4-Isopropylbenzohydrazide **4o**

Obtained as a white solid in 78% yield. ^{1}H-NMR (DSMO-d_6), δ: 9.69 (s, 1H), 7.75 (d, J= 8.2 Hz, 2H), 7.31 (d, J= 8.2 Hz, 2H), 3.78 (bs, 2H), 2.97-2.88 (m, 1H), 1.21 (d, J= 6.9 Hz, 6H). ^{13}C-NMR (DMSO-d_6), δ: 166.4, 152.1, 131.4, 127.5, 126.7, 33.8, 24.1.

- 2,5-Dihydroxybenzohydrazide **4s**

Obtained as a white solid in 92% yield. ^{1}H-NMR (DSMO-d_6), δ: 11.55 (s, 1H), 9.85 (s, 1H), 8.97 (s, 1H), 7.19 (d, J= 2.9 Hz, 1H), 6.83 (d, J= 8.8 Hz, 1H), 6.73 (d, J= 8.8 Hz, 1H), 4.59 (bs, 2H). ^{13}C-NMR (DMSO-d_6), δ: 167.9, 152.2, 149.7, 121.3, 118.1, 115.4, 113.4.

- 2,5-Dimethylbenzohydrazide **4t**

Obtained as a white solid in 60% yield. ^{1}H-NMR (DSMO-d_6), δ: 9.34 (s, 1H), 7.12 (m, 2H), 7.10 (s, 1H), 4.42 (bs, 2H), 2.27 (s, 3H), 2.18 (s, 3H). ^{13}C-NMR (DMSO-d_6), δ: 169.0, 136.0, 134.8, 132.9, 130.8, 130.3, 128.3, 20.9, 19.4.

- 2-Hydroxy-4-methylbenzohydrazide **4u**

Obtained as a white solid in 71% yield. ^{1}H-NMR (DSMO-d_6), δ: 12.43 (bs, 1H), 9.99 (s, 1H), 7.69 (d, J= 8.1 Hz, 1H), 6.71 (s, 1H), 6.68 (d, J= 8.1 Hz, 1H), 4.60 (bs, 2H), 2.26 (s, 3H). ^{13}C-NMR (DMSO-d_6), δ: 168.6, 160.3, 144.3, 127.3, 120.1, 117.9, 19.9.

- 4-Hydroxy-2-methylbenzohydrazide **4v**

Obtained as a white solid in 75% yield. ^{1}H-NMR (DSMO-d_6), δ: 9.62 (bs, 1H), 9.18 (s, 1H), 7.16 (d, J= 8.3 Hz, 1H), 6.60 (s, 1H), 6.57 (d, J= 8.3 Hz, 1H), 4.35 (bs, 2H), 2.28 (s, 3H). ^{13}C-NMR (DMSO-d_6), δ: 168.4, 159.7, 144.1, 127.5, 118.1, 21.3.

- 2,6-Dihydroxybenzohydrazide **4y**

Obtained as an off-white solid in 71% yield. ^{1}H-NMR (DSMO-d_6), δ: 11.54 (s, 2H), 7.25 (t, J= 8.2 Hz, 1H), 7.15 (t, J= 8.2 Hz, 1H), 6.45 (d, J= 8.2 Hz, 2H), 3.41 (s, 2H). ^{13}C-NMR (DMSO-d_6), δ: 160.4, 134.1, 132.9, 107.8, 102.5.

- 3-Methyl-[1,1'-biphenyl]-4-carbohydrazide **4ab**

Obtained as a white solid in 77% yield. ^{1}H-NMR (DSMO-d_6), δ: 9.50 (s, 1H), 7.73 (d, J= 7.4 Hz, 2H), 7.60 (s, 1H), 7.57-7.51 (m, 3H), 7.45-7.43 (m, 2H), 4.52 (d, J= 3.6 Hz, 2H), 2.47 (s, 3H). ^{13}C-NMR (DMSO-d_6), δ: 168.7, 141.5, 139.9, 136.8, 135.0, 129.4, 129.2, 128.4, 128.2, 127.2, 124.1, 20.0.

- 4-(4-Methylpiperazin-1-yl)benzohydrazide **55**

Obtained as a white solid in 71% yield. ^{1}H-NMR (DSMO-d_6), δ: 9.50 (s, 1H), 7.70 (d, J= 8.9 Hz, 2H), 6.93 (d, J= 8.9 Hz, 2H), 4.02 (bs, 2H), 3.22 (t, J= 4.9 Hz, 4H), 2.42 (t, J= 4.9 Hz, 4H), 2.21 (s, 3H). ^{13}C-NMR (DMSO-d_6), δ: 166.4, 153.1, 128.6, 122.9, 114.1, 54.9, 47.4, 46.2.

- *tert*-Butyl 4-(4-(hydrazinecarbonyl)phenyl)piperazine-1-carboxylate **56**

Obtained as a white solid in 74% yield. ^{1}H-NMR (DSMO-d_6), δ: 9.52 (s, 1H), 7.72 (d, J= 8.8 Hz, 2H), 6.95 (d, J= 8.8 Hz, 2H), 4.36 (bs, 2H), 3.45 (bs, 4H), 3.22 (bs, 4H), 1.42 (s, 9H). ^{13}C-NMR (DMSO-d_6), δ: 166.3, 154.3, 152.9, 128.6, 123.3, 114.5, 79.5, 47.5, 46.0, 28.5.

- 3-Hydroxy-[1,1'-biphenyl]-4-carbohydrazide **62**

Obtained as a white solid in 71% yield. ^{1}H-NMR (DSMO-d_6), δ: 12.64 (bs, 1H), 10.18 (s, 1H), 7.95 (d, J= 8.6 Hz, 1H), 7.75 (d, J= 7.6 Hz, 2H), 7.53 (t, J= 7.6 Hz, 2H), 7.45 (t, J= 7.6 Hz, 1H), 7.26 – 7.19 (m, 2H), 4.73 (s, 2H). ^{13}C-NMR (DMSO-d_6), δ: 168.2, 160.5, 145.4, 139.3, 129.4, 128.7, 128.1, 127.2, 117.6, 115.4.

- 4-(Furan-2-yl)-2-hydroxybenzohydrazide **63**

Obtained as a white solid in 98% yield. ^{1}H-NMR (DSMO-d_6), δ: 10.15 (bs, 1H), 8.89 (s, 1H), 7.91 (d, J= 8.3 Hz, 1H), 8.86 (s, 1H), 7.28-7.25 (m, 2H), 7.14 (d, J= 3.4 Hz, 1H), 3.40 (s, 2H). ^{13}C-NMR (DMSO-d_6), δ: 167.5, 152.3, 144.3, 135.0, 128.2, 127.8, 126.3, 115.1, 114.4, 113.5, 111.7.

- 4-(1*H*-Tetrazol-5-yl)benzohydrazide **71**

Obtained as a white solid in 78% yield. ^{1}H-NMR (DSMO-d_6), δ: 11.78 (bs, 2H), 8.25 (d, J= 8.4 Hz, 2H), 8.14 (d, J= 8.4 Hz, 2H), 3.44 (bs, 2H). ^{13}C-NMR (DMSO-d_6), δ: 165.6, 133.1, 129.2, 127.8.

- 2-Methyl-4-(1*H*-tetrazol-5-yl)benzohydrazide **73**

Obtained as a white solid in 56% yield. ^{1}H-NMR ($CDCl_3$), δ: 9.95 (s, 1H), 7.94 (s, 1H), 7.88 (d, J= 7.9 Hz, 1H), 7.48 (d, J= 7.9 Hz, 1H), 2.43 (s, 3H). ^{13}C-NMR ($CDCl_3$), δ: 168.1, 137.4, 129.2, 128.8, 124.6, 19.8.

General procedure for the preparation of substituted ethyl (4-piperazinyl)benzoates, 53-54

Ethyl 4-fluorobenzoate (0.50 mL, 2.74 mmol, 1 eq.) and the appropriately *N*-substituted piperazine (2.74 mmol, 1 eq.) were dissolved in DMSO (8 mL) before K_2CO_3 (5.48 mmol, 2 eq.) was added while stirring. The solution was heated to reflux until completion (monitored by TLC), then cooled to room temperature and diluted with water (15 mL). The solution was then extracted with ethyl acetate (3x 15 mL) and the combined organic layers were washed with water (20 mL) and brine (20 mL) before being dried over Na_2SO_4 and concentrated under reduced pressure. The crude residue was purified by flash column chromatography.

- Ethyl 4-(4-methylpiperazin-1-yl)benzoate **53**

Purified by flash column chromatography eluting with DCM:MeOH 100:0 v/v increasing to DCM:MeOH 85:15 in 13 CV. Obtained as a white solid in 87% yield. ^{1}H-NMR ($CDCl_3$), δ: 7.92 (d, J= 8.9 Hz, 2H), 6.87 (d, J= 8.9, 2H), 4.32 (q, J= 7.1 Hz, 2H), 3.34 (t, J= 5.1 Hz, 4H), 2.55 (t, J= 5.1 Hz, 4H), 2.35 (s, 3H), 1.36 (t, J= 7.1 Hz, 3H). ^{13}C-NMR ($CDCl_3$), δ: 165.9, 135.6, 130.8, 119.6, 111.7, 60.9, 57.2, 52.0, 46.6, 14.1.

- *tert*-Butyl 4-(4-(ethoxycarbonyl)phenyl)piperazine-1-carboxylate **54**

Purified by flash column chromatography eluting with DCM:MeOH 100:0 v/v increasing to DCM:MeOH 90:10 in 12 CV. Obtained as a white solid in 74% yield. ^{1}H-NMR (DSMO-d_6), δ: 7.94 (d, J= 8.8 Hz, 2H), 6.86 (d, J= 8.8 Hz, 2H), 4.32 (q, J= 7.1 Hz, 2H), 3.58 (t, J= 4.8 Hz, 4H), 3.29 (t, J= 4.8 Hz, 4H), 1.48 (s, 9H), 1.37 (t, J= 4.8 Hz, 3H). ^{13}C-NMR (DSMO-d_6), δ: 166.6, 154.7, 131.2, 114.3, 80.0, 60.5, 47.9, 28.4, 14.5.

Synthesis of *tert*-butyl 4-(4-(2-(2,4-dimethylbenzoyl)hydrazine-1-carbonyl)phenyl)piperazine-1-carboxylate, 58

Substituted benzohydrazide **56** (0.100 g, 0.6 mmol, 1 eq.) and 2,4-dimethylbenzoyl chloride (0.6 mmol, 1 eq.) were dissolved in separate solutions of anhydrous THF (3 mL and 4 mL, respectively). The acyl chloride solution was cooled to 0 °C and triethylamine (0.17 mL, 1.20 mmol, 2 eq.) was added, followed by the dropwise addition of the hydrazide solution. The resulting solution was stirred at room temperature for 16 hours, and then poured into water (25 mL). The resulting precipitate was isolated by vacuum filtration and washed thoroughly with 40-60 petroleum ether. The crude residue was purified by recrystallisation from DCM/*n*-hexane to afford the title compound as a white solid in 46% yield. ^{1}H-NMR (DMSO-d_6), δ: 10.22 (s, 1H), 9.99 (s, 1H), 7.83 (d, J= 8.8 Hz, 2H), 7.35 (d, J= 7.8 Hz, 2H), 7.11-7.08 (m, 2H), 7.02 (d, J= 8.8 Hz, 2H), 3.47 (bs, 4H), 3.28 (bs, 4H), 2.40 (s, 3H), 2.32 (s, 3H), 1.43 (s, 9H). ^{13}C-NMR (DMSO-d_6), δ: 169.1, 165.38, 154.3, 139.9, 136.5, 132.5, 131.7, 129.3, 128.0, 126.5, 114.3, 79.5, 47.3, 28.5, 21.3, 19.9.

General procedure for the preparation of substituted methyl 4-(1*H*-tetrazol-5-yl)benzoates, 69-70

The appropriate methyl 4-cyanobenzoate **67** or **68** (6.22 mmol, 1 eq.) was dissolved in DMF (12 mL) under a N_2 atmosphere before sodium azide (9.33 mmol, 1.5 eq.) and ammonium chloride (6.22 mmol, 1 eq.) were added with stirring. This solution heated under reflux until completion (monitored by TLC), before being cooled to room temperature and quenched with sat. aq. $NaHCO_3$ solution (20 mL). The solution was then acidified with 2 M HCl until a precipitate formed, which was isolated by vacuum filtration and washed with cold 40-60 petroleum ether to give a crude solid. The crude residue was purified by flash column chromatography.

- Methyl 4-(1H-tetrazol-5-yl)benzoate **69**

Purified by flash column chromatography eluting with DCM:MeOH 100:0 v/v increasing to DCM:MeOH 85:15 in 12 CV. Obtained as a white solid in 85% yield. ^{1}H-NMR (DSMO-d_6), δ: 12.45 (bs, 1H), 7.75 (bs, 2H), 7.36 (bs, 2H), 3.79 (s, 3H). ^{13}C-NMR (DSMO-d_6), δ: 165.9, 155.2, 136.1, 130.1, 127.3, 51.3.

- Methyl 2-methyl-4-(1*H*-tetrazol-5-yl)benzoate **70**

Purified by flash column chromatography eluting with DCM:MeOH 100:0 v/v increasing to DCM:MeOH 90:10 in 13 CV. Obtained as a white solid in 87% yield. ^{1}H-NMR ($CDCl_3$), δ: 12.98 (s, 1H), 8.07 (d, J= 7.8 Hz, 1H), 8.03 (s, 1H), 7.97 (d, J= 7.8 Hz, 1H), 3.95 (s, 3H), 2.70 (s, 3H). ^{13}C-NMR ($CDCl_3$), δ: 168.0, 155.2, 140.1, 134.8, 130.3, 129.9, 124.4, 51.5, 18.8.

General procedure for the preparation of benzoyl azides, 77-78

The appropriate benzoyl chloride **75** or **76** (14.32 mmol, 1 eq.) was dissolved in acetone (10 mL). Sodium azide (20.61 mmol, 1.4 eq.) was dissolved in water (5 mL) and cooled to 0 °C. The benzoyl chloride solution was added to the azide solution at 0 °C over one hour before being stirred at room temperature for 16 hours. The acetone was then removed under reduced pressure and the remaining solution was diluted with water (15 mL) and extracted with ethyl acetate

(3x 30 mL). The combined organic layers were dried over Na_2SO_4 and the solvent removed under reduced pressure to give a crude solid, which was then purified by flash column chromatography.

• 2,4-Dimethylbenzoyl azide **77**

Purified by flash column chromatography eluting with EtOAc:MeOH 100:0 v/v increasing to EtOAc:MeOH 90:10 v/v in 10 CV. Obtained as a white solid in 70% yield. ^{1}H-NMR (DSMO-d_6), δ: 7.50 (d, J= 8.1 Hz, 1H), 6.86 (m, 2H), 2.43 (s, 3H), 2.22 (s, 3H). ^{13}C-NMR (DSMO-d_6), δ: 168.7, 136.3, 134.8, 129.1, 128.9, 127.8, 21.3, 20.0.

• 3,4-Dichlorobenzoyl azide **78**

Purified by flash column chromatography eluting with EtOAc:MeOH 100:0 v/v increasing to EtOAc:MeOH 85:15 v/v in 10 CV. Obtained as a white solid in 78% yield. ^{1}H-NMR (DSMO-d_6), δ: 8.10 (s, 1H), 7.84 (d, J= 7.4 Hz, 1H), 7.54 (d, J= 7.4 Hz, 1H). ^{13}C-NMR (DSMO-d_6), δ: 170.7, 139.1, 133.4, 131.3, 130.8, 130.4, 128.4.

General procedure for the preparation of iocyanates, 79-80

The appropriate benzoyl azide **77** or **78** (0.50 mmol, 1 eq.) was suspended in toluene (8 mL) and heated to 120 °C with stirring for 4 hours before being cooled to room temperature to afford the desired isocyanate, which was not isolated, but used directly for the next synthetic stage.

Synthesis of (*E*)-(naphthalen-1-ylmethylene)hydrazine, 81

1-Naphthaldehyde (11.5 mmol, 1 eq.) was suspended in ethanol (10 mL) and hydrazine monohydrate (34.5 mmol, 3 eq.) was added dropwise with stirring. The solution was heated to reflux overnight, and then cooled to room temperature. The solvent was removed under reduced pressure to afford a crude residue which was purified by recrystallisation from DCM/*n*-hexane to afford the title compound as a white solid in 93% yield. ^{1}H-NMR (DMSO-d_6), δ: 8.67 (d, J= 8.3 Hz, 1H), 8.42 (s, 1H), 7.93 (d, J= 6.6 Hz, 1H), 7.82 (d, J= 6.6 Hz, 1H), 7.72 (d, J= 6.6 Hz, 1H), 7.57-7.48 (m, 3H), 6.97 (bs, 2H). ^{13}C-NMR (DMSO-d_6), δ: 146.5, 133.4, 130.6, 128.0, 127.8, 127.7, 126.4, 125.9, 124.7.

Synthesis of 1-isothiocyanato-2,4-dimethylbenzene, 84

Thiophosgene (1.24 mmol, 1 eq.) was added dropwise to a stirred solution of 2,4-dimethylaniline (1.24 mmol, 1 eq.) and Et_3N (3.71mmol, 3 eq.) in anhydrous THF (5 mL). The mixture was stirred at 0°C for 30 minutes, then the reaction was stirred at room temperature for further 30 minutes. Water (15 mL) was then added to the mixture, and the product was extracted with EtOAc (3x 20 mL). The combined organic layers were dried over Na_2SO_4 and concentrated under *vacuum*, giving the desired intermediate in 99% yield, which was used for the next step without further characterisation.

Synthesis of 2-((2,4-dimethylphenyl)amino)-2-oxoacetic acid, 90

a. 2,4-Dimethylaniline **86** (1.83 mmol, 1 eq.) was dissolved in DCM (10 mL) under a N_2 atmosphere before adding Et_3N (2.01 mmol, 1.1 eq.) and cooling the solution to 0°C. Ethyl oxalylchloride (1.83 mmol, 1 eq.) was added to the solution at 0°C over 10 minutes and allowed to stir at 0°C for 2 hours. The solution was then diluted with DCM (10 mL) and washed with 2 M HCl (15 mL), sat. $NaHCO_3$ (15 mL), and brine (15 mL). The organic layer was then dried over Na_2SO_4 and the solvent removed under reduced pressure to afford the intermediate ester ethyl 2-((2,4-dimethylphenyl)amino)-2-oxoacetate as a white solid in 97% yield. ^{1}H-NMR ($CDCl_3$), δ: 8.77 (s, 1H), 7.87 (d, J= 8.2 Hz, 1H), 7.05 (d, J= 8.2 Hz, 1H), 7.03 (s, 1H), 4.42 (q, J= 7.2 Hz, 2H), 2.30 (s, 3H), 2.28 (s, 3H), 1.43 (t, J= 7.2 Hz, 3H). ^{13}C-NMR ($CDCl_3$), δ: 161.3, 1539, 135.7, 131.8, 131.3, 128.6, 127.5, 121.9, 63.7, 20.9, 17.5, 14.0.

b. Ethyl 2-((2,4-dimethylphenyl)amino)-2-oxoacetate (1.5 mmol, 1 eq) was dissolved in ethanol (5 mL) and cooled to 0 °C. Potassium hydroxide (4.5 mmol, 3 eq.) was dissolved in water (5 mL) and added to the ethanol solution with stirring at 0 °C. This solution was allowed to stir at room temperature for two hours before being adjusted to pH 1 with concentrated HCl. The solvent was then removed under reduced pressure and the resulting slurry was recrystallised from water. The purified crystals were filtered and washed with cold water and 40-60 petroleum ether to give the title product as a white solid in 93% yield. ^{1}H-NMR ($CDCl_3$), δ: 8.86 (s, 1H), 7.80 (d, J= 8.0 Hz, 1H), 7.08 (s, 1H), 7.06 (bs, 1H), 2.32 (s, 3H), 2.31 (s, 3H). ^{13}C-NMR ($CDCl_3$), δ: 160.2, 154.9, 136.7, 131.6, 130.9, 128.8, 127.7, 121.7, 21.0, 17.4.

Synthesis of 5,6-dichloro-1*H*-benzo[*d*]imidazol-2-amine, 102

4,5-Dichloro-1,2-diaminobenzene (4.69 mmol, 3.2 eq.) was dissolved in a 1:1 mix of methanol: water (80 mL) before cyanogen bromide (1.47 mmol, 1 eq.) was added, and the solution heated to 50 °C for one hour. The solution was then cooled to room temperature before the methanol was removed under reduced pressure. The remaining solution was basified with 1 M aq. NaOH to reach pH ~ 8. This solution was then extracted with ethyl acetate (4x 30 mL), and the combined organic layers were washed with water (2x 50 mL) and brine (2x 50 mL) before being dried over Na_2SO_4. The solvent was then removed under reduced pressure to afford the title compound as a white solid in 73% yield. ^{1}H-NMR (DMSO-d_6), δ: 10.91 (bs, 1H), 7.26 (s, 2H), 6.53 (bs, 2H). ^{13}C-NMR (DMSO-d_6), δ: 159.3, 141.0, 137.2, 130.9, 120.2, 117.6.

Synthesis of 1-(azidomethyl)-2,4-dimethylbenzene, 106

2,4-Dimethylbenzyl bromide (1.27 mmol, 1 eq.) and sodium azide (6.41 mmol, 5 eq.) were dissolved in DMF (5.4 mL) under a N_2 atmosphere and then heated to 80 °C for 72 hours. The solution was then cooled to room temperature and extracted with DCM (3x 20 mL) before the combined organic layers were washed with water (3x 20 mL) and brine (20 mL). The organic layers were then dried over Na_2SO_4 and the solvent removed under reduced pressure to afford the title compound as a white solid in 95% yield. ^{1}H-NMR (DMSO-d_6), δ: 7.20 (d, J= 7.6 Hz, 1H), 7.07 (s, 1H), 7.02 (d, J= 7.6 Hz, 1H), 4.41 (s, 2H), 2.30 (s, 3H), 2.26 (s, 3H). ^{13}C-NMR (DMSO-d_6), δ: 138.9, 136.7, 135.3, 130.8, 128.8, 125.9, 53.0, 21.6, 19.1.

Representative ^{1}H, ^{13}C and ^{19}F-NMR spectra for intermediates and final products

Ethyl 2,4-dimethylbenzoate, **3f**

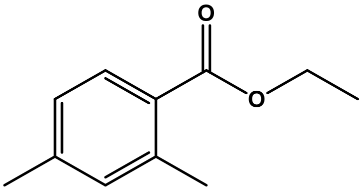

^{1}H-NMR ($CDCl_3$):

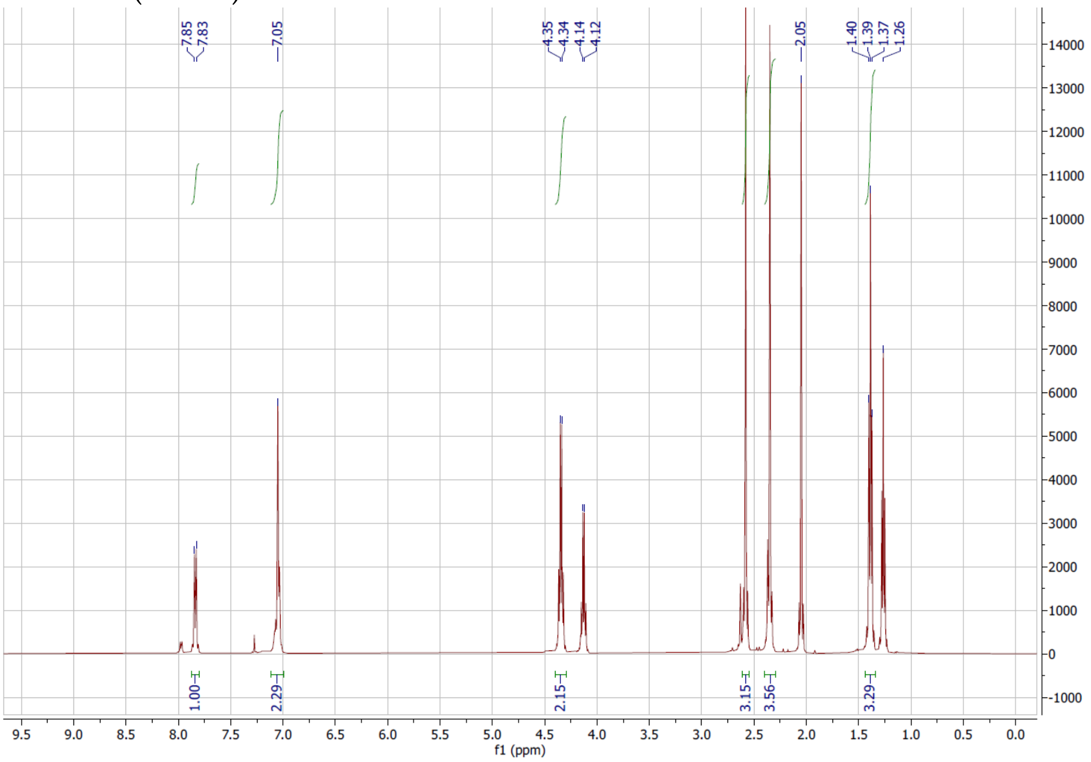

^{13}C-NMR ($CDCl_3$):

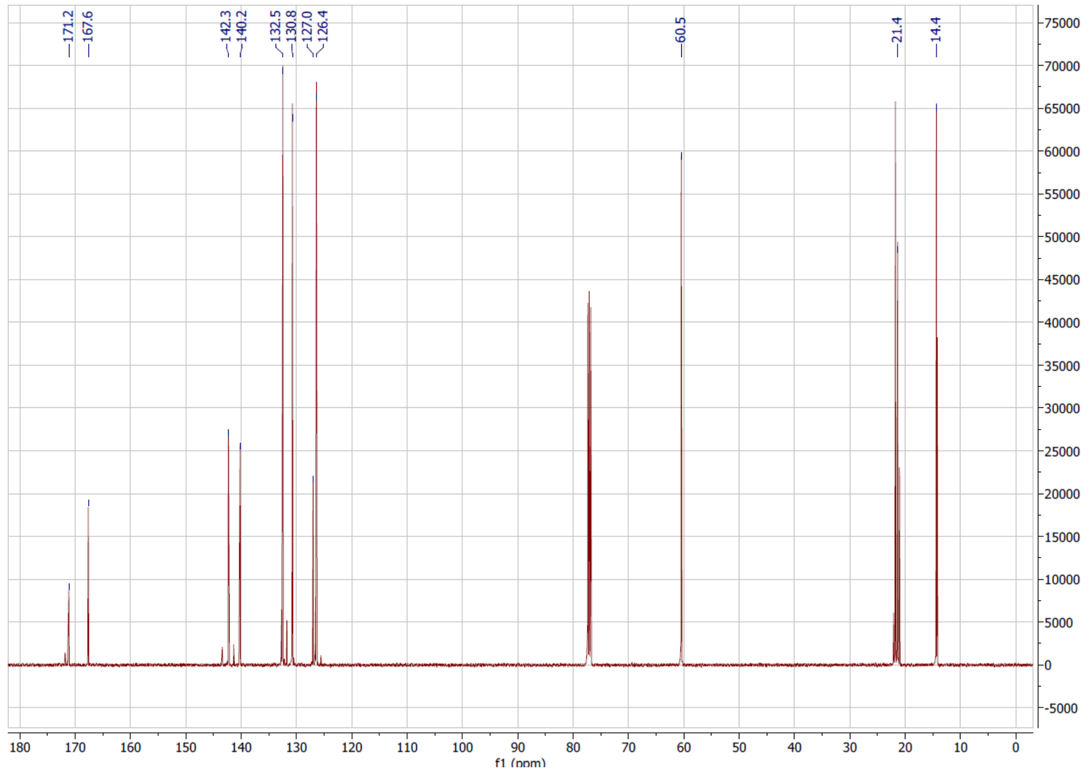

Ethyl 2,4-dichlorobenzoate, **3h**

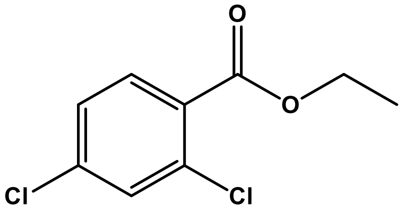

^{1}H-NMR ($CDCl_3$):

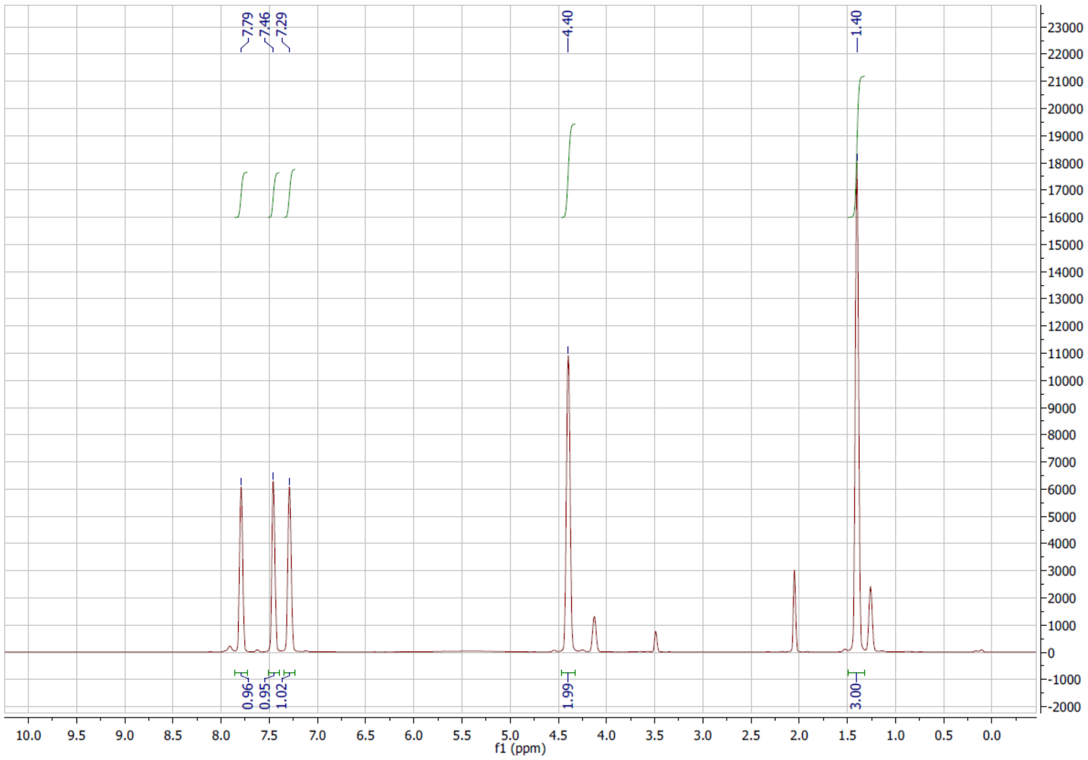

^{13}C-NMR ($CDCl_3$):

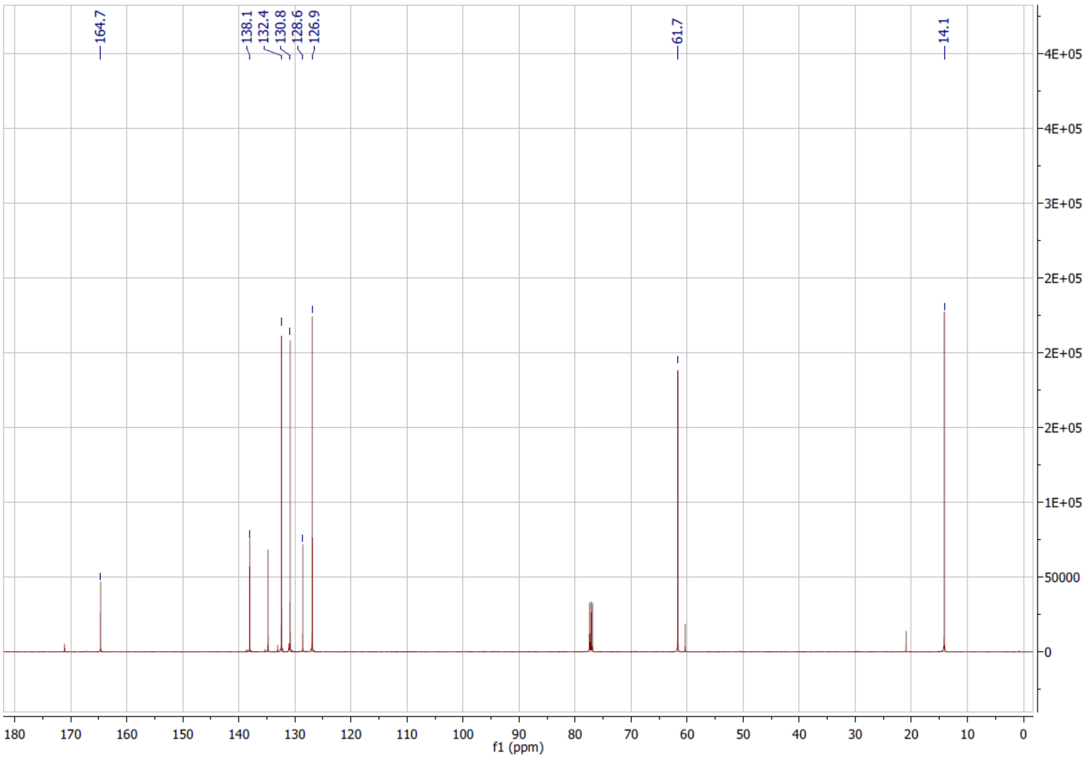

Ethyl 4-bromo-2-methylbenzoate, **3aa**

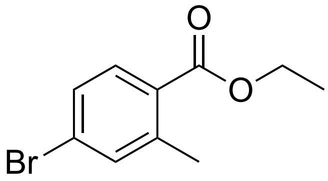

^{1}H-NMR (DMSO-d$_6$):

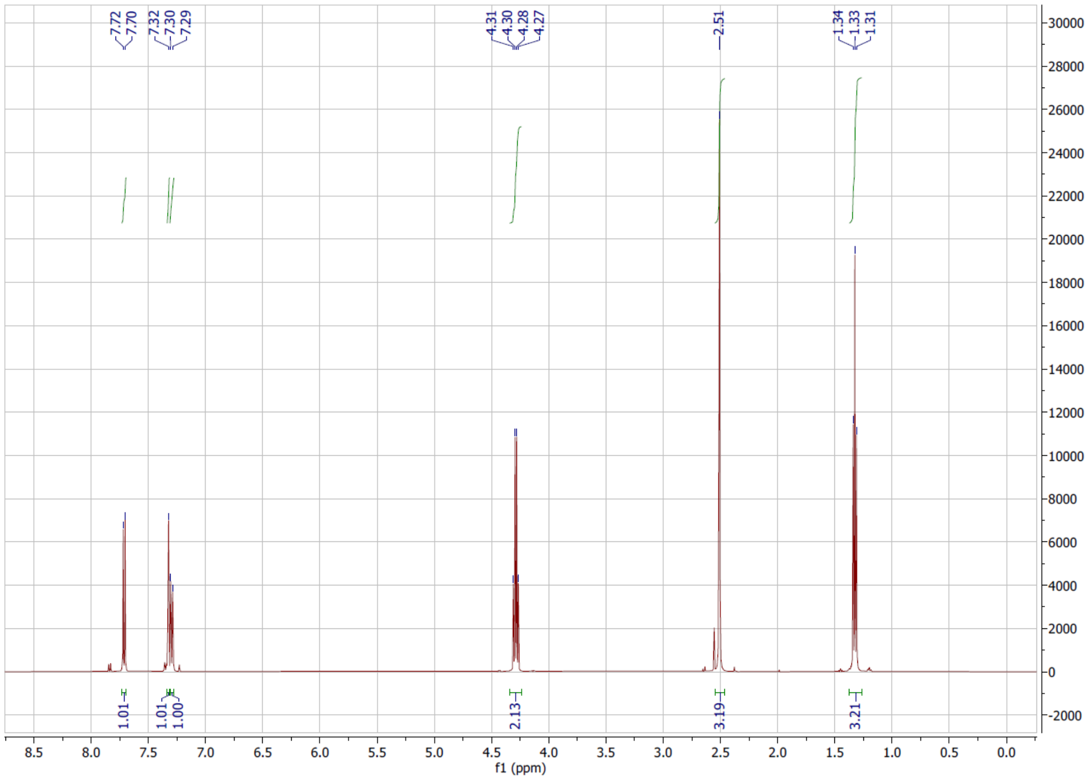

^{13}C-NMR (DMSO-d$_6$):

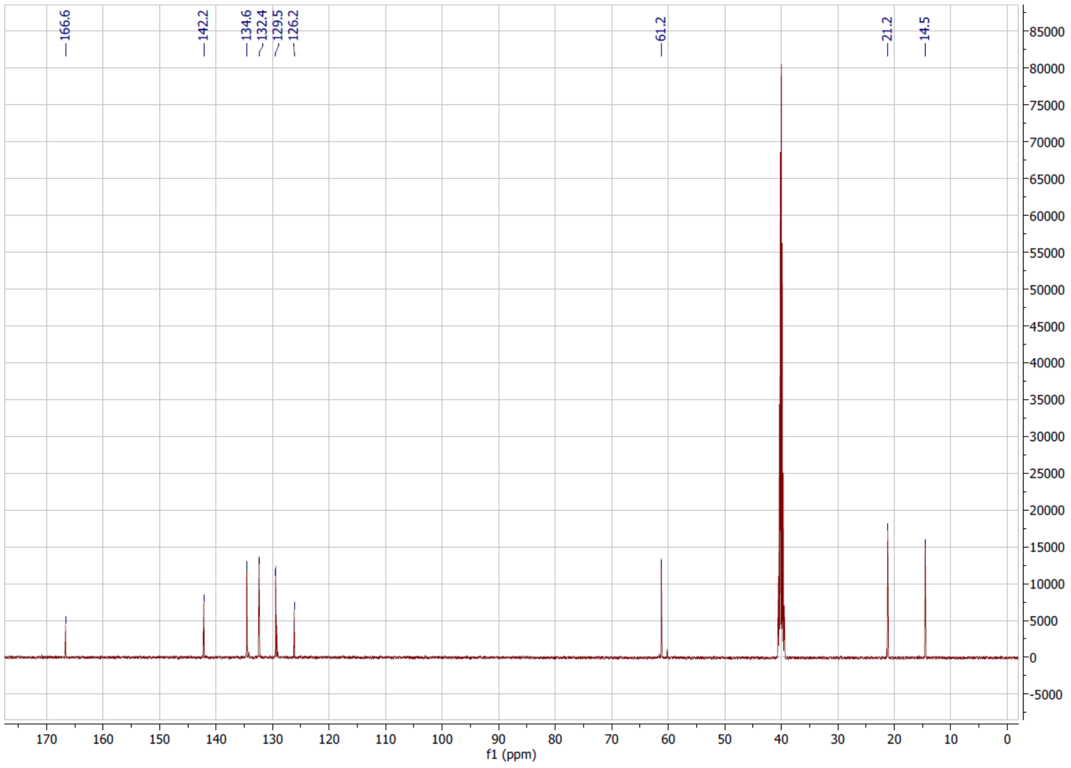

Methyl 2-Methyl-4-(1H-tetrazol-5-yl)benzoate, **70**

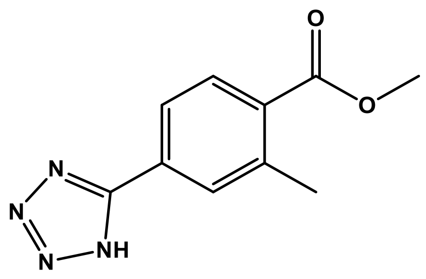

^{1}H-NMR ($CDCl_3$):

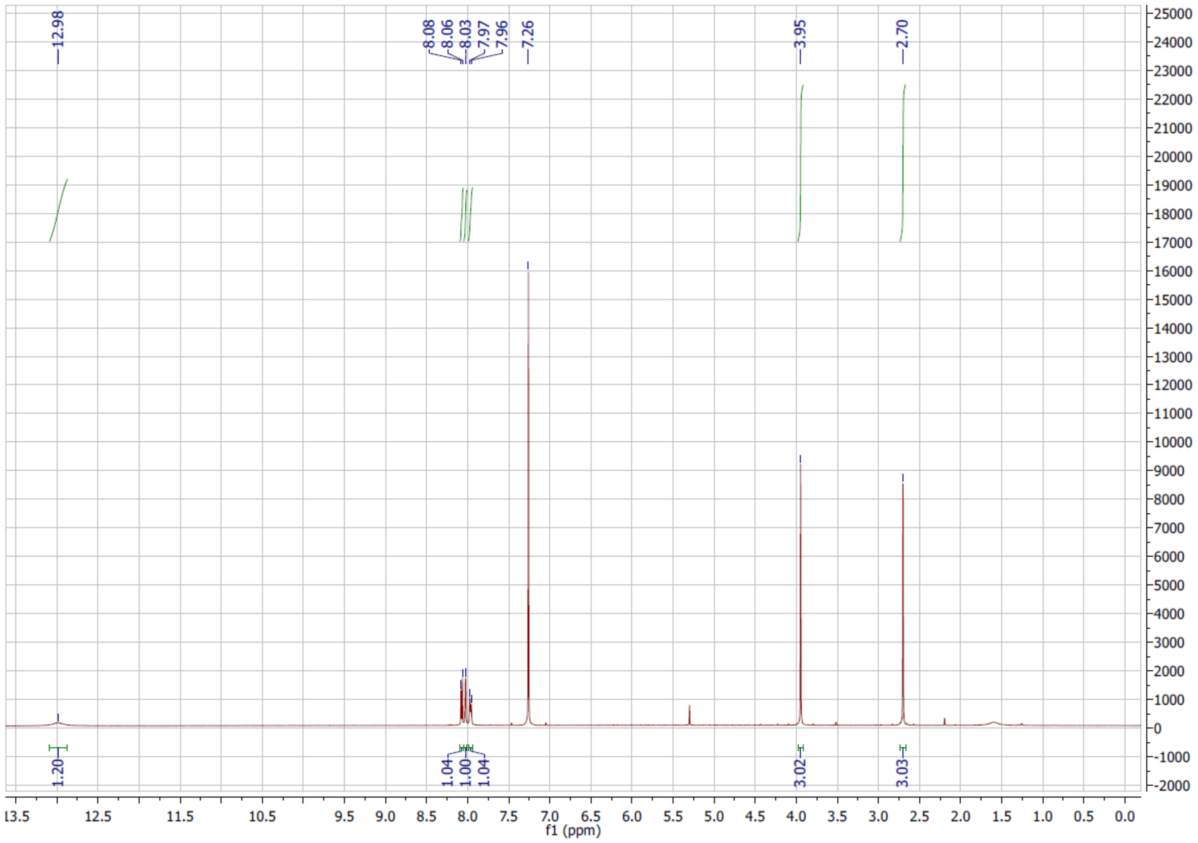

12.98
8.08
8.06
8.03
7.97
7.96
7.26
3.95
2.70
1.20
1.04
1.00
1.04
3.02
3.03
f1 (ppm)

2,4-Dihydroxybenzohydrazide, **4a**

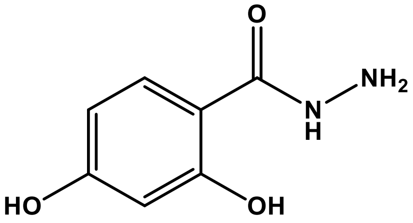

^{1}H-NMR (DMSO-d6):

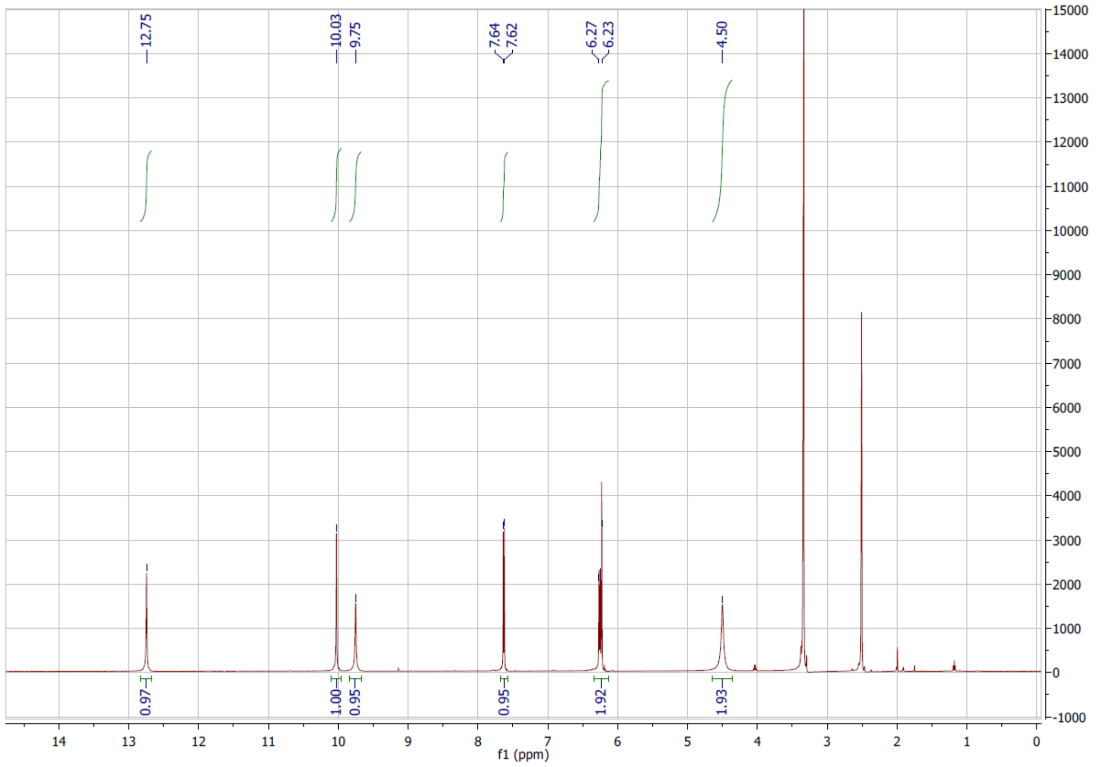

^{13}C-NMR (DMSO-d6):

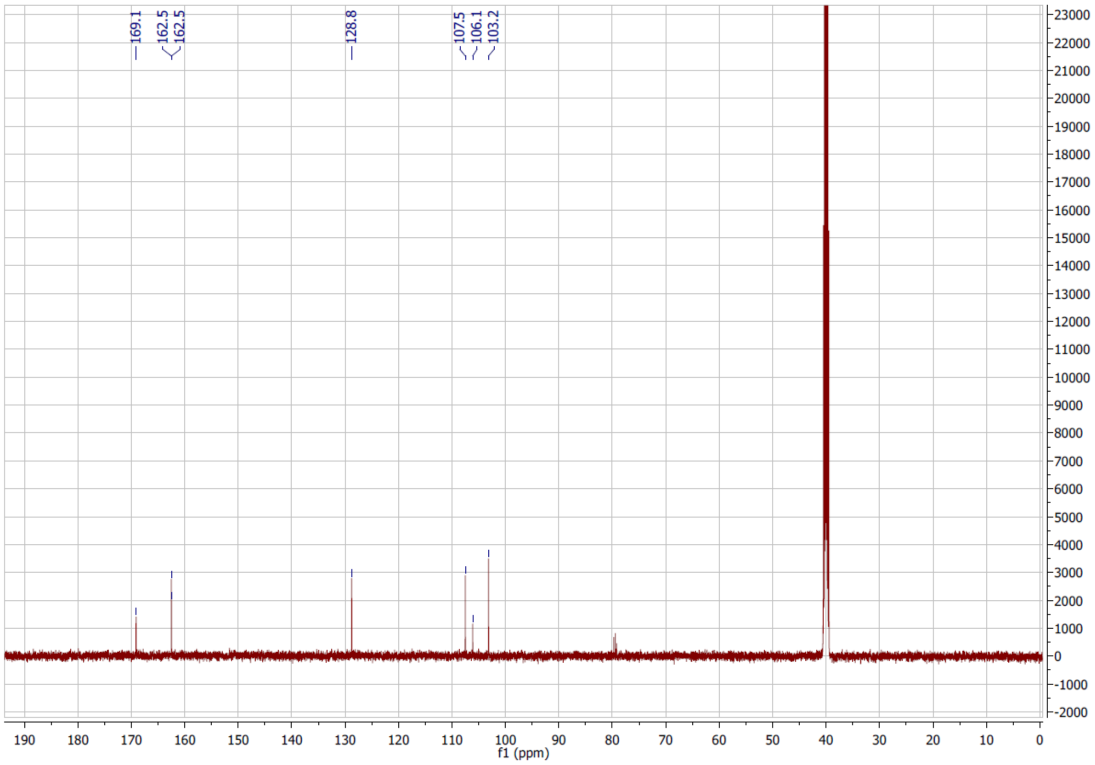

2,4-Dimethylbenzohydrazide, **4c**

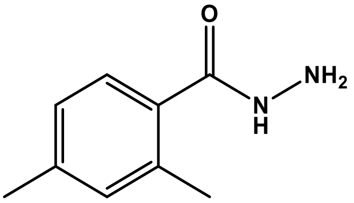

^{1}H-NMR (DMSO-d$_6$):

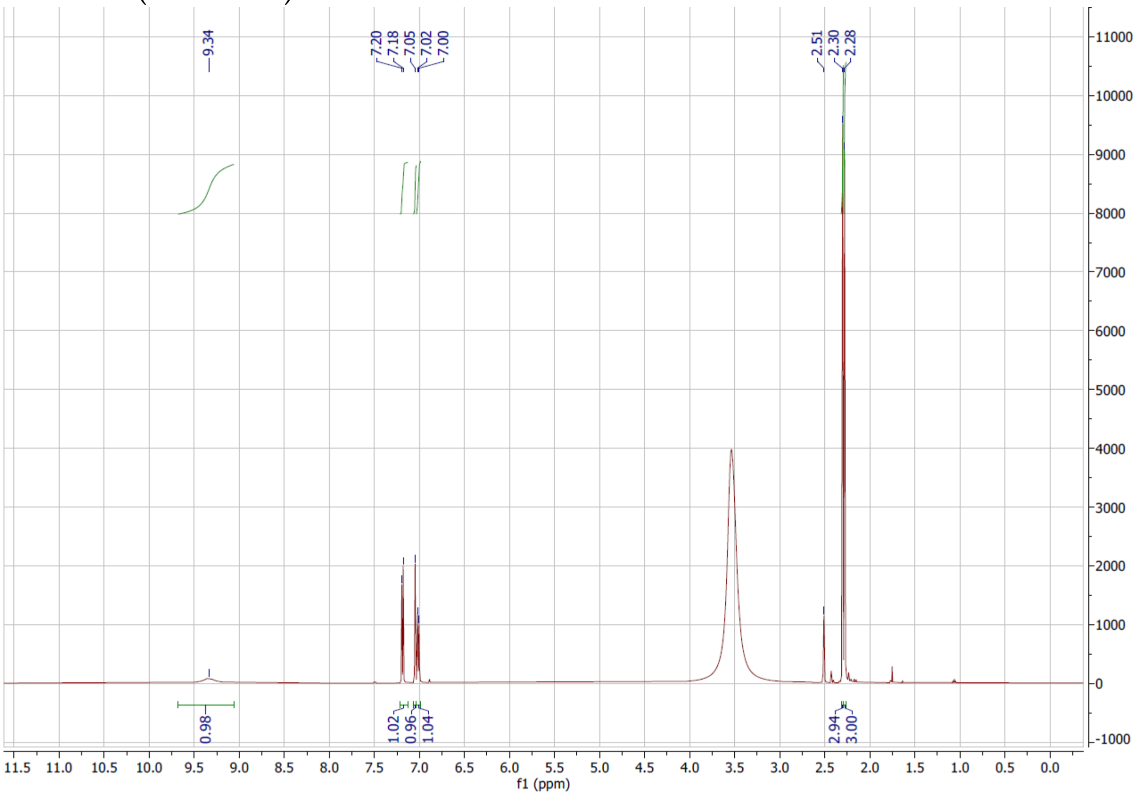

^{13}C-NMR (DMSO-d$_6$):

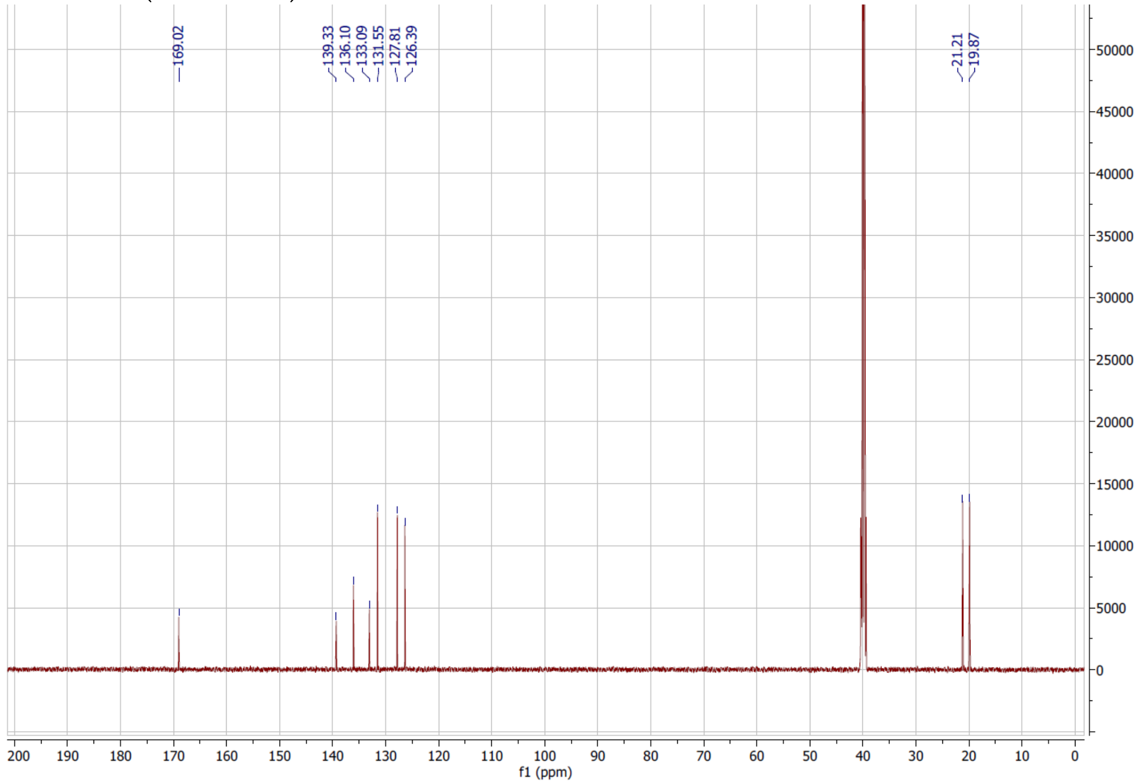

2,4-Dichlorobenzohydrazide, **4h**

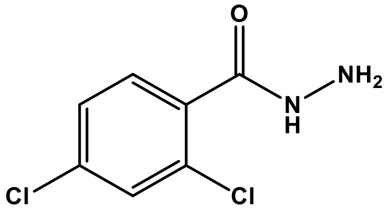

^{1}H-NMR (DMSO-d6):

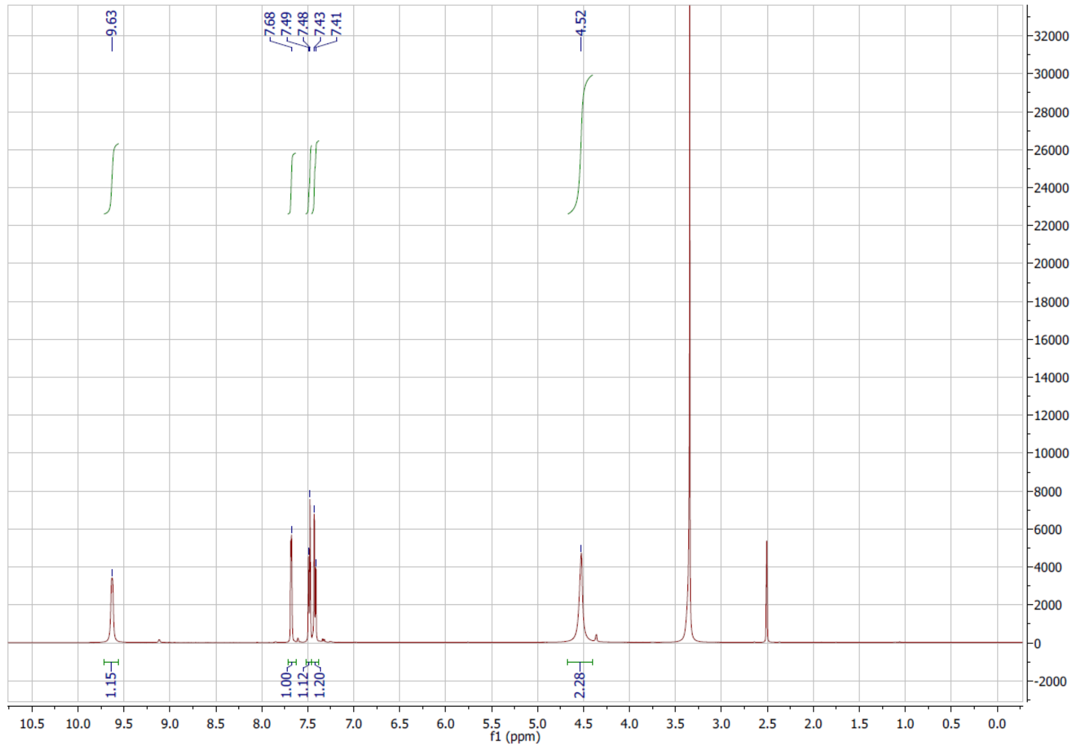

^{13}C-NMR (DMSO-d6):

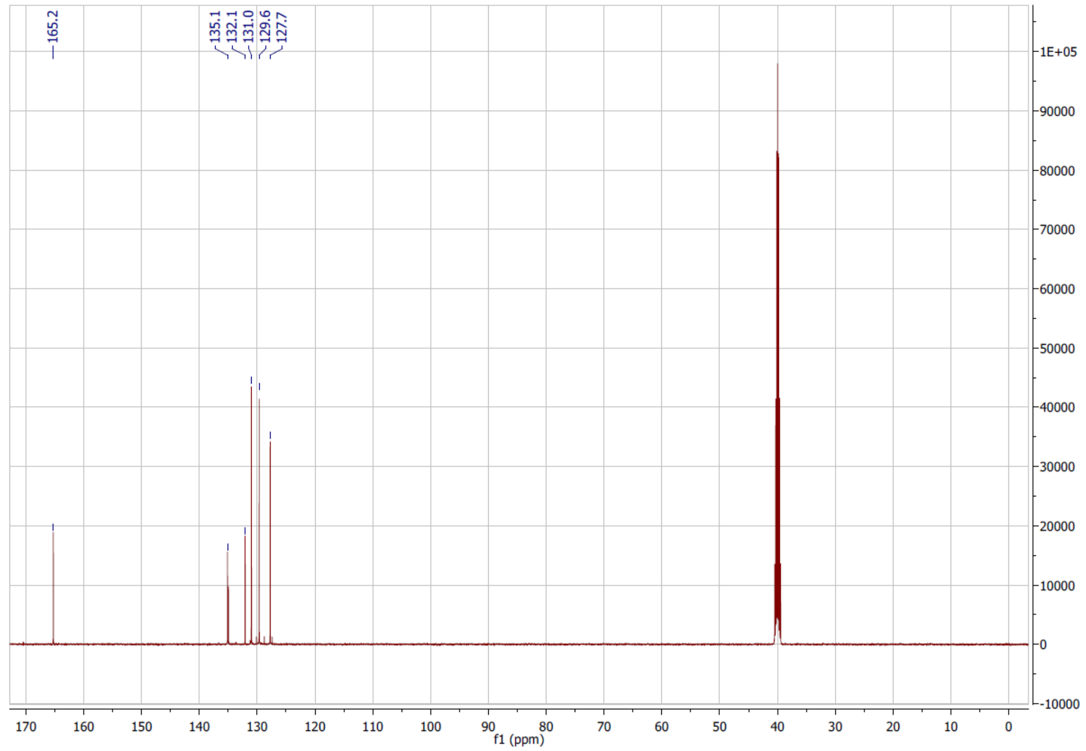

4-Methyl-2-hydroxybenzohydrazide, **4u**

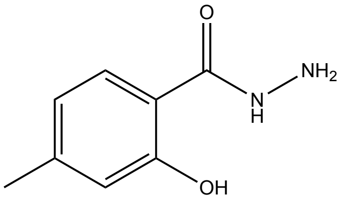

^{1}H-NMR (DMSO-d$_6$):

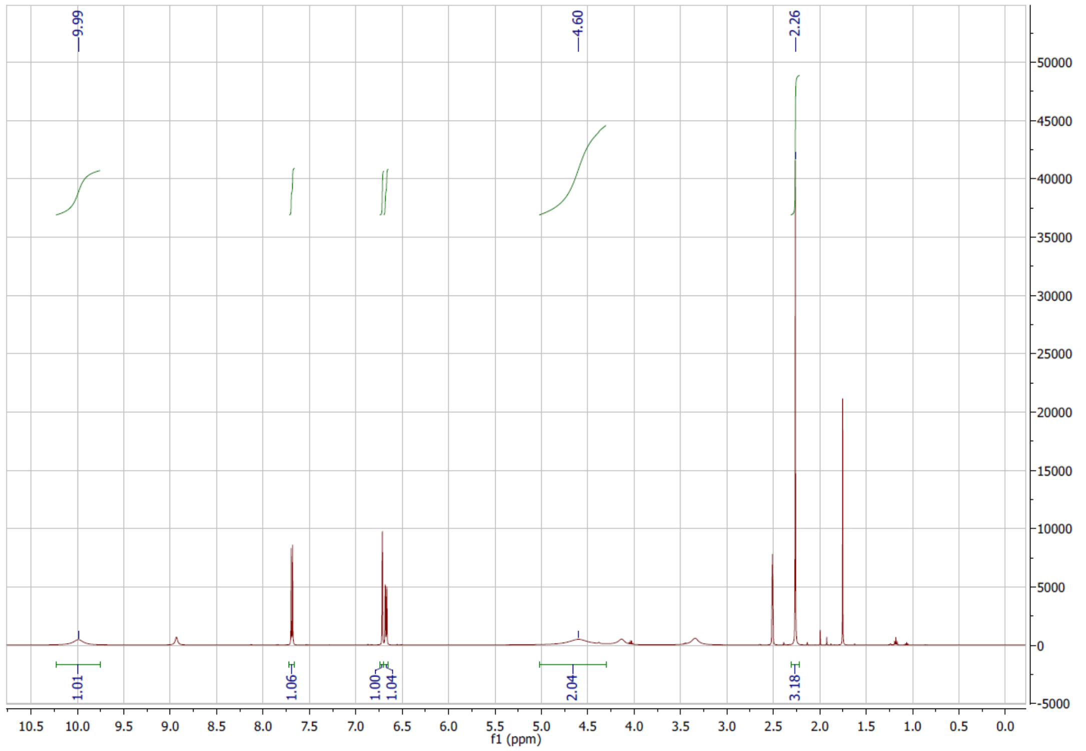

^{13}C-NMR (DMSO-d$_6$):

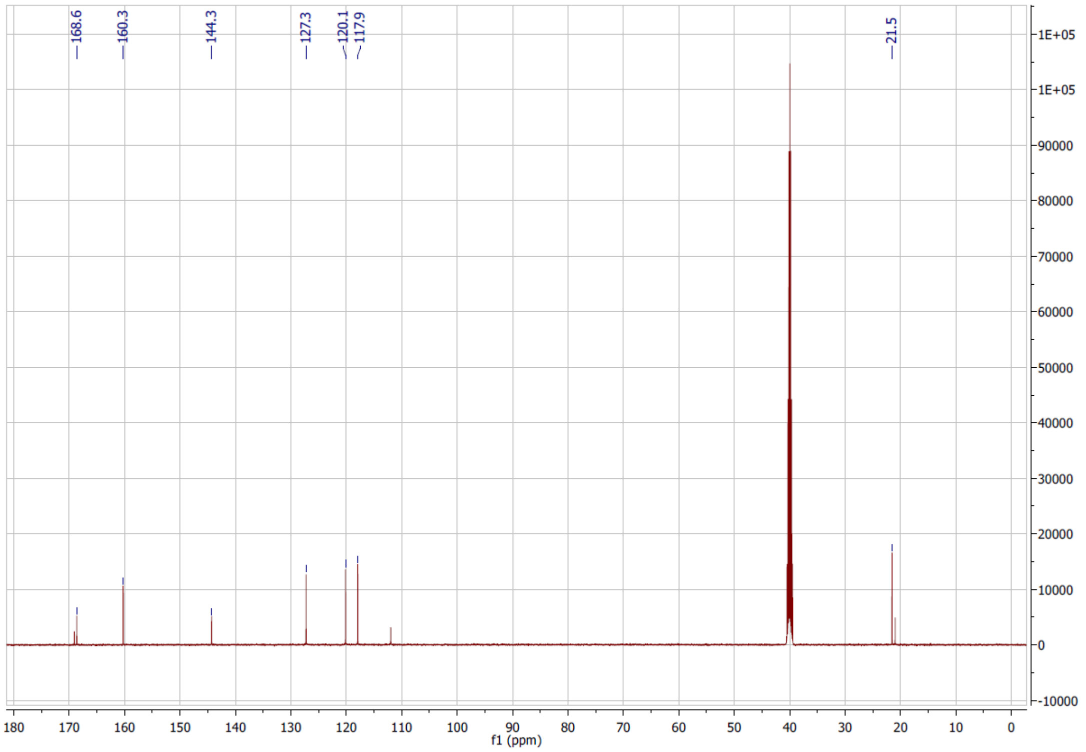

2-Methyl-4-(1H-tetrazol-5-yl)benzohydrazide, **72**

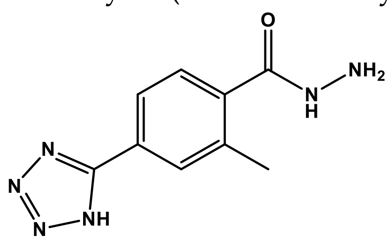

^{1}H-NMR (DMSO-d$_6$):

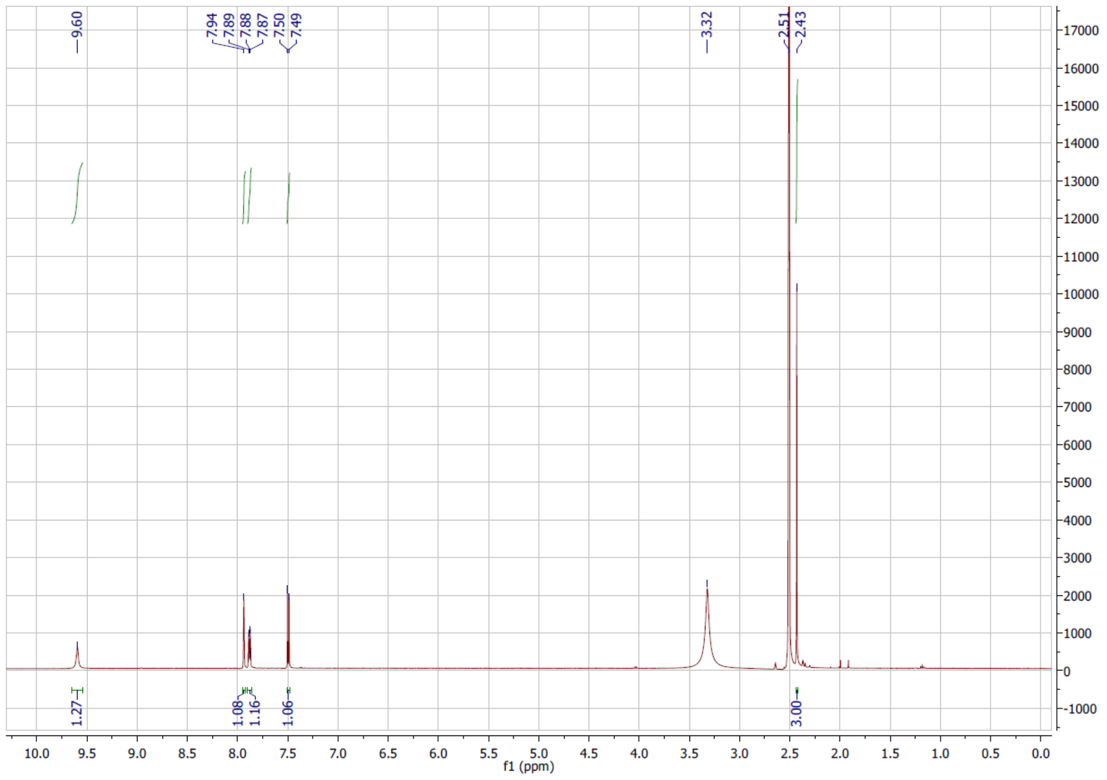

^{13}C-NMR (DMSO-d$_6$):

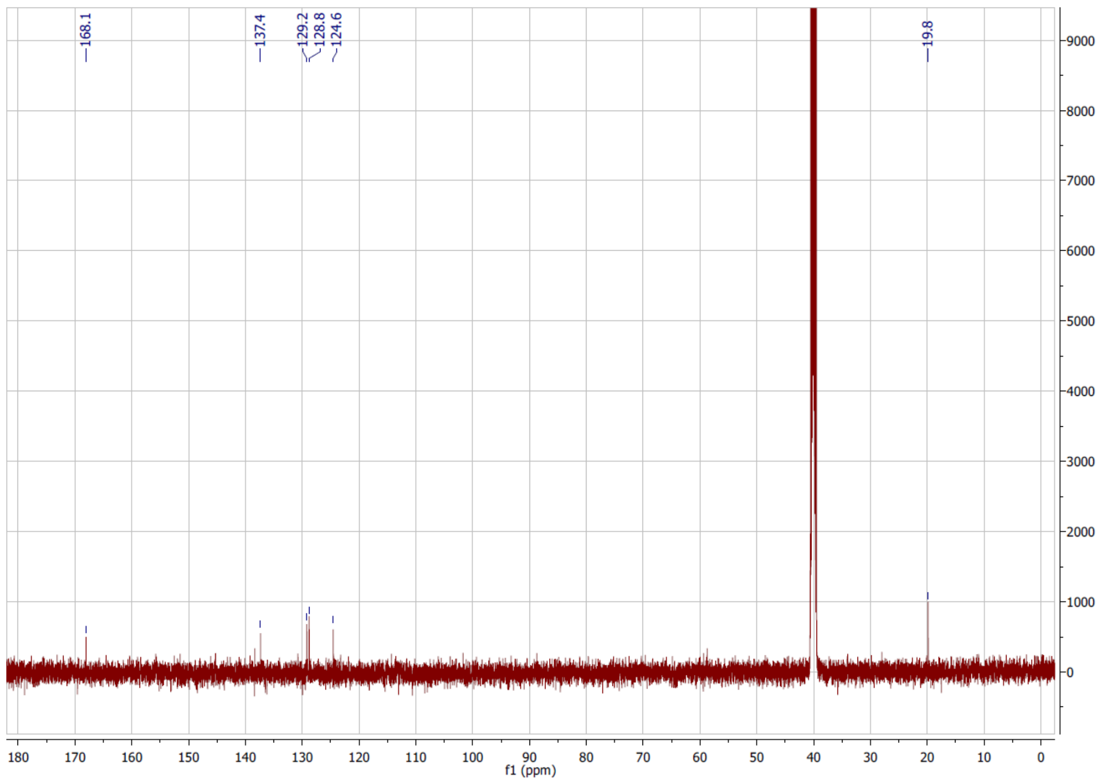

2,4-Dimethyl-*N′*-(naphthalen-1-ylmethylene)benzohydrazide, **1c**

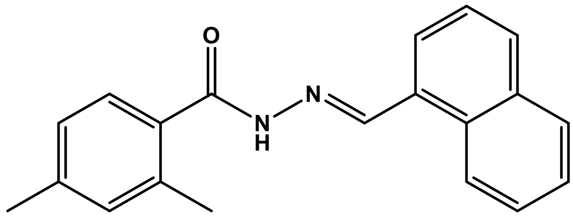

^{1}H-NMR (DMSO-d$_6$):

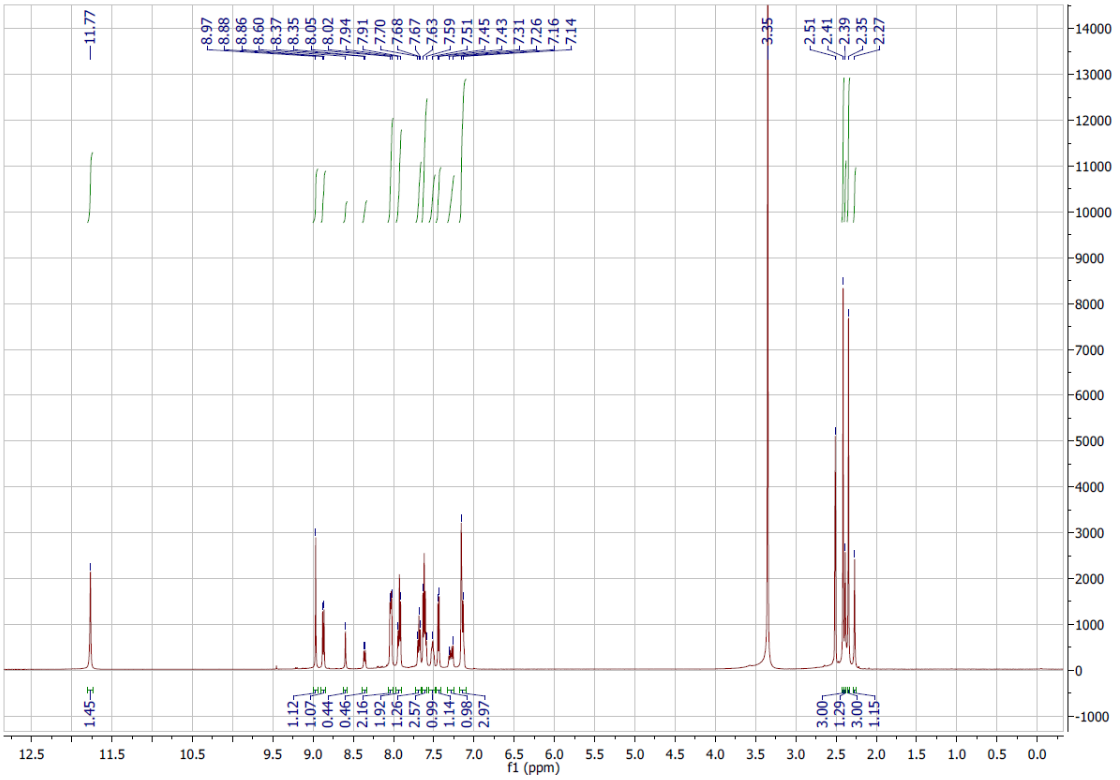

^{13}C-NMR (DMSO-d$_6$):

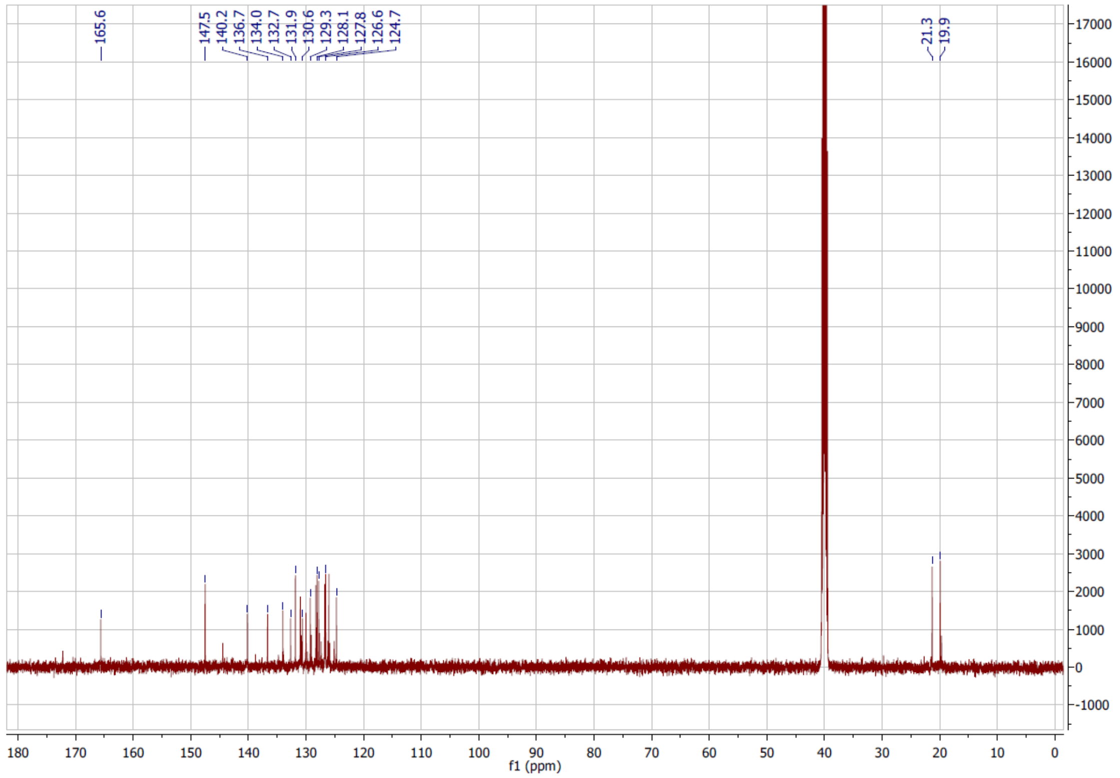

2-Hydroxy-4-methyl-*N′*-(naphthalen-1-ylmethylene)benzohydrazide, **1u**

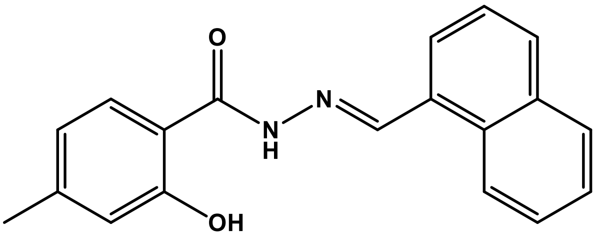

^{1}H-NMR (DMSO-d_6):

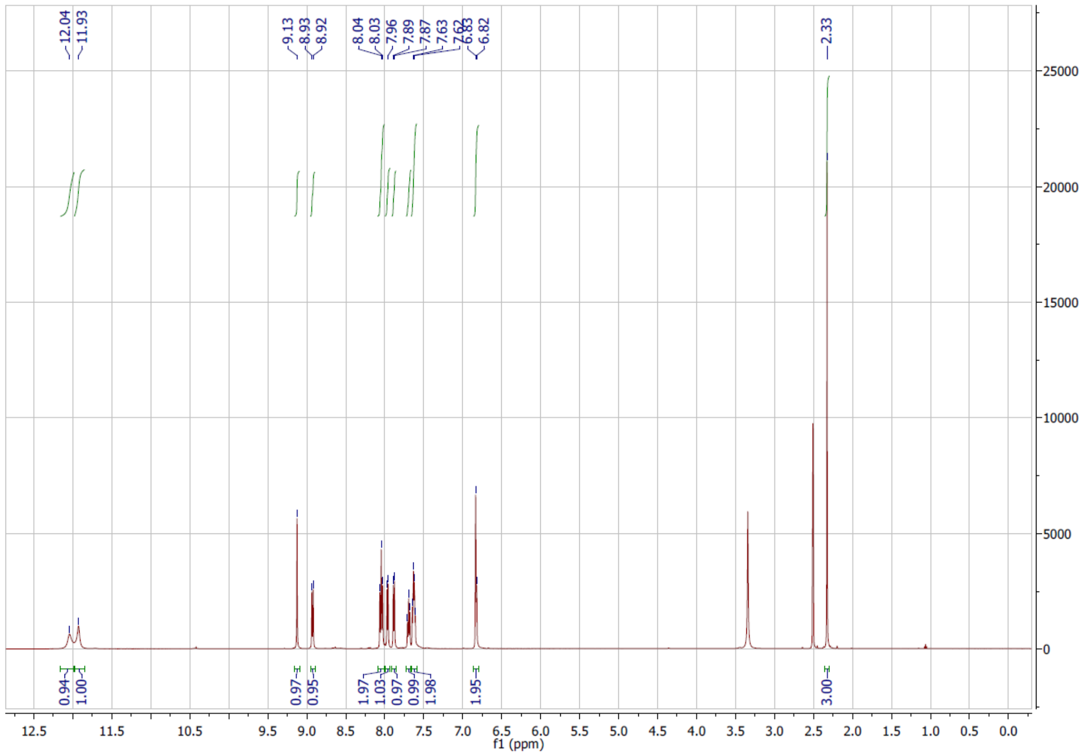

^{13}C-NMR (DMSO-d_6):

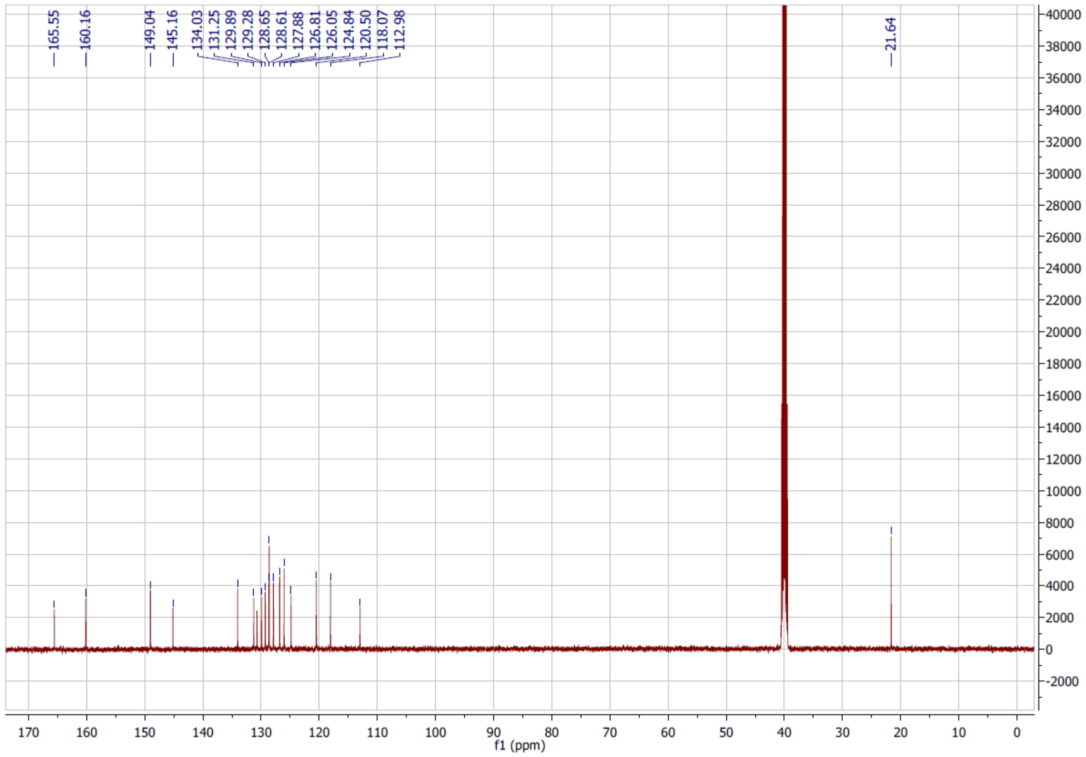

N'-(3,4-Dichlorobenzylidene)-2,4-dihydroxybenzohydrazide, **18a**

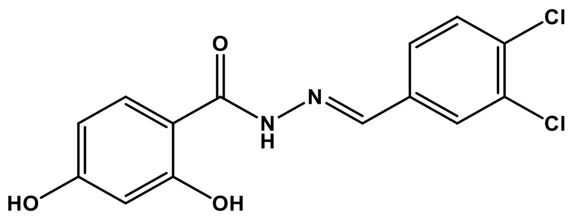

^{1}H-NMR (DMSO-d$_6$):

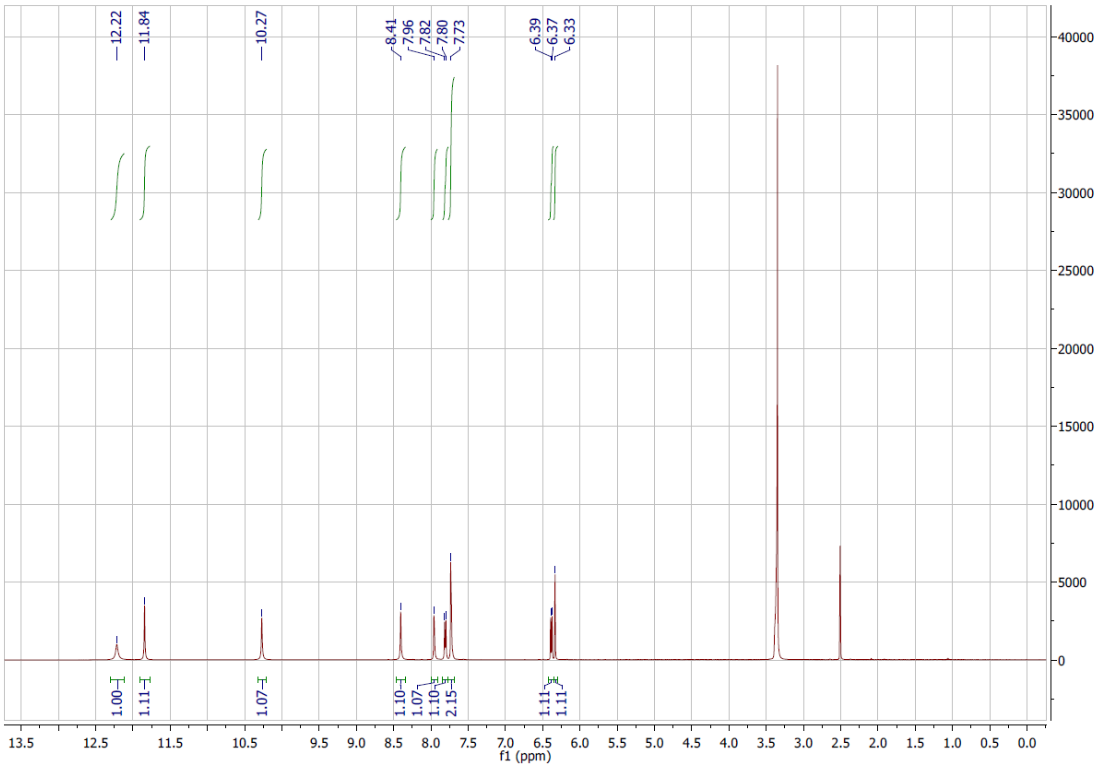

^{13}C-NMR (DMSO-d$_6$):

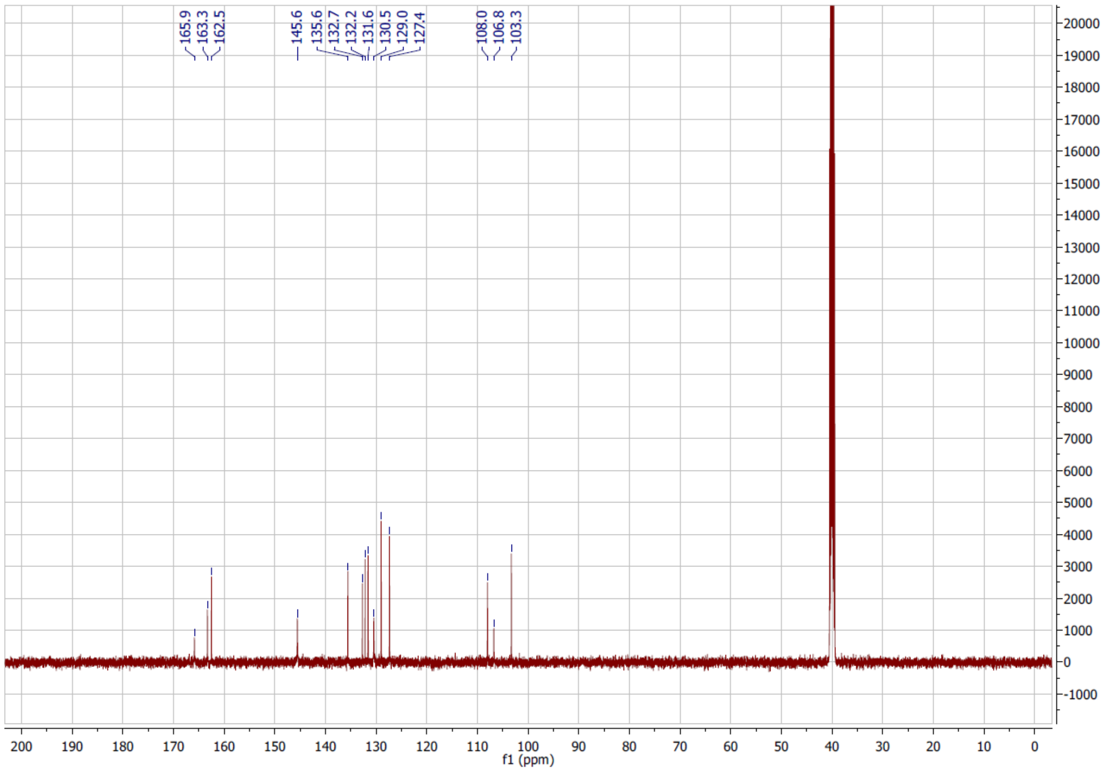

2,4-Dihydroxy-N'-(4-(trifluoromethyl)benzylidene)benzohydrazide, **20a**

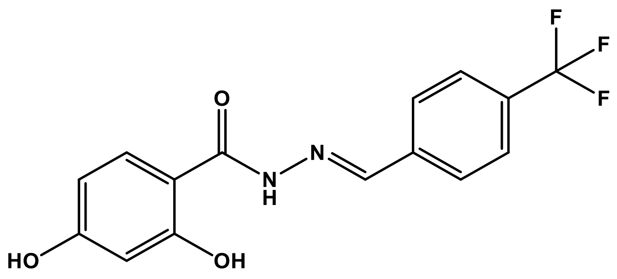

^{1}H-NMR (DMSO-d$_6$):

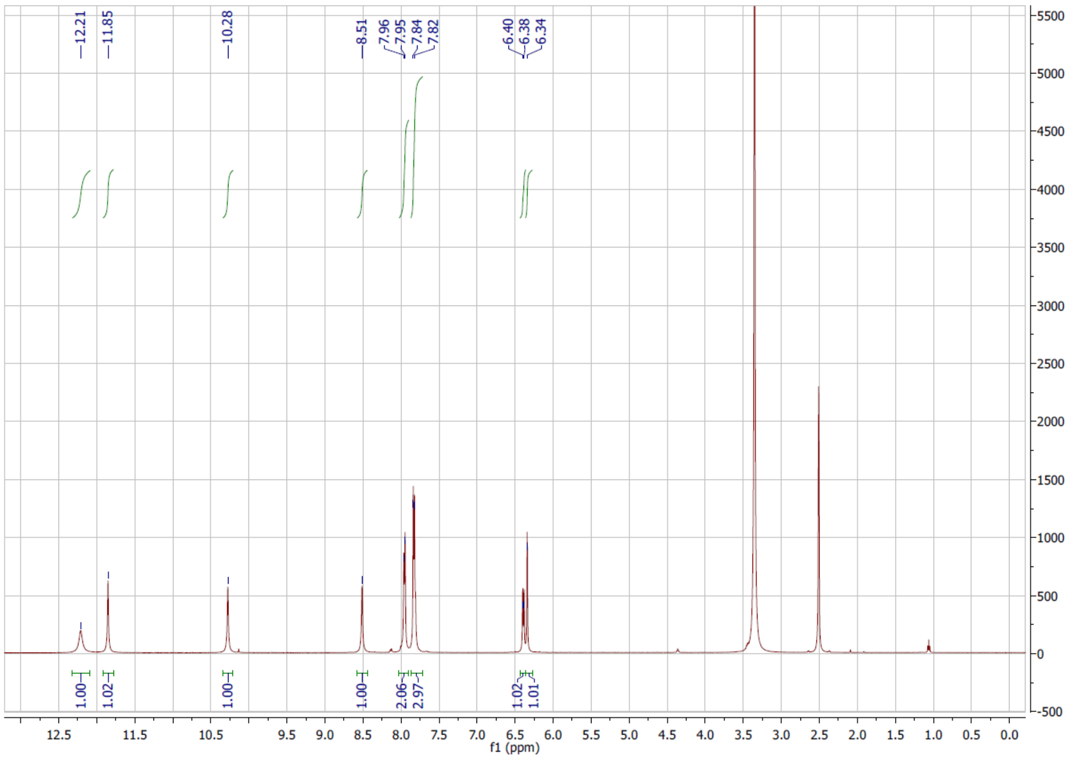

^{13}C-NMR (DMSO-d$_6$):

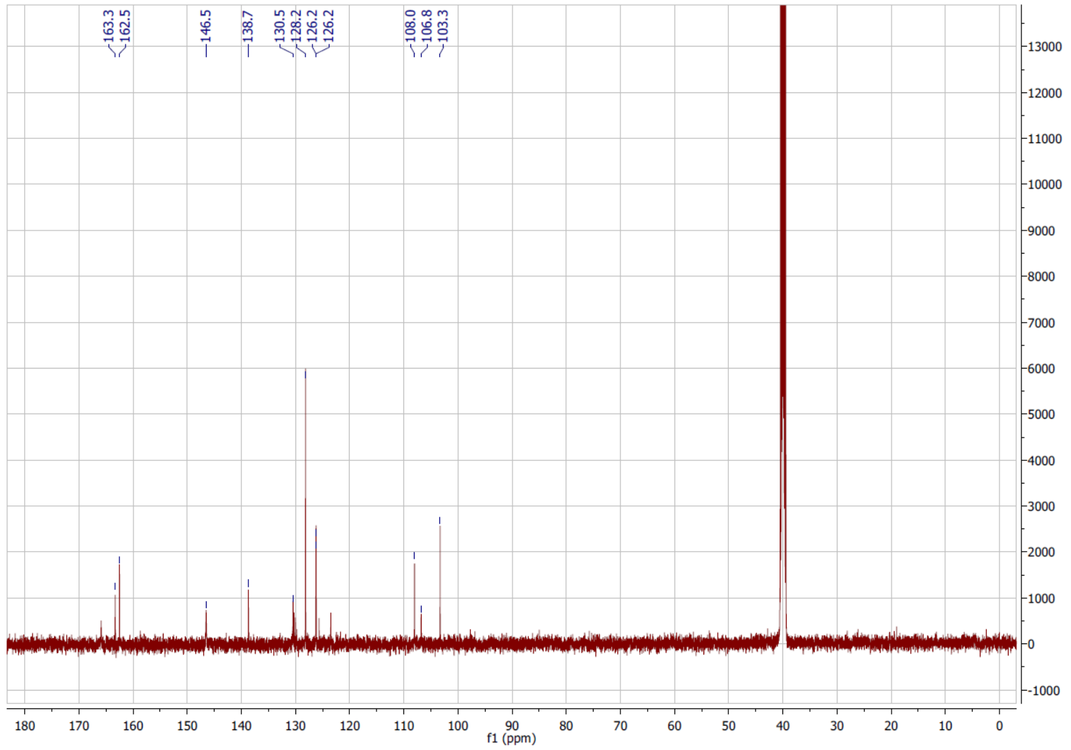

^{19}F-NMR (DMSO-d$_6$):

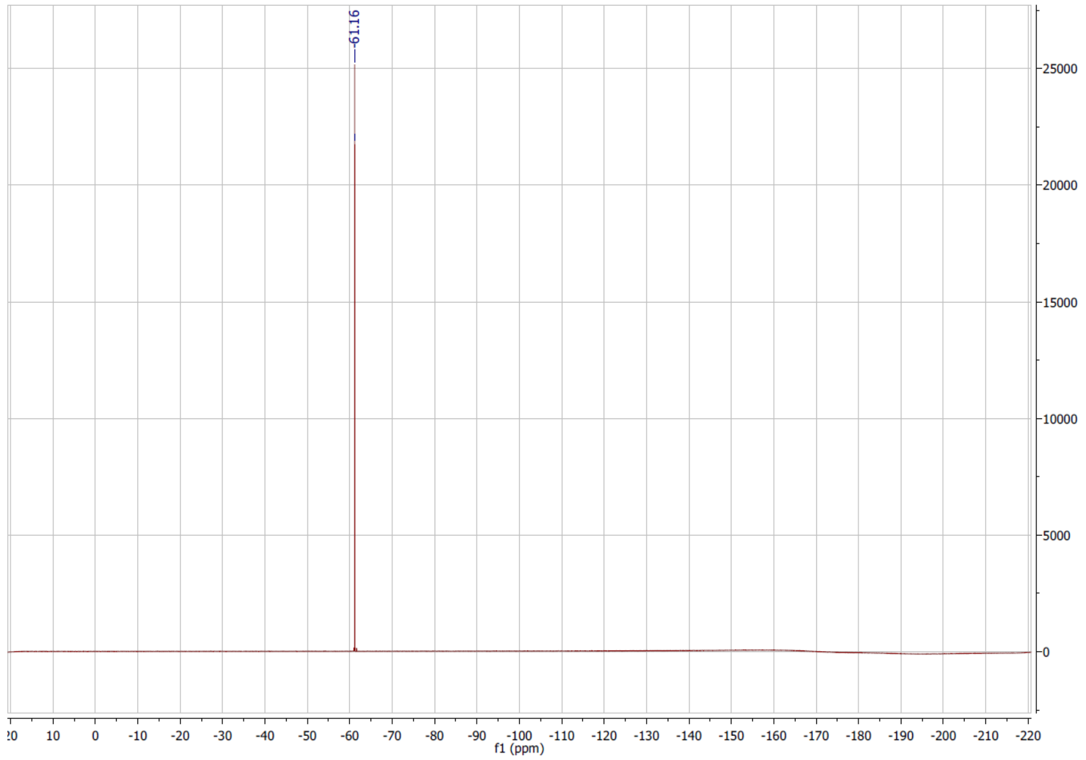

-61.16
25000
20000
15000
10000
5000
0
20
10
0
-10
-20
-30
-40
-50
-60
-70
-80
-90
-100
-110
-120
-130
-140
-150
-160
-170
-180
-190
-200
-210
-220
f1 (ppm)

N′-(4-(*tert*-Butyl)benzylidene)-2,4-dimethylbenzohydrazide, **22f**

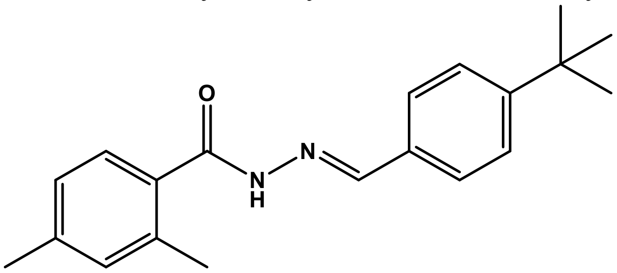

^{1}H-NMR (DMSO-d6):

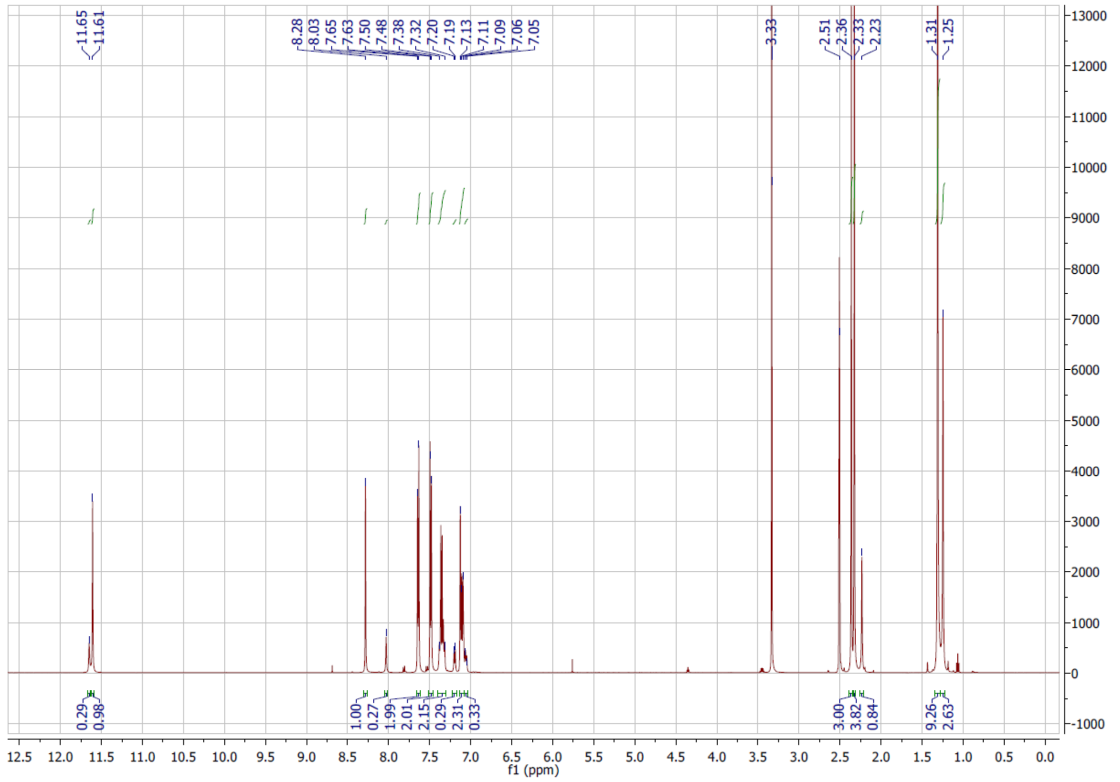

^{13}C-NMR (DMSO-d6):

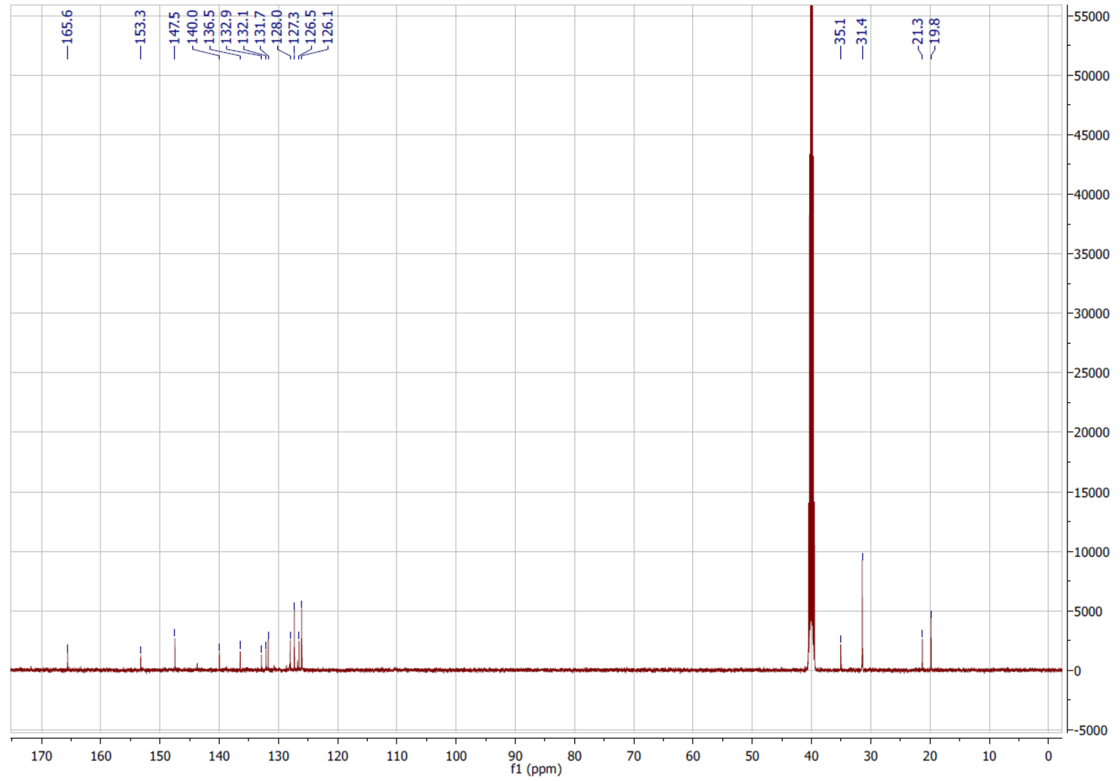

N′-(2,3-Difluorobenzylidene)-2,4-dihydroxybenzohydrazide, **30a**

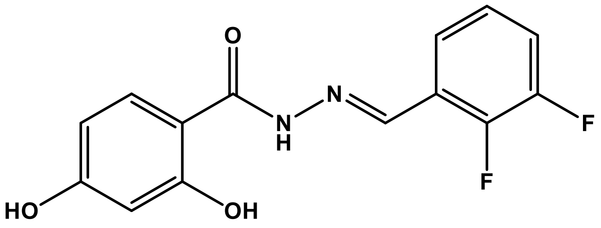

^{1}H-NMR (DMSO-d6):

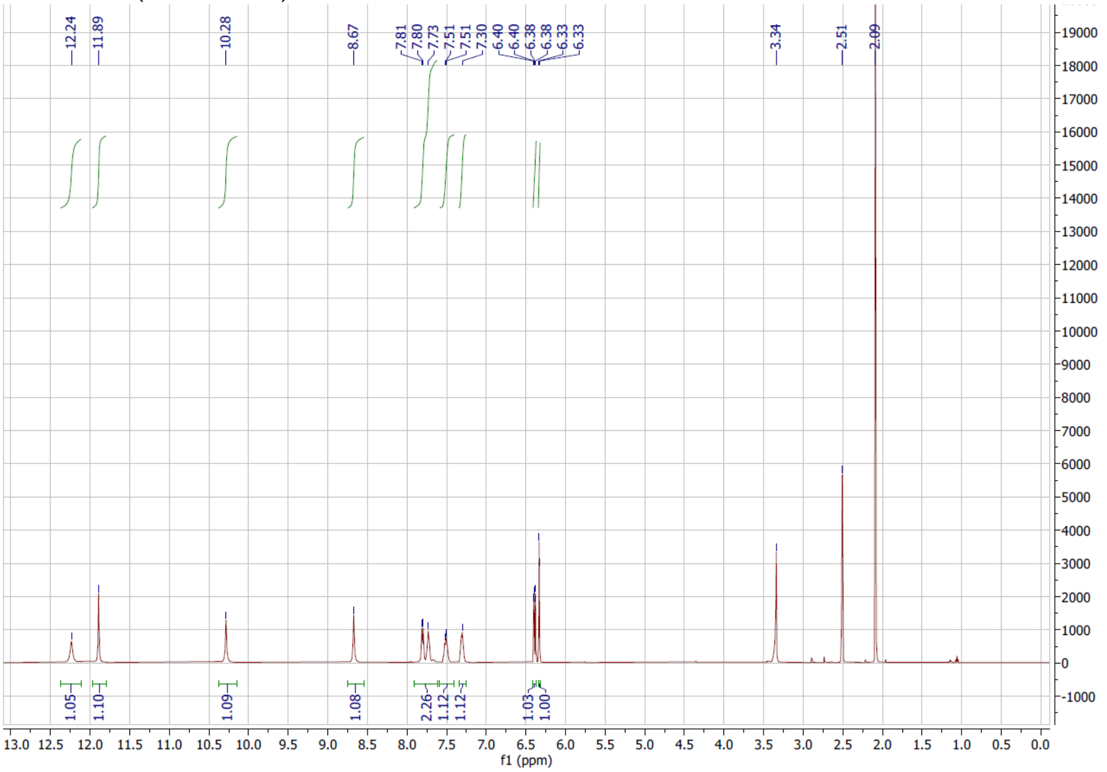

^{13}C-NMR (DMSO-d6):

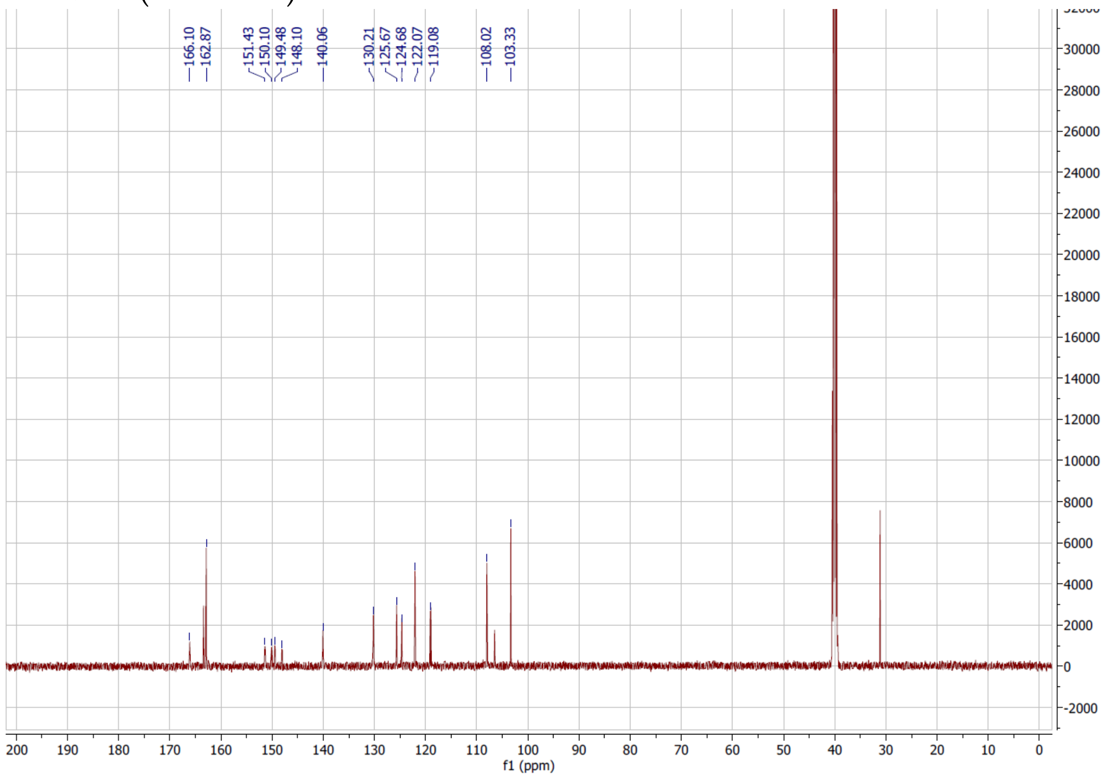

^{19}F-NMR (DMSO-d6):

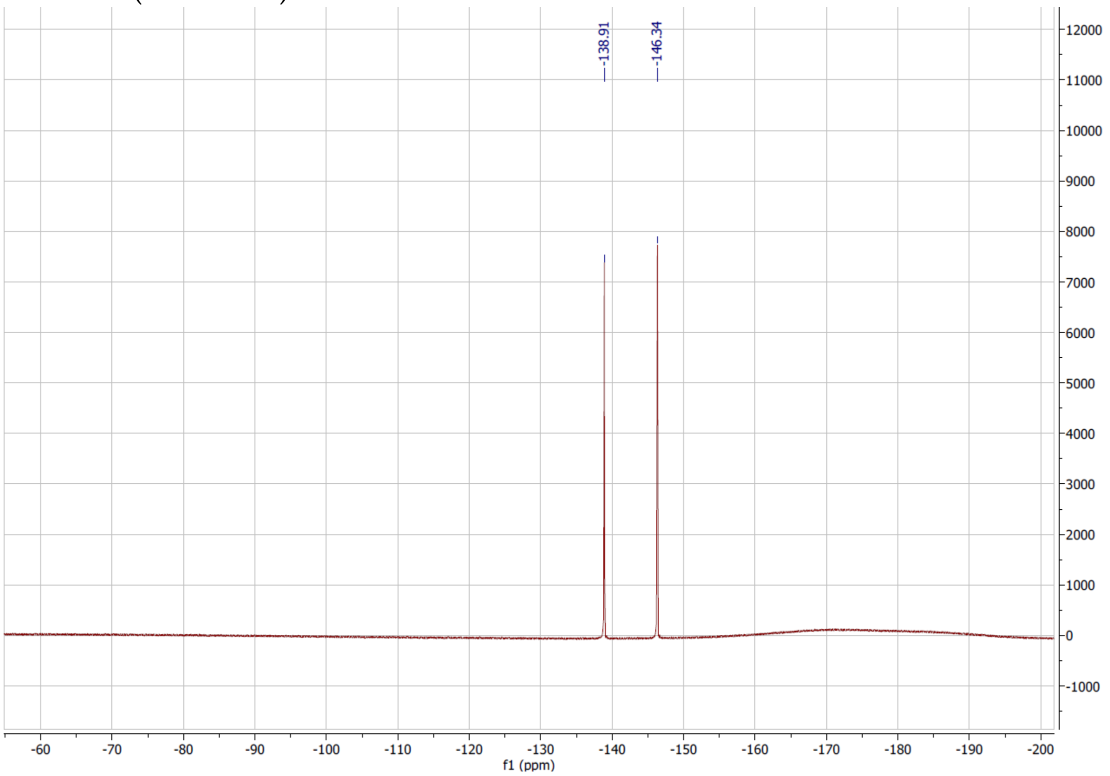

N′-(2,3-Dichlorobenzylidene)-2,4-dimethylbenzohydrazide, **31f**

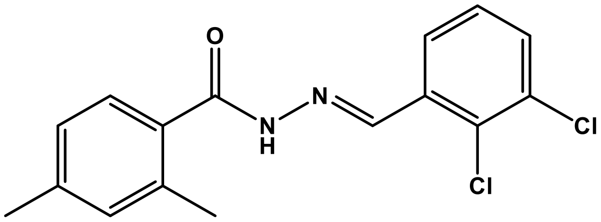

^{1}H-NMR (DMSO-d6):

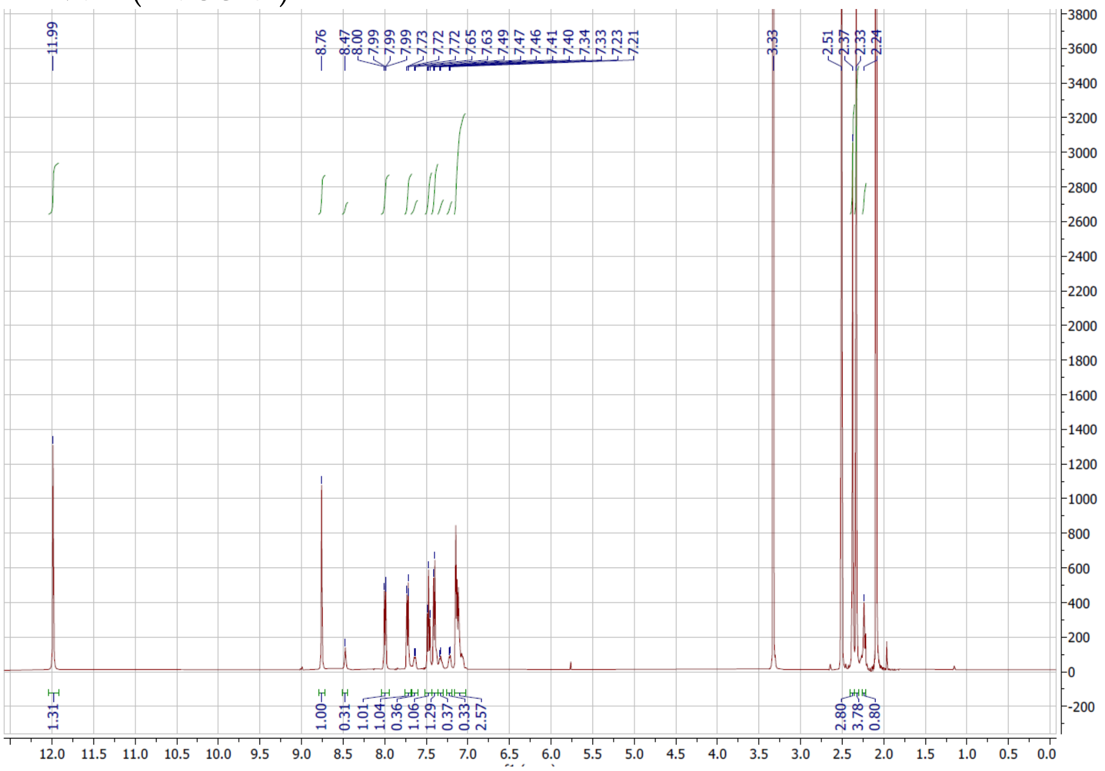

^{13}C-NMR (DMSO-d6):

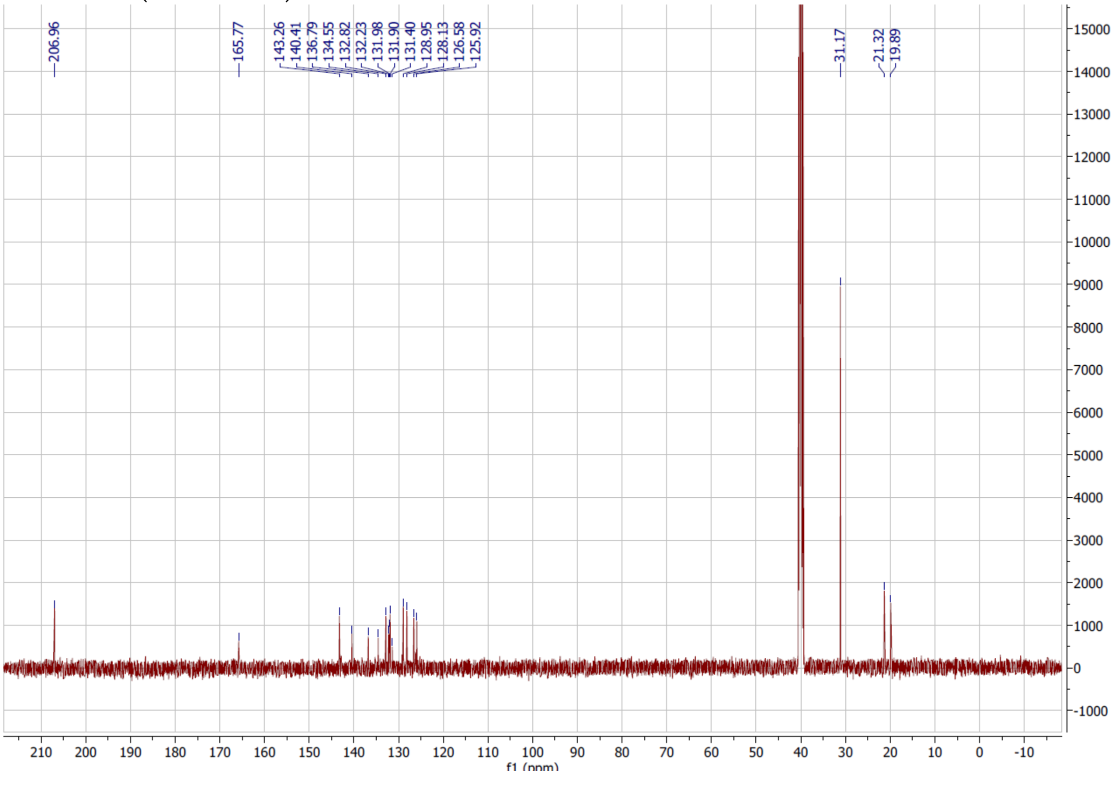

2,4-Dihydroxy-N'-((8-hydroxynaphthalen-1-yl)methylene)benzohydrazide, **43a**

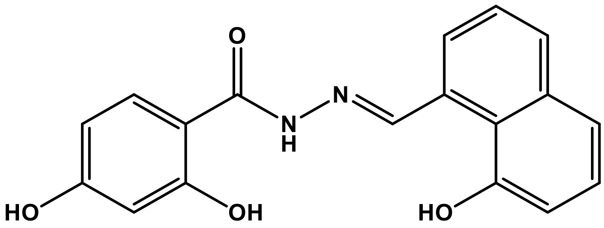

^{1}H-NMR (DMSO-d6):

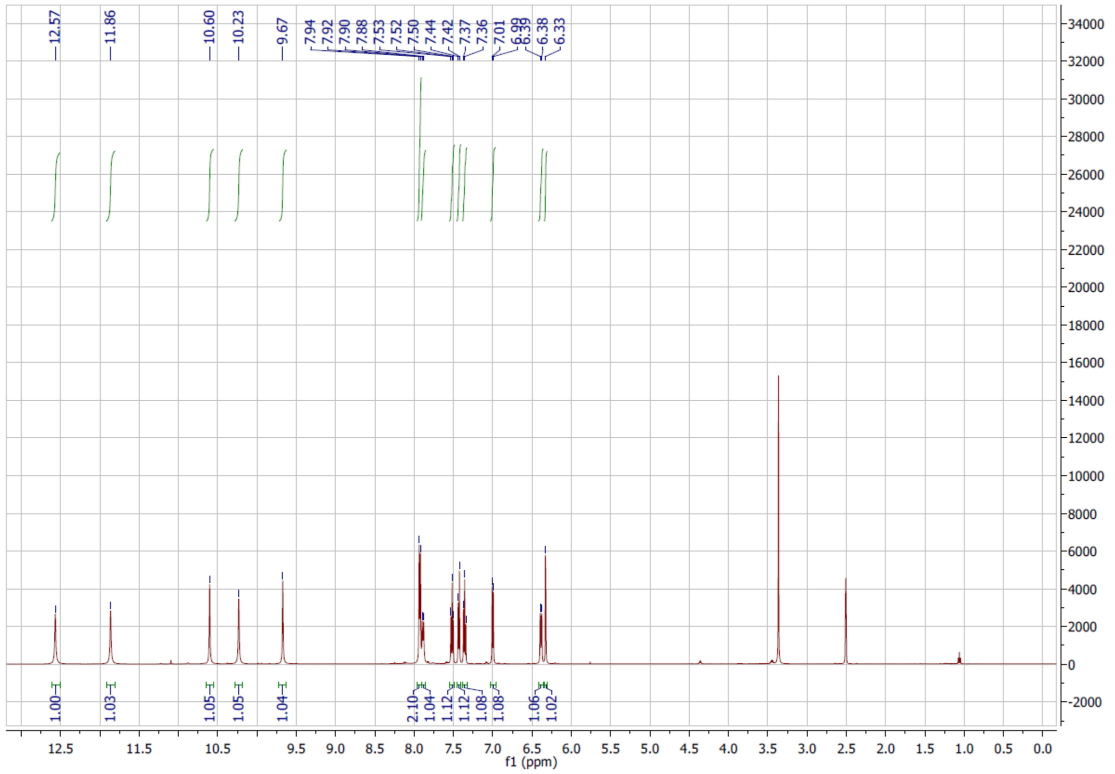

^{13}C-NMR (DMSO-d6):

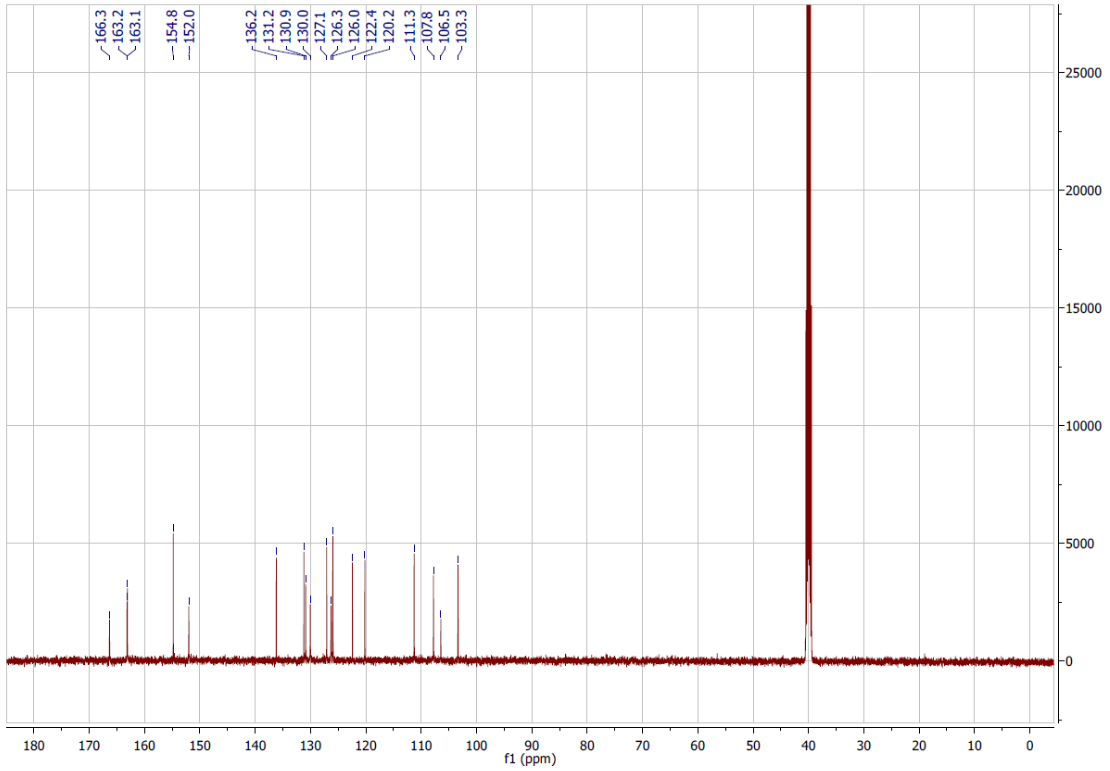

3,4-Dichloro-*N′*-(2,4-dihydroxybenzylidene)benzohydrazide, **46z**

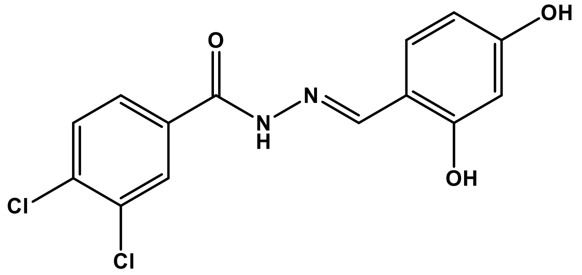

^{1}H-NMR (DMSO-d6):

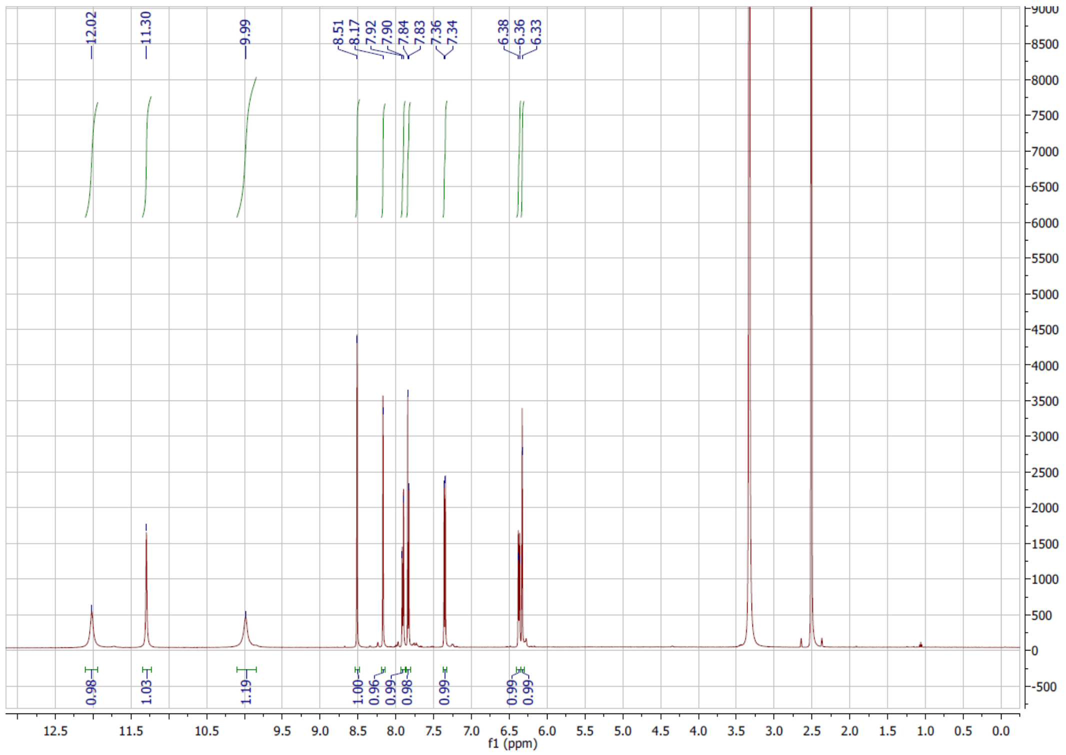

^{13}C-NMR (DMSO-d6):

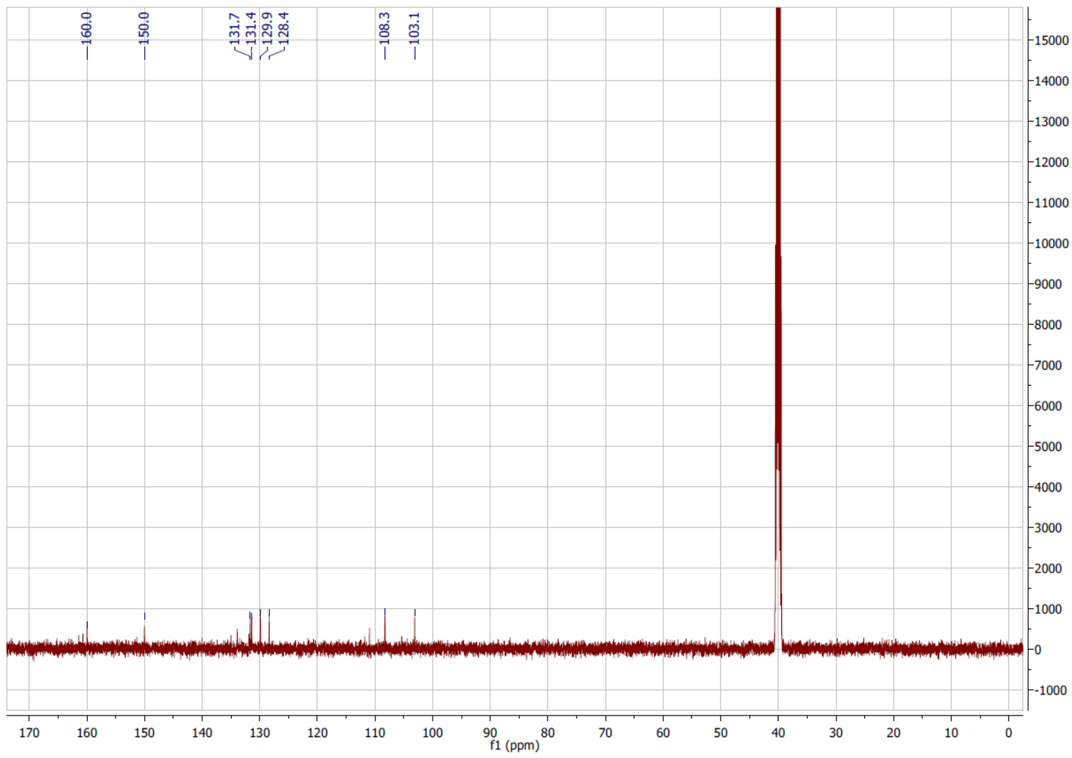

2,4-Dimethyl-*N′*-(naphthalen-1-ylmethyl)benzohydrazide, **47**

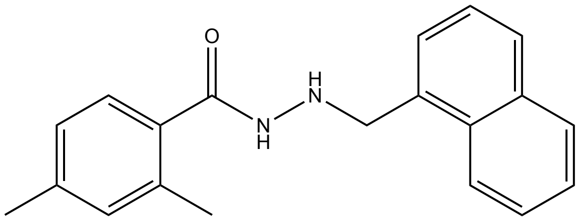

^{1}H-NMR (DMSO-d$_6$):

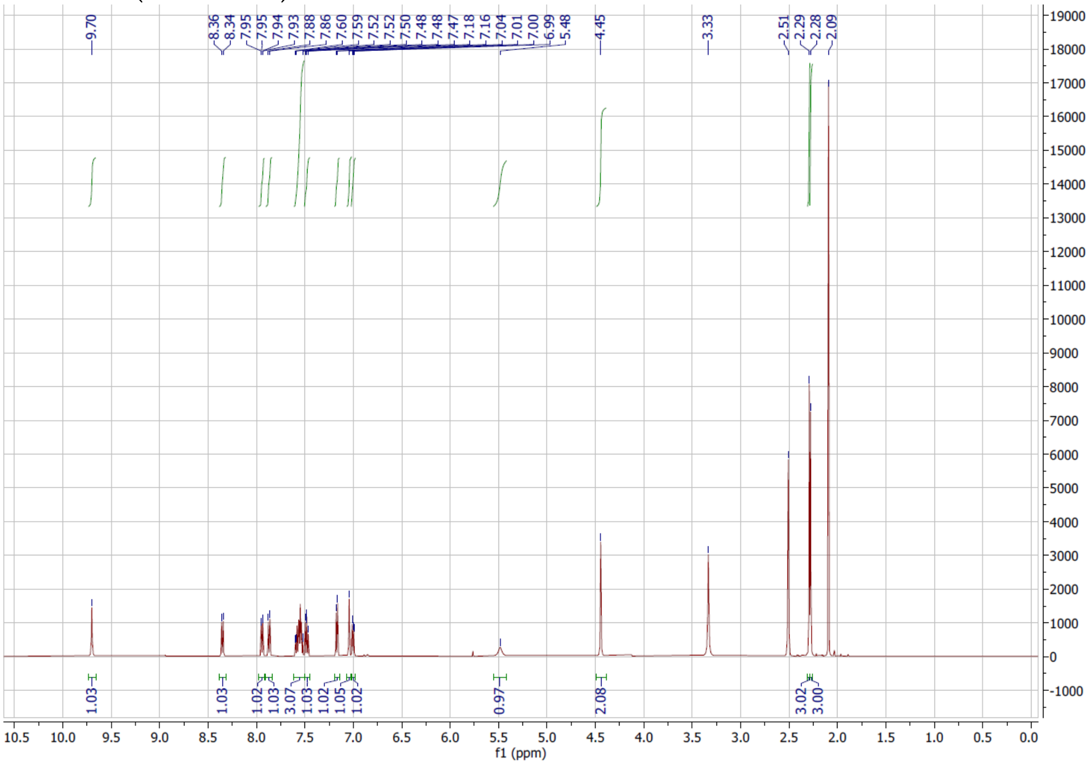

^{13}C-NMR (DMSO-d$_6$):

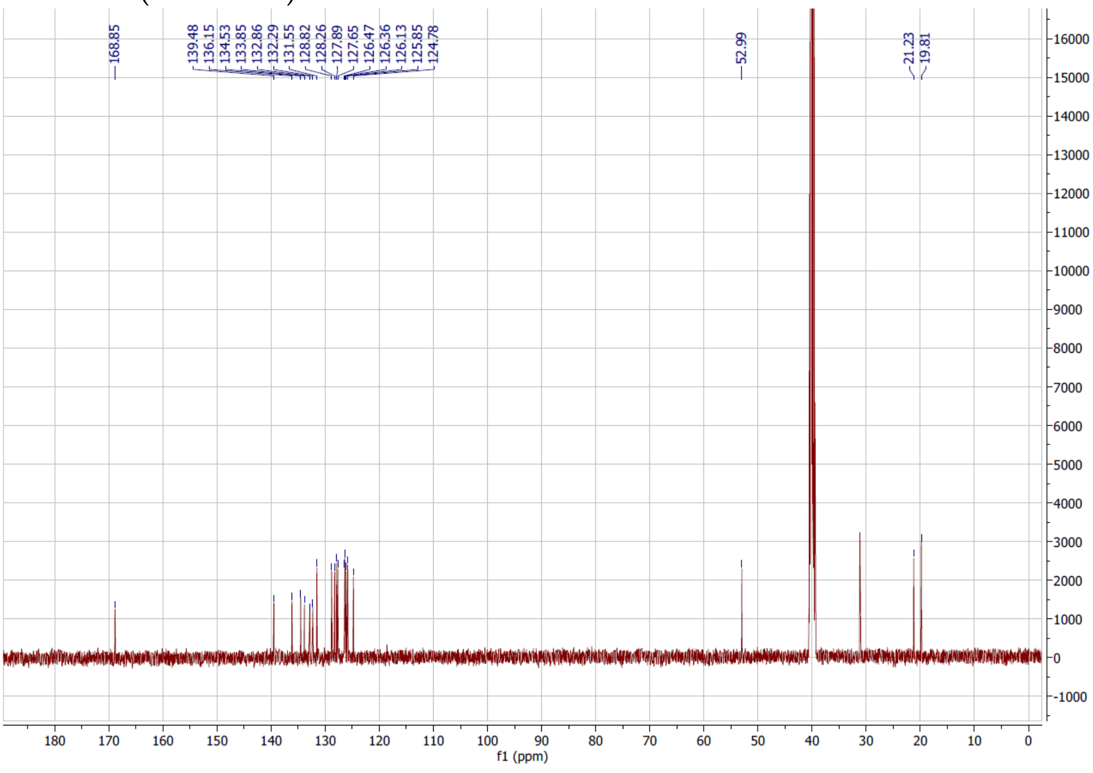

N′-(2,4-Dimethylbenzoyl)-1-naphthohydrazide, **51**

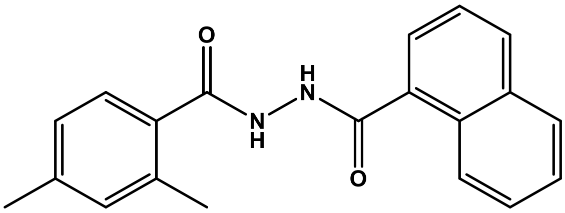

^{1}H-NMR (DMSO-d$_6$):

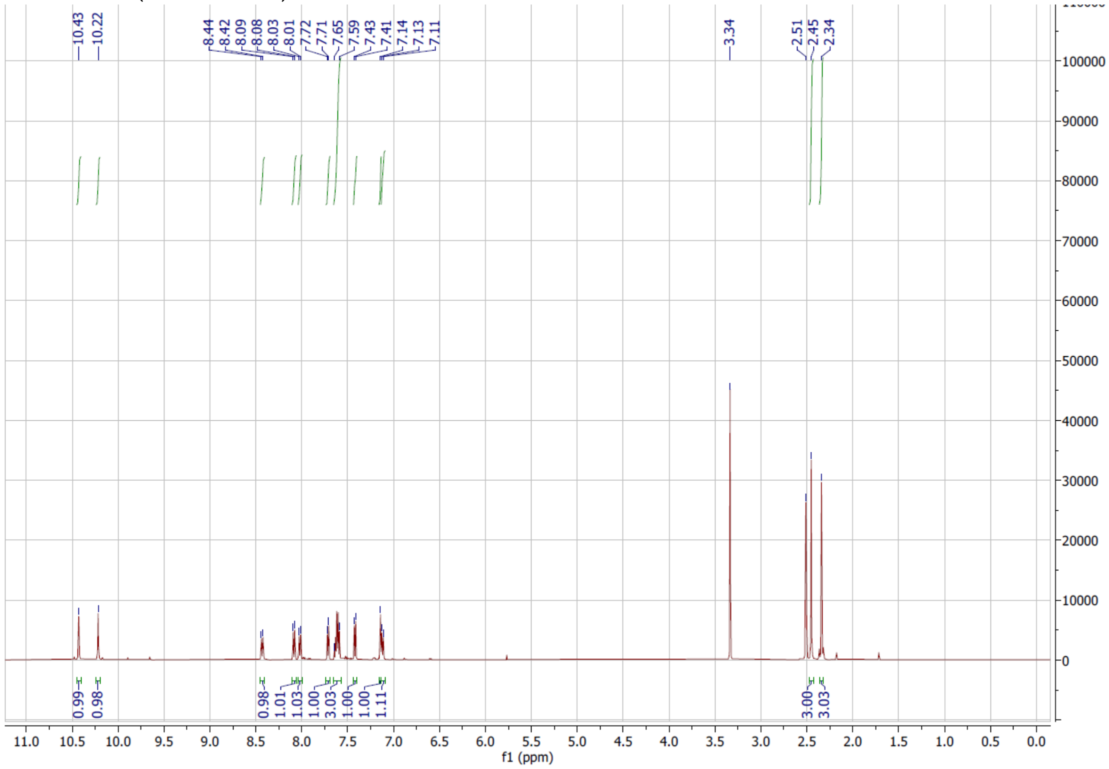

^{13}C-NMR (DMSO-d$_6$):

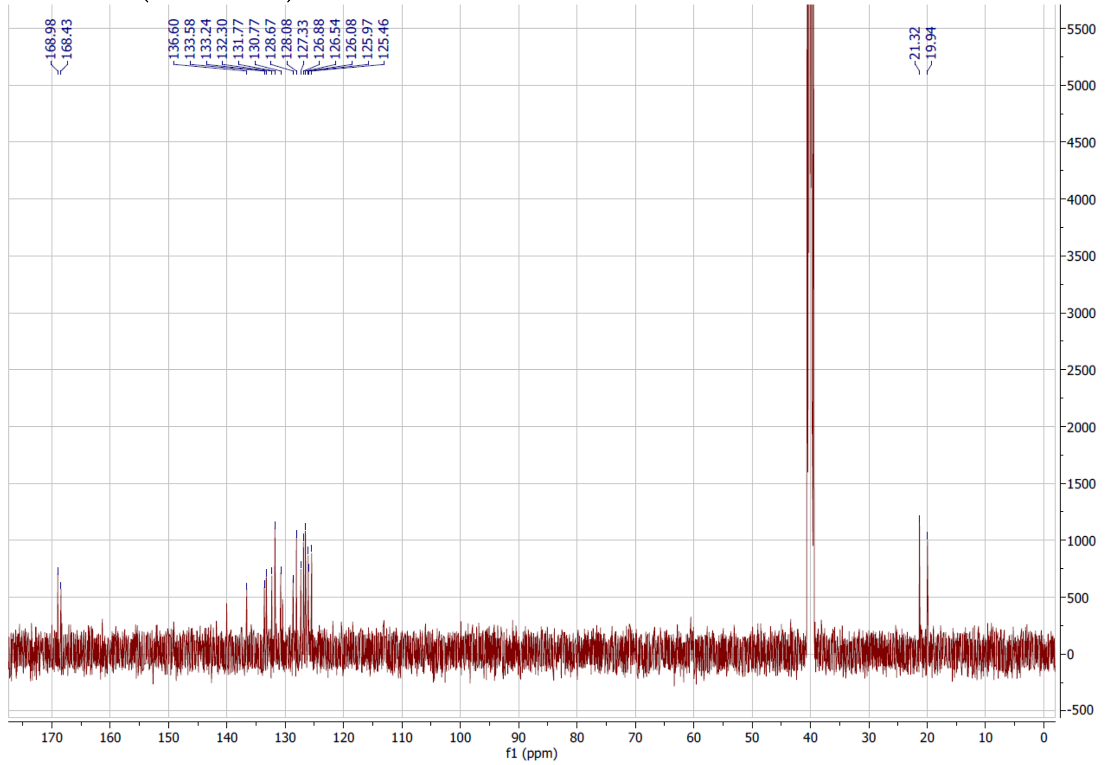

N′-(3,4-Dichlorobenzoyl)-2,4-dimethylbenzohydrazide, **52**

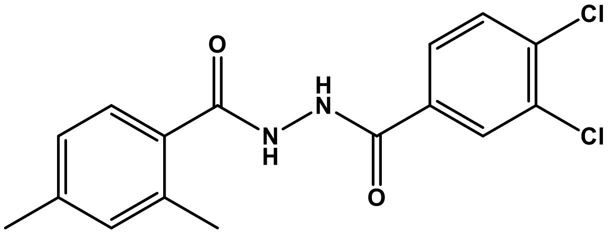

^{1}H-NMR (DMSO-d6):

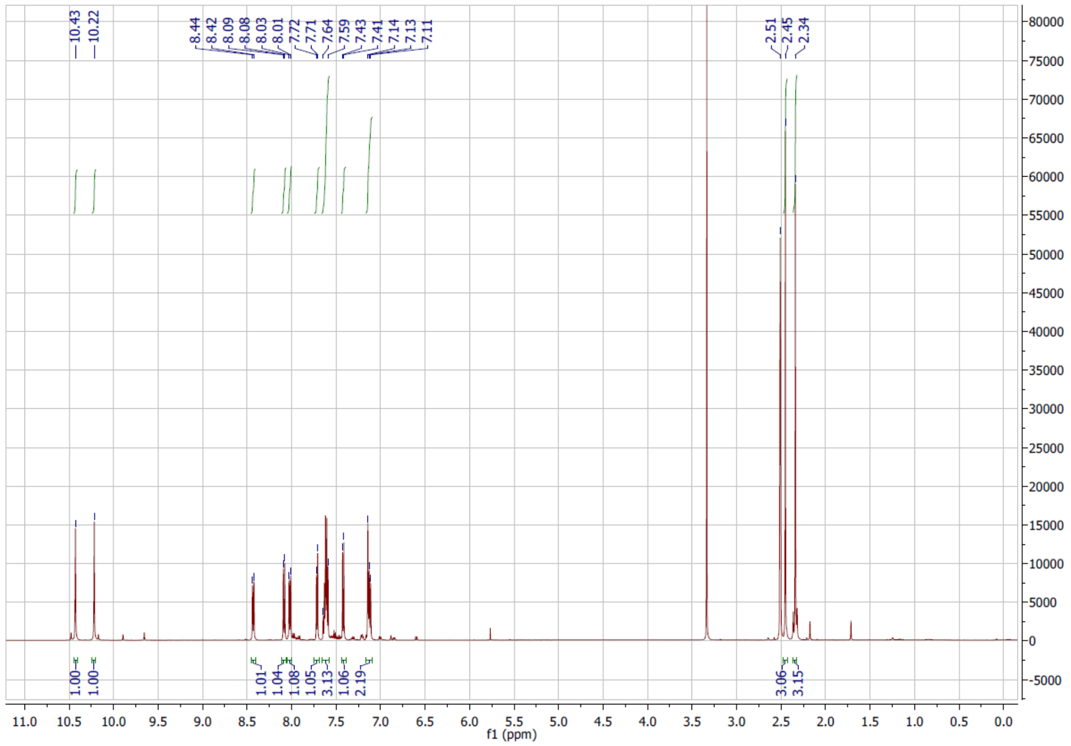

^{13}C-NMR (DMSO-d6):

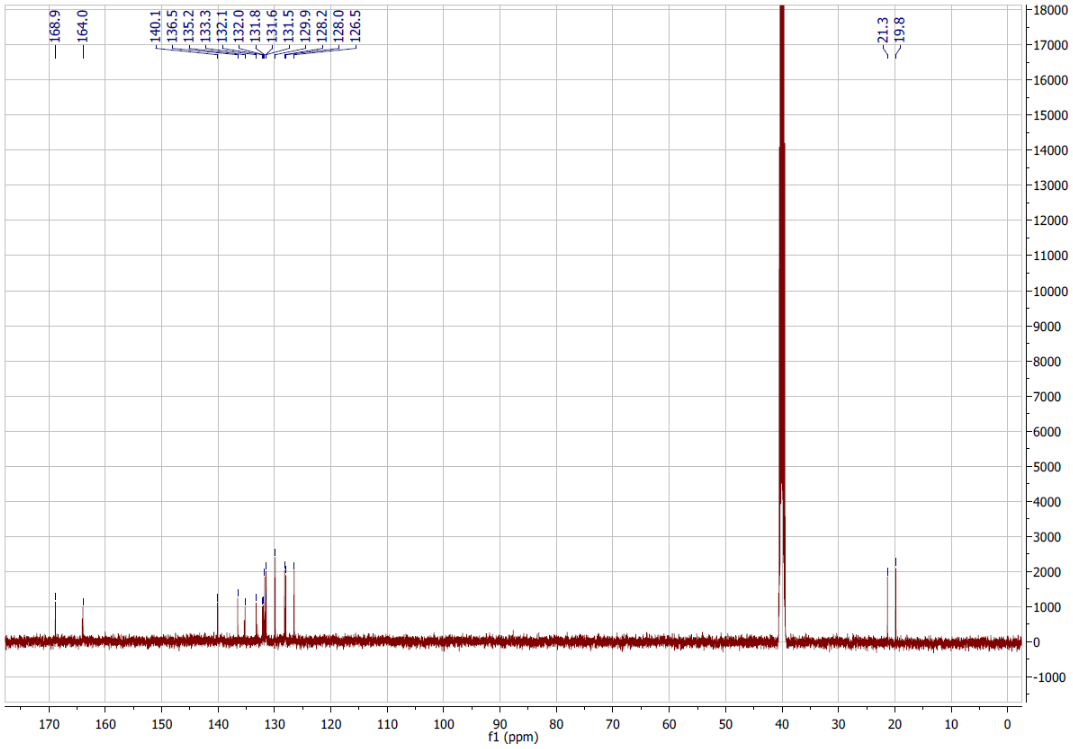

N′-(3,4-Dichlorobenzoyl)-2-methyl-4-(1*H*-tetrazol-5-yl)benzohydrazide, **74**

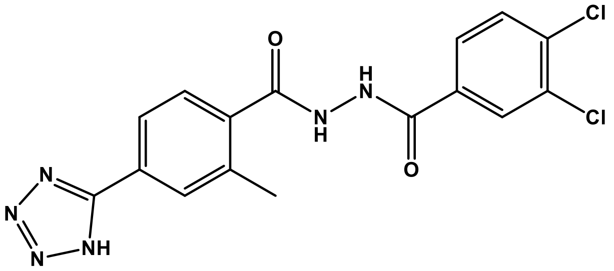

^{1}H-NMR (DMSO-d6):

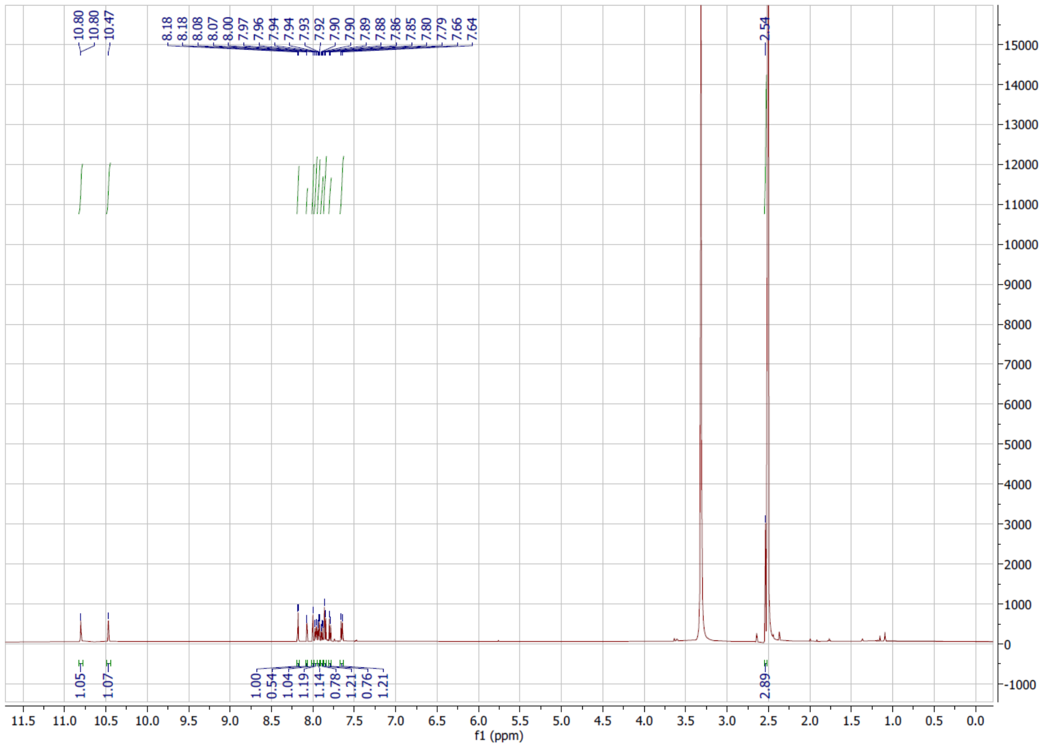

^{13}C-NMR (DMSO-d6):

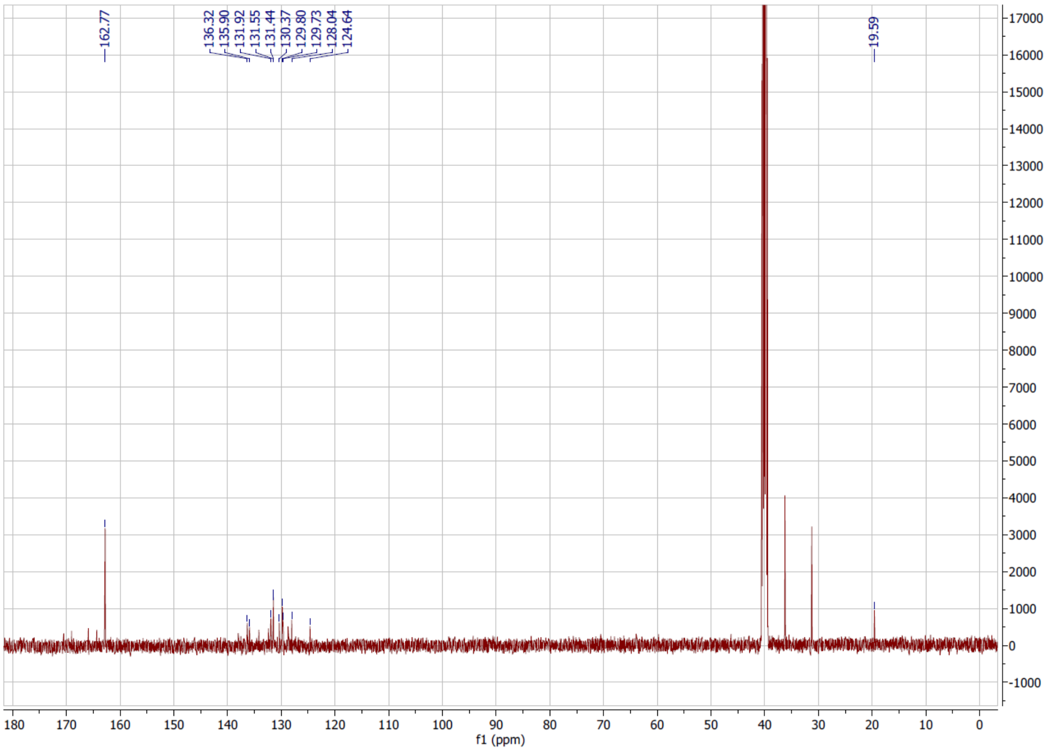

3,4-Dichlorobenzoyl azide, **78**

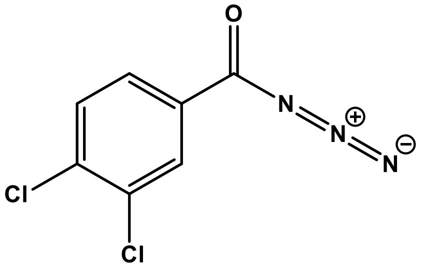

^{1}H-NMR ($CDCl_3$):

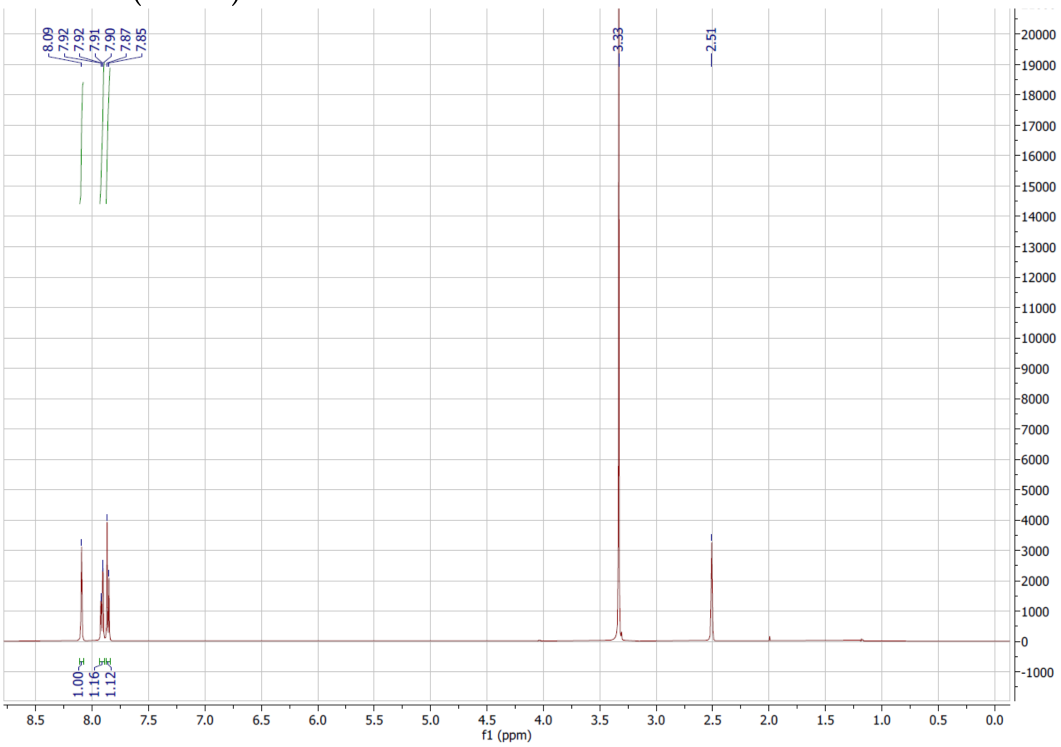

^{13}C-NMR ($CDCl_3$):

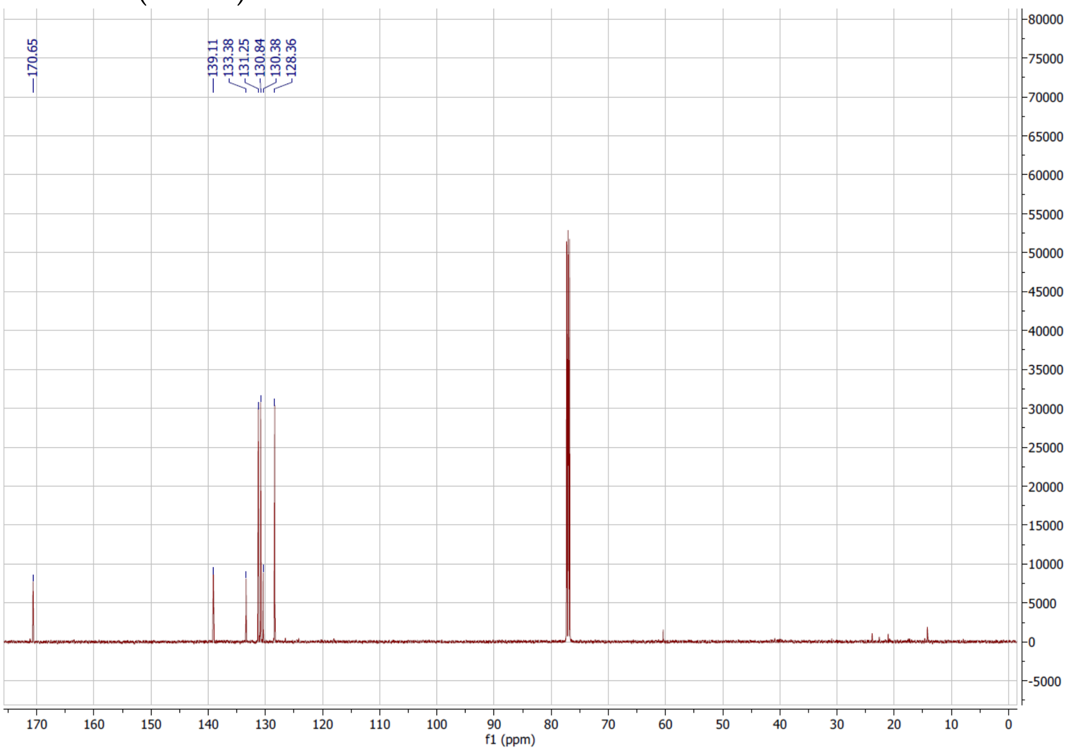

N-(2,4-Dimethylphenyl)-3-(naphthalen-1-yl)triaz-2-ene-1-carboxamide, **82**

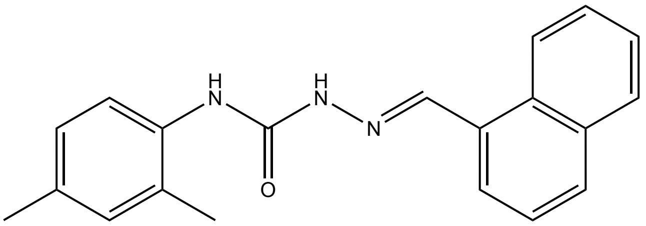

^{1}H-NMR (DMSO-d$_6$):

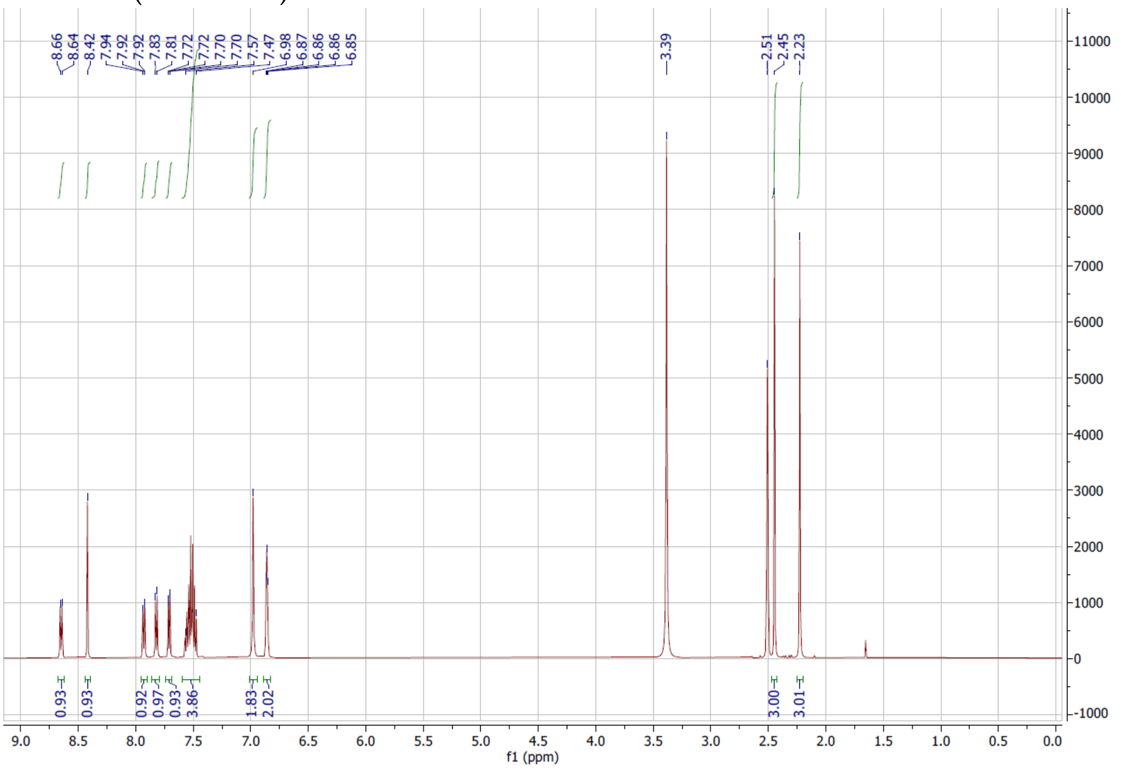

^{13}C-NMR (DMSO-d$_6$):

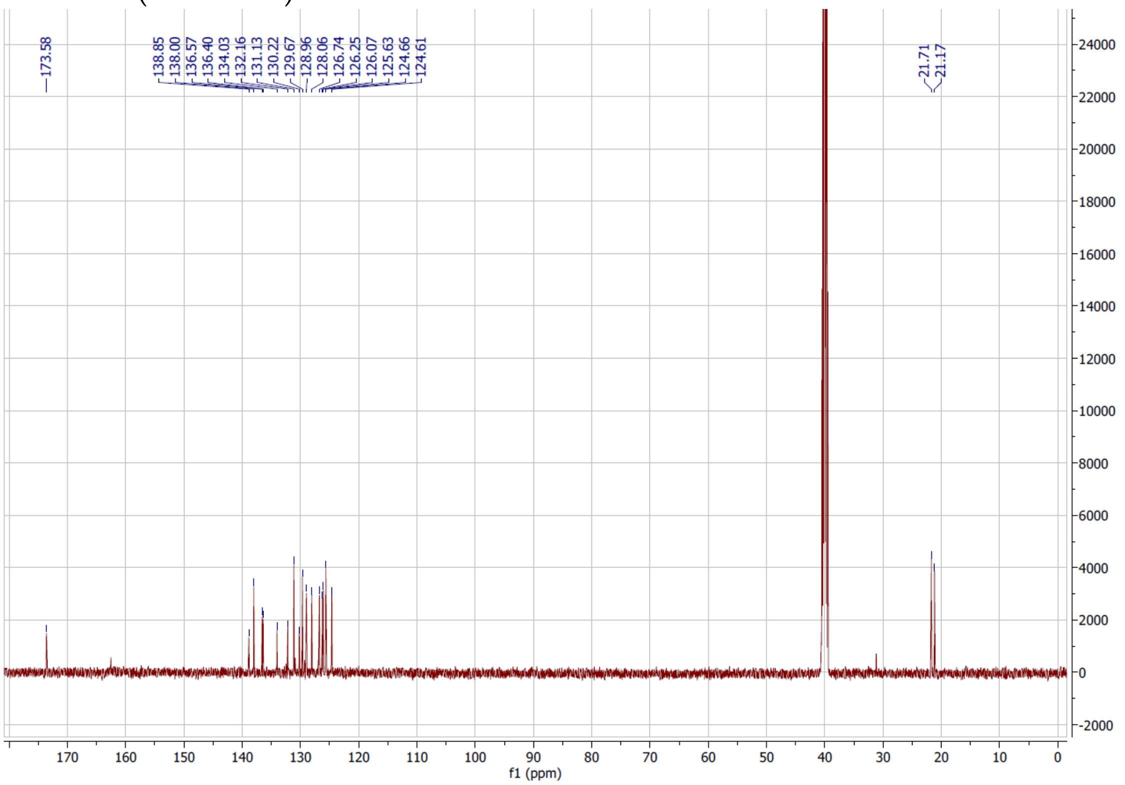

1-(3,4-Dichlorophenyl)-3-(2,4-dimethylphenyl)urea, **88**

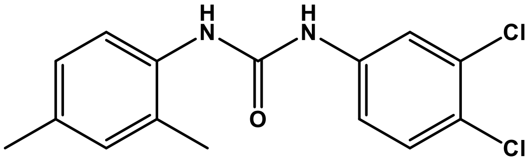

^{1}H-NMR (DMSO-d$_6$):

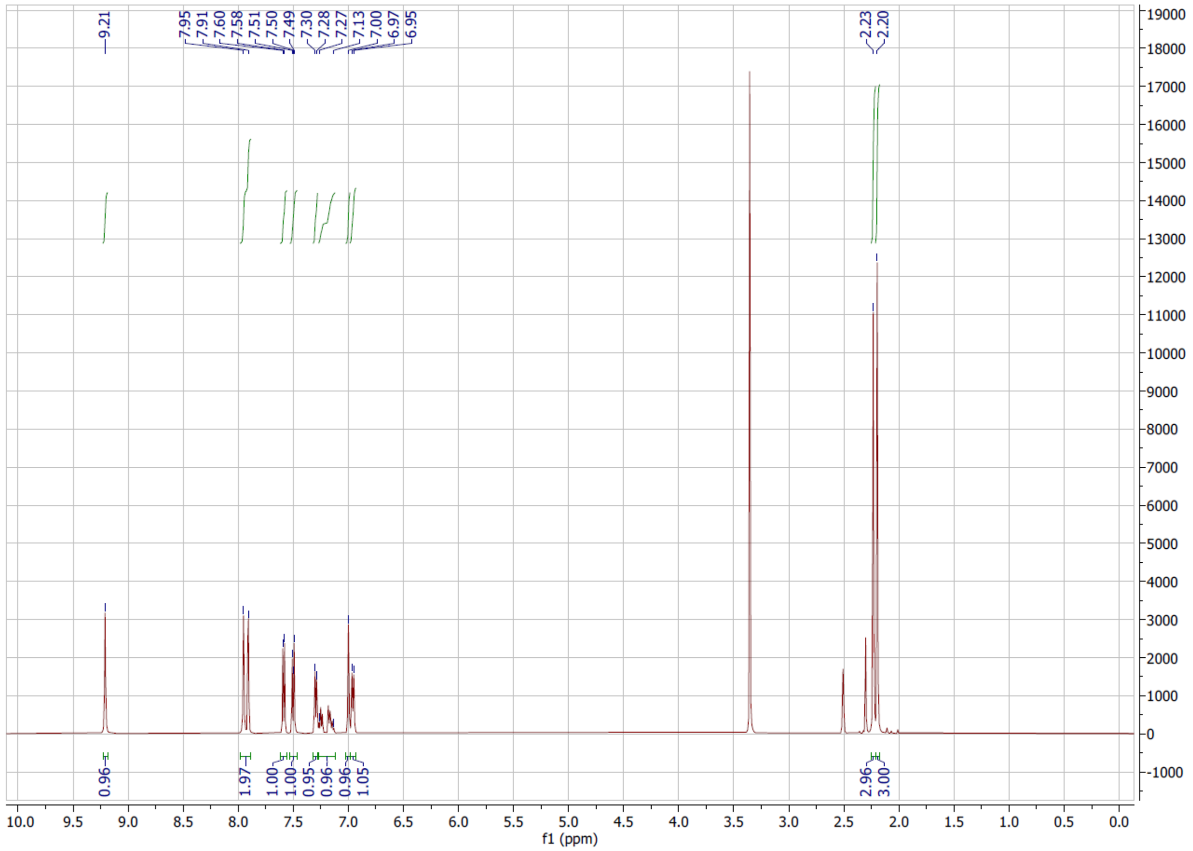

^{13}C-NMR (DMSO-d$_6$):

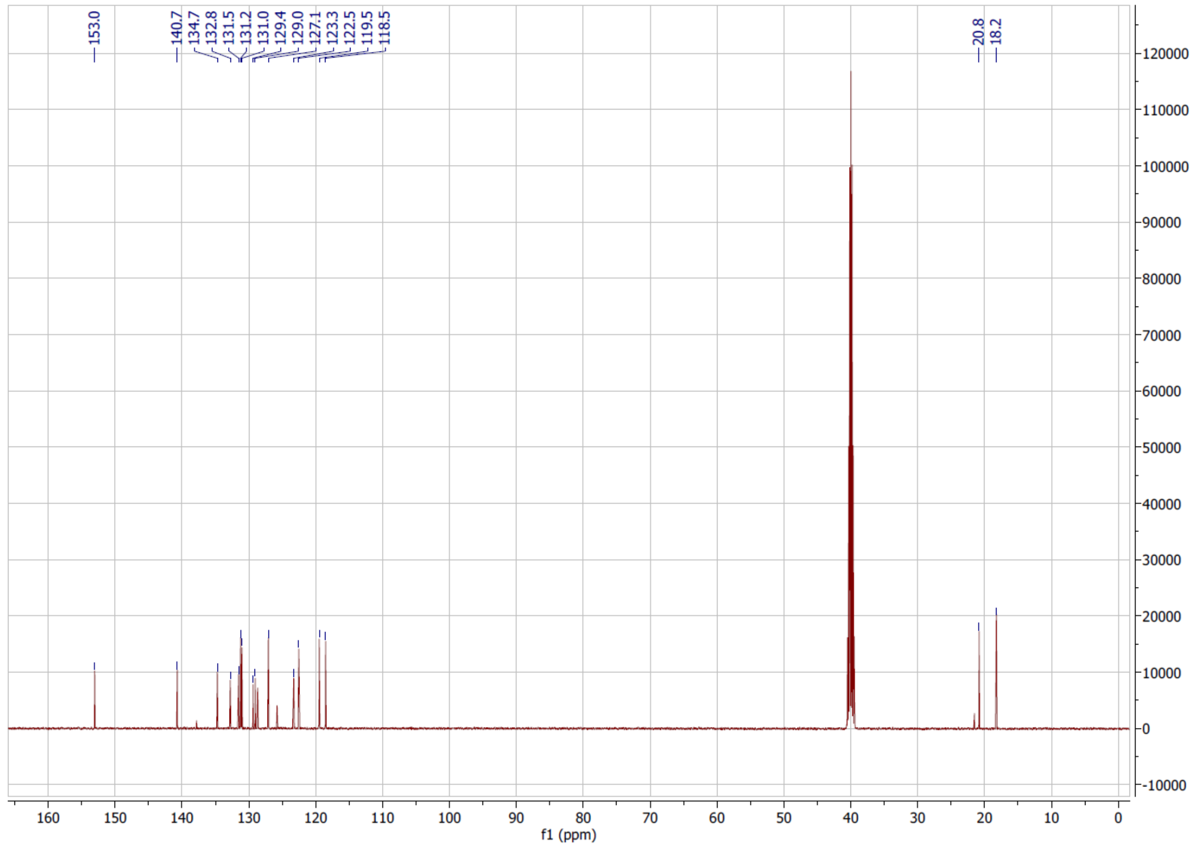

2-((2,4-Dimethylphenyl)amino)-2-oxoacetic acid, **90**

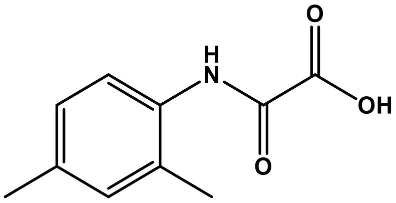

^{1}H-NMR ($CDCl_3$):

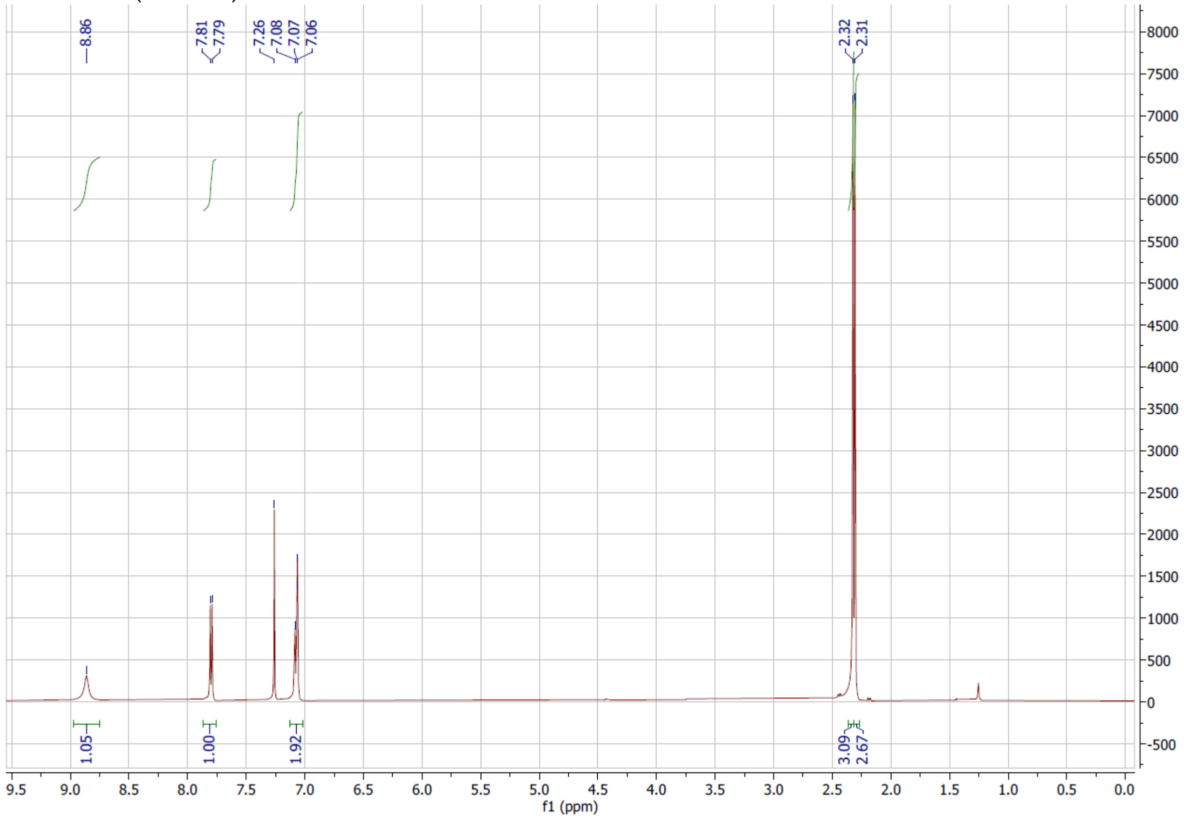

^{13}C-NMR ($CDCl_3$):

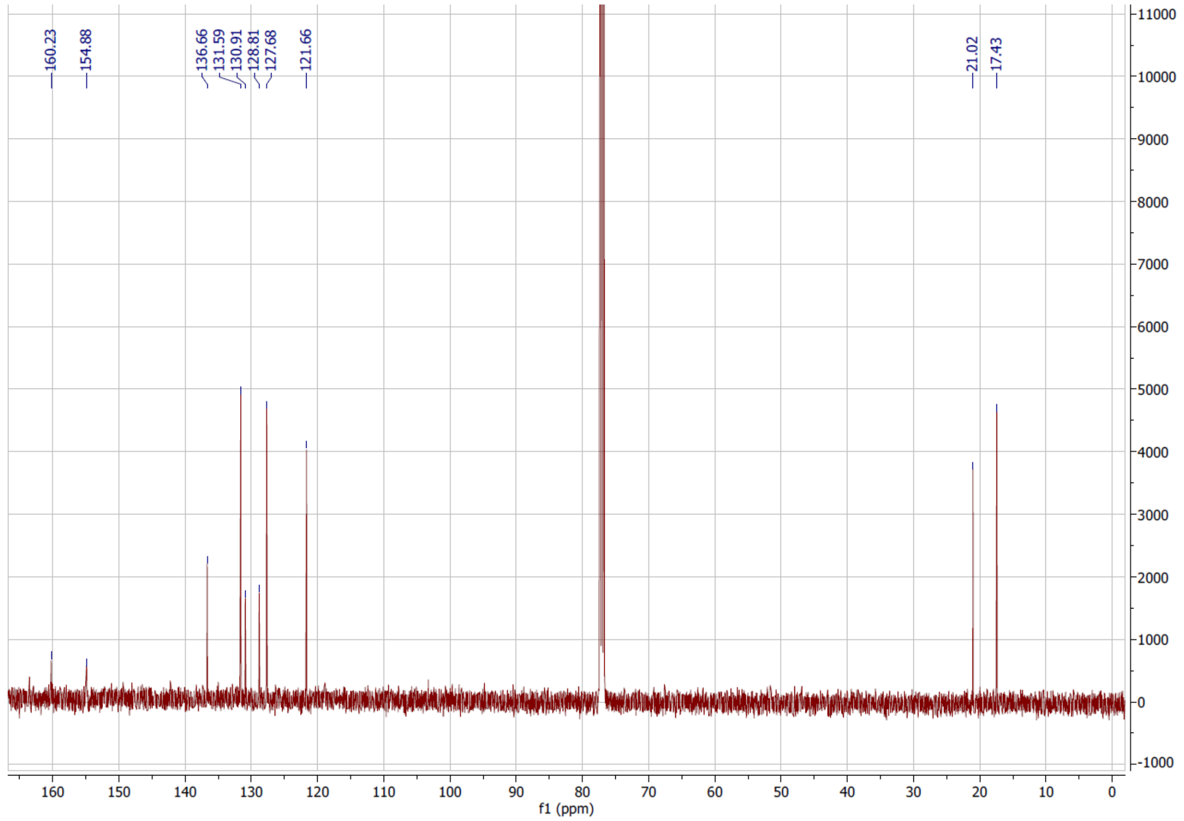

N[1]-(3,4-Dichlorophenyl)-*N*[2]-(2,4-dimethylphenyl)oxalamide, **91**

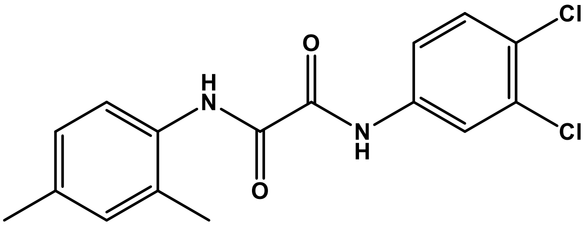

^{1}H-NMR ($CDCl_3$):

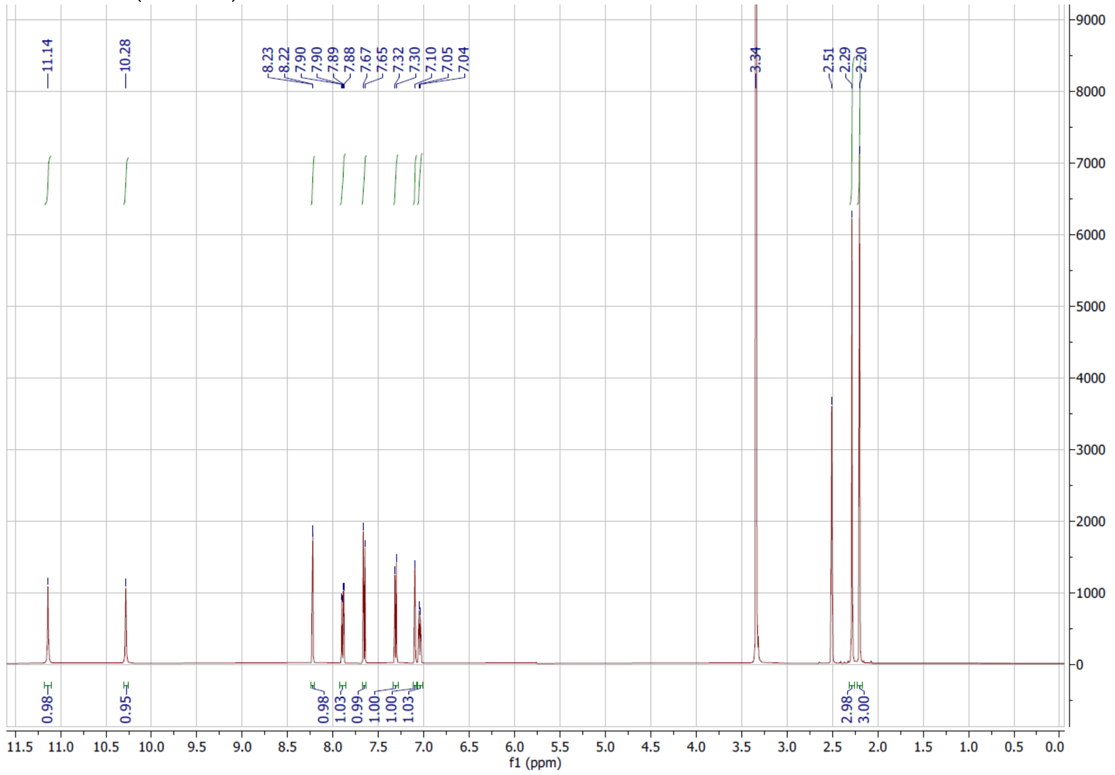

^{13}C-NMR ($CDCl_3$):

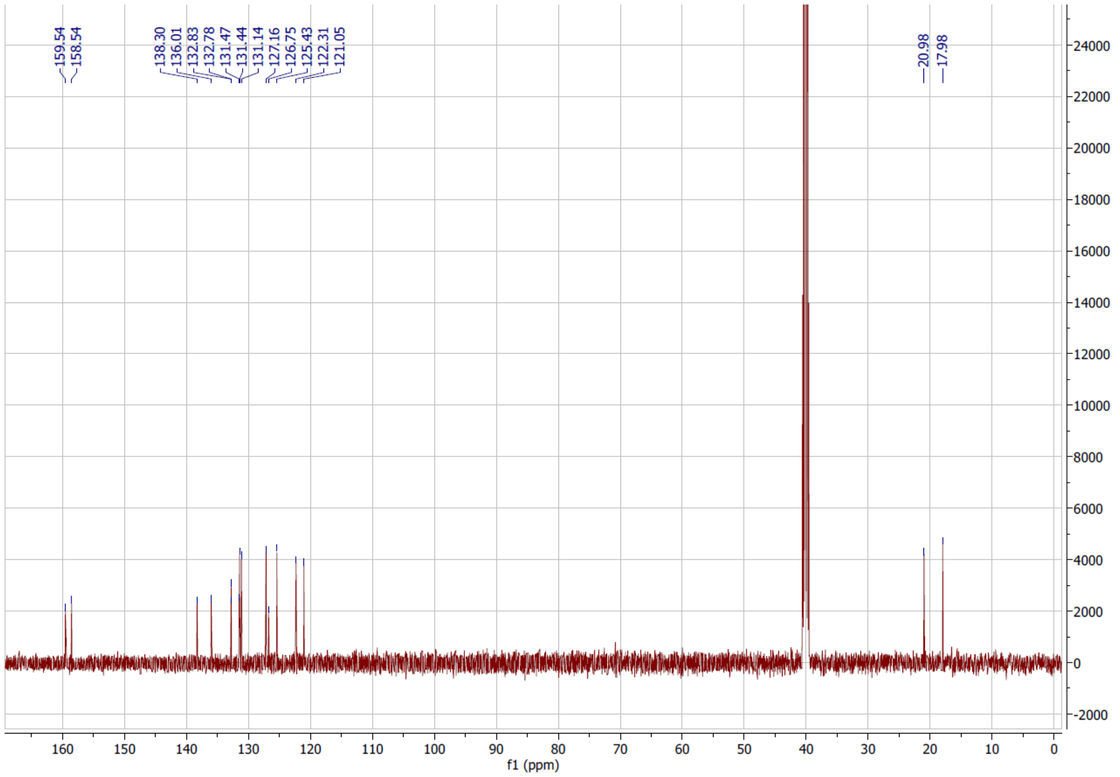

(*E*)-*N*-(3,4-Dichlorobenzyl)-3-(2,4-dimethylphenyl)acrylamide, **96**

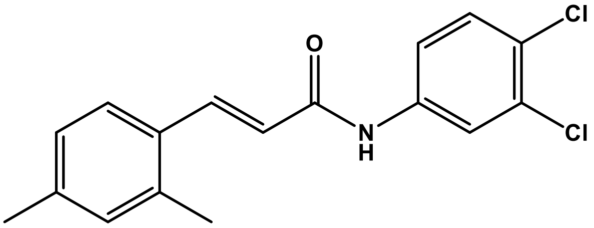

^{1}H-NMR ($CDCl_3$):

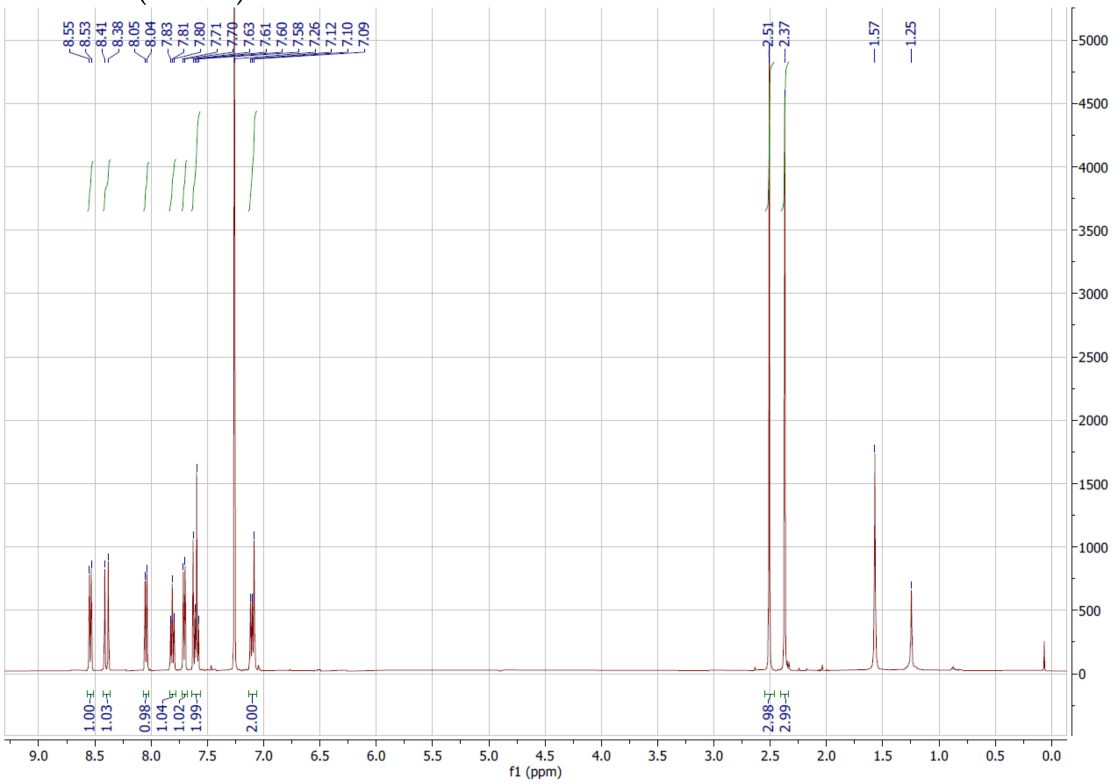

^{13}C-NMR ($CDCl_3$):

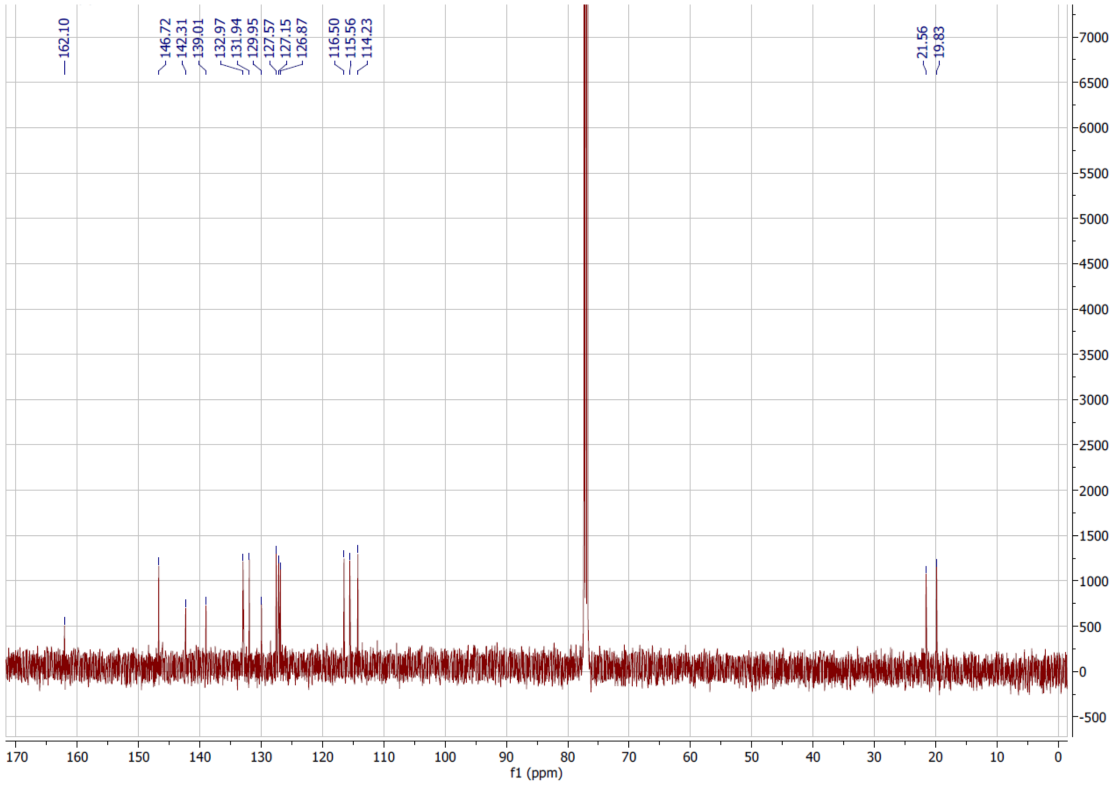

(E)-3-(3,4-Dichlorophenyl)-*N*-(2,4-dimethylphenyl)acrylamide, **98**

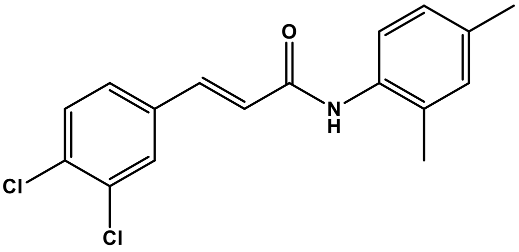

^{1}H-NMR (DMSO-d$_6$):

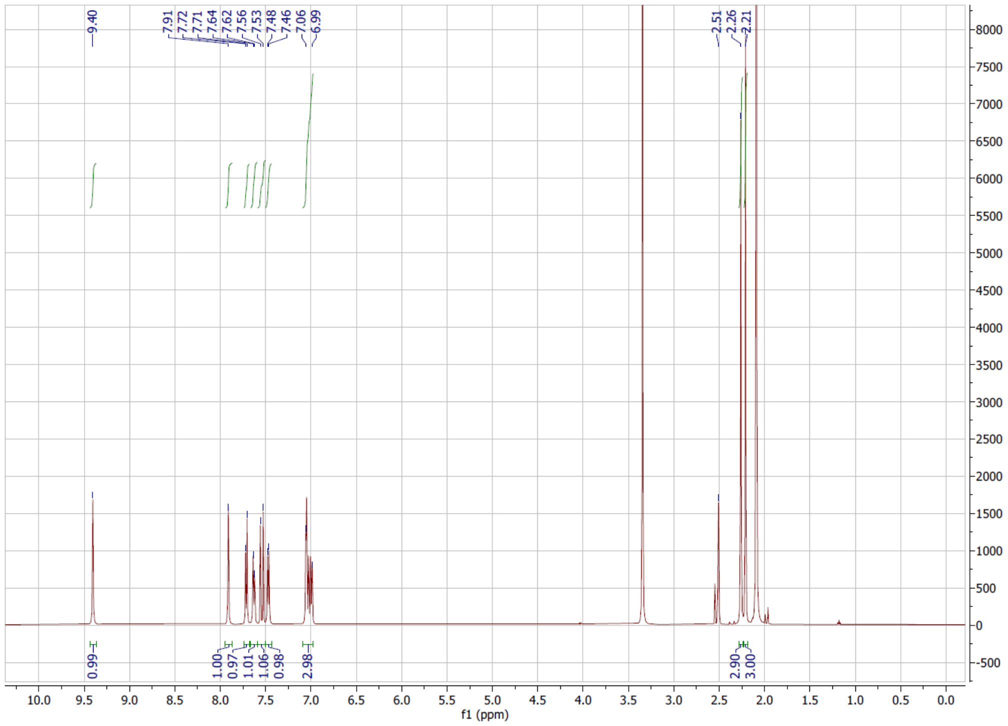

^{13}C-NMR (DMSO-d$_6$):

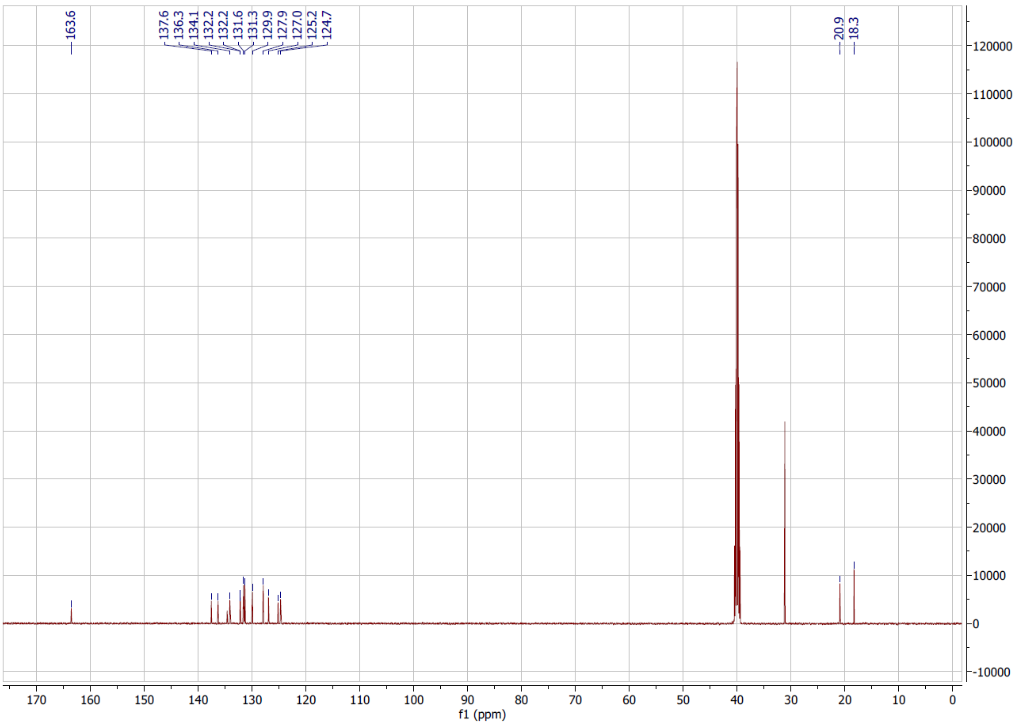

(E)-3-(3,4-Dichlorophenyl)-*N*-(2,4-dimethylbenzyl)acrylamide, **99**

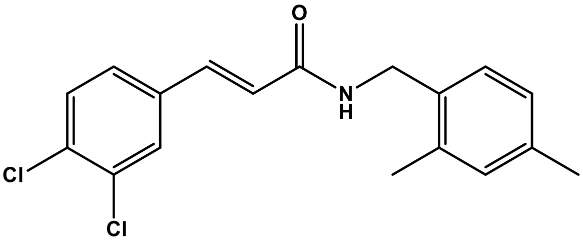

^{1}H-NMR (DMSO-d_6):

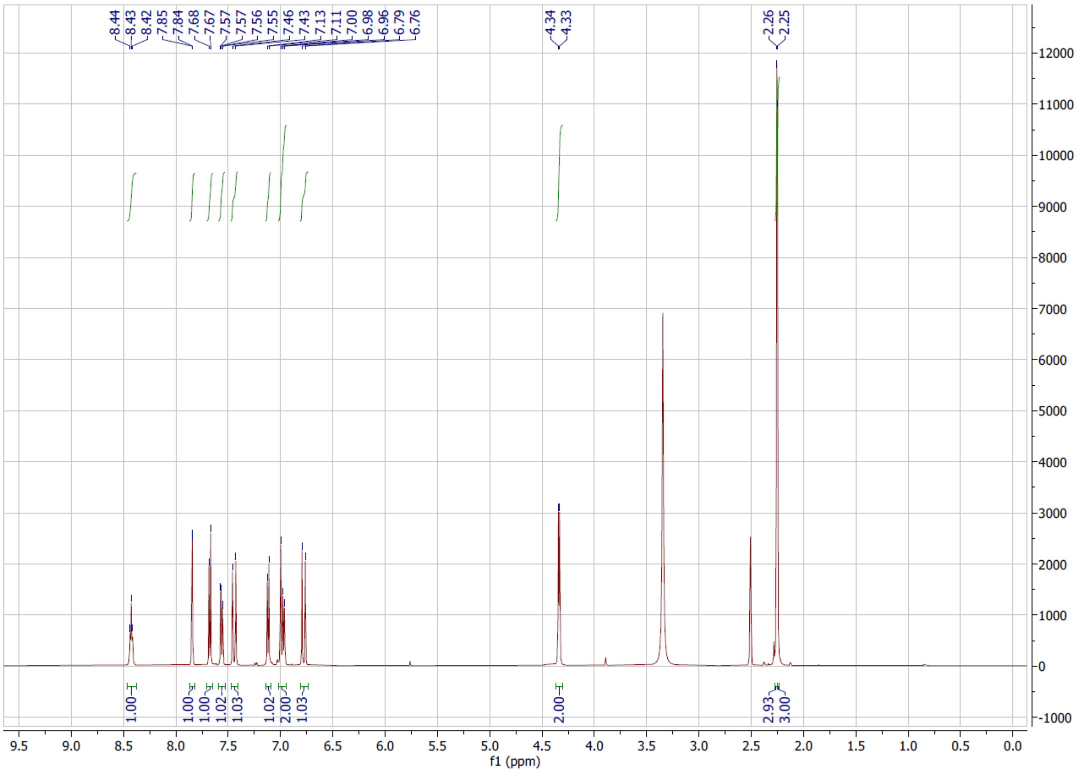

^{13}C-NMR (DMSO-d$_6$):

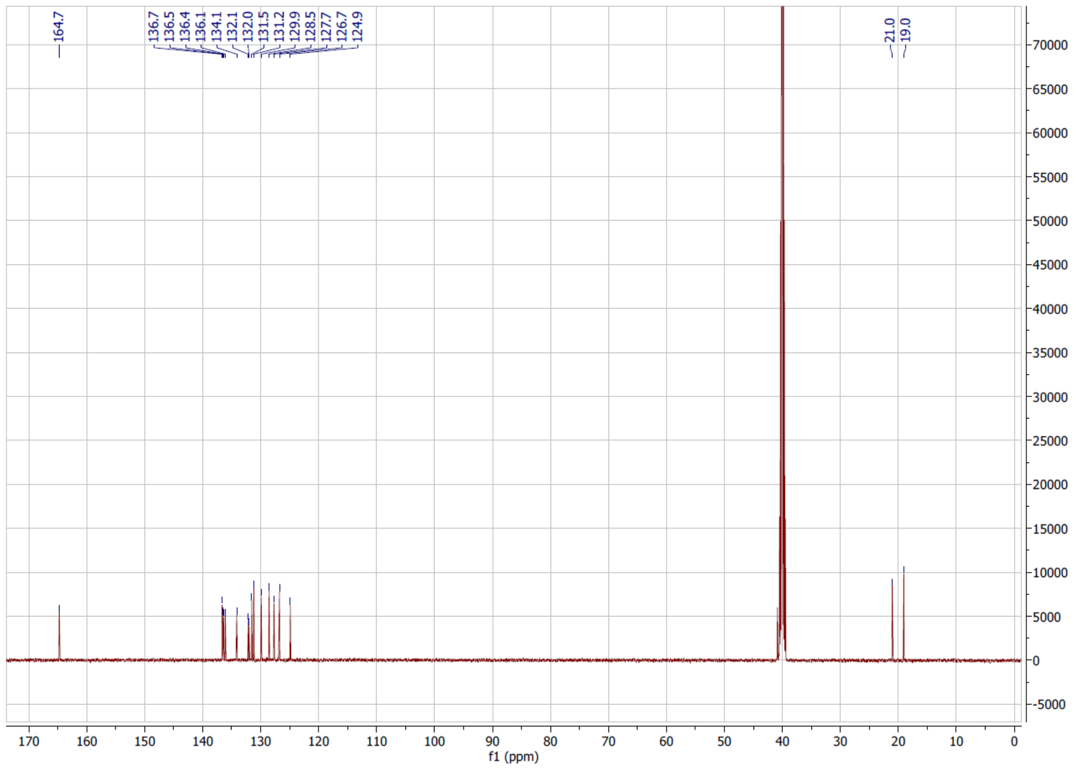

(E)-3-(2,4-Dimethylphenyl)-*N*-((1R,3S)-4-fluorobicyclo[1.1.1]pentan-2-yl)acrylamide, **101**

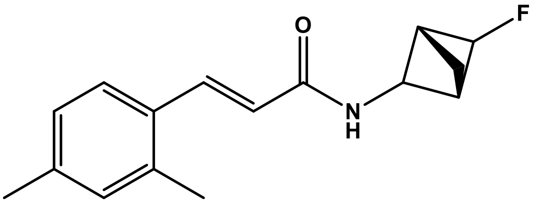

^{1}H-NMR (DMSO-d$_6$):

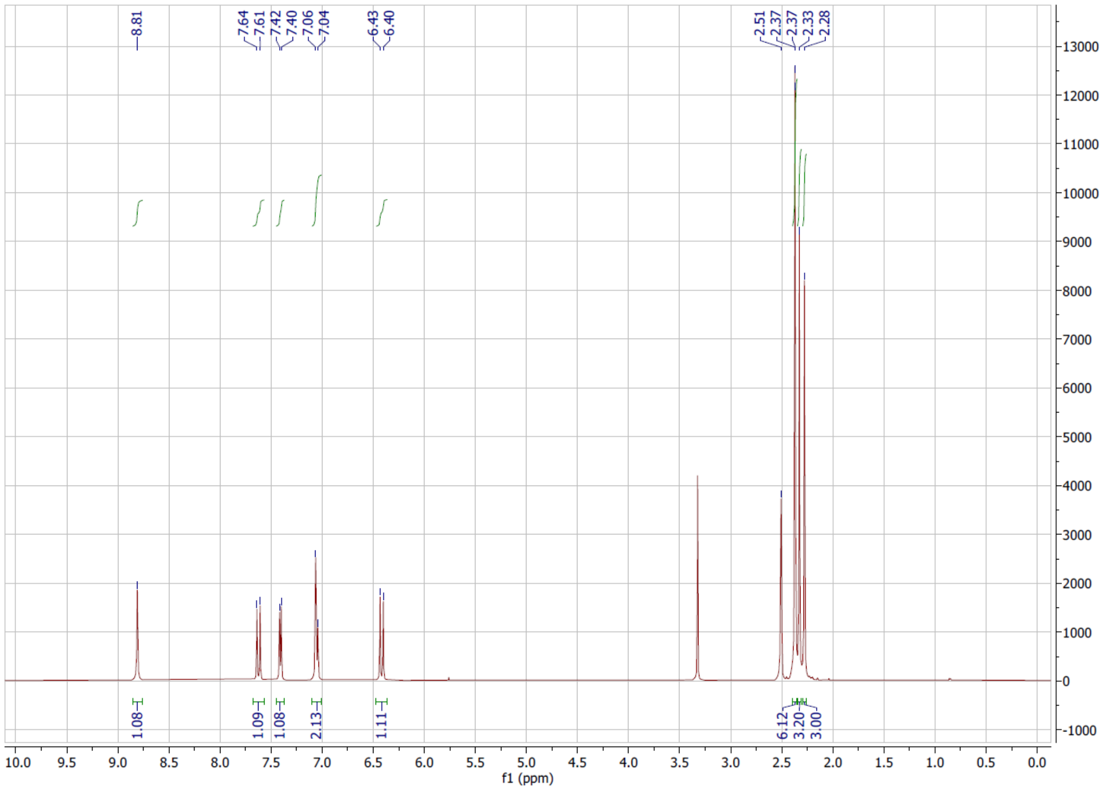

^{13}C-NMR (DMSO-d$_6$):

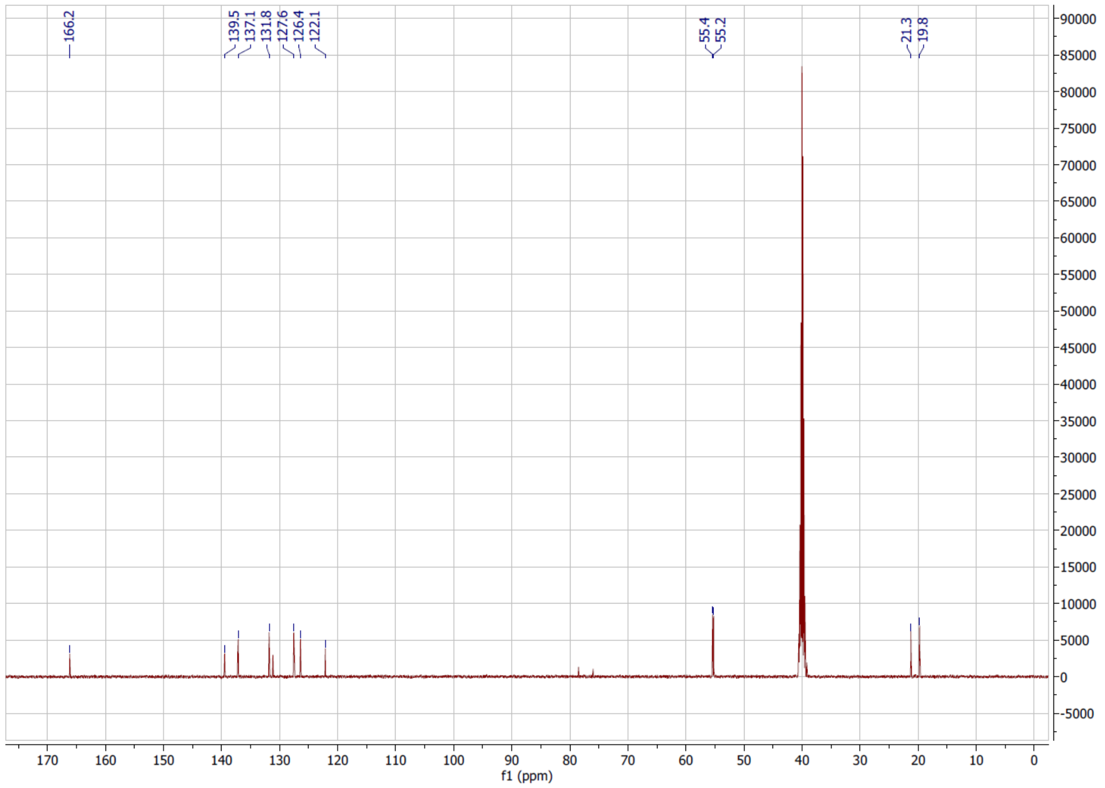

^{19}F-NMR (DMSO-d$_6$):

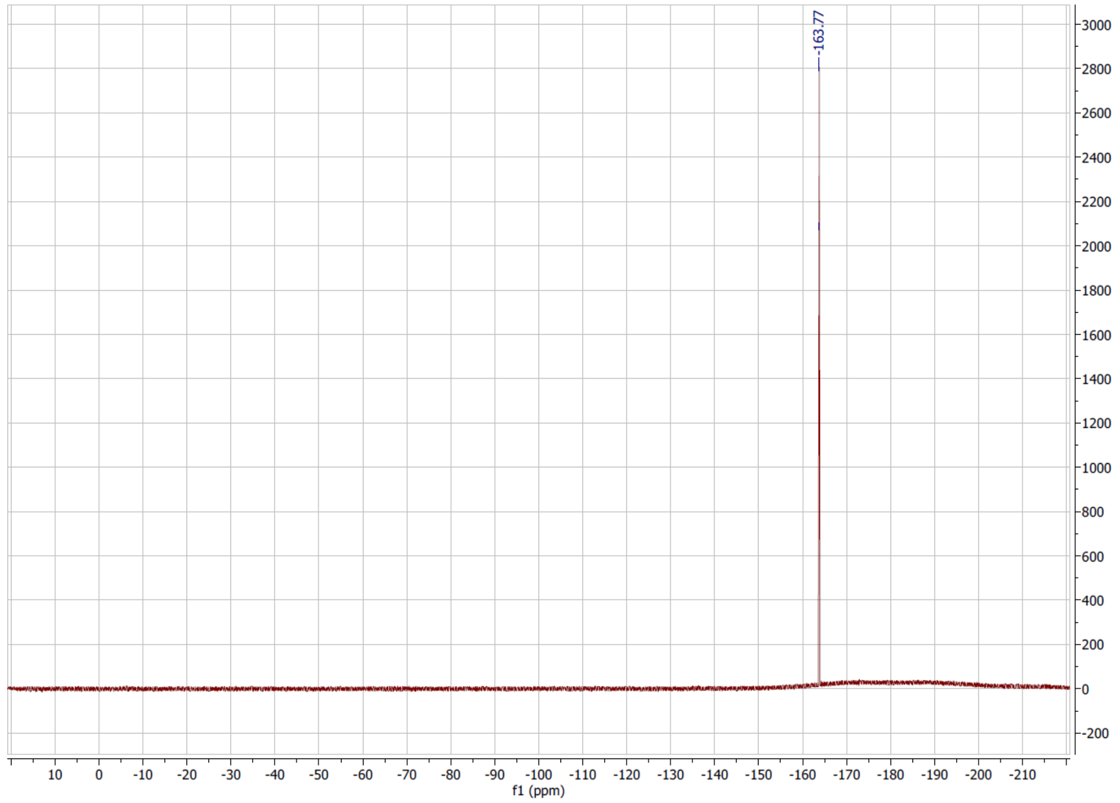

N-(5,6-Dichloro-1*H*-benzo[*d*]imidazol-2-yl)-2,4-dimethylbenzamide, **103**

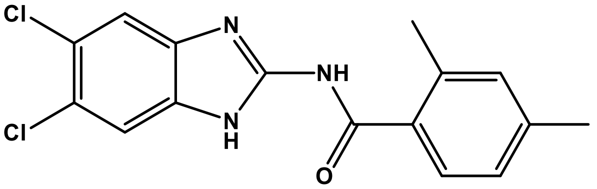

^{1}H-NMR (DMSO-d$_6$):

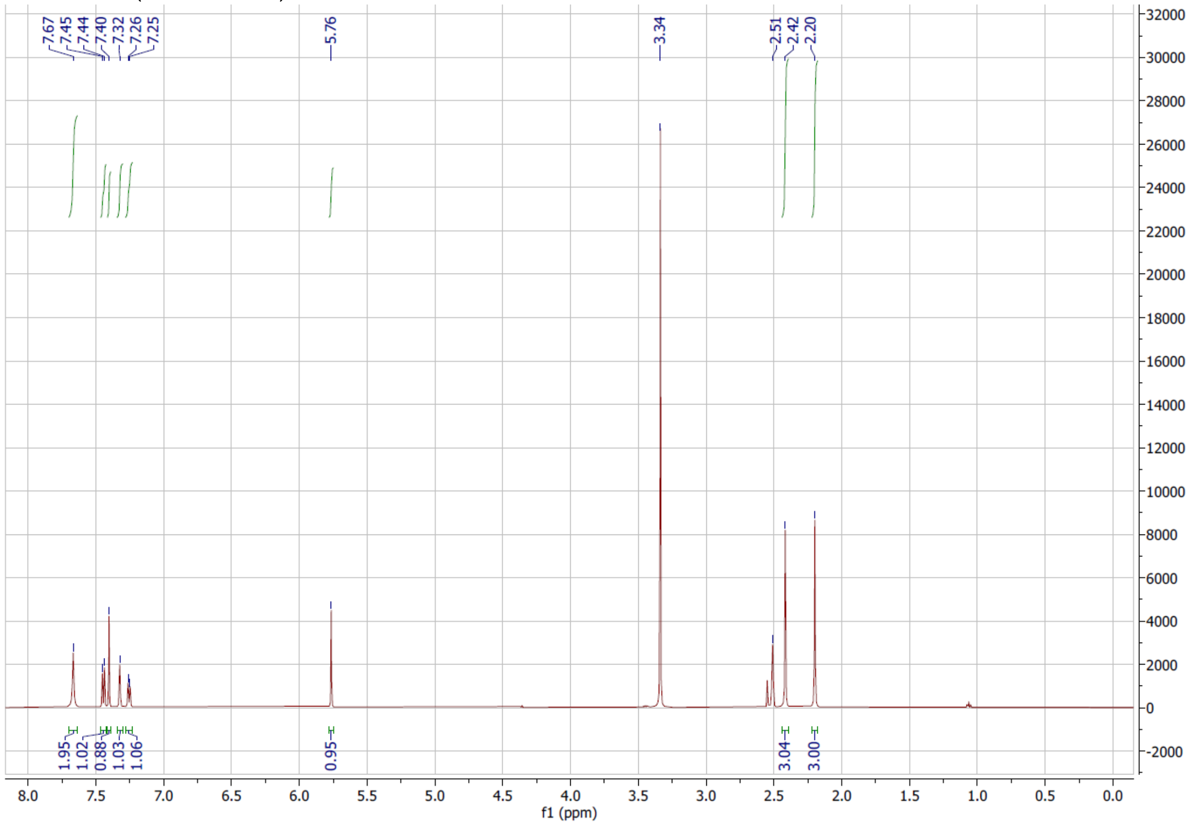

^{13}C-NMR (DMSO-d$_6$):

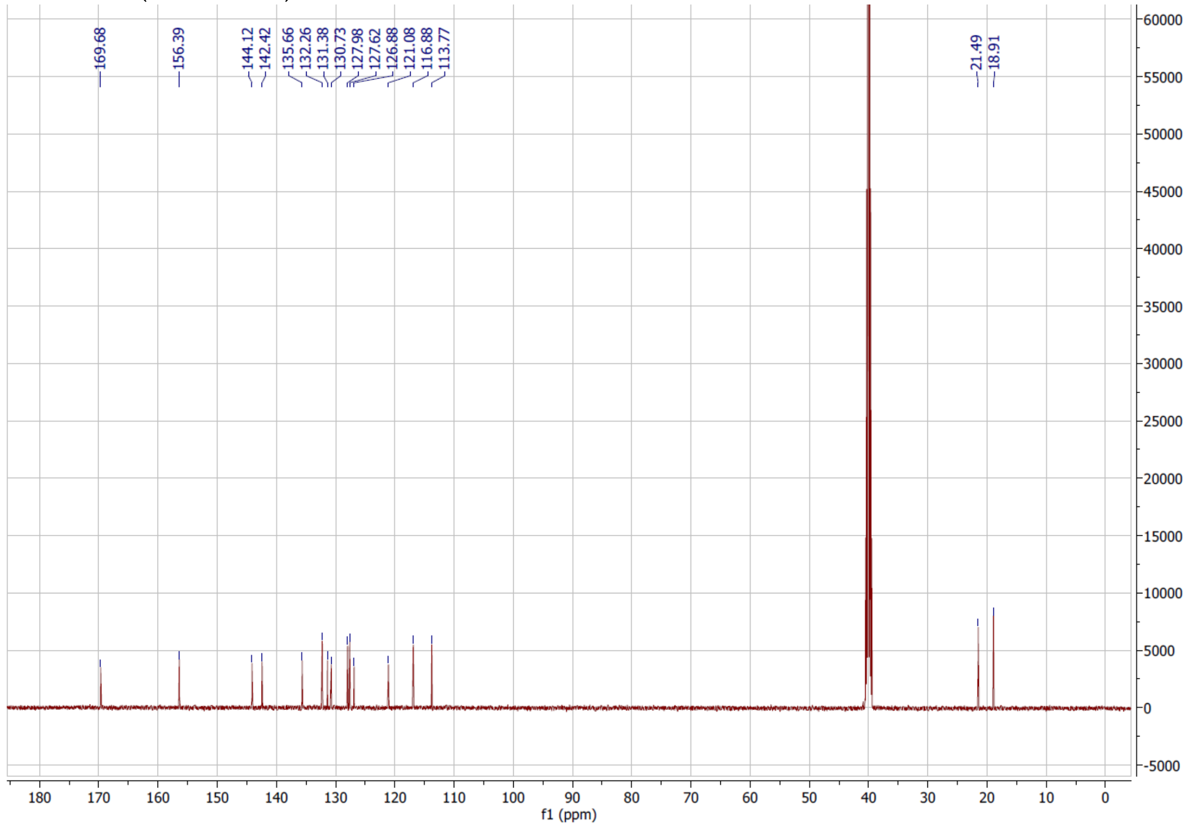

2-(3,4-Dichlorophenyl)-5-(2,4-dimethylphenyl)-1,3,4-oxadiazole, **105**

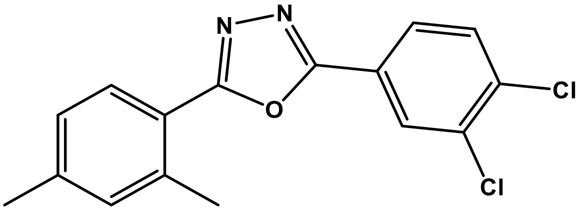

^{1}H-NMR ($CDCl_3$):

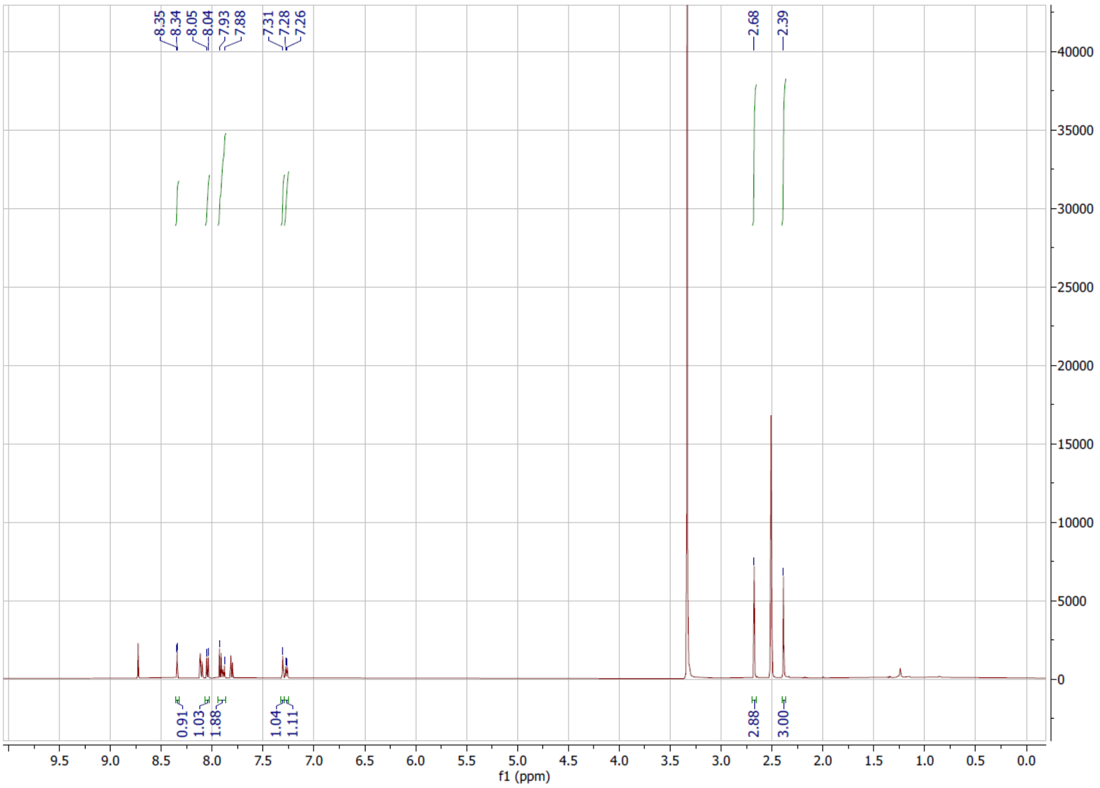

^{13}C-NMR ($CDCl_3$):

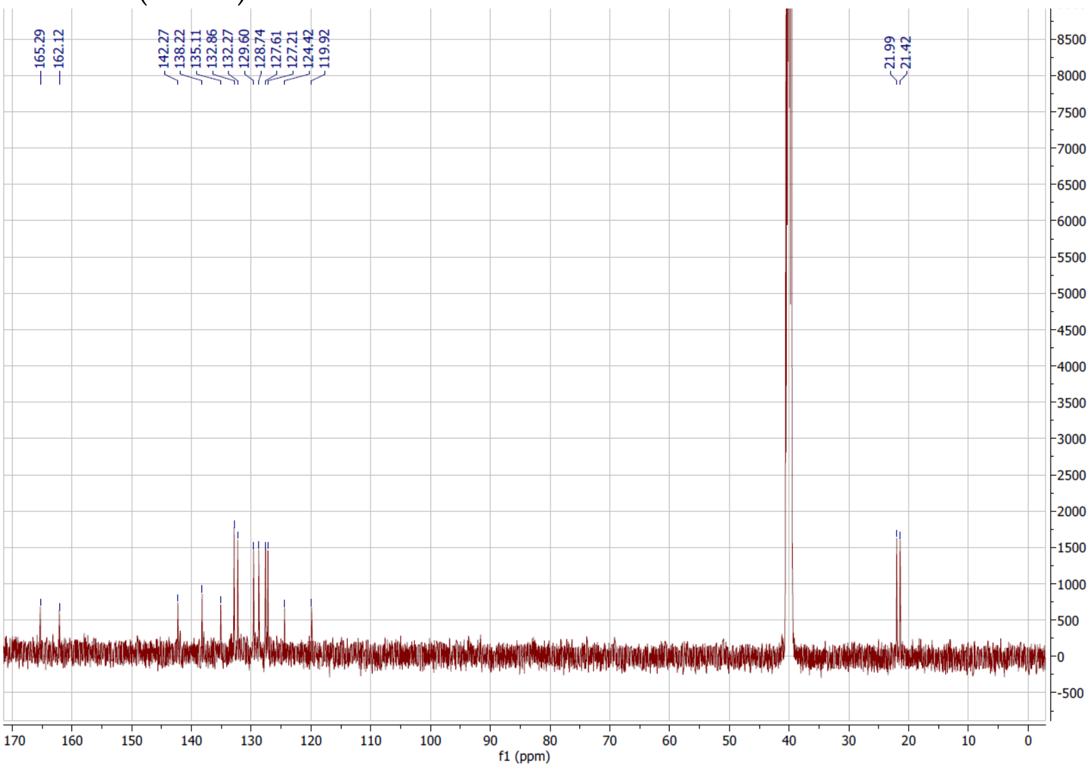

4-(3,4-Dichlorophenyl)-1-(2,4-dimethylbenzyl)-1*H*-1,2,3-triazole, **107**

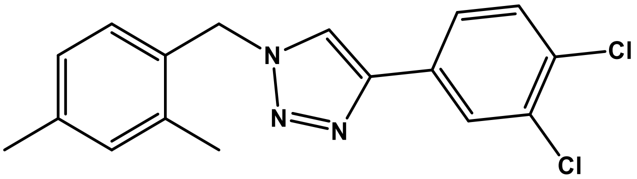

^{1}H-NMR (CDCl$_3$):

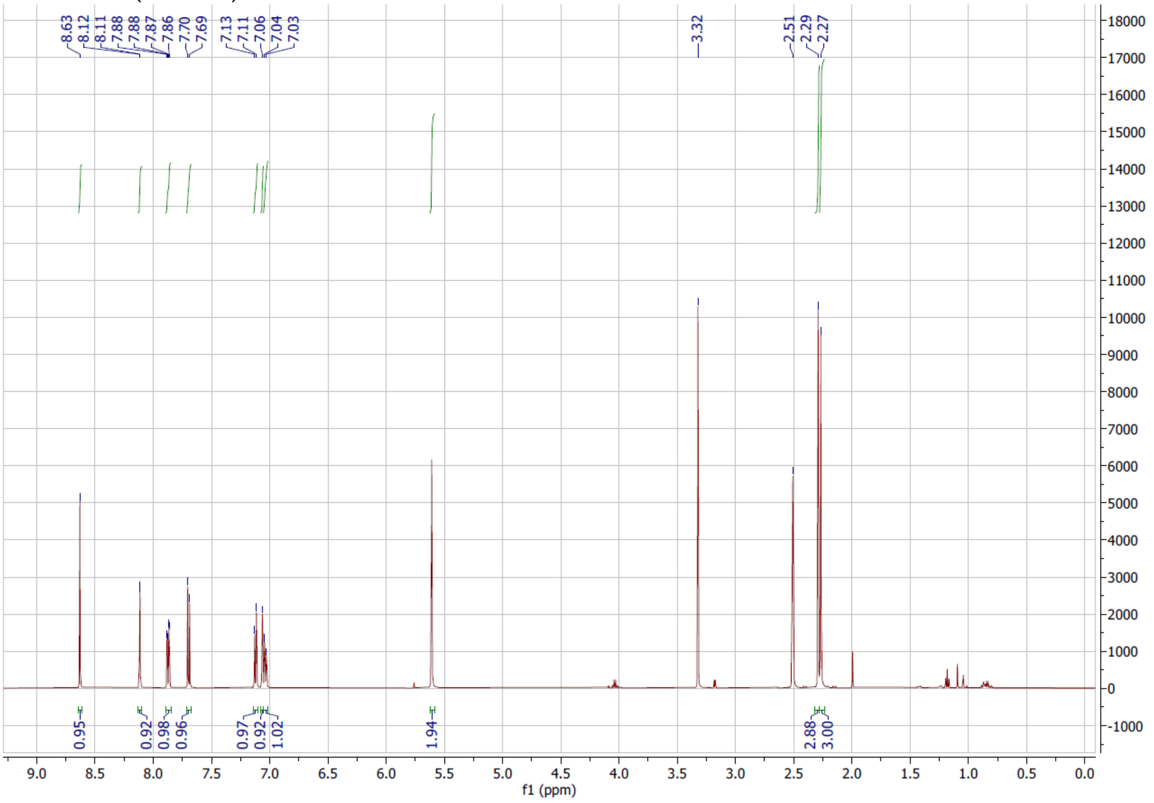

^{13}C-NMR (CDCl$_3$):

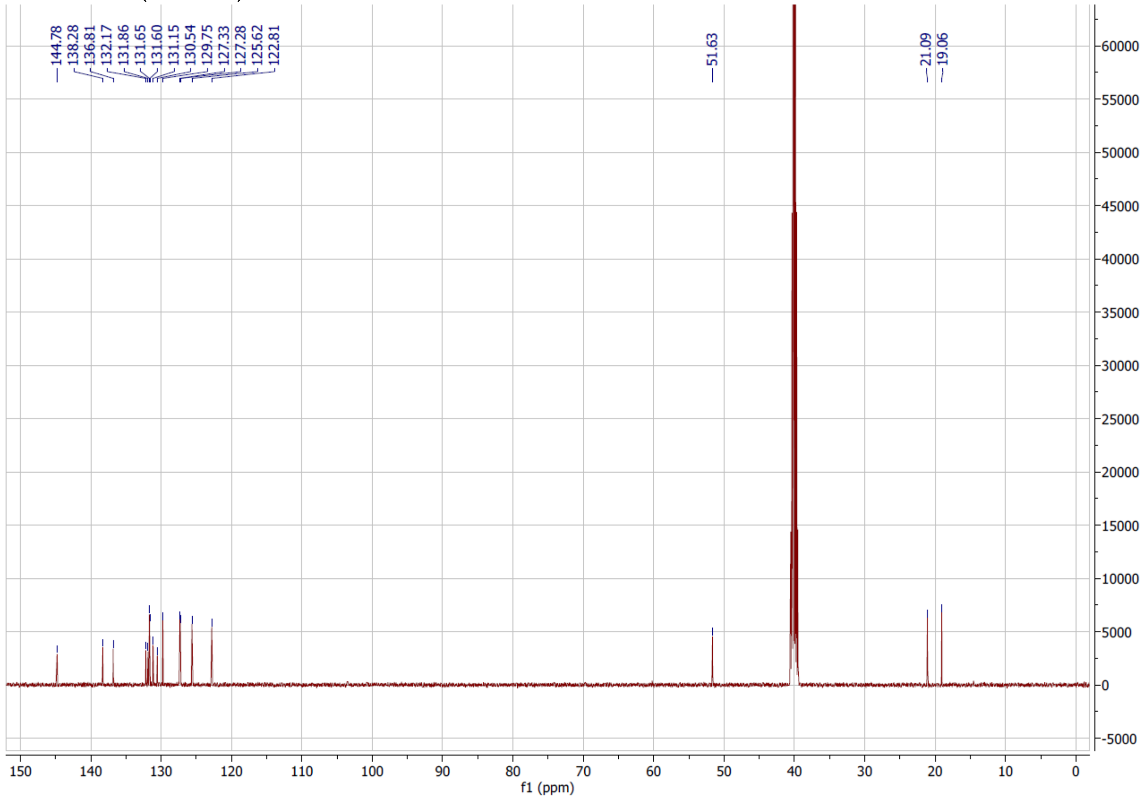

References

(1) Mendenhall, M.; Russell, A.; Juelich, T.; Messina, E. L.; Smee, D. F.; Freiberg, A. N.; Holbrook, M. R.; Furuta, Y.; de la Torre, J. C.; Nunberg, J. H.; Gowen, B. B. T-705 (Favipiravir) Inhibition of Arenavirus Replication in Cell Culture. *Antimicrob. Agents Chemother.* **2011**, *55* (2), 782-787. DOI: 10.1128/aac.01219-10.

(2) Trepanier, D. J.; Ure, D. R.; Foster, R. T. In Vitro Phase I Metabolism of CRV431, a Novel Oral Drug Candidate for Chronic Hepatitis B. *Pharmaceutics* **2017**, *9* (4), 51. DOI: 10.3390/pharmaceutics9040051.

(3) Ling, A.; Hong, Y.; Gonzalez, J.; Gregor, V.; Polinsky, A.; Kuki, A.; Shi, S.; Teston, K.; Murphy, D.; Porter, J.; Kiel, D.; Lakis, J.; Anderes, K.; May, J.; Knudsen, L. B.; Lau, J. Identification of Alkylidene Hydrazides as Glucagon Receptor Antagonists. *J. Med. Chem.* **2001**, *44* (19), 3141-3149. DOI: 10.1021/jm000547o.

(4) van Dijken, D. J.; Kovaříček, P.; Ihrig, S. P.; Hecht, S. Acylhydrazones as Widely Tunable Photoswitches. *J. Am. Chem. Soc.* **2015**, *137* (47), 14982-14991. DOI: 10.1021/jacs.5b09519.

(5) Chen, C.; Dolla, N. K.; Casadei, G.; Bremner, J. B.; Lewis, K.; Kelso, M. J. Diarylacylhydrazones: Clostridium-selective antibacterials with activity against stationary-phase cells. *Bioorg. Med. Chem. Lett.* **2014**, *24* (2), 595-600. DOI: 10.1016/j.bmcl.2013.12.015.

(6) Demurtas, M.; Baldisserotto, A.; Lampronti, I.; Moi, D.; Balboni, G.; Pacifico, S.; Vertuani, S.; Manfredini, S.; Onnis, V. Indole derivatives as multifunctional drugs: Synthesis and evaluation of antioxidant, photoprotective and antiproliferative activity of indole hydrazones. *Bioorg. Chem.* **2019**, *85*, 568-576. DOI: 10.1016/j.bioorg.2019.02.007.

(7) Suresh, D. M.; Sajan, D.; Diao, Y.-P.; Němec, I.; Hubert Joe, I.; Bena Jothy, V. Structural conformations and density functional study on the intramolecular charge transfer based on vibrational spectra of 2,4-dihydroxy-N′-(4-methoxybenzylidene)benzohydrazide. *Spectrochim. Acta, Part A* **2013**, *110*, 157-168. DOI: 10.1016/j.saa.2013.01.067.
